# Supplementary material for: DMRforPairs: identifying Differentially Methylated Regions between unique samples using array based methylation profiles
Source: BMC Bioinformatics. 2014 May 15;15:141. doi: 10.1186/1471-2105-15-141 (PMC4046028; doi:10.1186/1471-2105-15-141)
Supplement: Additional file 2 — DMRforPairs output for the comparison of A431-MCF7 and NA17018-NA17105. Please start from the HTML files in each folder. Available via the BMC Bioinformatics website. [file 1471-2105-15-141-S2.zip › 1394847754114233_MOESM2_ESM/A431_MCF7/significant.html]

DMRforPairs output generated on Tue Nov 19 17:07:20 2013. Identified regions were set to contain at least 4 probes with a maximum distance of 200 bp between individual probes (n=29404). Regions in which median methylation levels (M-values) between the samples differed at least |1.4| (n=9744) (=relevant) were tested for statistical significance (significant: p<0.05; multiple testing adjusted, n=2626). The folowing samples were studied: A431,MCF7. Chromosomal positions are indicated in bp. N indicates the number of probes in a region. ID indicates the region ID. Values in the columns per sample indicate median beta values. dM indicates the largest median difference (absolute) between any of the sample pairs. p indicates uncorrected p-value from Mann-Whitney U test (n=2) or Kruskal Wallis test (n>2). p.adj denotes the multiple testing corrected p-value (method:fdr). Gene symbols of overlapping transcripts are listed for the exact region and within a margin of 10000 bp of the region.

| Thumbnail | Chr | Start | End | Links | Length | n | ID | Class | A431 | MCF7 | Gene.Symbol | dM | p | p.adj |
| --- | --- | --- | --- | --- | --- | --- | --- | --- | --- | --- | --- | --- | --- | --- |
|  | 16 | 699218 | 699357 | PDFSTATSENSEMBLUCSC | 140 | 5 | 14972 | tss | 0.001474 | 0.989376 | FAM195A; WDR90; AL022341.3 (margin: FAM195A; WDR90; AL022341.1; AL022341.3; LA16c-349E10.1) | 17.96 | 7.94e-03 | 3.19e-02 |
|  | 16 | 699218 | 699500 | PDFSTATSENSEMBLUCSC | 283 | 6 | 15468 | island | 0.003816 | 0.987805 | FAM195A; WDR90; AL022341.3 (margin: FAM195A; WDR90; AL022341.1; AL022341.3; LA16c-349E10.1) | 16.64 | 2.16e-03 | 1.45e-02 |
|  | 7 | 27153636 | 27153944 | PDFSTATSENSEMBLUCSC | 309 | 5 | 37489 | tss | 0.000914 | 0.984478 | HOXA-AS2; HOXA3 (margin: HOXA-AS2; HOXA2; HOXA3) | 15.97 | 7.94e-03 | 3.19e-02 |
|  | 17 | 46682308 | 46682413 | PDFSTATSENSEMBLUCSC | 106 | 6 | 16657 | gene;island | 0.001299 | 0.985588 | HOXB-AS3; HOXB3; HOXB6 (margin: HOXB-AS3; HOXB3; HOXB5; HOXB6; HOXB7; HOXB8) | 15.62 | 2.16e-03 | 1.45e-02 |
|  | 7 | 27153580 | 27153944 | PDFSTATSENSEMBLUCSC | 365 | 7 | 36930 | gene;island | 0.003833 | 0.984256 | HOXA-AS2; HOXA3 (margin: HOXA-AS2; HOXA2; HOXA3) | 15.03 | 5.83e-04 | 6.47e-03 |
|  | 19 | 52074293 | 52074501 | PDFSTATSENSEMBLUCSC | 209 | 8 | 20998 | tss;island | 0.005935 | 0.984765 | ZNF175 (margin: ZNF175; SIGLEC28P; SIGLEC29P) | 14.38 | 1.55e-04 | 2.71e-03 |
|  | 6 | 26045532 | 26045987 | PDFSTATSENSEMBLUCSC | 456 | 5 | 35512 | island | 0.001452 | 0.939584 | U91328.2; HIST1H3C; HIST1H2BB (margin: U91328.2; HIST1H3C; HIST1H2AB; HIST1H2BB; HIST1H1C) | 14.32 | 7.94e-03 | 3.19e-02 |
|  | 3 | 37902229 | 37902741 | PDFSTATSENSEMBLUCSC | 513 | 5 | 28223 | tss;island | 0.004721 | 0.965672 | CTDSPL; AC093415.2 (margin: CTDSPL; AC093415.2) | 14.32 | 7.94e-03 | 3.19e-02 |
|  | 22 | 21368291 | 21368765 | PDFSTATSENSEMBLUCSC | 475 | 8 | 26989 | tss;island | 0.003739 | 0.979388 | P2RX6; TUBA3FP (margin: THAP7-AS1; P2RX6; THAP7; TUBA3FP; AC002472.1) | 14.29 | 1.55e-04 | 2.71e-03 |
|  | 1 | 77332991 | 77333159 | PDFSTATSENSEMBLUCSC | 169 | 6 | 1703 | tss | 0.977517 | 0.006373 | ST6GALNAC5 (margin: ST6GALNAC5) | 14.24 | 2.16e-03 | 1.45e-02 |
|  | 1 | 90309205 | 90309605 | PDFSTATSENSEMBLUCSC | 401 | 5 | 622 | gene;island | 0.004863 | 0.984824 | RP11-302M6.4; LRRC8D (margin: RP11-302M6.4; LRRC8D) | 14.20 | 7.94e-03 | 3.19e-02 |
|  | 15 | 41061984 | 41062273 | PDFSTATSENSEMBLUCSC | 290 | 6 | 13296 | gene | 0.001681 | 0.963081 | C15orf62; DNAJC17 (margin: RP11-532F12.6; GCHFR; C15orf62; DNAJC17) | 14.11 | 2.16e-03 | 1.45e-02 |
|  | 6 | 27106988 | 27107756 | PDFSTATSENSEMBLUCSC | 769 | 11 | 33617 | gene;island | 0.007674 | 0.971868 | HIST1H4I; HIST1H2BK (margin: HIST1H2AG; HIST1H4I; HIST1H2AH; MIR3143; HIST1H2BJ; HIST1H2BK) | 13.93 | 2.84e-06 | 1.96e-04 |
|  | 16 | 31483137 | 31483534 | PDFSTATSENSEMBLUCSC | 398 | 5 | 15212 | tss;island | 0.006349 | 0.981665 | TGFB1I1 (margin: ARMC5; TGFB1I1; SLC5A2) | 13.85 | 7.94e-03 | 3.19e-02 |
|  | 2 | 85640762 | 85641254 | PDFSTATSENSEMBLUCSC | 493 | 6 | 24478 | island | 0.001772 | 0.984150 | CAPG (margin: SH2D6; CAPG) | 13.84 | 2.16e-03 | 1.45e-02 |
|  | 12 | 89744471 | 89744877 | PDFSTATSENSEMBLUCSC | 407 | 8 | 9238 | gene;island | 0.004694 | 0.967936 | DUSP6 (margin: DUSP6) | 13.69 | 1.55e-04 | 2.71e-03 |
|  | 16 | 57836242 | 57836706 | PDFSTATSENSEMBLUCSC | 465 | 6 | 15969 | island | 0.008506 | 0.979331 | CTD-2600O9.1; KIFC3 (margin: CTD-2600O9.1; KIFC3) | 13.65 | 2.16e-03 | 1.45e-02 |
|  | 2 | 27603611 | 27604131 | PDFSTATSENSEMBLUCSC | 521 | 11 | 23267 | tss | 0.009595 | 0.975245 | ZNF513; PPM1G (margin: SNX17; EIF2B4; ZNF513; PPM1G; FTH1P3) | 13.63 | 2.84e-06 | 1.96e-04 |
|  | 10 | 17270300 | 17270601 | PDFSTATSENSEMBLUCSC | 302 | 5 | 4517 | gene | 0.007815 | 0.989157 | VIM; VIM-AS1 (margin: VIM; VIM-AS1; RP11-124N14.3) | 13.61 | 7.94e-03 | 3.19e-02 |
|  | 1 | 24307153 | 24307535 | PDFSTATSENSEMBLUCSC | 383 | 5 | 1354 | tss;island | 0.001968 | 0.971472 | SRSF10 (margin: SRSF10) | 13.60 | 7.94e-03 | 3.19e-02 |
|  | 4 | 40517938 | 40518143 | PDFSTATSENSEMBLUCSC | 206 | 5 | 29987 | gene | 0.011117 | 0.973169 | RBM47 (margin: RBM47) | 13.42 | 7.94e-03 | 3.19e-02 |
|  | 11 | 33037397 | 33037626 | PDFSTATSENSEMBLUCSC | 230 | 5 | 7230 | tss | 0.009358 | 0.989353 | DEPDC7 (margin: DEPDC7; Y\_RNA) | 13.35 | 7.94e-03 | 3.19e-02 |
|  | 2 | 29337946 | 29338258 | PDFSTATSENSEMBLUCSC | 313 | 9 | 23282 | tss | 0.005635 | 0.988847 | CLIP4 (margin: CLIP4) | 13.32 | 4.11e-05 | 1.17e-03 |
|  | 5 | 912383 | 912860 | PDFSTATSENSEMBLUCSC | 478 | 6 | 31516 | gene;island | 0.008493 | 0.984922 | TRIP13 (margin: TRIP13) | 13.29 | 2.16e-03 | 1.45e-02 |
|  | 2 | 29337946 | 29338432 | PDFSTATSENSEMBLUCSC | 487 | 10 | 24198 | island | 0.008064 | 0.989186 | CLIP4 (margin: CLIP4) | 13.26 | 1.08e-05 | 4.82e-04 |
|  | 11 | 121986961 | 121987203 | PDFSTATSENSEMBLUCSC | 243 | 7 | 6948 | gene;tss | 0.981970 | 0.012363 | RP11-166D19.1; BLID (margin: RP11-166D19.1; BLID) | 13.05 | 5.83e-04 | 6.47e-03 |
|  | 7 | 27195918 | 27196555 | PDFSTATSENSEMBLUCSC | 638 | 11 | 38353 | island | 0.005620 | 0.979773 | HOXA-AS3; RP1-170O19.21; HOXA7 (margin: HOXA-AS3; RP1-170O19.21; HOXA-AS4; HOXA3; HOXA6; HOXA7; HOXA9; RP1-170O19.20) | 13.03 | 2.84e-06 | 1.96e-04 |
|  | 7 | 27196296 | 27196555 | PDFSTATSENSEMBLUCSC | 260 | 7 | 37494 | tss | 0.005620 | 0.979246 | HOXA-AS3; RP1-170O19.21; HOXA7 (margin: HOXA-AS3; RP1-170O19.21; HOXA-AS4; HOXA3; HOXA6; HOXA7; HOXA9; RP1-170O19.20) | 13.03 | 5.83e-04 | 6.47e-03 |
|  | 18 | 44526430 | 44526756 | PDFSTATSENSEMBLUCSC | 327 | 6 | 19260 | tss | 0.960910 | 0.002831 | KATNAL2 (margin: KATNAL2) | 13.03 | 2.16e-03 | 1.45e-02 |
|  | 13 | 88323266 | 88323607 | PDFSTATSENSEMBLUCSC | 342 | 5 | 11807 | island | 0.284860 | 0.002756 | SLITRK5; MIR4500HG (margin: SLITRK5; MIR4500HG) | 12.96 | 7.94e-03 | 3.19e-02 |
|  | 16 | 56659336 | 56659724 | PDFSTATSENSEMBLUCSC | 389 | 7 | 15949 | island | 0.007345 | 0.970085 | MT1E (margin: MT1L; MT1E; MT1M; MT1JP; AC026461.1) | 12.93 | 5.83e-04 | 6.47e-03 |
|  | 12 | 21809973 | 21810762 | PDFSTATSENSEMBLUCSC | 790 | 12 | 9080 | gene | 0.007273 | 0.976024 | LDHB (margin: RP11-59N23.3; LDHB) | 12.92 | 7.40e-07 | 7.21e-05 |
|  | 21 | 26934197 | 26934682 | PDFSTATSENSEMBLUCSC | 486 | 6 | 26615 | island | 0.005765 | 0.975697 | MIR155HG (margin: MIR155HG) | 12.87 | 2.16e-03 | 1.45e-02 |
|  | 7 | 27154262 | 27155039 | PDFSTATSENSEMBLUCSC | 778 | 14 | 37490 | tss | 0.003101 | 0.969552 | HOXA-AS2; HOXA3 (margin: HOXA-AS2; HOXA2; HOXA3) | 12.86 | 4.99e-08 | 9.34e-06 |
|  | 1 | 77332991 | 77333229 | PDFSTATSENSEMBLUCSC | 239 | 8 | 3393 | island | 0.980715 | 0.008635 | ST6GALNAC5 (margin: ST6GALNAC5) | 12.85 | 1.55e-04 | 2.71e-03 |
|  | 11 | 33037397 | 33037916 | PDFSTATSENSEMBLUCSC | 520 | 7 | 8185 | island | 0.011832 | 0.978505 | DEPDC7 (margin: DEPDC7; Y\_RNA) | 12.80 | 5.83e-04 | 6.47e-03 |
|  | 4 | 176987009 | 176987577 | PDFSTATSENSEMBLUCSC | 569 | 7 | 30186 | gene | 0.740243 | 0.004721 | WDR17 (margin: WDR17) | 12.79 | 5.83e-04 | 6.47e-03 |
|  | 12 | 51566731 | 51567112 | PDFSTATSENSEMBLUCSC | 382 | 9 | 9712 | tss | 0.011744 | 0.977694 | TFCP2 (margin: RNU6-199P; TFCP2) | 12.65 | 4.11e-05 | 1.17e-03 |
|  | 12 | 30948636 | 30948794 | PDFSTATSENSEMBLUCSC | 159 | 5 | 10308 | island | 0.007858 | 0.980801 | LINC00941 (margin: LINC00941) | 12.65 | 7.94e-03 | 3.19e-02 |
|  | 7 | 92672812 | 92673176 | PDFSTATSENSEMBLUCSC | 365 | 5 | 38665 | island | 0.008107 | 0.960080 | (margin: ) | 12.62 | 7.94e-03 | 3.19e-02 |
|  | 4 | 165877875 | 165878317 | PDFSTATSENSEMBLUCSC | 443 | 8 | 30171 | gene;island | 0.982038 | 0.020190 | RP11-366M4.8; FAM218A; TRIM61 (margin: RP11-366M4.8; FAM218A; TRIM61; RP11-366M4.11) | 12.61 | 1.55e-04 | 2.71e-03 |
|  | 11 | 33037530 | 33037916 | PDFSTATSENSEMBLUCSC | 387 | 6 | 6572 | gene | 0.013750 | 0.983929 | DEPDC7 (margin: DEPDC7; Y\_RNA) | 12.52 | 2.16e-03 | 1.45e-02 |
|  | 14 | 75593812 | 75594058 | PDFSTATSENSEMBLUCSC | 247 | 9 | 12516 | tss | 0.006636 | 0.982689 | RP11-950C14.7; NEK9 (margin: RP11-950C14.7; NEK9; HIF1AP1; TMED10) | 12.52 | 4.11e-05 | 1.17e-03 |
|  | 6 | 138893673 | 138893718 | PDFSTATSENSEMBLUCSC | 46 | 5 | 35253 | tss | 0.985415 | 0.014239 | NHSL1 (margin: NHSL1) | 12.51 | 7.94e-03 | 3.19e-02 |
|  | 14 | 75593334 | 75594058 | PDFSTATSENSEMBLUCSC | 725 | 14 | 13013 | island | 0.006478 | 0.983746 | RP11-950C14.7; NEK9 (margin: RP11-950C14.7; NEK9; HIF1AP1; TMED10) | 12.50 | 4.99e-08 | 9.34e-06 |
|  | 14 | 75593334 | 75593738 | PDFSTATSENSEMBLUCSC | 405 | 5 | 12144 | gene | 0.005556 | 0.984694 | RP11-950C14.7; NEK9 (margin: RP11-950C14.7; NEK9; HIF1AP1; TMED10) | 12.48 | 7.94e-03 | 3.19e-02 |
|  | 18 | 44526430 | 44527026 | PDFSTATSENSEMBLUCSC | 597 | 8 | 19493 | island | 0.960910 | 0.003157 | KATNAL2 (margin: KATNAL2) | 12.45 | 1.55e-04 | 2.71e-03 |
|  | 6 | 125284203 | 125284359 | PDFSTATSENSEMBLUCSC | 157 | 6 | 35208 | tss;island | 0.010135 | 0.985424 | RNF217; RP11-510H23.1 (margin: RNF217; RP11-510H23.1) | 12.43 | 2.16e-03 | 1.45e-02 |
|  | 20 | 57581903 | 57582292 | PDFSTATSENSEMBLUCSC | 390 | 6 | 25478 | gene | 0.008945 | 0.982509 | CTSZ (margin: NELFCD; CTSZ) | 12.41 | 2.16e-03 | 1.45e-02 |
|  | 19 | 9649241 | 9649542 | PDFSTATSENSEMBLUCSC | 302 | 9 | 21500 | island | 0.004427 | 0.965967 | ZNF426 (margin: ZNF426) | 12.39 | 4.11e-05 | 1.17e-03 |
|  | 19 | 9649310 | 9649542 | PDFSTATSENSEMBLUCSC | 233 | 7 | 20426 | tss | 0.008980 | 0.965967 | ZNF426 (margin: ZNF426) | 12.38 | 5.83e-04 | 6.47e-03 |
|  | 14 | 69256677 | 69256977 | PDFSTATSENSEMBLUCSC | 301 | 5 | 12121 | gene;island | 0.010901 | 0.978651 | ZFP36L1 (margin: ZFP36L1) | 12.31 | 7.94e-03 | 3.19e-02 |
|  | 6 | 30652202 | 30652399 | PDFSTATSENSEMBLUCSC | 198 | 9 | 33802 | gene;island | 0.019670 | 0.982218 | PPP1R18 (margin: DHX16; PPP1R18; NRM) | 12.26 | 4.11e-05 | 1.17e-03 |
|  | 1 | 25566331 | 25566753 | PDFSTATSENSEMBLUCSC | 423 | 5 | 2868 | island | 0.002736 | 0.954348 | C1orf63 (margin: RP3-465N24.6; SYF2; C1orf63) | 12.14 | 7.94e-03 | 3.19e-02 |
|  | 1 | 207224090 | 207224740 | PDFSTATSENSEMBLUCSC | 651 | 9 | 4146 | island | 0.004897 | 0.954783 | PFKFB2; YOD1 (margin: PFKFB2; snoU13; YOD1) | 12.12 | 4.11e-05 | 1.17e-03 |
|  | 2 | 27603547 | 27604131 | PDFSTATSENSEMBLUCSC | 585 | 12 | 24181 | island | 0.013656 | 0.973814 | ZNF513; PPM1G (margin: SNX17; EIF2B4; ZNF513; PPM1G; FTH1P3) | 12.04 | 7.40e-07 | 7.21e-05 |
|  | 7 | 92466078 | 92466386 | PDFSTATSENSEMBLUCSC | 309 | 6 | 37711 | tss;island | 0.007206 | 0.964979 | AC002454.1; CDK6 (margin: AC002454.1; CDK6) | 11.98 | 2.16e-03 | 1.45e-02 |
|  | 17 | 79881468 | 79881543 | PDFSTATSENSEMBLUCSC | 76 | 5 | 16929 | gene;tss;island | 0.009827 | 0.987489 | RP11-498C9.12; MAFG (margin: RP11-498C9.12; MAFG-AS1; RP11-498C9.13; SIRT7; MAFG; PYCR1) | 11.97 | 7.94e-03 | 3.19e-02 |
|  | 2 | 79220029 | 79220528 | PDFSTATSENSEMBLUCSC | 500 | 5 | 24460 | island | 0.936069 | 0.011013 | (margin: ) | 11.95 | 7.94e-03 | 3.19e-02 |
|  | 8 | 145025059 | 145025178 | PDFSTATSENSEMBLUCSC | 120 | 5 | 39966 | tss | 0.007933 | 0.967393 | PLEC (margin: PLEC; MIR661) | 11.87 | 7.94e-03 | 3.19e-02 |
|  | 22 | 45608428 | 45608713 | PDFSTATSENSEMBLUCSC | 286 | 9 | 27219 | tss | 0.011787 | 0.979508 | KIAA0930 (margin: KIAA0930; MIR1249) | 11.85 | 4.11e-05 | 1.17e-03 |
|  | 11 | 6341842 | 6341908 | PDFSTATSENSEMBLUCSC | 67 | 7 | 7106 | tss | 0.016418 | 0.981032 | PRKCDBP (margin: PRKCDBP) | 11.81 | 5.83e-04 | 6.47e-03 |
|  | 20 | 57581903 | 57582371 | PDFSTATSENSEMBLUCSC | 469 | 7 | 26299 | island | 0.010674 | 0.982445 | CTSZ (margin: NELFCD; TUBB1; CTSZ) | 11.80 | 5.83e-04 | 6.47e-03 |
|  | 5 | 11904015 | 11904127 | PDFSTATSENSEMBLUCSC | 113 | 6 | 32695 | island | 0.949890 | 0.016061 | CTNND2 (margin: CTNND2) | 11.78 | 2.16e-03 | 1.45e-02 |
|  | 3 | 48507354 | 48507618 | PDFSTATSENSEMBLUCSC | 265 | 6 | 27767 | gene | 0.000825 | 0.796395 | ATRIP; TREX1; SHISA5 (margin: ATRIP; TREX1; SHISA5) | 11.77 | 2.16e-03 | 1.45e-02 |
|  | 5 | 137225191 | 137225509 | PDFSTATSENSEMBLUCSC | 319 | 5 | 31763 | gene;island | 0.002456 | 0.960502 | MYOT; PKD2L2; RP11-381K20.2 (margin: MYOT; PKD2L2; RP11-381K20.2) | 11.76 | 7.94e-03 | 3.19e-02 |
|  | 3 | 50378407 | 50378664 | PDFSTATSENSEMBLUCSC | 258 | 9 | 28353 | tss | 0.010074 | 0.974616 | ZMYND10-AS1; RASSF1; ZMYND10 (margin: ZMYND10-AS1; CYB561D2; XXcos-LUCA11.5; RASSF1; ZMYND10; NPRL2) | 11.73 | 4.11e-05 | 1.17e-03 |
|  | 7 | 27154262 | 27155548 | PDFSTATSENSEMBLUCSC | 1287 | 18 | 36931 | gene;island | 0.005790 | 0.969552 | HOXA-AS2; HOXA3 (margin: HOXA-AS2; HOXA2; HOXA3) | 11.71 | 2.20e-10 | 1.16e-07 |
|  | 15 | 79382548 | 79383167 | PDFSTATSENSEMBLUCSC | 620 | 7 | 13447 | gene;island | 0.944524 | 0.009283 | RASGRF1 (margin: RASGRF1) | 11.70 | 5.83e-04 | 6.47e-03 |
|  | 7 | 24796981 | 24797486 | PDFSTATSENSEMBLUCSC | 506 | 5 | 36922 | gene | 0.008012 | 0.951322 | DFNA5 (margin: DFNA5) | 11.64 | 7.94e-03 | 3.19e-02 |
|  | 2 | 176994142 | 176994448 | PDFSTATSENSEMBLUCSC | 307 | 5 | 23739 | tss | 0.012218 | 0.970804 | HOXD8; HOXD-AS2 (margin: HOXD10; HOXD9; HOXD8; HOXD3; HOXD-AS2) | 11.62 | 7.94e-03 | 3.19e-02 |
|  | 12 | 51566379 | 51567112 | PDFSTATSENSEMBLUCSC | 734 | 11 | 10422 | island | 0.011744 | 0.973162 | TFCP2 (margin: RNU6-199P; TFCP2) | 11.62 | 2.84e-06 | 1.96e-04 |
|  | 1 | 150254280 | 150254546 | PDFSTATSENSEMBLUCSC | 267 | 5 | 1905 | tss;island | 0.879954 | 0.005045 | C1orf54; C1orf51 (margin: C1orf54; C1orf51; MRPS21) | 11.61 | 7.94e-03 | 3.19e-02 |
|  | 6 | 28979210 | 28979498 | PDFSTATSENSEMBLUCSC | 289 | 12 | 35608 | island | 0.018409 | 0.977578 | (margin: ZNF311) | 11.61 | 7.40e-07 | 7.21e-05 |
|  | 19 | 37997675 | 37997831 | PDFSTATSENSEMBLUCSC | 157 | 8 | 20740 | tss | 0.899233 | 0.002090 | ZNF793; CTD-3064H18.1 (margin: ZNF793; CTD-3064H18.1; CTD-3064H18.2) | 11.60 | 1.55e-04 | 2.71e-03 |
|  | 11 | 796361 | 796607 | PDFSTATSENSEMBLUCSC | 247 | 5 | 7028 | tss;island | 0.007354 | 0.958939 | SLC25A22 (margin: CEND1; SLC25A22; PIDD) | 11.58 | 7.94e-03 | 3.19e-02 |
|  | 11 | 6341717 | 6341908 | PDFSTATSENSEMBLUCSC | 192 | 8 | 8013 | island | 0.018490 | 0.980499 | PRKCDBP (margin: PRKCDBP) | 11.58 | 1.55e-04 | 2.71e-03 |
|  | 2 | 178257176 | 178257923 | PDFSTATSENSEMBLUCSC | 748 | 14 | 22943 | gene;tss;island | 0.011288 | 0.953897 | AGPS; NFE2L2; AC074286.1 (margin: AGPS; NFE2L2; AC074286.1) | 11.57 | 4.99e-08 | 9.34e-06 |
|  | 7 | 25219543 | 25220162 | PDFSTATSENSEMBLUCSC | 620 | 12 | 38322 | island | 0.011678 | 0.965690 | C7orf31 (margin: AC004129.7; C7orf31) | 11.56 | 7.40e-07 | 7.21e-05 |
|  | 22 | 50968250 | 50968343 | PDFSTATSENSEMBLUCSC | 94 | 5 | 26931 | gene | 0.012995 | 0.976905 | TYMP; ODF3B (margin: NCAPH2; SCO2; TYMP; ODF3B) | 11.54 | 7.94e-03 | 3.19e-02 |
|  | 22 | 50968297 | 50968516 | PDFSTATSENSEMBLUCSC | 220 | 5 | 27258 | tss | 0.012080 | 0.976905 | TYMP; ODF3B (margin: NCAPH2; SCO2; TYMP; ODF3B) | 11.54 | 7.94e-03 | 3.19e-02 |
|  | 6 | 105584551 | 105584780 | PDFSTATSENSEMBLUCSC | 230 | 5 | 35145 | tss;island | 0.013436 | 0.967087 | BVES-AS1; BVES (margin: BVES-AS1; BVES) | 11.54 | 7.94e-03 | 3.19e-02 |
|  | 3 | 11178593 | 11178758 | PDFSTATSENSEMBLUCSC | 166 | 5 | 28154 | tss | 0.004118 | 0.882237 | HRH1 (margin: HRH1) | 11.53 | 7.94e-03 | 3.19e-02 |
|  | 22 | 45608345 | 45608713 | PDFSTATSENSEMBLUCSC | 369 | 10 | 26901 | gene | 0.010202 | 0.977688 | KIAA0930 (margin: KIAA0930; MIR1249) | 11.45 | 1.08e-05 | 4.82e-04 |
|  | 3 | 196065289 | 196065688 | PDFSTATSENSEMBLUCSC | 400 | 10 | 28812 | tss | 0.008387 | 0.963775 | TM4SF19 (margin: TM4SF19; RNU6-910P; UBXN7) | 11.45 | 1.08e-05 | 4.82e-04 |
|  | 22 | 50968250 | 50968516 | PDFSTATSENSEMBLUCSC | 267 | 6 | 27629 | island | 0.012538 | 0.975918 | TYMP; ODF3B (margin: NCAPH2; SCO2; TYMP; ODF3B) | 11.45 | 2.16e-03 | 1.45e-02 |
|  | 14 | 23834860 | 23834995 | PDFSTATSENSEMBLUCSC | 136 | 6 | 12302 | tss | 0.012711 | 0.979647 | EFS (margin: RP11-124D2.3; IL25; CMTM5; EFS) | 11.43 | 2.16e-03 | 1.45e-02 |
|  | 2 | 202122309 | 202122669 | PDFSTATSENSEMBLUCSC | 361 | 6 | 23817 | tss | 0.004098 | 0.903978 | CASP8 (margin: CASP8) | 11.43 | 2.16e-03 | 1.45e-02 |
|  | 4 | 44728642 | 44728857 | PDFSTATSENSEMBLUCSC | 216 | 7 | 30370 | tss | 0.002233 | 0.560186 | GNPDA2 (margin: GNPDA2) | 11.39 | 5.83e-04 | 6.47e-03 |
|  | 2 | 220117599 | 220117945 | PDFSTATSENSEMBLUCSC | 347 | 7 | 23029 | gene;tss;island | 0.010294 | 0.976942 | TUBA4B; TUBA4A (margin: STK16; TUBA4B; GLB1L; TUBA4A) | 11.37 | 5.83e-04 | 6.47e-03 |
|  | 6 | 28367279 | 28367898 | PDFSTATSENSEMBLUCSC | 620 | 9 | 35588 | island | 0.010310 | 0.940185 | ZSCAN12 (margin: RNU2-45P; ZSCAN12) | 11.34 | 4.11e-05 | 1.17e-03 |
|  | 1 | 21616619 | 21617100 | PDFSTATSENSEMBLUCSC | 482 | 5 | 295 | gene;island | 0.012588 | 0.976768 | ECE1 (margin: RP5-1071N3.1; ECE1) | 11.34 | 7.94e-03 | 3.19e-02 |
|  | 21 | 34398085 | 34398265 | PDFSTATSENSEMBLUCSC | 181 | 8 | 26642 | island | 0.938258 | 0.005250 | OLIG2; AP000282.2 (margin: OLIG2; AP000282.2) | 11.33 | 1.55e-04 | 2.71e-03 |
|  | 7 | 48129797 | 48129992 | PDFSTATSENSEMBLUCSC | 196 | 5 | 37006 | gene;island | 0.005170 | 0.925872 | UPP1 (margin: UPP1) | 11.32 | 7.94e-03 | 3.19e-02 |
|  | 4 | 165877875 | 165878091 | PDFSTATSENSEMBLUCSC | 217 | 5 | 30665 | tss | 0.976683 | 0.033206 | RP11-366M4.8; FAM218A; TRIM61 (margin: RP11-366M4.8; FAM218A; TRIM61; RP11-366M4.11) | 11.31 | 7.94e-03 | 3.19e-02 |
|  | 7 | 27209131 | 27209582 | PDFSTATSENSEMBLUCSC | 452 | 10 | 38358 | island | 0.014704 | 0.977453 | HOXA-AS4; HOXA9; RP1-170O19.20; MIR196B; HOXA10 (margin: RP1-170O19.21; HOXA-AS4; HOXA7; HOXA9; RP1-170O19.20; MIR196B; HOXA10; HOXA11) | 11.30 | 1.08e-05 | 4.82e-04 |
|  | 6 | 106546704 | 106546824 | PDFSTATSENSEMBLUCSC | 121 | 5 | 34434 | gene | 0.004456 | 0.965134 | PRDM1; RP1-134E15.3 (margin: PRDM1; RP1-134E15.3) | 11.29 | 7.94e-03 | 3.19e-02 |
|  | 5 | 140207460 | 140207636 | PDFSTATSENSEMBLUCSC | 177 | 5 | 32352 | tss | 0.957192 | 0.013723 | PCDHA1; PCDHA2; PCDHA3; PCDHA4; PCDHA5; PCDHA6 (margin: PCDHA1; PCDHA2; PCDHA3; PCDHA4; PCDHA5; PCDHA6; PCDHA7) | 11.29 | 7.94e-03 | 3.19e-02 |
|  | 21 | 42797847 | 42798386 | PDFSTATSENSEMBLUCSC | 540 | 6 | 26430 | gene;island | 0.018467 | 0.973560 | MX1 (margin: MX1) | 11.28 | 2.16e-03 | 1.45e-02 |
|  | 19 | 37997675 | 37998171 | PDFSTATSENSEMBLUCSC | 497 | 11 | 21957 | island | 0.884806 | 0.002573 | ZNF793; CTD-3064H18.1 (margin: ZNF793; CTD-3064H18.1; CTD-3064H18.2) | 11.28 | 2.84e-06 | 1.96e-04 |
|  | 1 | 209825672 | 209825943 | PDFSTATSENSEMBLUCSC | 272 | 7 | 2230 | tss | 0.004107 | 0.896473 | LAMB3 (margin: LAMB3; RP1-28O10.1) | 11.26 | 5.83e-04 | 6.47e-03 |
|  | 1 | 89664260 | 89664582 | PDFSTATSENSEMBLUCSC | 323 | 6 | 619 | gene | 0.018831 | 0.978538 | GBP4 (margin: GBP4) | 11.26 | 2.16e-03 | 1.45e-02 |
|  | 7 | 44152996 | 44153500 | PDFSTATSENSEMBLUCSC | 505 | 6 | 36990 | gene;island | 0.978531 | 0.016479 | AEBP1; POLD2 (margin: AEBP1; MIR4649; POLD2; RNA5SP230) | 11.24 | 2.16e-03 | 1.45e-02 |
|  | 4 | 163085235 | 163085411 | PDFSTATSENSEMBLUCSC | 177 | 8 | 30658 | tss | 0.954803 | 0.006920 | FSTL5 (margin: FSTL5) | 11.24 | 1.55e-04 | 2.71e-03 |
|  | 3 | 192445388 | 192445594 | PDFSTATSENSEMBLUCSC | 207 | 7 | 28792 | tss | 0.916534 | 0.006587 | FGF12; RNU1-20P (margin: FGF12; RNU1-20P) | 11.22 | 5.83e-04 | 6.47e-03 |
|  | 3 | 50378191 | 50378664 | PDFSTATSENSEMBLUCSC | 474 | 11 | 29153 | island | 0.015913 | 0.974616 | ZMYND10-AS1; RASSF1; ZMYND10 (margin: ZMYND10-AS1; CYB561D2; XXcos-LUCA11.5; RASSF1; ZMYND10; NPRL2) | 11.21 | 2.84e-06 | 1.96e-04 |
|  | 18 | 28622474 | 28623159 | PDFSTATSENSEMBLUCSC | 686 | 13 | 19446 | island | 0.013858 | 0.969944 | DSC3 (margin: DSC3) | 11.21 | 1.92e-07 | 2.60e-05 |
|  | 6 | 27840957 | 27841423 | PDFSTATSENSEMBLUCSC | 467 | 11 | 34728 | tss;island | 0.036927 | 0.984328 | HIST1H3I; HIST1H4L (margin: HIST1H2AL; HIST1H2BPS2; HIST1H1B; HIST1H3I; HIST1H4L) | 11.21 | 2.84e-06 | 1.96e-04 |
|  | 6 | 27840957 | 27841230 | PDFSTATSENSEMBLUCSC | 274 | 5 | 33624 | gene | 0.047288 | 0.978076 | HIST1H3I; HIST1H4L (margin: HIST1H2AL; HIST1H2BPS2; HIST1H1B; HIST1H3I; HIST1H4L) | 11.21 | 7.94e-03 | 3.19e-02 |
|  | 15 | 84047906 | 84048308 | PDFSTATSENSEMBLUCSC | 403 | 5 | 14399 | island | 0.007696 | 0.965035 | RP11-382A20.4 (margin: RP11-382A20.4) | 11.19 | 7.94e-03 | 3.19e-02 |
|  | 18 | 28622811 | 28623159 | PDFSTATSENSEMBLUCSC | 349 | 10 | 19220 | tss | 0.015294 | 0.970967 | DSC3 (margin: DSC3) | 11.18 | 1.08e-05 | 4.82e-04 |
|  | 7 | 27209195 | 27209582 | PDFSTATSENSEMBLUCSC | 388 | 9 | 37497 | tss | 0.013288 | 0.977706 | HOXA-AS4; HOXA9; RP1-170O19.20; MIR196B; HOXA10 (margin: RP1-170O19.21; HOXA-AS4; HOXA7; HOXA9; RP1-170O19.20; MIR196B; HOXA10; HOXA11) | 11.18 | 4.11e-05 | 1.17e-03 |
|  | 2 | 71503484 | 71503883 | PDFSTATSENSEMBLUCSC | 400 | 7 | 24396 | island | 0.788977 | 0.005315 | ZNF638 (margin: ZNF638) | 11.14 | 5.83e-04 | 6.47e-03 |
|  | 19 | 571107 | 571416 | PDFSTATSENSEMBLUCSC | 310 | 5 | 20274 | tss;island | 0.908118 | 0.009494 | BSG; AC009005.2 (margin: BSG; AC009005.2) | 11.11 | 7.94e-03 | 3.19e-02 |
|  | 6 | 28367544 | 28367898 | PDFSTATSENSEMBLUCSC | 355 | 6 | 34741 | tss | 0.013260 | 0.946440 | ZSCAN12 (margin: RNU2-45P; ZSCAN12) | 11.11 | 2.16e-03 | 1.45e-02 |
|  | 17 | 49008322 | 49008925 | PDFSTATSENSEMBLUCSC | 604 | 7 | 18563 | island | 0.018188 | 0.964140 | (margin: RP11-700H6.1) | 11.09 | 5.83e-04 | 6.47e-03 |
|  | 12 | 49393166 | 49393408 | PDFSTATSENSEMBLUCSC | 243 | 7 | 9680 | tss | 0.021603 | 0.984336 | RP11-386G11.5; DDN (margin: RP11-386G11.3; RP11-386G11.5; DDN; PRKAG1) | 11.09 | 5.83e-04 | 6.47e-03 |
|  | 12 | 49393027 | 49393408 | PDFSTATSENSEMBLUCSC | 382 | 8 | 10381 | island | 0.021250 | 0.908442 | RP11-386G11.5; DDN (margin: RP11-386G11.3; RP11-386G11.5; DDN; PRKAG1) | 11.08 | 1.55e-04 | 2.71e-03 |
|  | 17 | 19648846 | 19649293 | PDFSTATSENSEMBLUCSC | 448 | 10 | 17194 | tss | 0.012939 | 0.939134 | ALDH3A1 (margin: RP11-311F12.2; AC005722.4; ALDH3A1) | 11.05 | 1.08e-05 | 4.82e-04 |
|  | 11 | 63655748 | 63656090 | PDFSTATSENSEMBLUCSC | 343 | 5 | 6655 | gene | 0.009720 | 0.927391 | MARK2 (margin: MARK2; RNU6-1306P) | 11.04 | 7.94e-03 | 3.19e-02 |
|  | 10 | 118031864 | 118032081 | PDFSTATSENSEMBLUCSC | 218 | 5 | 4773 | gene;tss;island | 0.817448 | 0.011891 | GFRA1 (margin: GFRA1) | 11.03 | 7.94e-03 | 3.19e-02 |
|  | 5 | 140207460 | 140208201 | PDFSTATSENSEMBLUCSC | 742 | 9 | 31797 | gene;island | 0.937397 | 0.013723 | PCDHA1; PCDHA2; PCDHA3; PCDHA4; PCDHA5; PCDHA6 (margin: PCDHA1; PCDHA2; PCDHA3; PCDHA4; PCDHA5; PCDHA6; PCDHA7) | 11.02 | 4.11e-05 | 1.17e-03 |
|  | 8 | 67454546 | 67454892 | PDFSTATSENSEMBLUCSC | 347 | 5 | 40356 | island | 0.963815 | 0.013273 | (margin: ) | 10.99 | 7.94e-03 | 3.19e-02 |
|  | 7 | 87257356 | 87257786 | PDFSTATSENSEMBLUCSC | 431 | 7 | 37073 | gene;island | 0.668913 | 0.003613 | RUNDC3B; ABCB1 (margin: snoU13; RUNDC3B; ABCB1) | 10.99 | 5.83e-04 | 6.47e-03 |
|  | 7 | 87257356 | 87257673 | PDFSTATSENSEMBLUCSC | 318 | 5 | 37687 | tss | 0.858652 | 0.009259 | RUNDC3B; ABCB1 (margin: snoU13; RUNDC3B; ABCB1) | 10.99 | 7.94e-03 | 3.19e-02 |
|  | 19 | 57702917 | 57703364 | PDFSTATSENSEMBLUCSC | 448 | 6 | 20244 | gene | 0.007256 | 0.951395 | ZNF264 (margin: ZNF264) | 10.99 | 2.16e-03 | 1.45e-02 |
|  | 6 | 152011103 | 152011415 | PDFSTATSENSEMBLUCSC | 313 | 5 | 35302 | tss | 0.900167 | 0.009987 | ESR1 (margin: ESR1) | 10.93 | 7.94e-03 | 3.19e-02 |
|  | 1 | 95006322 | 95006977 | PDFSTATSENSEMBLUCSC | 656 | 6 | 643 | gene;island | 0.002054 | 0.775091 | F3 (margin: F3) | 10.91 | 8.66e-03 | 3.36e-02 |
|  | 13 | 96296708 | 96297338 | PDFSTATSENSEMBLUCSC | 631 | 12 | 11827 | island | 0.021332 | 0.984614 | DZIP1 (margin: DZIP1; DNAJC3-AS1) | 10.89 | 1.48e-06 | 1.37e-04 |
|  | 13 | 96296979 | 96297338 | PDFSTATSENSEMBLUCSC | 360 | 10 | 11503 | tss | 0.033612 | 0.984614 | DZIP1 (margin: DZIP1; DNAJC3-AS1) | 10.89 | 2.17e-05 | 8.94e-04 |
|  | 19 | 1132256 | 1132537 | PDFSTATSENSEMBLUCSC | 282 | 8 | 19644 | gene | 0.019989 | 0.985408 | SBNO2 (margin: SBNO2) | 10.88 | 1.55e-04 | 2.71e-03 |
|  | 3 | 49395574 | 49395991 | PDFSTATSENSEMBLUCSC | 418 | 8 | 29116 | island | 0.012079 | 0.971158 | GPX1; RHOA (margin: GPX1; RHOA; RHOA-IT1) | 10.87 | 1.55e-04 | 2.71e-03 |
|  | 17 | 4981403 | 4981610 | PDFSTATSENSEMBLUCSC | 208 | 5 | 17040 | tss;island | 0.908852 | 0.006288 | ZFP3; RP11-46I8.3 (margin: ZFP3; RP11-46I8.3) | 10.86 | 7.94e-03 | 3.19e-02 |
|  | 4 | 158141449 | 158141570 | PDFSTATSENSEMBLUCSC | 122 | 5 | 30654 | tss;island | 0.979948 | 0.029928 | GRIA2 (margin: GRIA2) | 10.84 | 7.94e-03 | 3.19e-02 |
|  | 5 | 114937535 | 114938459 | PDFSTATSENSEMBLUCSC | 925 | 11 | 31721 | gene;island | 0.018747 | 0.982651 | AC010226.4; TICAM2; TMED7-TICAM2 (margin: AC010226.4; TICAM2; TMED7-TICAM2; TMED7) | 10.83 | 2.84e-06 | 1.96e-04 |
|  | 7 | 25219840 | 25220162 | PDFSTATSENSEMBLUCSC | 323 | 9 | 37480 | tss | 0.011957 | 0.960595 | C7orf31 (margin: AC004129.7; C7orf31) | 10.82 | 4.11e-05 | 1.17e-03 |
|  | 3 | 48632625 | 48633144 | PDFSTATSENSEMBLUCSC | 520 | 8 | 28301 | tss | 0.011260 | 0.962315 | COL7A1 (margin: COL7A1; UQCRC1) | 10.81 | 1.55e-04 | 2.71e-03 |
|  | 14 | 23834710 | 23834995 | PDFSTATSENSEMBLUCSC | 286 | 8 | 12718 | island | 0.012988 | 0.974690 | EFS (margin: RP11-124D2.3; IL25; CMTM5; EFS) | 10.81 | 6.22e-04 | 6.71e-03 |
|  | 8 | 98290148 | 98290601 | PDFSTATSENSEMBLUCSC | 454 | 9 | 40476 | island | 0.964551 | 0.023812 | TSPYL5 (margin: TSPYL5) | 10.80 | 4.11e-05 | 1.17e-03 |
|  | 3 | 11610138 | 11610338 | PDFSTATSENSEMBLUCSC | 201 | 5 | 27668 | gene;island | 0.009809 | 0.975604 | VGLL4 (margin: ATG7; VGLL4) | 10.77 | 7.94e-03 | 3.19e-02 |
|  | 13 | 67804285 | 67804744 | PDFSTATSENSEMBLUCSC | 460 | 9 | 11769 | island | 0.780391 | 0.004872 | PCDH9 (margin: PCDH9) | 10.76 | 4.11e-05 | 1.17e-03 |
|  | 13 | 67804509 | 67804744 | PDFSTATSENSEMBLUCSC | 236 | 7 | 11471 | tss | 0.794527 | 0.004872 | PCDH9 (margin: PCDH9) | 10.76 | 5.83e-04 | 6.47e-03 |
|  | 15 | 83952722 | 83953068 | PDFSTATSENSEMBLUCSC | 347 | 5 | 13459 | gene;island | 0.025098 | 0.979800 | RP11-382A20.4; BNC1 (margin: RP11-382A20.4; BNC1) | 10.76 | 7.94e-03 | 3.19e-02 |
|  | 1 | 25256939 | 25257191 | PDFSTATSENSEMBLUCSC | 253 | 6 | 330 | gene;tss;island | 0.030781 | 0.937064 | RUNX3 (margin: RUNX3) | 10.74 | 2.16e-03 | 1.45e-02 |
|  | 2 | 128158438 | 128159058 | PDFSTATSENSEMBLUCSC | 621 | 6 | 24688 | island | 0.022968 | 0.943948 | AC068282.3 (margin: AC068282.3) | 10.74 | 2.16e-03 | 1.45e-02 |
|  | 17 | 19648718 | 19649293 | PDFSTATSENSEMBLUCSC | 576 | 11 | 16484 | gene;island | 0.013915 | 0.950760 | ALDH3A1 (margin: RP11-311F12.2; AC005722.4; ALDH3A1) | 10.72 | 2.84e-06 | 1.96e-04 |
|  | 21 | 34398085 | 34398226 | PDFSTATSENSEMBLUCSC | 142 | 6 | 26516 | tss | 0.791043 | 0.005250 | OLIG2; AP000282.2 (margin: OLIG2; AP000282.2) | 10.68 | 2.16e-03 | 1.45e-02 |
|  | 19 | 50979561 | 50979755 | PDFSTATSENSEMBLUCSC | 195 | 6 | 20177 | gene;island | 0.015864 | 0.965028 | EMC10; FAM71E1 (margin: MYBPC2; EMC10; FAM71E1; CTD-2545M3.2; CTD-2545M3.8) | 10.65 | 2.16e-03 | 1.45e-02 |
|  | 3 | 149374761 | 149375293 | PDFSTATSENSEMBLUCSC | 533 | 7 | 27992 | gene;island | 0.007860 | 0.973288 | WWTR1-AS1; WWTR1 (margin: WWTR1-AS1; WWTR1; WWTR1-IT1) | 10.63 | 5.83e-04 | 6.47e-03 |
|  | 7 | 92237896 | 92238364 | PDFSTATSENSEMBLUCSC | 469 | 6 | 37086 | gene | 0.975002 | 0.019117 | CDK6 (margin: CDK6) | 10.63 | 2.16e-03 | 1.45e-02 |
|  | 19 | 17413767 | 17414399 | PDFSTATSENSEMBLUCSC | 633 | 9 | 21734 | island | 0.014257 | 0.975877 | MRPL34; ABHD8 (margin: MRPL34; DDA1; ABHD8; CTD-2278I10.4) | 10.61 | 4.11e-05 | 1.17e-03 |
|  | 3 | 158390329 | 158390821 | PDFSTATSENSEMBLUCSC | 493 | 10 | 28012 | gene | 0.957981 | 0.016181 | GFM1; LXN (margin: GFM1; LXN) | 10.61 | 1.08e-05 | 4.82e-04 |
|  | 16 | 4588826 | 4589127 | PDFSTATSENSEMBLUCSC | 302 | 7 | 15071 | tss | 0.008563 | 0.977908 | CDIP1 (margin: CDIP1) | 10.61 | 4.08e-03 | 2.47e-02 |
|  | 19 | 17414284 | 17414399 | PDFSTATSENSEMBLUCSC | 116 | 6 | 20585 | tss | 0.012743 | 0.949612 | MRPL34; ABHD8 (margin: MRPL34; DDA1; ABHD8; CTD-2278I10.4) | 10.61 | 2.16e-03 | 1.45e-02 |
|  | 1 | 32041507 | 32042037 | PDFSTATSENSEMBLUCSC | 531 | 7 | 1448 | tss | 0.009848 | 0.948990 | TINAGL1; RP11-73M7.1 (margin: TINAGL1; RP11-73M7.1) | 10.59 | 5.83e-04 | 6.47e-03 |
|  | 13 | 20392406 | 20392706 | PDFSTATSENSEMBLUCSC | 301 | 5 | 11562 | island | 0.978530 | 0.029956 | ST6GALNAC4P1 (margin: ST6GALNAC4P1; ZMYM5) | 10.58 | 7.94e-03 | 3.19e-02 |
|  | 19 | 40314862 | 40315143 | PDFSTATSENSEMBLUCSC | 282 | 5 | 22005 | island | 0.982789 | 0.050287 | DYRK1B (margin: DYRK1B; FBL) | 10.58 | 7.94e-03 | 3.19e-02 |
|  | 20 | 13976093 | 13976190 | PDFSTATSENSEMBLUCSC | 98 | 7 | 26012 | island | 0.855699 | 0.004673 | MACROD2; SEL1L2 (margin: MACROD2; RPS3P1; SEL1L2) | 10.56 | 5.83e-04 | 6.47e-03 |
|  | 19 | 50832552 | 50832861 | PDFSTATSENSEMBLUCSC | 310 | 6 | 22304 | island | 0.848330 | 0.011421 | NR1H2; KCNC3 (margin: NR1H2; KCNC3; NAPSB) | 10.54 | 2.16e-03 | 1.45e-02 |
|  | 20 | 48099248 | 48099479 | PDFSTATSENSEMBLUCSC | 232 | 7 | 25808 | tss | 0.898502 | 0.010212 | KCNB1 (margin: KCNB1) | 10.52 | 5.83e-04 | 6.47e-03 |
|  | 11 | 22851383 | 22851591 | PDFSTATSENSEMBLUCSC | 209 | 7 | 7204 | tss | 0.933412 | 0.004292 | RP11-17A1.3; SVIP (margin: RP11-17A1.3; SVIP) | 10.50 | 5.83e-04 | 6.47e-03 |
|  | 7 | 83277941 | 83278138 | PDFSTATSENSEMBLUCSC | 198 | 5 | 37068 | gene | 0.776585 | 0.002733 | SEMA3E (margin: SEMA3E) | 10.49 | 7.94e-03 | 3.19e-02 |
|  | 6 | 26044204 | 26044460 | PDFSTATSENSEMBLUCSC | 257 | 6 | 34683 | tss;island | 0.054543 | 0.985655 | U91328.2; HIST1H3C; HIST1H2BB (margin: U91328.2; HIST1H3C; HIST1H3B; HIST1H2AB; HIST1H2BB; HIST1H1C) | 10.44 | 2.16e-03 | 1.45e-02 |
|  | 3 | 197183271 | 197183662 | PDFSTATSENSEMBLUCSC | 392 | 6 | 29817 | island | 0.025600 | 0.960186 | AC128709.2; AC128709.3; AC128709.4 (margin: AC128709.2; AC128709.3; AC128709.4) | 10.43 | 2.16e-03 | 1.45e-02 |
|  | 19 | 37407257 | 37407486 | PDFSTATSENSEMBLUCSC | 230 | 6 | 20006 | gene | 0.909618 | 0.006031 | ZNF568; ZNF829 (margin: ZNF345; ZNF568; ZNF829) | 10.39 | 2.16e-03 | 1.45e-02 |
|  | 1 | 51810823 | 51811185 | PDFSTATSENSEMBLUCSC | 363 | 5 | 1622 | tss | 0.030217 | 0.975769 | TTC39A (margin: RP11-275F13.1; TTC39A; EPS15) | 10.38 | 7.94e-03 | 3.19e-02 |
|  | 15 | 39871808 | 39872186 | PDFSTATSENSEMBLUCSC | 379 | 7 | 13576 | tss;island | 0.003617 | 0.770523 | THBS1 (margin: THBS1) | 10.37 | 5.83e-04 | 6.47e-03 |
|  | 8 | 10586678 | 10587055 | PDFSTATSENSEMBLUCSC | 378 | 7 | 39199 | gene;island | 0.011931 | 0.966725 | CTD-2135J3.3; SOX7 (margin: CTD-2135J3.3; SOX7) | 10.36 | 5.83e-04 | 6.47e-03 |
|  | 3 | 53032638 | 53033167 | PDFSTATSENSEMBLUCSC | 530 | 6 | 27827 | gene;island | 0.029937 | 0.975782 | SFMBT1; RP11-894J14.5 (margin: SFMBT1; RP11-894J14.5) | 10.33 | 2.16e-03 | 1.45e-02 |
|  | 20 | 48099146 | 48099479 | PDFSTATSENSEMBLUCSC | 334 | 8 | 26234 | island | 0.909396 | 0.010603 | KCNB1 (margin: KCNB1) | 10.33 | 2.95e-03 | 1.88e-02 |
|  | 4 | 186456837 | 186457012 | PDFSTATSENSEMBLUCSC | 176 | 5 | 30702 | tss | 0.925963 | 0.011100 | PDLIM3 (margin: PDLIM3) | 10.28 | 7.94e-03 | 3.19e-02 |
|  | 4 | 175750507 | 175751001 | PDFSTATSENSEMBLUCSC | 495 | 10 | 30686 | tss | 0.847449 | 0.013931 | ADAM29; GLRA3 (margin: ADAM29; GLRA3) | 10.27 | 1.08e-05 | 4.82e-04 |
|  | 12 | 21809973 | 21811034 | PDFSTATSENSEMBLUCSC | 1062 | 21 | 10280 | island | 0.014872 | 0.965898 | LDHB (margin: RP11-59N23.3; LDHB) | 10.26 | 3.72e-12 | 5.17e-09 |
|  | 10 | 118032872 | 118033370 | PDFSTATSENSEMBLUCSC | 499 | 11 | 5382 | tss;island | 0.869783 | 0.006086 | GFRA1 (margin: GFRA1) | 10.26 | 2.84e-06 | 1.96e-04 |
|  | 10 | 118032872 | 118033115 | PDFSTATSENSEMBLUCSC | 244 | 7 | 4774 | gene | 0.941245 | 0.006086 | GFRA1 (margin: GFRA1) | 10.26 | 5.83e-04 | 6.47e-03 |
|  | 11 | 22851321 | 22851591 | PDFSTATSENSEMBLUCSC | 271 | 8 | 8137 | island | 0.924659 | 0.009330 | RP11-17A1.3; SVIP (margin: RP11-17A1.3; SVIP) | 10.23 | 1.55e-04 | 2.71e-03 |
|  | 19 | 59066570 | 59066700 | PDFSTATSENSEMBLUCSC | 131 | 7 | 21150 | tss;island | 0.002624 | 0.583028 | CHMP2A; UBE2M (margin: TRIM28; AC016629.8; RN7SL525P; CHMP2A; UBE2M; MZF1) | 10.22 | 5.83e-04 | 6.47e-03 |
|  | 2 | 202122309 | 202122832 | PDFSTATSENSEMBLUCSC | 524 | 8 | 22988 | gene | 0.002760 | 0.875523 | CASP8 (margin: CASP8) | 10.16 | 1.55e-04 | 2.71e-03 |
|  | 17 | 72855943 | 72856181 | PDFSTATSENSEMBLUCSC | 239 | 5 | 18726 | island | 0.045745 | 0.990572 | GRIN2C (margin: GRIN2C; FDXR) | 10.14 | 7.94e-03 | 3.19e-02 |
|  | 11 | 66103823 | 66104174 | PDFSTATSENSEMBLUCSC | 352 | 7 | 8509 | island | 0.004602 | 0.830360 | RP11-867G23.12; RIN1; BRMS1 (margin: RP11-867G23.12; RP11-867G23.8; RIN1; BRMS1; B3GNT1) | 10.13 | 5.83e-04 | 6.47e-03 |
|  | 1 | 85527430 | 85527943 | PDFSTATSENSEMBLUCSC | 514 | 7 | 1721 | tss | 0.008497 | 0.975686 | WDR63 (margin: WDR63) | 10.13 | 5.83e-04 | 6.47e-03 |
|  | 19 | 49223814 | 49224165 | PDFSTATSENSEMBLUCSC | 352 | 5 | 20925 | tss;island | 0.987176 | 0.034829 | MAMSTR; RASIP1 (margin: MAMSTR; RASIP1) | 10.10 | 7.94e-03 | 3.19e-02 |
|  | 6 | 31025933 | 31026287 | PDFSTATSENSEMBLUCSC | 355 | 6 | 33849 | gene | 0.001543 | 0.145543 | HCG22 (margin: HCG22) | 10.09 | 2.16e-03 | 1.45e-02 |
|  | 11 | 10476494 | 10476662 | PDFSTATSENSEMBLUCSC | 169 | 5 | 6506 | gene;tss;island | 0.009191 | 0.897041 | AMPD3 (margin: AMPD3) | 10.09 | 7.94e-03 | 3.19e-02 |
|  | 8 | 145024637 | 145025178 | PDFSTATSENSEMBLUCSC | 542 | 9 | 39502 | gene;island | 0.018046 | 0.958787 | PLEC (margin: PLEC; MIR661) | 10.08 | 4.11e-05 | 1.17e-03 |
|  | 20 | 13976093 | 13976143 | PDFSTATSENSEMBLUCSC | 51 | 6 | 25605 | tss | 0.876097 | 0.005956 | MACROD2; SEL1L2 (margin: MACROD2; RPS3P1; SEL1L2) | 10.08 | 2.16e-03 | 1.45e-02 |
|  | 7 | 27205159 | 27205658 | PDFSTATSENSEMBLUCSC | 500 | 10 | 37496 | tss | 0.045967 | 0.966025 | HOXA9; RP1-170O19.20 (margin: HOXA-AS3; RP1-170O19.21; HOXA-AS4; HOXA7; HOXA9; RP1-170O19.20; MIR196B; HOXA10) | 10.07 | 1.08e-05 | 4.82e-04 |
|  | 3 | 72704324 | 72704701 | PDFSTATSENSEMBLUCSC | 378 | 5 | 29281 | island | 0.938345 | 0.011033 | (margin: ) | 10.06 | 7.94e-03 | 3.19e-02 |
|  | 1 | 21877524 | 21877781 | PDFSTATSENSEMBLUCSC | 258 | 5 | 298 | gene;tss | 0.973677 | 0.025881 | ALPL (margin: ALPL) | 10.03 | 7.94e-03 | 3.19e-02 |
|  | 11 | 12030088 | 12030914 | PDFSTATSENSEMBLUCSC | 827 | 13 | 6511 | gene | 0.022862 | 0.971522 | DKK3 (margin: DKK3) | 10.03 | 1.92e-07 | 2.60e-05 |
|  | 13 | 92050675 | 92050843 | PDFSTATSENSEMBLUCSC | 169 | 9 | 11496 | tss | 0.586370 | 0.007782 | GPC5 (margin: GPC5) | 10.00 | 4.11e-05 | 1.17e-03 |
|  | 7 | 25019747 | 25019943 | PDFSTATSENSEMBLUCSC | 197 | 5 | 38320 | island | 0.019260 | 0.958923 | OSBPL3 (margin: OSBPL3) | 9.98 | 7.94e-03 | 3.19e-02 |
|  | 7 | 134143906 | 134144288 | PDFSTATSENSEMBLUCSC | 383 | 11 | 37911 | tss | 0.024920 | 0.982342 | AKR1B1 (margin: AKR1B1) | 9.97 | 2.84e-06 | 1.96e-04 |
|  | 7 | 99155673 | 99156387 | PDFSTATSENSEMBLUCSC | 715 | 13 | 37760 | tss;island | 0.007404 | 0.896113 | ZNF655; FAM200A (margin: ZNF655; FAM200A) | 9.97 | 1.92e-07 | 2.60e-05 |
|  | 1 | 51810337 | 51811185 | PDFSTATSENSEMBLUCSC | 849 | 8 | 3261 | island | 0.029659 | 0.975807 | TTC39A (margin: RP11-275F13.1; TTC39A; EPS15) | 9.97 | 1.55e-04 | 2.71e-03 |
|  | 6 | 132272342 | 132272790 | PDFSTATSENSEMBLUCSC | 449 | 6 | 36481 | island | 0.564627 | 0.006562 | RP11-69I8.3; CTGF (margin: RP11-69I8.3; CTGF) | 9.96 | 2.16e-03 | 1.45e-02 |
|  | 1 | 228225533 | 228225886 | PDFSTATSENSEMBLUCSC | 354 | 5 | 1045 | gene;island | 0.047340 | 0.978439 | WNT3A (margin: WNT3A) | 9.96 | 7.94e-03 | 3.19e-02 |
|  | 6 | 30070738 | 30071612 | PDFSTATSENSEMBLUCSC | 875 | 27 | 33720 | gene;island | 0.932903 | 0.010832 | TRIM31-AS1; TRIM31 (margin: TRIM31-AS1; TRIM31) | 9.94 | 1.95e-06 | 1.79e-04 |
|  | 19 | 11998686 | 11999148 | PDFSTATSENSEMBLUCSC | 463 | 7 | 19850 | gene | 0.088674 | 0.956262 | ZNF69 (margin: ZNF439; ZNF69) | 9.92 | 5.83e-04 | 6.47e-03 |
|  | 20 | 6103629 | 6104274 | PDFSTATSENSEMBLUCSC | 646 | 7 | 25992 | island | 0.009234 | 0.898500 | FERMT1 (margin: FERMT1) | 9.92 | 1.11e-02 | 4.14e-02 |
|  | 12 | 21810791 | 21811034 | PDFSTATSENSEMBLUCSC | 244 | 9 | 9600 | tss | 0.018450 | 0.956870 | LDHB (margin: RP11-59N23.3; LDHB) | 9.90 | 4.11e-05 | 1.17e-03 |
|  | 19 | 52004992 | 52005188 | PDFSTATSENSEMBLUCSC | 197 | 6 | 20996 | tss | 0.009491 | 0.910986 | SIGLEC12 (margin: CEACAM18; SIGLEC27P; SIGLEC12; SIGLEC26P) | 9.89 | 2.16e-03 | 1.45e-02 |
|  | 6 | 28778091 | 28778676 | PDFSTATSENSEMBLUCSC | 586 | 9 | 35595 | island | 0.951181 | 0.012313 | (margin: ) | 9.89 | 4.11e-05 | 1.17e-03 |
|  | 10 | 116164598 | 116164955 | PDFSTATSENSEMBLUCSC | 358 | 6 | 5377 | tss;island | 0.034999 | 0.973863 | AFAP1L2 (margin: AFAP1L2) | 9.86 | 2.16e-03 | 1.45e-02 |
|  | 2 | 219866424 | 219866628 | PDFSTATSENSEMBLUCSC | 205 | 6 | 25102 | island | 0.507048 | 0.007961 | AC097468.4; MIR375; CCDC108 (margin: AC097468.4; CRYBA2; MIR375; CCDC108) | 9.84 | 4.33e-03 | 2.52e-02 |
|  | 17 | 37123669 | 37123949 | PDFSTATSENSEMBLUCSC | 281 | 8 | 17302 | tss | 0.913609 | 0.017886 | FBXO47 (margin: FBXO47) | 9.84 | 1.55e-04 | 2.71e-03 |
|  | 11 | 117069780 | 117069966 | PDFSTATSENSEMBLUCSC | 187 | 7 | 7717 | tss | 0.028597 | 0.960869 | SIDT2; TAGLN (margin: SIDT2; TAGLN; PCSK7) | 9.83 | 5.83e-04 | 6.47e-03 |
|  | 13 | 29292888 | 29293454 | PDFSTATSENSEMBLUCSC | 567 | 11 | 11642 | island | 0.929417 | 0.016220 | SLC46A3 (margin: SLC46A3; CYP51P2) | 9.83 | 2.84e-06 | 1.96e-04 |
|  | 8 | 98290229 | 98290601 | PDFSTATSENSEMBLUCSC | 373 | 8 | 39829 | tss | 0.961593 | 0.025739 | TSPYL5 (margin: TSPYL5) | 9.83 | 1.55e-04 | 2.71e-03 |
|  | 1 | 13910555 | 13910796 | PDFSTATSENSEMBLUCSC | 242 | 6 | 235 | gene;tss;island | 0.049612 | 0.979461 | PDPN (margin: PDPN) | 9.80 | 2.16e-03 | 1.45e-02 |
|  | 3 | 141087187 | 141087363 | PDFSTATSENSEMBLUCSC | 177 | 5 | 27973 | gene | 0.007575 | 0.812234 | ZBTB38; RP11-438D8.2 (margin: ZBTB38; RP11-438D8.2) | 9.78 | 7.94e-03 | 3.19e-02 |
|  | 16 | 3096477 | 3096662 | PDFSTATSENSEMBLUCSC | 186 | 5 | 15049 | tss | 0.033886 | 0.906769 | MMP25 (margin: RP11-473M20.5; MMP25; CCDC64B; RP11-473M20.7) | 9.77 | 7.94e-03 | 3.19e-02 |
|  | 19 | 52408259 | 52408520 | PDFSTATSENSEMBLUCSC | 262 | 8 | 21000 | tss | 0.039673 | 0.971608 | ZNF649 (margin: CTC-429C10.2; ZNF649) | 9.76 | 1.09e-03 | 1.10e-02 |
|  | 1 | 204183337 | 204183655 | PDFSTATSENSEMBLUCSC | 319 | 7 | 2197 | tss | 0.954737 | 0.011183 | GOLT1A (margin: GOLT1A; PLEKHA6) | 9.74 | 5.83e-04 | 6.47e-03 |
|  | 16 | 4588414 | 4589127 | PDFSTATSENSEMBLUCSC | 714 | 11 | 15671 | island | 0.019614 | 0.977908 | CDIP1 (margin: CDIP1) | 9.72 | 3.40e-05 | 1.17e-03 |
|  | 1 | 43613433 | 43613627 | PDFSTATSENSEMBLUCSC | 195 | 7 | 3158 | island | 0.913609 | 0.012452 | FAM183A (margin: FAM183A) | 9.72 | 5.83e-04 | 6.47e-03 |
|  | 19 | 18335182 | 18335429 | PDFSTATSENSEMBLUCSC | 248 | 5 | 19931 | gene;island | 0.637108 | 0.002962 | PDE4C (margin: AC068499.10; PDE4C) | 9.71 | 7.94e-03 | 3.19e-02 |
|  | 5 | 89854293 | 89854979 | PDFSTATSENSEMBLUCSC | 687 | 11 | 32955 | island | 0.692951 | 0.011913 | GPR98 (margin: GPR98) | 9.70 | 2.84e-06 | 1.96e-04 |
|  | 5 | 89854632 | 89854979 | PDFSTATSENSEMBLUCSC | 348 | 5 | 31687 | gene | 0.918695 | 0.011913 | GPR98 (margin: GPR98) | 9.70 | 7.94e-03 | 3.19e-02 |
|  | 19 | 52430190 | 52430632 | PDFSTATSENSEMBLUCSC | 443 | 7 | 21001 | tss | 0.001913 | 0.480264 | ZNF613 (margin: ZNF613) | 9.70 | 5.83e-04 | 6.47e-03 |
|  | 7 | 134143823 | 134144288 | PDFSTATSENSEMBLUCSC | 466 | 12 | 38935 | island | 0.024656 | 0.978145 | AKR1B1 (margin: AKR1B1) | 9.70 | 7.40e-07 | 7.21e-05 |
|  | 19 | 50832651 | 50832861 | PDFSTATSENSEMBLUCSC | 211 | 5 | 20974 | tss | 0.788778 | 0.016828 | NR1H2; KCNC3 (margin: NR1H2; KCNC3; NAPSB) | 9.69 | 7.94e-03 | 3.19e-02 |
|  | 21 | 42733729 | 42733894 | PDFSTATSENSEMBLUCSC | 166 | 5 | 26556 | tss | 0.022285 | 0.842877 | MX2 (margin: FAM3B; MX2) | 9.66 | 7.94e-03 | 3.19e-02 |
|  | 19 | 1132294 | 1132537 | PDFSTATSENSEMBLUCSC | 244 | 7 | 20293 | tss | 0.021376 | 0.982191 | SBNO2 (margin: SBNO2) | 9.66 | 5.83e-04 | 6.47e-03 |
|  | 4 | 175750109 | 175751001 | PDFSTATSENSEMBLUCSC | 893 | 13 | 31438 | island | 0.913758 | 0.011559 | ADAM29; GLRA3 (margin: ADAM29; GLRA3) | 9.66 | 1.92e-07 | 2.60e-05 |
|  | 11 | 13689411 | 13689686 | PDFSTATSENSEMBLUCSC | 276 | 5 | 7158 | tss;island | 0.411502 | 0.011516 | FAR1; FAR1-IT1 (margin: FAR1; FAR1-IT1) | 9.65 | 7.94e-03 | 3.19e-02 |
|  | 17 | 75315486 | 75315567 | PDFSTATSENSEMBLUCSC | 82 | 5 | 17696 | tss | 0.014663 | 0.851854 | SEPT9 (margin: SEPT9) | 9.64 | 7.94e-03 | 3.19e-02 |
|  | 12 | 106533840 | 106533903 | PDFSTATSENSEMBLUCSC | 64 | 5 | 9966 | tss | 0.058686 | 0.979669 | NUAK1 (margin: NUAK1) | 9.63 | 7.94e-03 | 3.19e-02 |
|  | 3 | 48632484 | 48633144 | PDFSTATSENSEMBLUCSC | 661 | 11 | 29090 | island | 0.013916 | 0.965343 | COL7A1 (margin: COL7A1; UQCRC1) | 9.63 | 2.84e-06 | 1.96e-04 |
|  | 11 | 124587993 | 124588484 | PDFSTATSENSEMBLUCSC | 492 | 5 | 8914 | island | 0.010895 | 0.966803 | (margin: ) | 9.61 | 7.94e-03 | 3.19e-02 |
|  | 7 | 38670804 | 38671001 | PDFSTATSENSEMBLUCSC | 198 | 6 | 36972 | gene;island | 0.725317 | 0.007844 | AMPH (margin: AMPH) | 9.60 | 2.16e-03 | 1.45e-02 |
|  | 1 | 50489654 | 50489954 | PDFSTATSENSEMBLUCSC | 301 | 8 | 1613 | tss | 0.835859 | 0.022435 | AGBL4 (margin: MTND2P29; AGBL4) | 9.60 | 1.55e-04 | 2.71e-03 |
|  | 15 | 45421578 | 45422095 | PDFSTATSENSEMBLUCSC | 518 | 11 | 13646 | tss;island | 0.121457 | 0.979476 | DUOX1; DUOXA1 (margin: DUOXA2; DUOX1; DUOXA1) | 9.60 | 2.84e-06 | 1.96e-04 |
|  | 2 | 176994142 | 176994764 | PDFSTATSENSEMBLUCSC | 623 | 9 | 24905 | island | 0.010562 | 0.932802 | HOXD8; HOXD-AS2 (margin: HOXD10; HOXD9; HOXD8; HOXD3; HOXD-AS2) | 9.59 | 2.76e-03 | 1.78e-02 |
|  | 20 | 52199520 | 52199778 | PDFSTATSENSEMBLUCSC | 259 | 5 | 26263 | island | 0.004588 | 0.853325 | ZNF217 (margin: RP4-724E16.2; ZNF217) | 9.57 | 7.94e-03 | 3.19e-02 |
|  | 1 | 43613433 | 43613567 | PDFSTATSENSEMBLUCSC | 135 | 5 | 1556 | tss | 0.917510 | 0.029188 | FAM183A (margin: FAM183A) | 9.56 | 7.94e-03 | 3.19e-02 |
|  | 18 | 53257019 | 53257340 | PDFSTATSENSEMBLUCSC | 322 | 5 | 19286 | tss;island | 0.042610 | 0.963112 | TCF4 (margin: TCF4) | 9.56 | 7.94e-03 | 3.19e-02 |
|  | 2 | 219858132 | 219858271 | PDFSTATSENSEMBLUCSC | 140 | 6 | 23887 | tss | 0.047441 | 0.967599 | CRYBA2 (margin: AC097468.4; FEV; CRYBA2; MIR375; CCDC108) | 9.55 | 2.16e-03 | 1.45e-02 |
|  | 1 | 24126017 | 24126230 | PDFSTATSENSEMBLUCSC | 214 | 5 | 320 | gene;island | 0.004854 | 0.685825 | GALE (margin: PITHD1; LYPLA2; GALE; HMGCL) | 9.53 | 7.94e-03 | 3.19e-02 |
|  | 2 | 69664772 | 69664869 | PDFSTATSENSEMBLUCSC | 98 | 5 | 23406 | tss | 0.003137 | 0.577149 | NFU1 (margin: NFU1) | 9.49 | 7.94e-03 | 3.19e-02 |
|  | 1 | 2063799 | 2064216 | PDFSTATSENSEMBLUCSC | 418 | 5 | 63 | gene;island | 0.028678 | 0.975813 | PRKCZ (margin: PRKCZ; RP5-892K4.1) | 9.47 | 7.94e-03 | 3.19e-02 |
|  | 2 | 191045309 | 191045668 | PDFSTATSENSEMBLUCSC | 360 | 6 | 23783 | tss | 0.087126 | 0.982349 | C2orf88 (margin: C2orf88; HIBCH) | 9.45 | 2.16e-03 | 1.45e-02 |
|  | 1 | 3615390 | 3615762 | PDFSTATSENSEMBLUCSC | 373 | 5 | 139 | gene | 0.096132 | 0.985532 | TP73 (margin: TP73) | 9.44 | 7.94e-03 | 3.19e-02 |
|  | 13 | 29293196 | 29293454 | PDFSTATSENSEMBLUCSC | 259 | 8 | 11370 | tss | 0.925900 | 0.017420 | SLC46A3 (margin: SLC46A3; CYP51P2) | 9.42 | 1.55e-04 | 2.71e-03 |
|  | 22 | 19710880 | 19711051 | PDFSTATSENSEMBLUCSC | 172 | 6 | 26788 | gene;tss;island | 0.054825 | 0.984456 | SEPT5; GP1BB (margin: SEPT5; GP1BB) | 9.42 | 2.16e-03 | 1.45e-02 |
|  | 7 | 138794512 | 138794752 | PDFSTATSENSEMBLUCSC | 241 | 7 | 37928 | tss | 0.005235 | 0.697692 | ZC3HAV1 (margin: ZC3HAV1) | 9.41 | 5.83e-04 | 6.47e-03 |
|  | 22 | 46481603 | 46481822 | PDFSTATSENSEMBLUCSC | 220 | 7 | 27228 | tss | 0.025801 | 0.961956 | FLJ27365 (margin: FLJ27365; MIR3619) | 9.36 | 5.83e-04 | 6.47e-03 |
|  | 5 | 101632286 | 101632559 | PDFSTATSENSEMBLUCSC | 274 | 9 | 32198 | tss | 0.967476 | 0.047995 | SLCO4C1 (margin: RN7SKP68; SLCO4C1) | 9.35 | 1.65e-04 | 2.80e-03 |
|  | 20 | 19915716 | 19915874 | PDFSTATSENSEMBLUCSC | 159 | 6 | 25369 | gene | 0.058450 | 0.976445 | RIN2 (margin: RIN2) | 9.35 | 2.16e-03 | 1.45e-02 |
|  | 1 | 190447232 | 190447640 | PDFSTATSENSEMBLUCSC | 409 | 5 | 2147 | tss | 0.460310 | 0.010099 | RP11-547I7.2; RP11-161I10.1; FAM5C (margin: RP11-547I7.2; RP11-161I10.1; FAM5C) | 9.35 | 7.94e-03 | 3.19e-02 |
|  | 3 | 32442928 | 32443496 | PDFSTATSENSEMBLUCSC | 569 | 6 | 27709 | gene;island | 0.128735 | 0.978788 | CMTM7 (margin: CMTM7) | 9.34 | 2.16e-03 | 1.45e-02 |
|  | 12 | 133022423 | 133022955 | PDFSTATSENSEMBLUCSC | 533 | 6 | 11058 | island | 0.174085 | 0.962201 | (margin: RP11-503G7.1) | 9.33 | 2.16e-03 | 1.45e-02 |
|  | 1 | 202183264 | 202183605 | PDFSTATSENSEMBLUCSC | 342 | 5 | 945 | gene;island | 0.046696 | 0.960675 | LGR6 (margin: LGR6) | 9.32 | 7.94e-03 | 3.19e-02 |
|  | 19 | 37406932 | 37407486 | PDFSTATSENSEMBLUCSC | 555 | 14 | 20732 | tss;island | 0.825523 | 0.007232 | ZNF568; ZNF829 (margin: ZNF345; ZNF568; ZNF829) | 9.31 | 4.99e-08 | 9.34e-06 |
|  | 1 | 207224090 | 207224388 | PDFSTATSENSEMBLUCSC | 299 | 5 | 975 | gene | 0.007758 | 0.958089 | PFKFB2; YOD1 (margin: PFKFB2; snoU13; YOD1) | 9.29 | 7.94e-03 | 3.19e-02 |
|  | 4 | 91048416 | 91048584 | PDFSTATSENSEMBLUCSC | 169 | 6 | 30498 | tss;island | 0.628345 | 0.005865 | CCSER1 (margin: CCSER1) | 9.28 | 2.16e-03 | 1.45e-02 |
|  | 7 | 138720786 | 138721065 | PDFSTATSENSEMBLUCSC | 280 | 9 | 37927 | tss;island | 0.030965 | 0.980251 | ZC3HAV1L (margin: ZC3HAV1L; ZC3HAV1) | 9.27 | 4.11e-05 | 1.17e-03 |
|  | 11 | 12030088 | 12031508 | PDFSTATSENSEMBLUCSC | 1421 | 21 | 8065 | island | 0.039606 | 0.971522 | DKK3 (margin: DKK3) | 9.26 | 2.72e-08 | 6.41e-06 |
|  | 18 | 59560365 | 59560495 | PDFSTATSENSEMBLUCSC | 131 | 5 | 19301 | tss | 0.762207 | 0.005339 | RNF152 (margin: RNF152) | 9.25 | 7.94e-03 | 3.19e-02 |
|  | 5 | 101631990 | 101632559 | PDFSTATSENSEMBLUCSC | 570 | 12 | 32986 | island | 0.953598 | 0.030635 | SLCO4C1 (margin: RN7SKP68; SLCO4C1) | 9.24 | 2.96e-06 | 2.02e-04 |
|  | 11 | 34535357 | 34535579 | PDFSTATSENSEMBLUCSC | 223 | 7 | 7242 | tss | 0.959613 | 0.037742 | ELF5 (margin: ELF5) | 9.24 | 5.83e-04 | 6.47e-03 |
|  | 6 | 28092048 | 28092370 | PDFSTATSENSEMBLUCSC | 323 | 8 | 34732 | tss | 0.880292 | 0.018527 | ZSCAN16; RP1-265C24.9 (margin: RP1-265C24.5; ZSCAN16; RP1-265C24.9) | 9.24 | 1.55e-04 | 2.71e-03 |
|  | 12 | 6560689 | 6560851 | PDFSTATSENSEMBLUCSC | 163 | 5 | 9028 | gene | 0.015785 | 0.899048 | CD27; TAPBPL; CD27-AS1 (margin: CD27; TAPBPL; CD27-AS1; VAMP1) | 9.20 | 7.94e-03 | 3.19e-02 |
|  | 12 | 56325715 | 56325867 | PDFSTATSENSEMBLUCSC | 153 | 6 | 9169 | gene;island | 0.001860 | 0.458642 | DGKA; WIBG (margin: DGKA; WIBG) | 9.18 | 2.16e-03 | 1.45e-02 |
|  | 2 | 97171137 | 97171449 | PDFSTATSENSEMBLUCSC | 313 | 5 | 22781 | gene;island | 0.004061 | 0.697676 | NEURL3 (margin: NEURL3) | 9.16 | 7.94e-03 | 3.19e-02 |
|  | 17 | 75315486 | 75315868 | PDFSTATSENSEMBLUCSC | 383 | 8 | 16804 | gene | 0.015659 | 0.855331 | SEPT9 (margin: SEPT9) | 9.16 | 1.55e-04 | 2.71e-03 |
|  | 11 | 12030187 | 12031508 | PDFSTATSENSEMBLUCSC | 1322 | 20 | 7153 | tss | 0.035832 | 0.970982 | DKK3 (margin: DKK3) | 9.15 | 1.06e-07 | 1.81e-05 |
|  | 5 | 23507450 | 23507656 | PDFSTATSENSEMBLUCSC | 207 | 9 | 32001 | tss | 0.846754 | 0.018159 | PRDM9 (margin: PRDM9) | 9.13 | 4.11e-05 | 1.17e-03 |
|  | 6 | 132128915 | 132129103 | PDFSTATSENSEMBLUCSC | 189 | 9 | 35224 | tss;island | 0.608125 | 0.004505 | ENPP1 (margin: ENPP1; RP3-323K23.3) | 9.11 | 4.11e-05 | 1.17e-03 |
|  | 12 | 54388841 | 54389264 | PDFSTATSENSEMBLUCSC | 424 | 5 | 10488 | island | 0.024892 | 0.969435 | HOXC5; HOXC6; HOXC9; HOXC-AS2 (margin: HOXC10; HOXC5; HOXC6; MIR196A2; HOXC9; HOXC-AS3; HOXC-AS2; HOXC-AS1) | 9.11 | 7.94e-03 | 3.19e-02 |
|  | 8 | 38964784 | 38965492 | PDFSTATSENSEMBLUCSC | 709 | 11 | 40239 | island | 0.987796 | 0.084596 | ADAM32 (margin: ADAM9; ADAM32) | 9.11 | 1.98e-05 | 8.44e-04 |
|  | 8 | 38964784 | 38965026 | PDFSTATSENSEMBLUCSC | 243 | 7 | 39662 | tss | 0.987257 | 0.084596 | ADAM32 (margin: ADAM9; ADAM32) | 9.11 | 2.33e-03 | 1.52e-02 |
|  | 19 | 12267592 | 12267796 | PDFSTATSENSEMBLUCSC | 205 | 6 | 20490 | tss | 0.018638 | 0.906795 | ZNF625-ZNF20; ZNF625 (margin: ZNF136; ZNF625-ZNF20; ZNF625) | 9.11 | 2.16e-03 | 1.45e-02 |
|  | 4 | 184826215 | 184826479 | PDFSTATSENSEMBLUCSC | 265 | 9 | 30693 | tss;island | 0.874283 | 0.018242 | STOX2 (margin: STOX2) | 9.10 | 4.11e-05 | 1.17e-03 |
|  | 16 | 66638210 | 66638438 | PDFSTATSENSEMBLUCSC | 229 | 8 | 15283 | tss | 0.836301 | 0.009440 | CMTM3 (margin: CMTM3; CMTM4) | 9.10 | 1.55e-04 | 2.71e-03 |
|  | 6 | 28129313 | 28129498 | PDFSTATSENSEMBLUCSC | 186 | 7 | 34734 | tss | 0.038744 | 0.862817 | ZNF192P1 (margin: ZKSCAN8; ZNF192P1) | 9.09 | 5.83e-04 | 6.47e-03 |
|  | 5 | 1882775 | 1883011 | PDFSTATSENSEMBLUCSC | 237 | 8 | 32645 | island | 0.678166 | 0.027929 | CTD-2194D22.3; IRX4 (margin: CTD-2194D22.3; CTD-2194D22.4; IRX4) | 9.08 | 1.55e-04 | 2.71e-03 |
|  | 5 | 1882885 | 1883011 | PDFSTATSENSEMBLUCSC | 127 | 6 | 31974 | tss | 0.726794 | 0.032784 | CTD-2194D22.3; IRX4 (margin: CTD-2194D22.3; CTD-2194D22.4; IRX4) | 9.08 | 2.16e-03 | 1.45e-02 |
|  | 4 | 37455280 | 37455499 | PDFSTATSENSEMBLUCSC | 220 | 7 | 30341 | tss;island | 0.877526 | 0.026067 | C4orf19 (margin: KIAA1239; C4orf19) | 9.08 | 5.83e-04 | 6.47e-03 |
|  | 2 | 75788212 | 75788552 | PDFSTATSENSEMBLUCSC | 341 | 8 | 23469 | tss | 0.128480 | 0.985477 | EVA1A (margin: EVA1A) | 9.08 | 1.55e-04 | 2.71e-03 |
|  | 8 | 58907018 | 58907387 | PDFSTATSENSEMBLUCSC | 370 | 6 | 40321 | island | 0.843875 | 0.015844 | FAM110B (margin: RP11-1112C15.1; FAM110B) | 9.05 | 2.16e-03 | 1.45e-02 |
|  | 2 | 75788038 | 75788552 | PDFSTATSENSEMBLUCSC | 515 | 9 | 22752 | gene;island | 0.133202 | 0.984694 | EVA1A (margin: EVA1A) | 9.03 | 4.11e-05 | 1.17e-03 |
|  | 2 | 219157083 | 219157702 | PDFSTATSENSEMBLUCSC | 620 | 11 | 23014 | gene;island | 0.010717 | 0.942961 | PNKD; TMBIM1 (margin: PNKD; TMBIM1) | 9.03 | 8.51e-05 | 2.07e-03 |
|  | 19 | 37701476 | 37701738 | PDFSTATSENSEMBLUCSC | 263 | 7 | 20735 | tss | 0.384546 | 0.002679 | CTC-454I21.3; ZNF585B (margin: ZNF383; CTC-454I21.3; ZNF585B) | 9.02 | 5.83e-04 | 6.47e-03 |
|  | 1 | 57110686 | 57110878 | PDFSTATSENSEMBLUCSC | 193 | 6 | 1652 | tss;island | 0.684087 | 0.008662 | PRKAA2; PPAP2B (margin: PRKAA2; PPAP2B) | 9.01 | 2.16e-03 | 1.45e-02 |
|  | 19 | 16178091 | 16178253 | PDFSTATSENSEMBLUCSC | 163 | 6 | 20566 | tss | 0.002484 | 0.607286 | CTD-2231E14.4; TPM4 (margin: CTD-2231E14.4; TPM4) | 9.01 | 2.16e-03 | 1.45e-02 |
|  | 12 | 106533667 | 106533903 | PDFSTATSENSEMBLUCSC | 237 | 6 | 10747 | island | 0.054368 | 0.972992 | NUAK1 (margin: NUAK1) | 9.00 | 2.16e-03 | 1.45e-02 |
|  | 5 | 118691415 | 118691794 | PDFSTATSENSEMBLUCSC | 380 | 6 | 31725 | gene;island | 0.003070 | 0.343675 | TNFAIP8 (margin: TNFAIP8) | 8.99 | 2.16e-03 | 1.45e-02 |
|  | 6 | 29855110 | 29855347 | PDFSTATSENSEMBLUCSC | 238 | 7 | 34756 | tss | 0.072403 | 0.966219 | HLA-H; HCG4P7 (margin: HLA-H; HLA-T; HCG4P7) | 8.98 | 5.83e-04 | 6.47e-03 |
|  | 19 | 20150159 | 20150505 | PDFSTATSENSEMBLUCSC | 347 | 7 | 20655 | tss;island | 0.665355 | 0.004091 | ZNF682 (margin: AC006539.3; ZNF682) | 8.96 | 5.83e-04 | 6.47e-03 |
|  | 2 | 191045309 | 191045885 | PDFSTATSENSEMBLUCSC | 577 | 8 | 22960 | gene;island | 0.121313 | 0.978846 | C2orf88 (margin: C2orf88; HIBCH) | 8.95 | 1.55e-04 | 2.71e-03 |
|  | 2 | 69664562 | 69664869 | PDFSTATSENSEMBLUCSC | 308 | 8 | 24369 | island | 0.003569 | 0.519370 | NFU1 (margin: NFU1) | 8.94 | 1.55e-04 | 2.71e-03 |
|  | 2 | 11679845 | 11680057 | PDFSTATSENSEMBLUCSC | 213 | 5 | 23191 | tss | 0.588034 | 0.009326 | GREB1; MIR4429 (margin: GREB1; MIR4429) | 8.94 | 7.94e-03 | 3.19e-02 |
|  | 2 | 238395764 | 238395947 | PDFSTATSENSEMBLUCSC | 184 | 5 | 25230 | island | 0.772013 | 0.008410 | MLPH (margin: MLPH) | 8.94 | 7.94e-03 | 3.19e-02 |
|  | 10 | 30723892 | 30724374 | PDFSTATSENSEMBLUCSC | 483 | 6 | 4550 | gene;island | 0.008516 | 0.820812 | MAP3K8 (margin: MAP3K8) | 8.93 | 2.16e-03 | 1.45e-02 |
|  | 1 | 50489240 | 50489954 | PDFSTATSENSEMBLUCSC | 715 | 15 | 3249 | island | 0.891673 | 0.023798 | AGBL4 (margin: MTND2P29; AGBL4) | 8.92 | 5.80e-07 | 7.07e-05 |
|  | 10 | 43892790 | 43893015 | PDFSTATSENSEMBLUCSC | 226 | 8 | 4570 | gene;tss;island | 0.861726 | 0.015783 | HNRNPF (margin: HNRNPF) | 8.92 | 1.55e-04 | 2.71e-03 |
|  | 14 | 24457893 | 24458014 | PDFSTATSENSEMBLUCSC | 122 | 6 | 12308 | tss | 0.013160 | 0.737206 | DHRS4L2; DHRS4-AS1 (margin: DHRS4L2; DHRS4-AS1) | 8.92 | 2.16e-03 | 1.45e-02 |
|  | 17 | 41446167 | 41446521 | PDFSTATSENSEMBLUCSC | 355 | 5 | 18369 | island | 0.004838 | 0.531986 | LINC00910 (margin: LINC00910) | 8.91 | 7.94e-03 | 3.19e-02 |
|  | 19 | 1401118 | 1401492 | PDFSTATSENSEMBLUCSC | 375 | 5 | 19654 | gene | 0.675243 | 0.004816 | GAMT (margin: NDUFS7; DAZAP1; AC005329.7; GAMT) | 8.87 | 7.94e-03 | 3.19e-02 |
|  | 1 | 155043322 | 155043967 | PDFSTATSENSEMBLUCSC | 646 | 7 | 3806 | island | 0.031025 | 0.980523 | EFNA4; EFNA3 (margin: ADAM15; EFNA4; EFNA3) | 8.86 | 5.83e-04 | 6.47e-03 |
|  | 6 | 166400871 | 166401051 | PDFSTATSENSEMBLUCSC | 181 | 5 | 34541 | gene;island | 0.457473 | 0.003310 | PDE10A; LINC00473 (margin: PDE10A; LINC00473) | 8.86 | 7.94e-03 | 3.19e-02 |
|  | 5 | 89854293 | 89854539 | PDFSTATSENSEMBLUCSC | 247 | 6 | 32174 | tss | 0.635778 | 0.013226 | GPR98 (margin: GPR98) | 8.85 | 2.16e-03 | 1.45e-02 |
|  | 2 | 11679845 | 11680144 | PDFSTATSENSEMBLUCSC | 300 | 7 | 22571 | gene | 0.588034 | 0.028536 | GREB1; MIR4429 (margin: GREB1; MIR4429) | 8.85 | 5.83e-04 | 6.47e-03 |
|  | 19 | 16178091 | 16178570 | PDFSTATSENSEMBLUCSC | 480 | 9 | 21712 | island | 0.004067 | 0.669571 | CTD-2231E14.4; TPM4 (margin: CTD-2231E14.4; TPM4) | 8.83 | 4.11e-05 | 1.17e-03 |
|  | 3 | 48700375 | 48700498 | PDFSTATSENSEMBLUCSC | 124 | 6 | 28305 | tss | 0.422085 | 0.003052 | RP11-148G20.1; CELSR3; NCKIPSD (margin: RP11-148G20.1; RP11-572O6.1; CELSR3; NCKIPSD) | 8.81 | 2.16e-03 | 1.45e-02 |
|  | 5 | 118690955 | 118691126 | PDFSTATSENSEMBLUCSC | 172 | 5 | 31724 | gene;tss;island | 0.004970 | 0.820874 | TNFAIP8 (margin: TNFAIP8) | 8.81 | 7.94e-03 | 3.19e-02 |
|  | 19 | 49127327 | 49127660 | PDFSTATSENSEMBLUCSC | 334 | 5 | 20132 | gene;island | 0.959783 | 0.017284 | SPHK2; AC022154.7 (margin: SPHK2; FAM83E; RPL18; AC022154.7; DBP) | 8.81 | 7.94e-03 | 3.19e-02 |
|  | 1 | 42384310 | 42384647 | PDFSTATSENSEMBLUCSC | 338 | 6 | 3142 | island | 0.604034 | 0.016815 | HIVEP3 (margin: HIVEP3) | 8.79 | 2.16e-03 | 1.45e-02 |
|  | 13 | 84456127 | 84456486 | PDFSTATSENSEMBLUCSC | 360 | 6 | 11186 | gene | 0.966255 | 0.071352 | SLITRK1 (margin: SLITRK1) | 8.78 | 8.66e-03 | 3.36e-02 |
|  | 10 | 17270087 | 17270601 | PDFSTATSENSEMBLUCSC | 515 | 10 | 5578 | island | 0.136589 | 0.983783 | VIM; VIM-AS1 (margin: VIM; VIM-AS1; RP11-124N14.3) | 8.78 | 1.08e-05 | 4.82e-04 |
|  | 11 | 106889318 | 106889745 | PDFSTATSENSEMBLUCSC | 428 | 7 | 7659 | tss;island | 0.386764 | 0.004799 | GUCY1A2 (margin: GUCY1A2) | 8.76 | 1.17e-03 | 1.13e-02 |
|  | 11 | 87908631 | 87908938 | PDFSTATSENSEMBLUCSC | 308 | 8 | 7612 | tss | 0.102286 | 0.971219 | MIR3166; RAB38 (margin: MIR3166; RAB38) | 8.76 | 1.55e-04 | 2.71e-03 |
|  | 17 | 78161467 | 78161572 | PDFSTATSENSEMBLUCSC | 106 | 5 | 17722 | tss | 0.011574 | 0.864894 | CARD14 (margin: CARD14) | 8.76 | 7.94e-03 | 3.19e-02 |
|  | 1 | 153508511 | 153508713 | PDFSTATSENSEMBLUCSC | 203 | 6 | 760 | gene | 0.003945 | 0.606352 | BX470102.3; S100A6; S100A5 (margin: BX470102.3; S100A6; S100A5; S100A4; S100A3) | 8.75 | 2.16e-03 | 1.45e-02 |
|  | 3 | 157824148 | 157824283 | PDFSTATSENSEMBLUCSC | 136 | 5 | 28682 | tss;island | 0.336309 | 0.002194 | RSRC1; SHOX2 (margin: RSRC1; SHOX2) | 8.75 | 7.94e-03 | 3.19e-02 |
|  | 5 | 140105626 | 140105729 | PDFSTATSENSEMBLUCSC | 104 | 6 | 32351 | tss | 0.001389 | 0.377814 | VTRNA1-3 (margin: VTRNA1-2; VTRNA1-3; RP11-515C16.7) | 8.73 | 2.16e-03 | 1.45e-02 |
|  | 22 | 46481603 | 46482023 | PDFSTATSENSEMBLUCSC | 421 | 10 | 27581 | island | 0.065337 | 0.964811 | FLJ27365 (margin: FLJ27365; MIR3619) | 8.73 | 1.08e-05 | 4.82e-04 |
|  | 4 | 156297854 | 156298050 | PDFSTATSENSEMBLUCSC | 197 | 5 | 30158 | gene | 0.860690 | 0.015627 | AC097467.2; MAP9 (margin: AC097467.2; RP11-27G13.5; MAP9; RP11-27G13.3; RP11-27G13.4) | 8.72 | 7.94e-03 | 3.19e-02 |
|  | 3 | 50604613 | 50605269 | PDFSTATSENSEMBLUCSC | 657 | 8 | 29160 | island | 0.051019 | 0.964121 | HEMK1; C3orf18 (margin: HEMK1; C3orf18) | 8.70 | 1.09e-03 | 1.10e-02 |
|  | 17 | 9479910 | 9479965 | PDFSTATSENSEMBLUCSC | 56 | 7 | 17127 | tss;island | 0.387108 | 0.003701 | WDR16; STX8 (margin: WDR16; STX8) | 8.70 | 5.83e-04 | 6.47e-03 |
|  | 18 | 59560091 | 59560495 | PDFSTATSENSEMBLUCSC | 405 | 8 | 19547 | island | 0.787620 | 0.010956 | RNF152 (margin: RNF152) | 8.70 | 1.55e-04 | 2.71e-03 |
|  | 16 | 3096477 | 3096711 | PDFSTATSENSEMBLUCSC | 235 | 6 | 15635 | island | 0.057479 | 0.931068 | MMP25 (margin: RP11-473M20.5; MMP25; CCDC64B; RP11-473M20.7) | 8.69 | 2.16e-03 | 1.45e-02 |
|  | 20 | 3220565 | 3220985 | PDFSTATSENSEMBLUCSC | 421 | 5 | 25969 | island | 0.040605 | 0.956441 | SLC4A11 (margin: SLC4A11; C20orf194) | 8.69 | 7.94e-03 | 3.19e-02 |
|  | 17 | 78161467 | 78161706 | PDFSTATSENSEMBLUCSC | 240 | 6 | 16859 | gene;island | 0.014000 | 0.878414 | CARD14 (margin: CARD14) | 8.69 | 2.16e-03 | 1.45e-02 |
|  | 6 | 30652647 | 30652907 | PDFSTATSENSEMBLUCSC | 261 | 5 | 33803 | gene;island | 0.083283 | 0.967976 | PPP1R18 (margin: DHX16; PPP1R18; NRM; RPL7P4) | 8.68 | 7.94e-03 | 3.19e-02 |
|  | 8 | 143868013 | 143868262 | PDFSTATSENSEMBLUCSC | 250 | 8 | 39939 | tss | 0.026861 | 0.884179 | RP11-706C16.8; LY6D (margin: RP11-706C16.8; LYNX1; LY6D) | 8.67 | 1.55e-04 | 2.71e-03 |
|  | 19 | 6740812 | 6741181 | PDFSTATSENSEMBLUCSC | 370 | 6 | 19771 | gene;island | 0.191525 | 0.968983 | TRIP10 (margin: TRIP10; C3; GPR108; CTD-3128G10.6; SH2D3A) | 8.66 | 2.16e-03 | 1.45e-02 |
|  | 20 | 44540794 | 44541153 | PDFSTATSENSEMBLUCSC | 360 | 5 | 25783 | tss | 0.192369 | 0.988378 | PLTP (margin: PLTP) | 8.66 | 7.94e-03 | 3.19e-02 |
|  | 12 | 104609432 | 104610064 | PDFSTATSENSEMBLUCSC | 633 | 8 | 10736 | island | 0.056908 | 0.982772 | TXNRD1 (margin: TXNRD1) | 8.66 | 1.55e-04 | 2.71e-03 |
|  | 3 | 139258700 | 139258948 | PDFSTATSENSEMBLUCSC | 249 | 7 | 28633 | tss | 0.093914 | 0.974384 | RP11-319G6.1; RBP1 (margin: RP11-319G6.1; RBP1) | 8.65 | 5.83e-04 | 6.47e-03 |
|  | 20 | 44540777 | 44541153 | PDFSTATSENSEMBLUCSC | 377 | 6 | 26209 | island | 0.202694 | 0.988870 | PLTP (margin: PLTP) | 8.65 | 2.16e-03 | 1.45e-02 |
|  | 7 | 107205042 | 107205392 | PDFSTATSENSEMBLUCSC | 351 | 5 | 37180 | gene;tss;island | 0.003187 | 0.673096 | DUS4L; COG5 (margin: DUS4L; COG5) | 8.64 | 7.94e-03 | 3.19e-02 |
|  | 3 | 139258658 | 139258948 | PDFSTATSENSEMBLUCSC | 291 | 8 | 29539 | island | 0.093103 | 0.974421 | RP11-319G6.1; RBP1 (margin: RP11-319G6.1; RBP1) | 8.62 | 1.55e-04 | 2.71e-03 |
|  | 17 | 8771003 | 8771331 | PDFSTATSENSEMBLUCSC | 329 | 5 | 17123 | tss | 0.008925 | 0.540892 | PIK3R6 (margin: PIK3R6; PIK3R5) | 8.62 | 7.94e-03 | 3.19e-02 |
|  | 13 | 88324432 | 88324879 | PDFSTATSENSEMBLUCSC | 448 | 5 | 11809 | island | 0.867562 | 0.021160 | SLITRK5; MIR4500HG (margin: SLITRK5; MIR4500HG) | 8.61 | 7.94e-03 | 3.19e-02 |
|  | 19 | 38794554 | 38794845 | PDFSTATSENSEMBLUCSC | 292 | 6 | 20014 | gene | 0.003595 | 0.543103 | CTB-102L5.4; C19orf33; YIF1B (margin: SPINT2; CTB-102L5.4; Y\_RNA; C19orf33; YIF1B) | 8.60 | 2.16e-03 | 1.45e-02 |
|  | 8 | 49647579 | 49647833 | PDFSTATSENSEMBLUCSC | 255 | 6 | 39301 | gene | 0.079211 | 0.979170 | EFCAB1 (margin: EFCAB1) | 8.60 | 2.16e-03 | 1.45e-02 |
|  | 10 | 729479 | 729956 | PDFSTATSENSEMBLUCSC | 478 | 6 | 4451 | gene | 0.052854 | 0.978291 | DIP2C (margin: DIP2C) | 8.60 | 2.16e-03 | 1.45e-02 |
|  | 19 | 9434309 | 9434924 | PDFSTATSENSEMBLUCSC | 616 | 10 | 20422 | tss | 0.029063 | 0.800087 | ZNF559; ZNF177 (margin: ZNF559; ZNF177) | 8.60 | 1.08e-05 | 4.82e-04 |
|  | 14 | 100626084 | 100626205 | PDFSTATSENSEMBLUCSC | 122 | 6 | 12602 | tss;island | 0.827248 | 0.015392 | DEGS2 (margin: DEGS2) | 8.60 | 2.16e-03 | 1.45e-02 |
|  | 17 | 7530871 | 7531394 | PDFSTATSENSEMBLUCSC | 524 | 13 | 16406 | gene;tss;island | 0.003860 | 0.461972 | SHBG; SAT2 (margin: SHBG; SAT2) | 8.59 | 1.92e-07 | 2.60e-05 |
|  | 1 | 159046773 | 159047163 | PDFSTATSENSEMBLUCSC | 391 | 5 | 2028 | tss | 0.018595 | 0.877316 | AIM2 (margin: AIM2; RP11-520H16.4) | 8.58 | 7.94e-03 | 3.19e-02 |
|  | 14 | 24457893 | 24458345 | PDFSTATSENSEMBLUCSC | 453 | 10 | 12723 | island | 0.010836 | 0.720686 | DHRS4L2; DHRS4-AS1 (margin: DHRS4L2; DHRS4-AS1) | 8.58 | 1.08e-05 | 4.82e-04 |
|  | 19 | 38085706 | 38085810 | PDFSTATSENSEMBLUCSC | 105 | 6 | 20008 | gene;tss;island | 0.401397 | 0.001503 | ZNF540; ZNF571 (margin: ZNF571-AS1; ZNF540; ZNF571) | 8.57 | 2.16e-03 | 1.45e-02 |
|  | 16 | 69141250 | 69141478 | PDFSTATSENSEMBLUCSC | 229 | 7 | 14817 | gene;island | 0.181953 | 0.984359 | HAS3 (margin: HAS3; CHTF8) | 8.54 | 5.83e-04 | 6.47e-03 |
|  | 16 | 69141250 | 69141437 | PDFSTATSENSEMBLUCSC | 188 | 5 | 15326 | tss | 0.106971 | 0.977922 | HAS3 (margin: HAS3; CHTF8) | 8.54 | 7.94e-03 | 3.19e-02 |
|  | 8 | 67940640 | 67941178 | PDFSTATSENSEMBLUCSC | 539 | 10 | 40363 | island | 0.645954 | 0.007102 | PPP1R42 (margin: PPP1R42) | 8.54 | 1.08e-05 | 4.82e-04 |
|  | 22 | 45596948 | 45597035 | PDFSTATSENSEMBLUCSC | 88 | 7 | 27218 | tss | 0.140214 | 0.984315 | KIAA0930; MIR1249 (margin: KIAA0930; MIR1249) | 8.53 | 5.83e-04 | 6.47e-03 |
|  | 22 | 45596797 | 45597035 | PDFSTATSENSEMBLUCSC | 239 | 8 | 26899 | gene;island | 0.153226 | 0.983490 | KIAA0930; MIR1249 (margin: KIAA0930; MIR1249) | 8.53 | 1.55e-04 | 2.71e-03 |
|  | 4 | 47839876 | 47840162 | PDFSTATSENSEMBLUCSC | 287 | 6 | 31025 | island | 0.151099 | 0.973189 | CORIN; RP11-121C2.2 (margin: CORIN; RP11-121C2.2; NFXL1) | 8.52 | 2.16e-03 | 1.45e-02 |
|  | 19 | 2427708 | 2428122 | PDFSTATSENSEMBLUCSC | 415 | 5 | 21284 | island | 0.008211 | 0.513268 | TMPRSS9; TIMM13; LMNB2 (margin: TMPRSS9; TIMM13; LMNB2) | 8.51 | 7.94e-03 | 3.19e-02 |
|  | 5 | 42949542 | 42950152 | PDFSTATSENSEMBLUCSC | 611 | 6 | 32761 | island | 0.216285 | 0.985877 | (margin: ) | 8.51 | 2.16e-03 | 1.45e-02 |
|  | 19 | 31640642 | 31640711 | PDFSTATSENSEMBLUCSC | 70 | 5 | 20673 | tss | 0.178352 | 0.984016 | AC020952.1 (margin: AC020952.1) | 8.51 | 7.94e-03 | 3.19e-02 |
|  | 12 | 123215151 | 123215684 | PDFSTATSENSEMBLUCSC | 534 | 9 | 10086 | tss | 0.945788 | 0.019804 | HCAR1 (margin: HCAR1; RP11-324E6.9) | 8.50 | 4.11e-05 | 1.17e-03 |
|  | 4 | 187476326 | 187477065 | PDFSTATSENSEMBLUCSC | 740 | 11 | 31483 | island | 0.810564 | 0.010342 | RP11-215A19.2; MTNR1A (margin: RP11-215A19.2; MTNR1A) | 8.48 | 3.94e-04 | 5.86e-03 |
|  | 4 | 187476543 | 187477065 | PDFSTATSENSEMBLUCSC | 523 | 9 | 30707 | tss | 0.810564 | 0.010342 | RP11-215A19.2; MTNR1A (margin: RP11-215A19.2; MTNR1A) | 8.48 | 2.76e-03 | 1.78e-02 |
|  | 12 | 107714673 | 107715146 | PDFSTATSENSEMBLUCSC | 474 | 7 | 9283 | gene;island | 0.076962 | 0.964106 | BTBD11 (margin: BTBD11) | 8.45 | 5.83e-04 | 6.47e-03 |
|  | 4 | 186456397 | 186457012 | PDFSTATSENSEMBLUCSC | 616 | 9 | 31477 | island | 0.836308 | 0.009316 | PDLIM3 (margin: PDLIM3) | 8.44 | 4.11e-05 | 1.17e-03 |
|  | 11 | 94278324 | 94278603 | PDFSTATSENSEMBLUCSC | 280 | 8 | 6867 | gene;island | 0.118704 | 0.979869 | PIWIL4; FUT4; RP11-867G2.8 (margin: PIWIL4; FUT4; RP11-867G2.8) | 8.43 | 1.55e-04 | 2.71e-03 |
|  | 10 | 128994432 | 128994702 | PDFSTATSENSEMBLUCSC | 271 | 6 | 5434 | tss | 0.739762 | 0.007243 | DOCK1; FAM196A (margin: DOCK1; FAM196A) | 8.43 | 2.16e-03 | 1.45e-02 |
|  | 19 | 48896922 | 48897279 | PDFSTATSENSEMBLUCSC | 358 | 6 | 20914 | tss;island | 0.834172 | 0.008599 | GRIN2D (margin: GRIN2D; KDELR1) | 8.43 | 2.16e-03 | 1.45e-02 |
|  | 2 | 119603383 | 119603969 | PDFSTATSENSEMBLUCSC | 587 | 5 | 22841 | gene;island | 0.059361 | 0.976684 | EN1 (margin: RP11-19E11.1; EN1) | 8.43 | 7.94e-03 | 3.19e-02 |
|  | 6 | 76059450 | 76059773 | PDFSTATSENSEMBLUCSC | 324 | 6 | 34396 | gene | 0.741377 | 0.029618 | FILIP1 (margin: RP11-415D17.3; FILIP1) | 8.43 | 4.33e-03 | 2.52e-02 |
|  | 13 | 78272372 | 78272639 | PDFSTATSENSEMBLUCSC | 268 | 7 | 11789 | island | 0.656925 | 0.007208 | SLAIN1; MIR3665 (margin: SLAIN1; MIR3665) | 8.42 | 5.83e-04 | 6.47e-03 |
|  | 12 | 81471867 | 81472177 | PDFSTATSENSEMBLUCSC | 311 | 6 | 9228 | gene | 0.676889 | 0.007213 | ACSS3 (margin: ACSS3) | 8.42 | 2.16e-03 | 1.45e-02 |
|  | 5 | 63802106 | 63802412 | PDFSTATSENSEMBLUCSC | 307 | 5 | 32089 | tss | 0.906224 | 0.021361 | RGS7BP (margin: RGS7BP) | 8.42 | 7.94e-03 | 3.19e-02 |
|  | 1 | 57111099 | 57111511 | PDFSTATSENSEMBLUCSC | 413 | 8 | 553 | gene;island | 0.678122 | 0.009737 | PRKAA2; PPAP2B (margin: PRKAA2; PPAP2B) | 8.40 | 1.55e-04 | 2.71e-03 |
|  | 22 | 27053195 | 27053470 | PDFSTATSENSEMBLUCSC | 276 | 10 | 27037 | tss | 0.218240 | 0.988351 | MIAT (margin: MIAT; ISCA2P1; CTA-373H7.7) | 8.40 | 1.08e-05 | 4.82e-04 |
|  | 19 | 9434309 | 9435206 | PDFSTATSENSEMBLUCSC | 898 | 13 | 21496 | island | 0.047853 | 0.872710 | ZNF559; ZNF177 (margin: ZNF559; ZNF177) | 8.39 | 1.92e-07 | 2.60e-05 |
|  | 3 | 113251061 | 113251417 | PDFSTATSENSEMBLUCSC | 357 | 5 | 29366 | island | 0.109499 | 0.000906 | SIDT1 (margin: SIDT1) | 8.38 | 7.94e-03 | 3.19e-02 |
|  | 1 | 55267006 | 55267293 | PDFSTATSENSEMBLUCSC | 288 | 7 | 1648 | tss | 0.009138 | 0.857175 | TTC22 (margin: RP11-67L3.2; C1orf177; TTC22) | 8.37 | 5.83e-04 | 6.47e-03 |
|  | 1 | 8271918 | 8272277 | PDFSTATSENSEMBLUCSC | 360 | 5 | 2634 | island | 0.008565 | 0.622882 | RP11-431K24.3 (margin: RP11-431K24.3; RNU1-7P; RP11-431K24.4) | 8.37 | 7.94e-03 | 3.19e-02 |
|  | 6 | 39281421 | 39281885 | PDFSTATSENSEMBLUCSC | 465 | 5 | 34325 | gene;island | 0.090577 | 0.970292 | KCNK17; KCNK16 (margin: KCNK17; KCNK16) | 8.36 | 7.94e-03 | 3.19e-02 |
|  | 14 | 61116048 | 61116506 | PDFSTATSENSEMBLUCSC | 459 | 7 | 12907 | island | 0.004193 | 0.548357 | SIX1 (margin: RP11-1042B17.5; SIX1) | 8.35 | 5.83e-04 | 6.47e-03 |
|  | 14 | 61116211 | 61116506 | PDFSTATSENSEMBLUCSC | 296 | 5 | 12439 | tss | 0.004213 | 0.554423 | SIX1 (margin: RP11-1042B17.5; SIX1) | 8.35 | 7.94e-03 | 3.19e-02 |
|  | 10 | 128994297 | 128994702 | PDFSTATSENSEMBLUCSC | 406 | 7 | 4818 | gene;island | 0.736439 | 0.008229 | DOCK1; FAM196A (margin: DOCK1; FAM196A) | 8.34 | 5.83e-04 | 6.47e-03 |
|  | 8 | 13424080 | 13424269 | PDFSTATSENSEMBLUCSC | 190 | 5 | 39561 | tss | 0.481329 | 0.004359 | C8orf48; RP11-145O15.3 (margin: C8orf48; RP11-145O15.3) | 8.34 | 7.94e-03 | 3.19e-02 |
|  | 11 | 64014518 | 64014598 | PDFSTATSENSEMBLUCSC | 81 | 5 | 7390 | tss;island | 0.461853 | 0.013209 | RP11-783K16.5; PPP1R14B; RP11-783K16.13 (margin: VEGFB; FKBP2; RP11-783K16.5; PLCB3; PPP1R14B; RP11-783K16.13) | 8.34 | 7.94e-03 | 3.19e-02 |
|  | 11 | 2170870 | 2171184 | PDFSTATSENSEMBLUCSC | 315 | 5 | 7059 | tss | 0.031741 | 0.753333 | IGF2-AS; IGF2; INS-IGF2 (margin: IGF2-AS; IGF2; INS-IGF2; INS) | 8.33 | 7.94e-03 | 3.19e-02 |
|  | 1 | 179557438 | 179558114 | PDFSTATSENSEMBLUCSC | 677 | 9 | 3999 | island | 0.099654 | 0.931889 | RP11-545A16.3; RP11-545A16.4 (margin: RNU5F-2P; RP11-545A16.3; RP11-545A16.4; TDRD5) | 8.31 | 2.76e-03 | 1.78e-02 |
|  | 22 | 27053195 | 27053934 | PDFSTATSENSEMBLUCSC | 740 | 15 | 27379 | island | 0.211227 | 0.988579 | MIAT (margin: MIAT; ISCA2P1; CTA-373H7.7) | 8.31 | 5.16e-08 | 9.48e-06 |
|  | 18 | 55862577 | 55862872 | PDFSTATSENSEMBLUCSC | 296 | 7 | 19120 | gene;island | 0.115909 | 0.976980 | NEDD4L; RP11-718I15.1 (margin: NEDD4L; RP11-718I15.1) | 8.31 | 5.83e-04 | 6.47e-03 |
|  | 19 | 12267308 | 12267796 | PDFSTATSENSEMBLUCSC | 489 | 8 | 21595 | island | 0.025189 | 0.906795 | ZNF625-ZNF20; ZNF625 (margin: ZNF136; ZNF625-ZNF20; ZNF625) | 8.28 | 1.55e-04 | 2.71e-03 |
|  | 13 | 53425635 | 53426032 | PDFSTATSENSEMBLUCSC | 398 | 5 | 11764 | island | 0.386905 | 0.001581 | (margin: PCDH8) | 8.28 | 7.94e-03 | 3.19e-02 |
|  | 8 | 23021325 | 23021690 | PDFSTATSENSEMBLUCSC | 366 | 6 | 40146 | island | 0.136673 | 0.976092 | TNFRSF10D (margin: TNFRSF10D) | 8.27 | 2.16e-03 | 1.45e-02 |
|  | 12 | 6560689 | 6561007 | PDFSTATSENSEMBLUCSC | 319 | 7 | 9511 | tss | 0.015785 | 0.898030 | CD27; TAPBPL; CD27-AS1 (margin: CD27; TAPBPL; CD27-AS1; VAMP1) | 8.27 | 5.83e-04 | 6.47e-03 |
|  | 6 | 5996893 | 5997375 | PDFSTATSENSEMBLUCSC | 483 | 6 | 35404 | island | 0.023421 | 0.836363 | NRN1 (margin: NRN1) | 8.26 | 2.16e-03 | 1.45e-02 |
|  | 1 | 31158026 | 31158299 | PDFSTATSENSEMBLUCSC | 274 | 5 | 2964 | island | 0.875772 | 0.023554 | (margin: ) | 8.25 | 7.94e-03 | 3.19e-02 |
|  | 11 | 2170831 | 2171184 | PDFSTATSENSEMBLUCSC | 354 | 6 | 6449 | gene | 0.037514 | 0.749320 | IGF2-AS; IGF2; INS-IGF2 (margin: IGF2-AS; IGF2; INS-IGF2; INS) | 8.24 | 2.16e-03 | 1.45e-02 |
|  | 1 | 109941043 | 109941201 | PDFSTATSENSEMBLUCSC | 159 | 5 | 1802 | tss;island | 0.946062 | 0.037674 | SORT1; PSMA5 (margin: SORT1; PSMA5) | 8.24 | 7.94e-03 | 3.19e-02 |
|  | 19 | 37569236 | 37569736 | PDFSTATSENSEMBLUCSC | 501 | 11 | 21950 | island | 0.802375 | 0.006859 | ZNF420; CTD-2293H3.2 (margin: ZNF420; CTD-2293H3.2) | 8.23 | 2.84e-06 | 1.96e-04 |
|  | 19 | 37569236 | 37569352 | PDFSTATSENSEMBLUCSC | 117 | 7 | 20733 | tss | 0.802375 | 0.006859 | ZNF420; CTD-2293H3.2 (margin: ZNF420; CTD-2293H3.2) | 8.23 | 5.83e-04 | 6.47e-03 |
|  | 6 | 87647370 | 87647644 | PDFSTATSENSEMBLUCSC | 275 | 5 | 34408 | gene;island | 0.926751 | 0.015316 | HTR1E (margin: HTR1E) | 8.21 | 7.94e-03 | 3.19e-02 |
|  | 8 | 61193715 | 61194292 | PDFSTATSENSEMBLUCSC | 578 | 11 | 40329 | island | 0.823630 | 0.013995 | CA8 (margin: CA8) | 8.21 | 2.84e-06 | 1.96e-04 |
|  | 7 | 94537584 | 94537893 | PDFSTATSENSEMBLUCSC | 310 | 9 | 37094 | gene;island | 0.823709 | 0.011077 | PPP1R9A (margin: PPP1R9A) | 8.20 | 4.11e-05 | 1.17e-03 |
|  | 16 | 88943191 | 88943641 | PDFSTATSENSEMBLUCSC | 451 | 6 | 14920 | gene;island | 0.178678 | 0.972056 | CBFA2T3 (margin: RP11-830F9.5; PABPN1L; CBFA2T3) | 8.20 | 2.16e-03 | 1.45e-02 |
|  | 1 | 2222253 | 2222674 | PDFSTATSENSEMBLUCSC | 422 | 5 | 72 | gene;island | 0.114408 | 0.954061 | SKI (margin: SKI) | 8.20 | 7.94e-03 | 3.19e-02 |
|  | 17 | 78800574 | 78800806 | PDFSTATSENSEMBLUCSC | 233 | 5 | 16875 | gene;island | 0.109899 | 0.972621 | RPTOR (margin: RPTOR) | 8.19 | 7.94e-03 | 3.19e-02 |
|  | 1 | 55266578 | 55267293 | PDFSTATSENSEMBLUCSC | 716 | 10 | 3302 | island | 0.019140 | 0.895996 | TTC22 (margin: RP11-67L3.2; C1orf177; TTC22) | 8.19 | 1.08e-05 | 4.82e-04 |
|  | 1 | 32714023 | 32714308 | PDFSTATSENSEMBLUCSC | 286 | 5 | 392 | gene;island | 0.871854 | 0.028445 | FAM167B (margin: FAM167B; LCK; MTMR9LP) | 8.18 | 7.94e-03 | 3.19e-02 |
|  | 4 | 13546145 | 13546673 | PDFSTATSENSEMBLUCSC | 529 | 9 | 30304 | tss | 0.739958 | 0.017081 | NKX3-2; AC006445.8 (margin: NKX3-2; AC006445.8) | 8.18 | 7.82e-04 | 8.17e-03 |
|  | 1 | 82267923 | 82268281 | PDFSTATSENSEMBLUCSC | 359 | 5 | 597 | gene;island | 0.479268 | 0.007504 | LPHN2 (margin: LPHN2) | 8.17 | 7.94e-03 | 3.19e-02 |
|  | 13 | 43566401 | 43566642 | PDFSTATSENSEMBLUCSC | 242 | 9 | 11414 | tss | 0.085550 | 0.970030 | EPSTI1 (margin: EPSTI1) | 8.17 | 4.11e-05 | 1.17e-03 |
|  | 8 | 121824218 | 121824929 | PDFSTATSENSEMBLUCSC | 712 | 10 | 40565 | island | 0.726989 | 0.007600 | RP11-713M15.2; SNTB1 (margin: RP11-713M15.2; SNTB1) | 8.17 | 1.05e-03 | 1.08e-02 |
|  | 17 | 80806135 | 80806435 | PDFSTATSENSEMBLUCSC | 301 | 5 | 16960 | gene | 0.078442 | 0.968522 | TBCD (margin: TBCD; ZNF750) | 8.17 | 7.94e-03 | 3.19e-02 |
|  | 12 | 15374457 | 15374609 | PDFSTATSENSEMBLUCSC | 153 | 6 | 9590 | tss | 0.687846 | 0.012008 | RERG (margin: RERG) | 8.17 | 2.16e-03 | 1.45e-02 |
|  | 6 | 29894619 | 29894908 | PDFSTATSENSEMBLUCSC | 290 | 11 | 34758 | tss | 0.038503 | 0.917549 | HLA-K; HCG4B (margin: HLA-K; HLA-U; HCG4B) | 8.14 | 5.67e-06 | 3.54e-04 |
|  | 6 | 28411279 | 28411423 | PDFSTATSENSEMBLUCSC | 145 | 6 | 34742 | tss | 0.100601 | 0.944974 | ZSCAN23 (margin: COX11P1; ZSCAN23) | 8.13 | 2.16e-03 | 1.45e-02 |
|  | 4 | 8582101 | 8582300 | PDFSTATSENSEMBLUCSC | 200 | 7 | 30922 | island | 0.657911 | 0.010474 | GPR78 (margin: GPR78) | 8.13 | 5.83e-04 | 6.47e-03 |
|  | 15 | 89438611 | 89438964 | PDFSTATSENSEMBLUCSC | 354 | 7 | 14423 | island | 0.107684 | 0.965608 | HAPLN3 (margin: HAPLN3; MFGE8) | 8.13 | 5.83e-04 | 6.47e-03 |
|  | 19 | 35645556 | 35645712 | PDFSTATSENSEMBLUCSC | 157 | 7 | 20701 | tss;island | 0.002698 | 0.594460 | FXYD7; FXYD5 (margin: FXYD1; CTD-2527I21.4; FXYD7; FXYD5) | 8.13 | 5.83e-04 | 6.47e-03 |
|  | 10 | 530635 | 531584 | PDFSTATSENSEMBLUCSC | 950 | 12 | 4441 | gene;island | 0.099031 | 0.974379 | DIP2C (margin: DIP2C) | 8.12 | 7.40e-07 | 7.21e-05 |
|  | 14 | 91580380 | 91580613 | PDFSTATSENSEMBLUCSC | 234 | 6 | 12176 | gene | 0.033793 | 0.892250 | C14orf159 (margin: C14orf159) | 8.12 | 2.16e-03 | 1.45e-02 |
|  | 19 | 50194120 | 50194252 | PDFSTATSENSEMBLUCSC | 133 | 5 | 20961 | tss;island | 0.202623 | 0.977257 | PRMT1; ADM5; CPT1C; CTB-33G10.6 (margin: PRMT1; MIR5088; ADM5; CPT1C; CTB-33G10.6) | 8.11 | 7.94e-03 | 3.19e-02 |
|  | 10 | 119302050 | 119302318 | PDFSTATSENSEMBLUCSC | 269 | 8 | 4785 | gene;island | 0.132328 | 0.979747 | EMX2; EMX2OS (margin: EMX2; EMX2OS) | 8.10 | 1.55e-04 | 2.71e-03 |
|  | 8 | 61193970 | 61194292 | PDFSTATSENSEMBLUCSC | 323 | 9 | 39724 | tss | 0.823630 | 0.015638 | CA8 (margin: CA8) | 8.10 | 4.11e-05 | 1.17e-03 |
|  | 11 | 87908558 | 87908938 | PDFSTATSENSEMBLUCSC | 381 | 9 | 8719 | island | 0.069801 | 0.964408 | MIR3166; RAB38 (margin: MIR3166; RAB38) | 8.09 | 4.11e-05 | 1.17e-03 |
|  | 1 | 153508511 | 153508875 | PDFSTATSENSEMBLUCSC | 365 | 10 | 3763 | island | 0.005899 | 0.606352 | BX470102.3; S100A6; S100A5 (margin: BX470102.3; S100A6; S100A5; S100A4; S100A3) | 8.08 | 1.08e-05 | 4.82e-04 |
|  | 7 | 93520074 | 93520566 | PDFSTATSENSEMBLUCSC | 493 | 13 | 37715 | tss | 0.839425 | 0.031040 | GNGT1; AC002076.10; TFPI2 (margin: GNGT1; AC002076.10; TFPI2) | 8.08 | 1.92e-07 | 2.60e-05 |
|  | 11 | 124311131 | 124311423 | PDFSTATSENSEMBLUCSC | 293 | 5 | 7782 | tss | 0.116300 | 0.978830 | OR8B8 (margin: OR8B8) | 8.08 | 7.94e-03 | 3.19e-02 |
|  | 2 | 220083306 | 220083951 | PDFSTATSENSEMBLUCSC | 646 | 10 | 25110 | island | 0.753240 | 0.010149 | ABCB6; ATG9A (margin: ZFAND2B; ANKZF1; ABCB6; ATG9A; AC068946.1) | 8.07 | 1.08e-05 | 4.82e-04 |
|  | 2 | 220083696 | 220083951 | PDFSTATSENSEMBLUCSC | 256 | 8 | 23895 | tss | 0.770464 | 0.010149 | ABCB6; ATG9A (margin: ZFAND2B; ANKZF1; ABCB6; ATG9A; AC068946.1) | 8.07 | 1.55e-04 | 2.71e-03 |
|  | 11 | 120530774 | 120530973 | PDFSTATSENSEMBLUCSC | 200 | 5 | 7764 | tss | 0.046716 | 0.935960 | GRIK4 (margin: GRIK4) | 8.06 | 7.94e-03 | 3.19e-02 |
|  | 16 | 604802 | 605347 | PDFSTATSENSEMBLUCSC | 546 | 6 | 15462 | island | 0.127057 | 0.972312 | CAPN15; LA16c-366D1.3 (margin: CAPN15; MIR3176; C16orf11; PIGQ; NHLRC4; LA16c-366D1.3) | 8.05 | 2.16e-03 | 1.45e-02 |
|  | 15 | 99789622 | 99789855 | PDFSTATSENSEMBLUCSC | 234 | 5 | 14503 | island | 0.003573 | 0.426038 | LRRC28; TTC23 (margin: LRRC28; AC022819.1; HSP90B2P; TTC23) | 8.05 | 7.94e-03 | 3.19e-02 |
|  | 11 | 101000436 | 101000717 | PDFSTATSENSEMBLUCSC | 282 | 6 | 8755 | island | 0.312186 | 0.013126 | PGR (margin: PGR) | 8.03 | 2.16e-03 | 1.45e-02 |
|  | 19 | 50706387 | 50706874 | PDFSTATSENSEMBLUCSC | 488 | 7 | 20973 | tss;island | 0.918338 | 0.023526 | MYH14 (margin: MYH14) | 8.03 | 5.83e-04 | 6.47e-03 |
|  | 7 | 94537716 | 94537893 | PDFSTATSENSEMBLUCSC | 178 | 8 | 37723 | tss | 0.817289 | 0.010912 | PPP1R9A (margin: PPP1R9A) | 8.03 | 1.55e-04 | 2.71e-03 |
|  | 6 | 146285286 | 146285836 | PDFSTATSENSEMBLUCSC | 551 | 11 | 35274 | tss;island | 0.003789 | 0.402435 | SHPRH (margin: SHPRH) | 8.03 | 8.51e-05 | 2.07e-03 |
|  | 5 | 140220686 | 140221106 | PDFSTATSENSEMBLUCSC | 421 | 6 | 31799 | gene;island | 0.841240 | 0.018679 | PCDHA1; PCDHA2; PCDHA3; PCDHA4; PCDHA5; PCDHA6; PCDHA7; PCDHA8 (margin: PCDHA1; PCDHA2; PCDHA3; PCDHA4; PCDHA5; PCDHA6; PCDHA7; PCDHA8; PCDHA9) | 8.02 | 2.16e-03 | 1.45e-02 |
|  | 11 | 818752 | 818917 | PDFSTATSENSEMBLUCSC | 166 | 5 | 7884 | island | 0.017277 | 0.544300 | PNPLA2 (margin: RPLP2; SNORA52; PNPLA2; EFCAB4A; PIDD; AP006621.8) | 8.02 | 7.94e-03 | 3.19e-02 |
|  | 16 | 67918484 | 67918965 | PDFSTATSENSEMBLUCSC | 482 | 8 | 16033 | island | 0.902547 | 0.031086 | EDC4; CTC-479C5.10; NRN1L (margin: EDC4; AC040162.1; CTC-479C5.10; NRN1L; PSKH1) | 8.02 | 1.86e-03 | 1.45e-02 |
|  | 1 | 3607097 | 3607425 | PDFSTATSENSEMBLUCSC | 329 | 7 | 138 | gene;island | 0.130280 | 0.979487 | TP73 (margin: TP73) | 8.01 | 5.83e-04 | 6.47e-03 |
|  | 7 | 27204663 | 27205658 | PDFSTATSENSEMBLUCSC | 996 | 17 | 38356 | island | 0.085212 | 0.964733 | HOXA9; RP1-170O19.20 (margin: HOXA-AS3; RP1-170O19.21; HOXA-AS4; HOXA7; HOXA9; RP1-170O19.20; MIR196B; HOXA10) | 8.01 | 8.57e-10 | 3.90e-07 |
|  | 2 | 97760606 | 97760749 | PDFSTATSENSEMBLUCSC | 144 | 7 | 23527 | tss;island | 0.008109 | 0.588898 | FAHD2B (margin: AC018892.9; RN7SL313P; FAHD2B) | 8.01 | 5.83e-04 | 6.47e-03 |
|  | 8 | 110703888 | 110704289 | PDFSTATSENSEMBLUCSC | 402 | 6 | 40541 | island | 0.681649 | 0.008420 | SYBU (margin: SYBU) | 7.99 | 2.16e-03 | 1.45e-02 |
|  | 7 | 45961772 | 45962236 | PDFSTATSENSEMBLUCSC | 465 | 13 | 37588 | tss;island | 0.840260 | 0.031424 | IGFBP3 (margin: IGFBP3) | 7.98 | 1.92e-07 | 2.60e-05 |
|  | 1 | 35220224 | 35220710 | PDFSTATSENSEMBLUCSC | 487 | 6 | 1478 | tss | 0.080881 | 0.961439 | GJB5; SMIM12 (margin: GJB5; GJB4; SMIM12; RP1-34M23.5) | 7.98 | 2.16e-03 | 1.45e-02 |
|  | 19 | 11531294 | 11531519 | PDFSTATSENSEMBLUCSC | 226 | 5 | 19840 | gene;island | 0.816227 | 0.026545 | RGL3; CCDC151 (margin: Y\_RNA; RGL3; CCDC151) | 7.97 | 7.94e-03 | 3.19e-02 |
|  | 2 | 227700458 | 227700568 | PDFSTATSENSEMBLUCSC | 111 | 6 | 23928 | tss;island | 0.731950 | 0.021903 | RHBDD1 (margin: RHBDD1) | 7.96 | 2.16e-03 | 1.45e-02 |
|  | 12 | 7282081 | 7282931 | PDFSTATSENSEMBLUCSC | 851 | 14 | 9546 | tss | 0.661019 | 0.010001 | CLSTN3; RBP5; RP11-273B20.1 (margin: C1RL-AS1; RNU6-485P; CLSTN3; RBP5; RP11-273B20.1) | 7.95 | 1.86e-05 | 7.98e-04 |
|  | 11 | 1471410 | 1471699 | PDFSTATSENSEMBLUCSC | 290 | 6 | 6420 | gene;island | 0.145169 | 0.968587 | BRSK2 (margin: BRSK2) | 7.94 | 2.16e-03 | 1.45e-02 |
|  | 5 | 128796079 | 128796266 | PDFSTATSENSEMBLUCSC | 188 | 5 | 33074 | island | 0.567654 | 0.010943 | ADAMTS19; ADAMTS19-AS1 (margin: ADAMTS19; ADAMTS19-AS1) | 7.93 | 7.94e-03 | 3.19e-02 |
|  | 3 | 187456175 | 187456579 | PDFSTATSENSEMBLUCSC | 405 | 5 | 28078 | gene;island | 0.156983 | 0.988083 | BCL6 (margin: RP11-211G3.3; RP11-211G3.2; BCL6) | 7.92 | 7.94e-03 | 3.19e-02 |
|  | 1 | 171810105 | 171810570 | PDFSTATSENSEMBLUCSC | 466 | 8 | 2093 | tss;island | 0.039977 | 0.833515 | DNM3 (margin: DNM3) | 7.90 | 1.55e-04 | 2.71e-03 |
|  | 7 | 138794401 | 138794752 | PDFSTATSENSEMBLUCSC | 352 | 9 | 38949 | island | 0.005235 | 0.674601 | ZC3HAV1 (margin: ZC3HAV1) | 7.90 | 4.11e-05 | 1.17e-03 |
|  | 3 | 48700269 | 48700498 | PDFSTATSENSEMBLUCSC | 230 | 8 | 29095 | island | 0.422085 | 0.005861 | RP11-148G20.1; CELSR3; NCKIPSD (margin: RP11-148G20.1; RP11-572O6.1; CELSR3; NCKIPSD) | 7.90 | 1.55e-04 | 2.71e-03 |
|  | 2 | 219857793 | 219858271 | PDFSTATSENSEMBLUCSC | 479 | 9 | 25100 | island | 0.253318 | 0.974547 | CRYBA2 (margin: AC097468.4; FEV; CRYBA2; MIR375; CCDC108) | 7.90 | 8.23e-05 | 2.06e-03 |
|  | 16 | 81526954 | 81527504 | PDFSTATSENSEMBLUCSC | 551 | 7 | 14854 | gene | 0.016507 | 0.868232 | CMIP (margin: CMIP) | 7.89 | 5.83e-04 | 6.47e-03 |
|  | 13 | 99135543 | 99135804 | PDFSTATSENSEMBLUCSC | 262 | 6 | 11201 | gene | 0.116801 | 0.932018 | STK24 (margin: STK24) | 7.88 | 2.16e-03 | 1.45e-02 |
|  | 1 | 179560700 | 179561111 | PDFSTATSENSEMBLUCSC | 412 | 6 | 4000 | island | 0.631001 | 0.009640 | RP11-545A16.4; TDRD5 (margin: RP11-545A16.3; RP11-545A16.4; TDRD5) | 7.87 | 2.16e-03 | 1.45e-02 |
|  | 8 | 22926785 | 22926911 | PDFSTATSENSEMBLUCSC | 127 | 7 | 39595 | tss | 0.015330 | 0.496727 | RP11-875O11.2; TNFRSF10B; RP11-875O11.3 (margin: RP11-875O11.2; TNFRSF10B; RP11-875O11.3) | 7.87 | 1.17e-03 | 1.13e-02 |
|  | 17 | 6899522 | 6899888 | PDFSTATSENSEMBLUCSC | 367 | 5 | 16380 | gene | 0.240198 | 0.977947 | ALOX12; AC027763.2; RP11-589P10.7; RP11-589P10.5 (margin: ALOX12; AC027763.2; RP11-589P10.7; RP11-589P10.5) | 7.86 | 7.94e-03 | 3.19e-02 |
|  | 5 | 57878188 | 57878526 | PDFSTATSENSEMBLUCSC | 339 | 7 | 32077 | tss;island | 0.785558 | 0.012728 | RAB3C (margin: RAB3C) | 7.85 | 5.83e-04 | 6.47e-03 |
|  | 3 | 46448753 | 46449100 | PDFSTATSENSEMBLUCSC | 348 | 5 | 27748 | gene | 0.021420 | 0.861580 | ACKR5; RP11-24F11.2 (margin: ACKR5; RP11-24F11.2) | 7.85 | 7.94e-03 | 3.19e-02 |
|  | 13 | 78272372 | 78272443 | PDFSTATSENSEMBLUCSC | 72 | 6 | 11486 | tss | 0.656605 | 0.007721 | SLAIN1; MIR3665 (margin: SLAIN1; MIR3665) | 7.84 | 2.16e-03 | 1.45e-02 |
|  | 11 | 120008025 | 120008607 | PDFSTATSENSEMBLUCSC | 583 | 6 | 6943 | gene;island | 0.062986 | 0.928554 | TRIM29 (margin: TRIM29) | 7.83 | 2.16e-03 | 1.45e-02 |
|  | 10 | 77163316 | 77163401 | PDFSTATSENSEMBLUCSC | 86 | 6 | 4643 | gene;tss;island | 0.009462 | 0.568389 | ZNF503-AS2; ZNF503 (margin: ZNF503-AS2; RP11-399K21.11; ZNF503) | 7.82 | 2.16e-03 | 1.45e-02 |
|  | 2 | 228029433 | 228029856 | PDFSTATSENSEMBLUCSC | 424 | 8 | 23058 | gene;tss;island | 0.750292 | 0.014867 | COL4A3; COL4A4 (margin: COL4A3; COL4A4) | 7.81 | 1.55e-04 | 2.71e-03 |
|  | 6 | 31275551 | 31275881 | PDFSTATSENSEMBLUCSC | 331 | 11 | 35762 | island | 0.689049 | 0.014808 | XXbac-BPG248L24.10 (margin: XXbac-BPG248L24.13; XXbac-BPG248L24.10) | 7.81 | 2.84e-06 | 1.96e-04 |
|  | 22 | 30901532 | 30901886 | PDFSTATSENSEMBLUCSC | 355 | 7 | 27413 | island | 0.052521 | 0.916292 | SEC14L4 (margin: SEC14L4) | 7.80 | 5.83e-04 | 6.47e-03 |
|  | 13 | 37006063 | 37006340 | PDFSTATSENSEMBLUCSC | 278 | 7 | 11132 | gene;tss;island | 0.785718 | 0.019720 | CCNA1 (margin: CCNA1) | 7.80 | 5.83e-04 | 6.47e-03 |
|  | 1 | 61542546 | 61542689 | PDFSTATSENSEMBLUCSC | 144 | 5 | 1661 | tss;island | 0.675588 | 0.009963 | NFIA (margin: NFIA; AC096534.1) | 7.79 | 7.94e-03 | 3.19e-02 |
|  | 17 | 30592997 | 30593034 | PDFSTATSENSEMBLUCSC | 38 | 5 | 17253 | tss;island | 0.657925 | 0.008674 | RHBDL3 (margin: RHBDL3) | 7.78 | 7.94e-03 | 3.19e-02 |
|  | 1 | 248100183 | 248100614 | PDFSTATSENSEMBLUCSC | 432 | 10 | 4422 | island | 0.757900 | 0.018502 | OR2L13; OR2X1P; RP11-438H8.8 (margin: OR2AJ1; OR2L13; OR2X1P; OR2L8; RP11-438H8.8) | 7.78 | 7.58e-05 | 1.98e-03 |
|  | 12 | 15373987 | 15374609 | PDFSTATSENSEMBLUCSC | 623 | 9 | 10268 | island | 0.748996 | 0.012201 | RERG (margin: RERG) | 7.78 | 4.11e-05 | 1.17e-03 |
|  | 6 | 46138725 | 46139019 | PDFSTATSENSEMBLUCSC | 295 | 10 | 35019 | tss | 0.532603 | 0.007367 | ENPP5 (margin: ENPP5) | 7.77 | 1.08e-05 | 4.82e-04 |
|  | 5 | 131347439 | 131347773 | PDFSTATSENSEMBLUCSC | 335 | 5 | 32266 | tss | 0.630365 | 0.020212 | AC034228.2; ACSL6 (margin: AC034228.3; AC034228.2; ACSL6) | 7.76 | 7.94e-03 | 3.19e-02 |
|  | 1 | 215256254 | 215256978 | PDFSTATSENSEMBLUCSC | 725 | 9 | 1009 | gene;island | 0.688374 | 0.019739 | KCNK2 (margin: KCNK2) | 7.76 | 7.82e-04 | 8.17e-03 |
|  | 2 | 214148856 | 214149035 | PDFSTATSENSEMBLUCSC | 180 | 7 | 23859 | tss | 0.240302 | 0.976442 | SPAG16; AC079610.2 (margin: SPAG16; RP11-105N14.2; AC079610.2) | 7.75 | 5.83e-04 | 6.47e-03 |
|  | 17 | 14204310 | 14204593 | PDFSTATSENSEMBLUCSC | 284 | 6 | 18034 | island | 0.510526 | 0.005011 | HS3ST3B1 (margin: HS3ST3B1; RP11-214O1.2; RP11-214O1.1) | 7.75 | 2.16e-03 | 1.45e-02 |
|  | 14 | 91580129 | 91580613 | PDFSTATSENSEMBLUCSC | 485 | 9 | 12558 | tss | 0.034577 | 0.905840 | C14orf159 (margin: C14orf159) | 7.74 | 4.11e-05 | 1.17e-03 |
|  | 4 | 176986621 | 176987577 | PDFSTATSENSEMBLUCSC | 957 | 14 | 31440 | island | 0.612494 | 0.008049 | WDR17 (margin: WDR17) | 7.74 | 3.49e-07 | 4.42e-05 |
|  | 6 | 152128258 | 152129036 | PDFSTATSENSEMBLUCSC | 779 | 20 | 34514 | gene;island | 0.487199 | 0.007051 | ESR1 (margin: ESR1; RP3-443C4.2) | 7.73 | 5.80e-11 | 4.35e-08 |
|  | 6 | 152128258 | 152128805 | PDFSTATSENSEMBLUCSC | 548 | 18 | 35305 | tss | 0.487199 | 0.008777 | ESR1 (margin: ESR1; RP3-443C4.2) | 7.73 | 8.82e-10 | 3.90e-07 |
|  | 4 | 8582101 | 8582287 | PDFSTATSENSEMBLUCSC | 187 | 6 | 30298 | tss | 0.652664 | 0.011809 | GPR78 (margin: GPR78) | 7.73 | 2.16e-03 | 1.45e-02 |
|  | 1 | 23751369 | 23751761 | PDFSTATSENSEMBLUCSC | 393 | 5 | 1341 | tss | 0.885921 | 0.027362 | TCEA3 (margin: TCEA3; ASAP3) | 7.73 | 7.94e-03 | 3.19e-02 |
|  | 2 | 242212422 | 242212528 | PDFSTATSENSEMBLUCSC | 107 | 5 | 23133 | gene;tss;island | 0.037830 | 0.925862 | HDLBP (margin: HDLBP) | 7.73 | 7.94e-03 | 3.19e-02 |
|  | 7 | 28996196 | 28996639 | PDFSTATSENSEMBLUCSC | 444 | 5 | 36947 | gene;island | 0.643632 | 0.018173 | AC005013.5; AC005013.1; TRIL (margin: AC005013.5; AC005013.1; TRIL) | 7.73 | 7.94e-03 | 3.19e-02 |
|  | 19 | 18118666 | 18119069 | PDFSTATSENSEMBLUCSC | 404 | 6 | 19926 | gene;island | 0.171320 | 0.979980 | ARRDC2 (margin: CTB-52I2.8; ARRDC2; CTB-52I2.5) | 7.73 | 2.16e-03 | 1.45e-02 |
|  | 10 | 116391467 | 116391843 | PDFSTATSENSEMBLUCSC | 377 | 5 | 4769 | gene;island | 0.141263 | 0.972966 | ABLIM1 (margin: ABLIM1) | 7.72 | 7.94e-03 | 3.19e-02 |
|  | 4 | 101111147 | 101111927 | PDFSTATSENSEMBLUCSC | 781 | 9 | 31203 | island | 0.742314 | 0.017194 | RP11-15B17.1; RP11-588P8.1; DDIT4L (margin: RP11-15B17.1; RP11-588P8.1; DDIT4L) | 7.72 | 5.64e-03 | 3.18e-02 |
|  | 3 | 50604613 | 50605156 | PDFSTATSENSEMBLUCSC | 544 | 6 | 27812 | gene | 0.051019 | 0.977475 | HEMK1; C3orf18 (margin: HEMK1; C3orf18) | 7.72 | 8.66e-03 | 3.36e-02 |
|  | 12 | 108733253 | 108733370 | PDFSTATSENSEMBLUCSC | 118 | 6 | 9980 | tss | 0.077269 | 0.925810 | CMKLR1 (margin: CMKLR1) | 7.71 | 2.16e-03 | 1.45e-02 |
|  | 19 | 50831762 | 50832037 | PDFSTATSENSEMBLUCSC | 276 | 5 | 20173 | gene;island | 0.941142 | 0.053825 | NR1H2; KCNC3 (margin: NR1H2; KCNC3; NAPSB) | 7.71 | 7.94e-03 | 3.19e-02 |
|  | 11 | 119993722 | 119994008 | PDFSTATSENSEMBLUCSC | 287 | 7 | 6942 | gene | 0.052219 | 0.943752 | TRIM29 (margin: TRIM29) | 7.71 | 5.83e-04 | 6.47e-03 |
|  | 1 | 40420503 | 40421002 | PDFSTATSENSEMBLUCSC | 500 | 13 | 3111 | island | 0.016041 | 0.536977 | MFSD2A (margin: MFSD2A; RP3-342P20.2; Y\_RNA) | 7.70 | 2.28e-04 | 3.75e-03 |
|  | 1 | 40420503 | 40420692 | PDFSTATSENSEMBLUCSC | 190 | 9 | 1531 | tss | 0.006049 | 0.543333 | MFSD2A (margin: MFSD2A; RP3-342P20.2; Y\_RNA) | 7.70 | 7.77e-03 | 3.19e-02 |
|  | 20 | 37230326 | 37230484 | PDFSTATSENSEMBLUCSC | 159 | 5 | 25737 | tss | 0.032872 | 0.874856 | ARHGAP40 (margin: ARHGAP40) | 7.69 | 7.94e-03 | 3.19e-02 |
|  | 1 | 50489240 | 50489596 | PDFSTATSENSEMBLUCSC | 357 | 7 | 522 | gene | 0.891673 | 0.032619 | AGBL4 (margin: MTND2P29; AGBL4) | 7.69 | 4.08e-03 | 2.47e-02 |
|  | 21 | 43786663 | 43786997 | PDFSTATSENSEMBLUCSC | 335 | 5 | 26567 | tss | 0.672652 | 0.016623 | TFF1 (margin: TFF1; TMPRSS3) | 7.68 | 7.94e-03 | 3.19e-02 |
|  | 7 | 5111488 | 5111669 | PDFSTATSENSEMBLUCSC | 182 | 8 | 37420 | tss;island | 0.204464 | 0.974606 | RBAK (margin: RBAK) | 7.68 | 1.55e-04 | 2.71e-03 |
|  | 14 | 37051693 | 37051949 | PDFSTATSENSEMBLUCSC | 257 | 7 | 12787 | island | 0.006821 | 0.558471 | NKX2-8 (margin: RPL29P3; NKX2-8; RN7SKP257) | 7.68 | 5.83e-04 | 6.47e-03 |
|  | 6 | 30653167 | 30653799 | PDFSTATSENSEMBLUCSC | 633 | 12 | 33804 | gene;island | 0.045732 | 0.899116 | PPP1R18 (margin: PPP1R18; NRM; RPL7P4) | 7.67 | 7.40e-07 | 7.21e-05 |
|  | 7 | 75932840 | 75932977 | PDFSTATSENSEMBLUCSC | 138 | 6 | 37053 | gene;island | 0.397926 | 0.003831 | HSPB1 (margin: HSPB1) | 7.64 | 2.16e-03 | 1.45e-02 |
|  | 12 | 121975121 | 121975669 | PDFSTATSENSEMBLUCSC | 549 | 5 | 9369 | gene;island | 0.824954 | 0.008835 | KDM2B (margin: KDM2B) | 7.64 | 7.94e-03 | 3.19e-02 |
|  | 7 | 87563462 | 87563649 | PDFSTATSENSEMBLUCSC | 188 | 6 | 37689 | tss | 0.490881 | 0.005335 | ADAM22 (margin: ADAM22) | 7.64 | 2.16e-03 | 1.45e-02 |
|  | 7 | 38468944 | 38469273 | PDFSTATSENSEMBLUCSC | 330 | 5 | 36971 | gene | 0.018347 | 0.851583 | AMPH (margin: AMPH) | 7.63 | 7.94e-03 | 3.19e-02 |
|  | 8 | 110704026 | 110704289 | PDFSTATSENSEMBLUCSC | 264 | 5 | 39874 | tss | 0.641600 | 0.010868 | SYBU (margin: SYBU) | 7.63 | 7.94e-03 | 3.19e-02 |
|  | 20 | 1206682 | 1206758 | PDFSTATSENSEMBLUCSC | 77 | 6 | 25535 | tss | 0.163230 | 0.003784 | RAD21L1 (margin: RAD21L1) | 7.62 | 2.16e-03 | 1.45e-02 |
|  | 14 | 23305548 | 23305957 | PDFSTATSENSEMBLUCSC | 410 | 6 | 12703 | island | 0.041398 | 0.890281 | MRPL52; MMP14 (margin: MRPL52; MMP14; SLC7A7) | 7.62 | 2.16e-03 | 1.45e-02 |
|  | 17 | 38465281 | 38465422 | PDFSTATSENSEMBLUCSC | 142 | 5 | 17329 | tss | 0.559284 | 0.005766 | RARA (margin: CDC6; CTD-2267D19.6; RARA) | 7.61 | 7.94e-03 | 3.19e-02 |
|  | 19 | 19281140 | 19281559 | PDFSTATSENSEMBLUCSC | 420 | 9 | 20636 | tss | 0.518657 | 0.005905 | MEF2BNB-MEF2B; MEF2B (margin: MEF2BNB-MEF2B; MEF2B; MEF2BNB) | 7.61 | 2.76e-03 | 1.78e-02 |
|  | 2 | 74730507 | 74730948 | PDFSTATSENSEMBLUCSC | 442 | 9 | 23461 | tss | 0.024516 | 0.584881 | RP11-523H20.3; LBX2-AS1; LBX2; PCGF1 (margin: TTC31; RP11-523H20.3; LBX2-AS1; TLX2; LBX2; AC005041.17; PCGF1) | 7.59 | 4.11e-05 | 1.17e-03 |
|  | 3 | 191048308 | 191048439 | PDFSTATSENSEMBLUCSC | 132 | 5 | 28084 | gene;island | 0.001429 | 0.324409 | CCDC50; UTS2B (margin: CCDC50; UTS2B) | 7.59 | 7.94e-03 | 3.19e-02 |
|  | 4 | 44728525 | 44728857 | PDFSTATSENSEMBLUCSC | 333 | 9 | 31018 | island | 0.005683 | 0.527410 | GNPDA2 (margin: GNPDA2) | 7.58 | 4.11e-05 | 1.17e-03 |
|  | 19 | 54372324 | 54373043 | PDFSTATSENSEMBLUCSC | 720 | 13 | 20209 | gene;island | 0.636065 | 0.006806 | MYADM; AC008753.6 (margin: AC008440.10; MYADM; PRKCG; AC008753.6; AC008440.5) | 7.57 | 1.92e-07 | 2.60e-05 |
|  | 19 | 54372324 | 54372713 | PDFSTATSENSEMBLUCSC | 390 | 9 | 21043 | tss | 0.617231 | 0.006806 | MYADM; AC008753.6 (margin: AC008440.10; MYADM; PRKCG; AC008753.6; AC008440.5) | 7.57 | 4.11e-05 | 1.17e-03 |
|  | 4 | 96470053 | 96470349 | PDFSTATSENSEMBLUCSC | 297 | 7 | 31191 | island | 0.640816 | 0.007889 | RP11-710C12.1; UNC5C (margin: RP11-710C12.1; UNC5C) | 7.57 | 5.83e-04 | 6.47e-03 |
|  | 4 | 96470237 | 96470349 | PDFSTATSENSEMBLUCSC | 113 | 5 | 30505 | tss | 0.792742 | 0.007889 | RP11-710C12.1; UNC5C (margin: RP11-710C12.1; UNC5C) | 7.57 | 7.94e-03 | 3.19e-02 |
|  | 17 | 21279561 | 21279696 | PDFSTATSENSEMBLUCSC | 136 | 5 | 17203 | tss | 0.455491 | 0.985082 | KCNJ12 (margin: KCNJ12) | 7.56 | 7.94e-03 | 3.19e-02 |
|  | 17 | 7197878 | 7198087 | PDFSTATSENSEMBLUCSC | 210 | 7 | 17072 | tss | 0.683375 | 0.004111 | YBX2 (margin: SLC2A4; RP1-4G17.2; YBX2) | 7.56 | 2.33e-03 | 1.52e-02 |
|  | 8 | 22560922 | 22561238 | PDFSTATSENSEMBLUCSC | 317 | 5 | 40142 | island | 0.002856 | 0.375158 | RP11-459E5.1 (margin: RP11-459E5.1; EGR3; PEBP4) | 7.56 | 7.94e-03 | 3.19e-02 |
|  | 6 | 106958303 | 106958645 | PDFSTATSENSEMBLUCSC | 343 | 5 | 35150 | tss;island | 0.873249 | 0.013001 | AIM1 (margin: AIM1) | 7.55 | 7.94e-03 | 3.19e-02 |
|  | 6 | 3848634 | 3849577 | PDFSTATSENSEMBLUCSC | 944 | 26 | 34602 | tss | 0.007421 | 0.498059 | RP11-420L9.4; FAM50B (margin: RP11-420L9.4; FAM50B) | 7.55 | 4.03e-15 | 1.31e-11 |
|  | 7 | 43797839 | 43798207 | PDFSTATSENSEMBLUCSC | 369 | 7 | 37558 | tss;island | 0.563564 | 0.008304 | BLVRA (margin: BLVRA; AC005189.6) | 7.55 | 4.08e-03 | 2.47e-02 |
|  | 15 | 93199018 | 93199180 | PDFSTATSENSEMBLUCSC | 163 | 6 | 14473 | island | 0.439713 | 0.008437 | FAM174B (margin: FAM174B) | 7.54 | 2.16e-03 | 1.45e-02 |
|  | 6 | 26189333 | 26189597 | PDFSTATSENSEMBLUCSC | 265 | 6 | 34692 | tss | 0.002518 | 0.259360 | HIST1H4D (margin: HIST1H2BE; HIST1H1PS1; HIST1H2BF; HIST1H4D; HIST1H3D; HIST1H2AD) | 7.54 | 2.16e-03 | 1.45e-02 |
|  | 19 | 36132263 | 36132627 | PDFSTATSENSEMBLUCSC | 365 | 6 | 20710 | tss;island | 0.212847 | 0.943455 | ETV2 (margin: RBM42; ETV2; COX6B1) | 7.54 | 8.66e-03 | 3.36e-02 |
|  | 18 | 20714217 | 20714496 | PDFSTATSENSEMBLUCSC | 280 | 6 | 19203 | tss;island | 0.494748 | 0.005530 | CABLES1; AC105247.1 (margin: CABLES1; AC105247.1) | 7.54 | 2.16e-03 | 1.45e-02 |
|  | 1 | 179560700 | 179560983 | PDFSTATSENSEMBLUCSC | 284 | 5 | 2119 | tss | 0.545865 | 0.011234 | RP11-545A16.4; TDRD5 (margin: RP11-545A16.3; RP11-545A16.4; TDRD5) | 7.53 | 7.94e-03 | 3.19e-02 |
|  | 11 | 119020099 | 119020168 | PDFSTATSENSEMBLUCSC | 70 | 6 | 6937 | gene;tss;island | 0.245700 | 0.005085 | ABCG4 (margin: ABCG4; AP002956.1) | 7.53 | 2.16e-03 | 1.45e-02 |
|  | 11 | 118401536 | 118401878 | PDFSTATSENSEMBLUCSC | 343 | 5 | 8858 | island | 0.410006 | 0.978223 | TTC36; TMEM25; RP11-770J1.3 (margin: KMT2A; TTC36; TMEM25; RP11-770J1.3) | 7.52 | 7.94e-03 | 3.19e-02 |
|  | 10 | 52833902 | 52834189 | PDFSTATSENSEMBLUCSC | 288 | 7 | 4590 | gene;tss;island | 0.777780 | 0.013817 | PRKG1 (margin: PRKG1; RP11-96B5.3) | 7.51 | 5.83e-04 | 6.47e-03 |
|  | 5 | 67583972 | 67584380 | PDFSTATSENSEMBLUCSC | 409 | 8 | 31637 | gene;island | 0.637094 | 0.009804 | PIK3R1 (margin: PIK3R1) | 7.51 | 1.55e-04 | 2.71e-03 |
|  | 5 | 67583972 | 67584222 | PDFSTATSENSEMBLUCSC | 251 | 6 | 32103 | tss | 0.637094 | 0.009804 | PIK3R1 (margin: PIK3R1) | 7.51 | 2.16e-03 | 1.45e-02 |
|  | 7 | 122526824 | 122526940 | PDFSTATSENSEMBLUCSC | 117 | 6 | 37867 | tss | 0.479181 | 0.007492 | CADPS2 (margin: CADPS2) | 7.50 | 2.16e-03 | 1.45e-02 |
|  | 6 | 29893926 | 29894341 | PDFSTATSENSEMBLUCSC | 416 | 16 | 34757 | tss;island | 0.060563 | 0.909336 | HLA-K; HCG4B (margin: HLA-K; HLA-U; HCG4B) | 7.50 | 6.65e-09 | 2.09e-06 |
|  | 18 | 70211774 | 70211974 | PDFSTATSENSEMBLUCSC | 201 | 6 | 19318 | tss;island | 0.667853 | 0.014394 | CBLN2 (margin: CBLN2) | 7.50 | 2.16e-03 | 1.45e-02 |
|  | 11 | 98891492 | 98891820 | PDFSTATSENSEMBLUCSC | 329 | 9 | 7640 | tss;island | 0.807820 | 0.019823 | CNTN5 (margin: CNTN5) | 7.50 | 4.11e-05 | 1.17e-03 |
|  | 11 | 63439115 | 63439313 | PDFSTATSENSEMBLUCSC | 199 | 7 | 7377 | tss | 0.805798 | 0.015898 | ATL3 (margin: RTN3; ATL3) | 7.50 | 5.83e-04 | 6.47e-03 |
|  | 3 | 158390510 | 158390821 | PDFSTATSENSEMBLUCSC | 312 | 7 | 28686 | tss | 0.967124 | 0.030208 | GFM1; LXN (margin: GFM1; LXN) | 7.49 | 5.83e-04 | 6.47e-03 |
|  | 3 | 10206254 | 10206731 | PDFSTATSENSEMBLUCSC | 478 | 10 | 28874 | island | 0.011123 | 0.629617 | IRAK2 (margin: IRAK2) | 7.49 | 1.08e-05 | 4.82e-04 |
|  | 3 | 73674074 | 73674702 | PDFSTATSENSEMBLUCSC | 629 | 12 | 28446 | tss | 0.687794 | 0.010838 | PDZRN3-AS1; PDZRN3 (margin: PDZRN3-AS1; PDZRN3) | 7.48 | 7.40e-07 | 7.21e-05 |
|  | 6 | 46097392 | 46097784 | PDFSTATSENSEMBLUCSC | 393 | 9 | 36184 | island | 0.555134 | 0.004956 | ENPP4; RP1-8B1.4 (margin: ENPP4; RP1-8B1.4) | 7.48 | 4.11e-05 | 1.17e-03 |
|  | 5 | 156886816 | 156887129 | PDFSTATSENSEMBLUCSC | 314 | 7 | 33342 | island | 0.151132 | 0.933745 | NIPAL4; CTB-109A12.1; ADAM19 (margin: NIPAL4; CTB-109A12.1; ADAM19) | 7.47 | 5.83e-04 | 6.47e-03 |
|  | 6 | 46097392 | 46097695 | PDFSTATSENSEMBLUCSC | 304 | 8 | 35018 | tss | 0.547611 | 0.005192 | ENPP4; RP1-8B1.4 (margin: ENPP4; RP1-8B1.4) | 7.47 | 1.55e-04 | 2.71e-03 |
|  | 5 | 140345461 | 140346403 | PDFSTATSENSEMBLUCSC | 943 | 14 | 31806 | gene;island | 0.654195 | 0.017370 | PCDHA1; PCDHA2; PCDHA3; PCDHA4; PCDHA5; PCDHA6; PCDHA7; PCDHA8; PCDHA9; PCDHA10; PCDHA11; PCDHA12; PCDHA13; PCDHAC1; PCDHAC2 (margin: PCDHA1; PCDHA2; PCDHA3; PCDHA4; PCDHA5; PCDHA6; PCDHA7; PCDHA8; PCDHA9; PCDHA10; PCDHA11; PCDHA12; PCDHA13; PCDHAC1; PCDHAC2) | 7.47 | 6.74e-03 | 3.19e-02 |
|  | 13 | 92050675 | 92051154 | PDFSTATSENSEMBLUCSC | 480 | 11 | 11814 | island | 0.586370 | 0.007782 | GPC5 (margin: GPC5) | 7.47 | 5.67e-06 | 3.54e-04 |
|  | 12 | 98850691 | 98851021 | PDFSTATSENSEMBLUCSC | 331 | 6 | 10703 | island | 0.260487 | 0.976988 | SLC9A7P1 (margin: SLC9A7P1) | 7.46 | 2.16e-03 | 1.45e-02 |
|  | 4 | 144480206 | 144480561 | PDFSTATSENSEMBLUCSC | 356 | 11 | 30614 | tss | 0.978698 | 0.244793 | SMARCA5; GUSBP5 (margin: SMARCA5; GUSBP5; RP11-481K16.2) | 7.45 | 2.84e-06 | 1.96e-04 |
|  | 4 | 172734266 | 172734514 | PDFSTATSENSEMBLUCSC | 249 | 6 | 30678 | tss;island | 0.712600 | 0.012023 | GALNTL6 (margin: GALNTL6) | 7.44 | 4.33e-03 | 2.52e-02 |
|  | 11 | 121970496 | 121970768 | PDFSTATSENSEMBLUCSC | 273 | 6 | 6946 | gene | 0.971004 | 0.179433 | RP11-166D19.1; MIR125B1 (margin: RP11-166D19.1; MIR125B1) | 7.43 | 2.16e-03 | 1.45e-02 |
|  | 11 | 125366238 | 125366590 | PDFSTATSENSEMBLUCSC | 353 | 9 | 7791 | tss;island | 0.277824 | 0.979808 | AP000708.1; FEZ1 (margin: AP000708.1; FEZ1) | 7.43 | 1.06e-02 | 4.01e-02 |
|  | 3 | 46448496 | 46448963 | PDFSTATSENSEMBLUCSC | 468 | 8 | 28274 | tss | 0.040052 | 0.864856 | ACKR5; RP11-24F11.2 (margin: ACKR5; RP11-24F11.2) | 7.43 | 1.55e-04 | 2.71e-03 |
|  | 10 | 63422511 | 63422841 | PDFSTATSENSEMBLUCSC | 331 | 8 | 5769 | island | 0.692062 | 0.021229 | C10orf107 (margin: C10orf107) | 7.43 | 1.55e-04 | 2.71e-03 |
|  | 1 | 197881327 | 197881540 | PDFSTATSENSEMBLUCSC | 214 | 5 | 2157 | tss;island | 0.035328 | 0.956617 | LHX9 (margin: C1orf53; LHX9) | 7.41 | 7.94e-03 | 3.19e-02 |
|  | 7 | 8473234 | 8473578 | PDFSTATSENSEMBLUCSC | 345 | 6 | 37439 | tss;island | 0.778704 | 0.025441 | NXPH1 (margin: NXPH1) | 7.41 | 2.16e-03 | 1.45e-02 |
|  | 15 | 78423938 | 78424152 | PDFSTATSENSEMBLUCSC | 215 | 6 | 13829 | tss | 0.522790 | 0.016524 | IDH3A; CIB2 (margin: IDH3A; RP11-285A1.1; CIB2) | 7.39 | 2.16e-03 | 1.45e-02 |
|  | 7 | 193229 | 193732 | PDFSTATSENSEMBLUCSC | 504 | 5 | 36701 | gene;island | 0.017854 | 0.682162 | FAM20C; AC093627.12 (margin: FAM20C; AC093627.12) | 7.39 | 7.94e-03 | 3.19e-02 |
|  | 10 | 135340445 | 135340850 | PDFSTATSENSEMBLUCSC | 406 | 7 | 5465 | tss | 0.041826 | 0.892464 | CYP2E1; SPRN; AL161645.2 (margin: RP11-108K14.4; CYP2E1; SPRN; AL161645.2) | 7.39 | 5.83e-04 | 6.47e-03 |
|  | 4 | 55991782 | 55991943 | PDFSTATSENSEMBLUCSC | 162 | 6 | 30402 | tss | 0.643531 | 0.024479 | KDR (margin: KDR) | 7.39 | 2.16e-03 | 1.45e-02 |
|  | 21 | 40984760 | 40984886 | PDFSTATSENSEMBLUCSC | 127 | 5 | 26552 | tss | 0.835622 | 0.021726 | B3GALT5; C21orf88 (margin: B3GALT5; C21orf88) | 7.39 | 7.94e-03 | 3.19e-02 |
|  | 5 | 42424356 | 42424604 | PDFSTATSENSEMBLUCSC | 249 | 5 | 31601 | gene;island | 0.707137 | 0.009842 | GHR (margin: GHR) | 7.38 | 7.94e-03 | 3.19e-02 |
|  | 2 | 214148856 | 214149200 | PDFSTATSENSEMBLUCSC | 345 | 9 | 25064 | island | 0.252636 | 0.968467 | SPAG16; AC079610.2 (margin: SPAG16; RP11-105N14.2; AC079610.2) | 7.38 | 4.11e-05 | 1.17e-03 |
|  | 11 | 24518149 | 24518448 | PDFSTATSENSEMBLUCSC | 300 | 9 | 7205 | tss;island | 0.678467 | 0.031119 | LUZP2 (margin: LUZP2) | 7.37 | 4.11e-05 | 1.17e-03 |
|  | 2 | 217560272 | 217560403 | PDFSTATSENSEMBLUCSC | 132 | 5 | 23871 | tss;island | 0.563550 | 0.009585 | AC007563.5; IGFBP5 (margin: AC007563.5; IGFBP5) | 7.37 | 7.94e-03 | 3.19e-02 |
|  | 5 | 35617687 | 35618055 | PDFSTATSENSEMBLUCSC | 369 | 10 | 32728 | island | 0.507961 | 0.006058 | SPEF2 (margin: SPEF2) | 7.36 | 1.50e-03 | 1.41e-02 |
|  | 5 | 35617687 | 35617969 | PDFSTATSENSEMBLUCSC | 283 | 8 | 32015 | tss | 0.510631 | 0.006665 | SPEF2 (margin: SPEF2) | 7.36 | 1.04e-02 | 3.96e-02 |
|  | 12 | 100750473 | 100750811 | PDFSTATSENSEMBLUCSC | 339 | 6 | 9941 | tss | 0.114190 | 0.933134 | SLC17A8 (margin: SLC17A8) | 7.36 | 2.16e-03 | 1.45e-02 |
|  | 10 | 134901199 | 134901305 | PDFSTATSENSEMBLUCSC | 107 | 7 | 5452 | tss | 0.867132 | 0.042019 | GPR123; RP13-439H18.4; AL445199.1 (margin: GPR123; RP13-439H18.4; AL445199.1) | 7.35 | 5.83e-04 | 6.47e-03 |
|  | 5 | 115910467 | 115910732 | PDFSTATSENSEMBLUCSC | 266 | 5 | 33032 | island | 0.436631 | 0.006489 | CTB-118N6.2; SEMA6A (margin: CTB-118N6.1; CTB-118N6.2; SEMA6A) | 7.35 | 7.94e-03 | 3.19e-02 |
|  | 8 | 121824330 | 121824929 | PDFSTATSENSEMBLUCSC | 600 | 9 | 39891 | tss | 0.726566 | 0.007639 | RP11-713M15.2; SNTB1 (margin: RP11-713M15.2; SNTB1) | 7.34 | 3.99e-03 | 2.47e-02 |
|  | 11 | 121970613 | 121970768 | PDFSTATSENSEMBLUCSC | 156 | 5 | 7769 | tss | 0.986994 | 0.305700 | RP11-166D19.1; MIR125B1 (margin: RP11-166D19.1; MIR125B1) | 7.33 | 7.94e-03 | 3.19e-02 |
|  | 5 | 134824531 | 134824947 | PDFSTATSENSEMBLUCSC | 417 | 6 | 33136 | island | 0.516441 | 0.015087 | (margin: ) | 7.33 | 2.16e-03 | 1.45e-02 |
|  | 8 | 70747067 | 70747646 | PDFSTATSENSEMBLUCSC | 580 | 7 | 40372 | island | 0.394780 | 0.004073 | RP11-159H10.3; SLCO5A1 (margin: RP11-159H10.3; SLCO5A1) | 7.32 | 1.17e-03 | 1.13e-02 |
|  | 4 | 94749865 | 94750061 | PDFSTATSENSEMBLUCSC | 197 | 6 | 30501 | tss | 0.560383 | 0.008257 | ATOH1 (margin: RNA5SP164; ATOH1) | 7.31 | 2.16e-03 | 1.45e-02 |
|  | 11 | 92931250 | 92931416 | PDFSTATSENSEMBLUCSC | 167 | 6 | 7618 | tss;island | 0.395396 | 0.006289 | SLC36A4 (margin: SLC36A4) | 7.31 | 2.16e-03 | 1.45e-02 |
|  | 10 | 94833520 | 94833632 | PDFSTATSENSEMBLUCSC | 113 | 6 | 5235 | tss | 0.467561 | 0.006944 | CYP26A1 (margin: CYP26C1; CYP26A1) | 7.31 | 2.16e-03 | 1.45e-02 |
|  | 10 | 63422511 | 63422668 | PDFSTATSENSEMBLUCSC | 158 | 7 | 5098 | tss | 0.667549 | 0.026702 | C10orf107 (margin: C10orf107) | 7.30 | 5.83e-04 | 6.47e-03 |
|  | 10 | 73533035 | 73533561 | PDFSTATSENSEMBLUCSC | 527 | 10 | 4624 | gene;island | 0.006436 | 0.547136 | CDH23; C10orf54 (margin: CDH23; C10orf54) | 7.30 | 1.08e-05 | 4.82e-04 |
|  | 17 | 46681111 | 46681401 | PDFSTATSENSEMBLUCSC | 291 | 6 | 16656 | gene | 0.003590 | 0.628672 | HOXB-AS3; HOXB3; HOXB6 (margin: HOXB-AS3; HOXB3; HOXB5; HOXB6; HOXB7; HOXB8) | 7.30 | 4.33e-03 | 2.52e-02 |
|  | 7 | 120497998 | 120498369 | PDFSTATSENSEMBLUCSC | 372 | 5 | 38863 | island | 0.434233 | 0.006446 | TSPAN12 (margin: TSPAN12) | 7.29 | 7.94e-03 | 3.19e-02 |
|  | 7 | 95025611 | 95026248 | PDFSTATSENSEMBLUCSC | 638 | 20 | 38678 | island | 0.162573 | 0.971275 | PON1; PON3 (margin: PON1; PON3; PON2) | 7.29 | 1.45e-11 | 1.57e-08 |
|  | 2 | 12856626 | 12857067 | PDFSTATSENSEMBLUCSC | 442 | 10 | 24085 | island | 0.186230 | 0.003452 | RP11-333O1.1; TRIB2 (margin: RP11-333O1.1; TRIB2) | 7.28 | 1.08e-05 | 4.82e-04 |
|  | 1 | 220921216 | 220921670 | PDFSTATSENSEMBLUCSC | 455 | 8 | 2262 | tss | 0.546819 | 0.009086 | MARC2 (margin: MARC2) | 7.28 | 1.55e-04 | 2.71e-03 |
|  | 3 | 182970675 | 182971069 | PDFSTATSENSEMBLUCSC | 395 | 11 | 28049 | gene;island | 0.006621 | 0.382088 | B3GNT5; MCF2L2 (margin: B3GNT5; MCF2L2; RNA5SP151) | 7.28 | 1.28e-02 | 4.76e-02 |
|  | 2 | 1497815 | 1498138 | PDFSTATSENSEMBLUCSC | 324 | 5 | 22531 | gene;island | 0.113098 | 0.958536 | TPO (margin: TPO) | 7.27 | 7.94e-03 | 3.19e-02 |
|  | 6 | 29429909 | 29430506 | PDFSTATSENSEMBLUCSC | 598 | 6 | 33658 | gene | 0.003507 | 0.470864 | OR2H1; UBDP1 (margin: OR2H1; OR11A1; UBDP1) | 7.27 | 2.16e-03 | 1.45e-02 |
|  | 4 | 55991683 | 55991943 | PDFSTATSENSEMBLUCSC | 261 | 8 | 31057 | island | 0.720908 | 0.024979 | KDR (margin: KDR) | 7.27 | 1.55e-04 | 2.71e-03 |
|  | 11 | 133938676 | 133939064 | PDFSTATSENSEMBLUCSC | 389 | 6 | 8974 | island | 0.218973 | 0.979688 | JAM3 (margin: JAM3) | 7.27 | 2.16e-03 | 1.45e-02 |
|  | 14 | 103989208 | 103989789 | PDFSTATSENSEMBLUCSC | 582 | 9 | 12649 | tss;island | 0.074624 | 0.979670 | CKB; RP11-600F24.7 (margin: TRMT61A; CKB; RP11-600F24.7) | 7.26 | 4.11e-05 | 1.17e-03 |
|  | 8 | 17434749 | 17435088 | PDFSTATSENSEMBLUCSC | 340 | 5 | 39217 | gene;island | 0.432513 | 0.005639 | PDGFRL (margin: SLC7A2; PDGFRL) | 7.26 | 7.94e-03 | 3.19e-02 |
|  | 8 | 142517341 | 142517553 | PDFSTATSENSEMBLUCSC | 213 | 5 | 39930 | tss | 0.027156 | 0.882560 | MROH5 (margin: AC138647.1; MROH5) | 7.25 | 7.94e-03 | 3.19e-02 |
|  | 10 | 75670653 | 75670903 | PDFSTATSENSEMBLUCSC | 251 | 8 | 4638 | gene;island | 0.181772 | 0.971353 | PLAU; C10orf55 (margin: PLAU; C10orf55) | 7.25 | 1.55e-04 | 2.71e-03 |
|  | 10 | 44880542 | 44880819 | PDFSTATSENSEMBLUCSC | 278 | 7 | 5055 | tss;island | 0.814968 | 0.028098 | AL137026.1; CXCL12 (margin: AL137026.1; CXCL12) | 7.25 | 5.83e-04 | 6.47e-03 |
|  | 19 | 12175107 | 12175540 | PDFSTATSENSEMBLUCSC | 434 | 7 | 20488 | tss | 0.174627 | 0.933873 | ZNF844 (margin: ZNF844; RNA5SP466; CTD-2006C1.10; ZNF878) | 7.25 | 5.83e-04 | 6.47e-03 |
|  | 4 | 13546111 | 13546673 | PDFSTATSENSEMBLUCSC | 563 | 10 | 30933 | island | 0.676668 | 0.018335 | NKX3-2; AC006445.8 (margin: NKX3-2; AC006445.8) | 7.24 | 3.25e-04 | 4.91e-03 |
|  | 11 | 8832817 | 8833095 | PDFSTATSENSEMBLUCSC | 279 | 6 | 6500 | gene;tss | 0.111682 | 0.946756 | RP11-318C2.1; ST5 (margin: RP11-318C2.1; ST5) | 7.24 | 2.16e-03 | 1.45e-02 |
|  | 21 | 45622096 | 45622493 | PDFSTATSENSEMBLUCSC | 398 | 5 | 26731 | island | 0.921074 | 0.072315 | AP001057.1 (margin: AP001057.1; AP001058.3) | 7.24 | 7.94e-03 | 3.19e-02 |
|  | 11 | 6440690 | 6440968 | PDFSTATSENSEMBLUCSC | 279 | 10 | 7110 | tss;island | 0.578195 | 0.018045 | APBB1 (margin: APBB1; HPX) | 7.22 | 3.25e-04 | 4.91e-03 |
|  | 10 | 134901199 | 134901689 | PDFSTATSENSEMBLUCSC | 491 | 9 | 6332 | island | 0.866980 | 0.042019 | GPR123; RP13-439H18.4; AL445199.1 (margin: GPR123; RP13-439H18.4; AL445199.1) | 7.22 | 4.11e-05 | 1.17e-03 |
|  | 19 | 20162642 | 20163138 | PDFSTATSENSEMBLUCSC | 497 | 7 | 21833 | island | 0.311244 | 0.003944 | (margin: AC006539.4) | 7.21 | 5.83e-04 | 6.47e-03 |
|  | 12 | 99548329 | 99548550 | PDFSTATSENSEMBLUCSC | 222 | 5 | 9262 | gene | 0.118350 | 0.931670 | ANKS1B (margin: ANKS1B) | 7.21 | 7.94e-03 | 3.19e-02 |
|  | 13 | 43566114 | 43566642 | PDFSTATSENSEMBLUCSC | 529 | 12 | 11698 | island | 0.127893 | 0.967059 | EPSTI1 (margin: EPSTI1) | 7.21 | 7.40e-07 | 7.21e-05 |
|  | 11 | 6440456 | 6440482 | PDFSTATSENSEMBLUCSC | 27 | 5 | 7109 | tss | 0.589523 | 0.011723 | APBB1 (margin: APBB1; HPX) | 7.21 | 7.94e-03 | 3.19e-02 |
|  | 7 | 98099806 | 98100419 | PDFSTATSENSEMBLUCSC | 614 | 7 | 38713 | island | 0.885376 | 0.031724 | (margin: AC074121.4) | 7.20 | 4.08e-03 | 2.47e-02 |
|  | 1 | 121260494 | 121261146 | PDFSTATSENSEMBLUCSC | 653 | 7 | 3668 | island | 0.028271 | 0.732992 | EMBP1 (margin: EMBP1; RP11-344P13.4) | 7.19 | 4.08e-03 | 2.47e-02 |
|  | 4 | 77172617 | 77172841 | PDFSTATSENSEMBLUCSC | 225 | 6 | 30447 | tss | 0.104825 | 0.002015 | FAM47E; FAM47E-STBD1 (margin: FAM47E; FAM47E-STBD1) | 7.19 | 4.33e-03 | 2.52e-02 |
|  | 4 | 144480206 | 144480740 | PDFSTATSENSEMBLUCSC | 535 | 12 | 31337 | island | 0.969658 | 0.244241 | SMARCA5; GUSBP5 (margin: SMARCA5; GUSBP5; RP11-481K16.2) | 7.18 | 7.40e-07 | 7.21e-05 |
|  | 5 | 112073348 | 112073769 | PDFSTATSENSEMBLUCSC | 422 | 15 | 31713 | gene | 0.006453 | 0.436353 | APC (margin: APC) | 7.18 | 1.29e-08 | 3.49e-06 |
|  | 5 | 112073348 | 112073544 | PDFSTATSENSEMBLUCSC | 197 | 11 | 32214 | tss | 0.006453 | 0.439442 | APC (margin: APC) | 7.18 | 2.84e-06 | 1.96e-04 |
|  | 7 | 95025736 | 95026248 | PDFSTATSENSEMBLUCSC | 513 | 19 | 37726 | tss | 0.169020 | 0.970806 | PON1; PON3 (margin: PON1; PON3; PON2) | 7.17 | 5.66e-11 | 4.35e-08 |
|  | 10 | 6622279 | 6622566 | PDFSTATSENSEMBLUCSC | 288 | 8 | 4935 | tss;island | 0.012952 | 0.847074 | PRKCQ-AS1; PRKCQ (margin: PRKCQ-AS1; RP11-5N23.3; PRKCQ) | 7.17 | 6.22e-04 | 6.71e-03 |
|  | 11 | 35441012 | 35441311 | PDFSTATSENSEMBLUCSC | 300 | 6 | 8207 | island | 0.602157 | 0.016611 | RP4-683L5.1; SLC1A2 (margin: RP4-683L5.1; SLC1A2) | 7.17 | 4.33e-03 | 2.52e-02 |
|  | 9 | 841340 | 841636 | PDFSTATSENSEMBLUCSC | 297 | 5 | 40801 | tss;island | 0.183238 | 0.972307 | DMRT1 (margin: DMRT1) | 7.16 | 7.94e-03 | 3.19e-02 |
|  | 7 | 45026259 | 45026583 | PDFSTATSENSEMBLUCSC | 325 | 8 | 37576 | tss;island | 0.009017 | 0.510605 | SNHG15 (margin: MYO1G; SNHG15) | 7.16 | 1.55e-04 | 2.71e-03 |
|  | 12 | 54366760 | 54367008 | PDFSTATSENSEMBLUCSC | 249 | 6 | 10484 | island | 0.004694 | 0.387656 | HOXC11; HOTAIR (margin: HOXC11; HOXC10; HOTAIR; HOXC-AS3) | 7.16 | 2.16e-03 | 1.45e-02 |
|  | 15 | 45421578 | 45422054 | PDFSTATSENSEMBLUCSC | 477 | 8 | 13327 | gene | 0.179751 | 0.938979 | DUOX1; DUOXA1 (margin: DUOXA2; DUOX1; DUOXA1) | 7.16 | 1.55e-04 | 2.71e-03 |
|  | 7 | 50633725 | 50634252 | PDFSTATSENSEMBLUCSC | 528 | 6 | 37595 | tss | 0.050865 | 0.840452 | DDC (margin: DDC) | 7.15 | 2.16e-03 | 1.45e-02 |
|  | 4 | 118006405 | 118006832 | PDFSTATSENSEMBLUCSC | 428 | 9 | 31264 | island | 0.512939 | 0.007954 | TRAM1L1 (margin: TRAM1L1) | 7.14 | 4.11e-05 | 1.17e-03 |
|  | 10 | 94833520 | 94833658 | PDFSTATSENSEMBLUCSC | 139 | 7 | 4680 | gene;island | 0.469482 | 0.007278 | CYP26A1 (margin: CYP26C1; CYP26A1) | 7.14 | 5.83e-04 | 6.47e-03 |
|  | 8 | 12990213 | 12991196 | PDFSTATSENSEMBLUCSC | 984 | 12 | 39212 | gene;island | 0.391183 | 0.005200 | DLC1 (margin: DLC1) | 7.12 | 7.40e-07 | 7.21e-05 |
|  | 4 | 48491941 | 48492344 | PDFSTATSENSEMBLUCSC | 404 | 8 | 31033 | island | 0.141045 | 0.955433 | SLC10A4; ZAR1 (margin: SLC10A4; ZAR1; FRYL) | 7.12 | 1.55e-04 | 2.71e-03 |
|  | 20 | 45523098 | 45523294 | PDFSTATSENSEMBLUCSC | 197 | 5 | 26222 | island | 0.312438 | 0.003545 | EYA2 (margin: EYA2; RP11-323C15.2) | 7.12 | 7.94e-03 | 3.19e-02 |
|  | 8 | 80803196 | 80803526 | PDFSTATSENSEMBLUCSC | 331 | 5 | 40410 | island | 0.291396 | 0.004024 | (margin: ) | 7.11 | 7.94e-03 | 3.19e-02 |
|  | 8 | 63998640 | 63998778 | PDFSTATSENSEMBLUCSC | 139 | 6 | 39731 | tss | 0.526332 | 0.009706 | TTPA (margin: TTPA) | 7.11 | 2.16e-03 | 1.45e-02 |
|  | 6 | 33172817 | 33173581 | PDFSTATSENSEMBLUCSC | 765 | 17 | 34228 | gene;island | 0.007682 | 0.387922 | SLC39A7; HSD17B8 (margin: RNY4P10; SLC39A7; HSD17B8; MIR219-1; RING1; ZNF70P1; RXRB) | 7.11 | 1.71e-09 | 6.42e-07 |
|  | 11 | 26353484 | 26353811 | PDFSTATSENSEMBLUCSC | 328 | 8 | 8140 | island | 0.616941 | 0.020281 | ANO3 (margin: ANO3) | 7.11 | 1.55e-04 | 2.71e-03 |
|  | 7 | 1491787 | 1492018 | PDFSTATSENSEMBLUCSC | 232 | 5 | 36753 | gene;island | 0.149291 | 0.969127 | MICALL2 (margin: AC102953.4; MICALL2) | 7.11 | 7.94e-03 | 3.19e-02 |
|  | 16 | 58059105 | 58059154 | PDFSTATSENSEMBLUCSC | 50 | 5 | 15269 | tss | 0.179183 | 0.003014 | MMP15 (margin: USB1; MMP15) | 7.11 | 7.94e-03 | 3.19e-02 |
|  | 5 | 87971794 | 87972265 | PDFSTATSENSEMBLUCSC | 472 | 7 | 31679 | gene;island | 0.459419 | 0.008097 | CTC-467M3.1; LINC00461 (margin: CTC-467M3.1; LINC00461) | 7.10 | 5.83e-04 | 6.47e-03 |
|  | 10 | 97666894 | 97667244 | PDFSTATSENSEMBLUCSC | 351 | 6 | 5250 | tss;island | 0.474981 | 0.006395 | RP11-248J23.7; C10orf131; ENTPD1-AS1 (margin: RP11-248J23.7; C10orf131; ENTPD1-AS1) | 7.10 | 2.16e-03 | 1.45e-02 |
|  | 12 | 51818350 | 51818768 | PDFSTATSENSEMBLUCSC | 419 | 7 | 10427 | island | 0.466417 | 0.008697 | SLC4A8; RP11-607P23.1 (margin: SLC4A8; RP11-607P23.1) | 7.08 | 1.17e-03 | 1.13e-02 |
|  | 3 | 187457128 | 187457732 | PDFSTATSENSEMBLUCSC | 605 | 7 | 28079 | gene;island | 0.087885 | 0.867960 | BCL6 (margin: RP11-211G3.3; RP11-211G3.2; BCL6) | 7.07 | 5.83e-04 | 6.47e-03 |
|  | 12 | 116996773 | 116997095 | PDFSTATSENSEMBLUCSC | 323 | 8 | 10038 | tss | 0.161729 | 0.969115 | MAP1LC3B2 (margin: RP11-809C9.2; MAP1LC3B2) | 7.06 | 1.09e-03 | 1.10e-02 |
|  | 4 | 164264796 | 164265012 | PDFSTATSENSEMBLUCSC | 217 | 7 | 30661 | tss;island | 0.680610 | 0.022560 | NPY5R; NPY1R (margin: NPY5R; NPY1R) | 7.05 | 5.83e-04 | 6.47e-03 |
|  | 4 | 48491941 | 48492304 | PDFSTATSENSEMBLUCSC | 364 | 7 | 30383 | tss | 0.172506 | 0.946471 | SLC10A4; ZAR1 (margin: SLC10A4; ZAR1; FRYL) | 7.04 | 5.83e-04 | 6.47e-03 |
|  | 19 | 37288291 | 37288793 | PDFSTATSENSEMBLUCSC | 503 | 6 | 21946 | island | 0.673186 | 0.018297 | CTD-2162K18.5 (margin: CTD-2162K18.5) | 7.04 | 2.16e-03 | 1.45e-02 |
|  | 4 | 6202379 | 6202609 | PDFSTATSENSEMBLUCSC | 231 | 6 | 30278 | tss;island | 0.623207 | 0.013714 | RP11-586D19.1; RP11-586D19.2; JAKMIP1 (margin: RP11-586D19.1; RP11-586D19.2; JAKMIP1) | 7.04 | 2.16e-03 | 1.45e-02 |
|  | 5 | 156886816 | 156887005 | PDFSTATSENSEMBLUCSC | 190 | 6 | 32456 | tss | 0.163736 | 0.951439 | NIPAL4; CTB-109A12.1; ADAM19 (margin: NIPAL4; CTB-109A12.1; ADAM19) | 7.04 | 2.16e-03 | 1.45e-02 |
|  | 11 | 120195717 | 120195905 | PDFSTATSENSEMBLUCSC | 189 | 5 | 7762 | tss;island | 0.852304 | 0.044329 | TMEM136 (margin: POU2F3; TMEM136; ARHGEF12) | 7.04 | 7.94e-03 | 3.19e-02 |
|  | 19 | 50004152 | 50004625 | PDFSTATSENSEMBLUCSC | 474 | 6 | 20952 | tss | 0.281142 | 0.936057 | RPS11; hsa-mir-150; MIR150; CTD-3148I10.13 (margin: RPL13A; SNORD32A; SNORD33; SNORD34; SNORD35A; RPS11; SNORD35B; hsa-mir-150; FCGRT; MIR150; CTD-3148I10.13) | 7.03 | 2.16e-03 | 1.45e-02 |
|  | 2 | 210636350 | 210636560 | PDFSTATSENSEMBLUCSC | 211 | 5 | 23852 | tss | 0.825957 | 0.022004 | UNC80 (margin: UNC80) | 7.03 | 7.94e-03 | 3.19e-02 |
|  | 1 | 220921216 | 220922217 | PDFSTATSENSEMBLUCSC | 1002 | 14 | 4219 | island | 0.534819 | 0.010064 | MARC2 (margin: MARC2) | 7.01 | 4.99e-08 | 9.34e-06 |
|  | 6 | 33538979 | 33539394 | PDFSTATSENSEMBLUCSC | 416 | 5 | 36041 | island | 0.869745 | 0.066346 | BAK1 (margin: BAK1) | 7.01 | 7.94e-03 | 3.19e-02 |
|  | 22 | 45405621 | 45405919 | PDFSTATSENSEMBLUCSC | 299 | 9 | 27216 | tss;island | 0.487334 | 0.009462 | PHF21B (margin: PHF21B; RP4-753M9.1) | 7.00 | 2.88e-04 | 4.58e-03 |
|  | 17 | 8869099 | 8869285 | PDFSTATSENSEMBLUCSC | 187 | 6 | 17124 | tss;island | 0.777673 | 0.023190 | CTB-41I6.1; CTB-41I6.2; PIK3R5 (margin: CTB-41I6.1; CTB-41I6.2; PIK3R5) | 7.00 | 2.16e-03 | 1.45e-02 |
|  | 5 | 140261653 | 140261810 | PDFSTATSENSEMBLUCSC | 158 | 6 | 32356 | tss | 0.981093 | 0.294043 | PCDHA1; PCDHA2; PCDHA3; PCDHA4; PCDHA5; PCDHA6; PCDHA7; PCDHA8; PCDHA9; PCDHA10; PCDHA11; PCDHA12; PCDHA13 (margin: PCDHA1; PCDHA2; PCDHA3; PCDHA4; PCDHA5; PCDHA6; PCDHA7; PCDHA8; PCDHA9; PCDHA10; PCDHA11; PCDHA12; PCDHA13) | 7.00 | 8.66e-03 | 3.36e-02 |
|  | 17 | 38599271 | 38599763 | PDFSTATSENSEMBLUCSC | 493 | 6 | 18306 | island | 0.827302 | 0.022852 | IGFBP4 (margin: IGFBP4; RP11-58O9.1) | 6.99 | 2.16e-03 | 1.45e-02 |
|  | 3 | 186649165 | 186649855 | PDFSTATSENSEMBLUCSC | 691 | 6 | 28076 | gene;island | 0.637317 | 0.014181 | ST6GAL1; AC007690.1 (margin: ST6GAL1; AC007690.1; RP11-42D20.1) | 6.98 | 2.16e-03 | 1.45e-02 |
|  | 10 | 73533407 | 73533561 | PDFSTATSENSEMBLUCSC | 155 | 7 | 5145 | tss | 0.008946 | 0.573310 | CDH23; C10orf54 (margin: CDH23; C10orf54) | 6.98 | 5.83e-04 | 6.47e-03 |
|  | 5 | 154091976 | 154092426 | PDFSTATSENSEMBLUCSC | 451 | 6 | 32450 | tss | 0.630488 | 0.012511 | LARP1 (margin: LARP1; RN7SL439P; Metazoa\_SRP) | 6.97 | 2.16e-03 | 1.45e-02 |
|  | 1 | 29101849 | 29102153 | PDFSTATSENSEMBLUCSC | 305 | 5 | 2952 | island | 0.194740 | 0.973939 | (margin: YTHDF2) | 6.96 | 7.94e-03 | 3.19e-02 |
|  | 4 | 94749865 | 94750205 | PDFSTATSENSEMBLUCSC | 341 | 7 | 31186 | island | 0.541368 | 0.009574 | ATOH1 (margin: RNA5SP164; ATOH1) | 6.96 | 5.83e-04 | 6.47e-03 |
|  | 8 | 67940823 | 67941178 | PDFSTATSENSEMBLUCSC | 356 | 7 | 39749 | tss | 0.624074 | 0.011857 | PPP1R42 (margin: PPP1R42) | 6.96 | 5.83e-04 | 6.47e-03 |
|  | 6 | 80657412 | 80657555 | PDFSTATSENSEMBLUCSC | 144 | 7 | 35083 | tss;island | 0.579808 | 0.011203 | ELOVL4 (margin: ELOVL4; GAPDHP63) | 6.95 | 5.83e-04 | 6.47e-03 |
|  | 2 | 70995349 | 70995607 | PDFSTATSENSEMBLUCSC | 259 | 8 | 23420 | tss;island | 0.359977 | 0.985026 | ADD2 (margin: ADD2; FIGLA) | 6.95 | 1.55e-04 | 2.71e-03 |
|  | 4 | 5712581 | 5713100 | PDFSTATSENSEMBLUCSC | 520 | 9 | 30880 | island | 0.022459 | 0.670293 | EVC; EVC2 (margin: EVC; EVC2) | 6.94 | 8.23e-05 | 2.06e-03 |
|  | 3 | 44902933 | 44903330 | PDFSTATSENSEMBLUCSC | 398 | 8 | 28264 | tss | 0.020158 | 0.510301 | KIF15; TMEM42; MIR564 (margin: KIF15; TMEM42; MIR564) | 6.93 | 1.55e-04 | 2.71e-03 |
|  | 5 | 150399781 | 150400672 | PDFSTATSENSEMBLUCSC | 892 | 9 | 33315 | island | 0.956282 | 0.106810 | GPX3 (margin: GPX3; TNIP1) | 6.92 | 1.85e-03 | 1.45e-02 |
|  | 5 | 1008828 | 1009204 | PDFSTATSENSEMBLUCSC | 377 | 5 | 32600 | island | 0.411998 | 0.010622 | NKD2 (margin: AC116351.2; RP11-43F13.4; NKD2; RP11-43F13.3) | 6.91 | 7.94e-03 | 3.19e-02 |
|  | 1 | 183154981 | 183155154 | PDFSTATSENSEMBLUCSC | 174 | 5 | 2134 | tss | 0.092016 | 0.915549 | LAMC2 (margin: LAMC2) | 6.91 | 7.94e-03 | 3.19e-02 |
|  | 10 | 133793442 | 133793734 | PDFSTATSENSEMBLUCSC | 293 | 5 | 4847 | gene;island | 0.982381 | 0.224085 | BNIP3 (margin: BNIP3) | 6.91 | 7.94e-03 | 3.19e-02 |
|  | 17 | 9929528 | 9929867 | PDFSTATSENSEMBLUCSC | 340 | 5 | 16440 | gene | 0.015714 | 0.685017 | GAS7 (margin: GAS7) | 6.90 | 7.94e-03 | 3.19e-02 |
|  | 19 | 12175107 | 12175719 | PDFSTATSENSEMBLUCSC | 613 | 10 | 21591 | island | 0.293095 | 0.937219 | ZNF844 (margin: ZNF844; RNA5SP466; CTD-2006C1.10; ZNF878) | 6.89 | 1.08e-05 | 4.82e-04 |
|  | 3 | 73673893 | 73674702 | PDFSTATSENSEMBLUCSC | 810 | 15 | 29286 | island | 0.642287 | 0.010218 | PDZRN3-AS1; PDZRN3 (margin: PDZRN3-AS1; PDZRN3) | 6.89 | 1.29e-08 | 3.49e-06 |
|  | 10 | 86001241 | 86001560 | PDFSTATSENSEMBLUCSC | 320 | 7 | 5194 | tss | 0.024339 | 0.916705 | LRIT1 (margin: RGR; LRIT1) | 6.89 | 4.08e-03 | 2.47e-02 |
|  | 7 | 24796981 | 24797884 | PDFSTATSENSEMBLUCSC | 904 | 12 | 38318 | island | 0.245013 | 0.965274 | DFNA5 (margin: DFNA5) | 6.88 | 3.64e-03 | 2.29e-02 |
|  | 11 | 62477052 | 62477624 | PDFSTATSENSEMBLUCSC | 573 | 9 | 7361 | tss | 0.410492 | 0.988075 | GNG3; BSCL2; RP11-831H9.16 (margin: GNG3; BSCL2; RP11-831H9.16; HNRNPUL2) | 6.88 | 4.11e-05 | 1.17e-03 |
|  | 16 | 30572739 | 30573013 | PDFSTATSENSEMBLUCSC | 275 | 5 | 15836 | island | 0.445333 | 0.989192 | (margin: AC002310.7; AC002310.13; ZNF764; ZNF688) | 6.88 | 7.94e-03 | 3.19e-02 |
|  | 6 | 166419479 | 166419943 | PDFSTATSENSEMBLUCSC | 465 | 6 | 36625 | island | 0.304983 | 0.004398 | (margin: ) | 6.88 | 4.33e-03 | 2.52e-02 |
|  | 6 | 150346721 | 150347053 | PDFSTATSENSEMBLUCSC | 333 | 10 | 35294 | tss | 0.014398 | 0.685007 | RAET1L (margin: RAET1M; RAET1L) | 6.87 | 2.17e-05 | 8.94e-04 |
|  | 6 | 26188969 | 26189597 | PDFSTATSENSEMBLUCSC | 629 | 10 | 35522 | island | 0.003306 | 0.279103 | HIST1H4D (margin: HIST1H2BE; HIST1H1PS1; HIST1H2BF; HIST1H4D; HIST1H3D; HIST1H2AD) | 6.87 | 1.08e-05 | 4.82e-04 |
|  | 14 | 75535757 | 75536561 | PDFSTATSENSEMBLUCSC | 805 | 9 | 13012 | island | 0.008778 | 0.498084 | ZC2HC1C; ACYP1 (margin: ZC2HC1C; ACYP1) | 6.87 | 4.11e-05 | 1.17e-03 |
|  | 22 | 37640314 | 37640514 | PDFSTATSENSEMBLUCSC | 201 | 5 | 27115 | tss | 0.025419 | 0.747675 | RAC2 (margin: RAC2) | 6.87 | 7.94e-03 | 3.19e-02 |
|  | 1 | 247275326 | 247275542 | PDFSTATSENSEMBLUCSC | 217 | 5 | 1124 | gene | 0.571079 | 0.009801 | C1orf229 (margin: FGFR3P6; ZNF669; C1orf229; ZNF124) | 6.87 | 7.94e-03 | 3.19e-02 |
|  | 5 | 174151043 | 174151567 | PDFSTATSENSEMBLUCSC | 525 | 7 | 32505 | tss | 0.355032 | 0.003599 | MSX2 (margin: MSX2) | 6.86 | 1.17e-03 | 1.13e-02 |
|  | 16 | 66638210 | 66638702 | PDFSTATSENSEMBLUCSC | 493 | 10 | 14782 | gene;island | 0.731925 | 0.009440 | CMTM3 (margin: CMTM3; CMTM4) | 6.86 | 1.05e-03 | 1.08e-02 |
|  | 12 | 66583029 | 66583431 | PDFSTATSENSEMBLUCSC | 403 | 5 | 9207 | gene;island | 0.249094 | 0.984925 | IRAK3 (margin: IRAK3) | 6.86 | 7.94e-03 | 3.19e-02 |
|  | 4 | 57976431 | 57976944 | PDFSTATSENSEMBLUCSC | 514 | 9 | 31076 | island | 0.013286 | 0.637867 | RP11-12A1.1; IGFBP7 (margin: RP11-12A1.1; IGFBP7; snoU13) | 6.85 | 4.11e-05 | 1.17e-03 |
|  | 10 | 75670653 | 75670793 | PDFSTATSENSEMBLUCSC | 141 | 7 | 5166 | tss | 0.208011 | 0.960350 | PLAU; C10orf55 (margin: PLAU; C10orf55) | 6.85 | 5.83e-04 | 6.47e-03 |
|  | 4 | 93226245 | 93226625 | PDFSTATSENSEMBLUCSC | 381 | 5 | 30061 | gene;island | 0.863815 | 0.058550 | GRID2; RP11-9B6.1 (margin: GRID2; RP11-9B6.1) | 6.84 | 7.94e-03 | 3.19e-02 |
|  | 3 | 132378605 | 132379275 | PDFSTATSENSEMBLUCSC | 671 | 9 | 27950 | gene;tss;island | 0.005477 | 0.401725 | UBA5; ACAD11; NPHP3 (margin: HSPA8P19; UBA5; ACAD11; NPHP3) | 6.84 | 4.11e-05 | 1.17e-03 |
|  | 7 | 87563462 | 87563931 | PDFSTATSENSEMBLUCSC | 470 | 11 | 38639 | island | 0.494824 | 0.010785 | ADAM22 (margin: ADAM22) | 6.84 | 5.67e-06 | 3.54e-04 |
|  | 12 | 89746101 | 89746771 | PDFSTATSENSEMBLUCSC | 671 | 10 | 10664 | island | 0.002736 | 0.231467 | DUSP6 (margin: DUSP6) | 6.84 | 1.08e-05 | 4.82e-04 |
|  | 20 | 36793705 | 36794002 | PDFSTATSENSEMBLUCSC | 298 | 10 | 25731 | tss | 0.638531 | 0.011227 | TGM2 (margin: TGM2) | 6.84 | 1.08e-05 | 4.82e-04 |
|  | 15 | 91497863 | 91498497 | PDFSTATSENSEMBLUCSC | 635 | 10 | 14464 | island | 0.762567 | 0.026799 | UNC45A; RCCD1; AC068831.6 (margin: UNC45A; RCCD1; PRC1-AS1; AC068831.6; PRC1) | 6.83 | 1.08e-05 | 4.82e-04 |
|  | 1 | 155035008 | 155035530 | PDFSTATSENSEMBLUCSC | 523 | 5 | 1978 | tss;island | 0.921286 | 0.055797 | ADAM15; EFNA4; EFNA3 (margin: DCST1; ADAM15; EFNA4; EFNA3) | 6.83 | 7.94e-03 | 3.19e-02 |
|  | 11 | 62476891 | 62477624 | PDFSTATSENSEMBLUCSC | 734 | 10 | 8355 | island | 0.388633 | 0.986732 | GNG3; BSCL2; RP11-831H9.16 (margin: GNG3; BSCL2; RP11-831H9.16; HNRNPUL2) | 6.83 | 4.33e-05 | 1.22e-03 |
|  | 3 | 181444368 | 181445156 | PDFSTATSENSEMBLUCSC | 789 | 9 | 28045 | gene;island | 0.169176 | 0.971031 | SOX2-OT (margin: SOX2-OT) | 6.83 | 8.23e-05 | 2.06e-03 |
|  | 13 | 20805380 | 20805679 | PDFSTATSENSEMBLUCSC | 300 | 8 | 11330 | tss | 0.152697 | 0.927213 | GJB6 (margin: GJB6) | 6.83 | 1.55e-04 | 2.71e-03 |
|  | 11 | 128761146 | 128761211 | PDFSTATSENSEMBLUCSC | 66 | 6 | 7807 | tss;island | 0.023015 | 0.640883 | KCNJ5 (margin: KCNJ5; RP11-740D6.3; C11orf45) | 6.82 | 2.16e-03 | 1.45e-02 |
|  | 7 | 56160717 | 56161020 | PDFSTATSENSEMBLUCSC | 304 | 7 | 37609 | tss | 0.714170 | 0.015311 | PHKG1 (margin: snoU13; PHKG1; CHCHD2) | 6.81 | 5.83e-04 | 6.47e-03 |
|  | 6 | 34433676 | 34433789 | PDFSTATSENSEMBLUCSC | 114 | 7 | 34919 | tss;island | 0.698268 | 0.008613 | PACSIN1 (margin: PACSIN1; BX255972.1) | 6.80 | 5.83e-04 | 6.47e-03 |
|  | 6 | 29855110 | 29855636 | PDFSTATSENSEMBLUCSC | 527 | 16 | 35639 | island | 0.040919 | 0.661666 | HLA-H; HCG4P7 (margin: HLA-H; HLA-T; HCG4P7) | 6.80 | 2.23e-07 | 2.90e-05 |
|  | 5 | 43020161 | 43020716 | PDFSTATSENSEMBLUCSC | 556 | 7 | 32765 | island | 0.004369 | 0.423874 | CTD-2035E11.3; CTD-2201E18.3 (margin: CTD-2035E11.3; CTD-2201E18.3) | 6.80 | 5.83e-04 | 6.47e-03 |
|  | 13 | 111522755 | 111522985 | PDFSTATSENSEMBLUCSC | 231 | 5 | 11540 | tss | 0.005884 | 0.468077 | LINC00346 (margin: LINC00346; ANKRD10) | 6.80 | 7.94e-03 | 3.19e-02 |
|  | 17 | 39968362 | 39968871 | PDFSTATSENSEMBLUCSC | 510 | 14 | 17345 | tss;island | 0.509380 | 0.007789 | FKBP10; LEPREL4 (margin: FKBP10; LEPREL4) | 6.79 | 1.99e-07 | 2.63e-05 |
|  | 1 | 150208519 | 150208726 | PDFSTATSENSEMBLUCSC | 208 | 8 | 1903 | tss;island | 0.004783 | 0.316037 | RNU2-17P; ANP32E (margin: RNU2-17P; ANP32E) | 6.79 | 1.55e-04 | 2.71e-03 |
|  | 8 | 8820844 | 8821258 | PDFSTATSENSEMBLUCSC | 415 | 5 | 40059 | island | 0.620522 | 0.018267 | RP11-62H7.2; SNORA70 (margin: RP11-62H7.2; SNORA70) | 6.79 | 7.94e-03 | 3.19e-02 |
|  | 18 | 18822579 | 18823249 | PDFSTATSENSEMBLUCSC | 671 | 7 | 19066 | gene;island | 0.927544 | 0.116218 | GREB1L; RP11-699A5.2 (margin: GREB1L; RP11-699A5.2) | 6.79 | 5.83e-04 | 6.47e-03 |
|  | 20 | 25039658 | 25039812 | PDFSTATSENSEMBLUCSC | 155 | 5 | 25644 | tss;island | 0.792545 | 0.125721 | ACSS1 (margin: ACSS1; VSX1) | 6.79 | 7.94e-03 | 3.19e-02 |
|  | 17 | 45056626 | 45056976 | PDFSTATSENSEMBLUCSC | 351 | 8 | 17438 | tss;island | 0.495075 | 0.008322 | GOSR2; RP11-156P1.2; LRRC37A17P; RPRML (margin: GOSR2; RP11-156P1.2; MIR5089; LRRC37A17P; RPRML; RP11-156P1.3) | 6.78 | 6.22e-04 | 6.71e-03 |
|  | 1 | 18691778 | 18692019 | PDFSTATSENSEMBLUCSC | 242 | 5 | 271 | gene;island | 0.053513 | 0.974561 | IGSF21 (margin: IGSF21) | 6.78 | 7.94e-03 | 3.19e-02 |
|  | 12 | 108523395 | 108523472 | PDFSTATSENSEMBLUCSC | 78 | 5 | 9979 | tss | 0.783637 | 0.032147 | WSCD2 (margin: WSCD2) | 6.77 | 7.94e-03 | 3.19e-02 |
|  | 6 | 41620951 | 41621322 | PDFSTATSENSEMBLUCSC | 372 | 6 | 34331 | gene;island | 0.862050 | 0.047630 | MDFI (margin: MDFI) | 6.77 | 2.16e-03 | 1.45e-02 |
|  | 6 | 31126599 | 31127379 | PDFSTATSENSEMBLUCSC | 781 | 21 | 34800 | tss | 0.007658 | 0.682361 | TCF19; CCHCR1 (margin: TCF19; CCHCR1; POU5F1) | 6.77 | 4.34e-08 | 9.34e-06 |
|  | 12 | 5541220 | 5541403 | PDFSTATSENSEMBLUCSC | 184 | 6 | 10182 | island | 0.077432 | 0.870977 | NTF3 (margin: NTF3) | 6.76 | 2.16e-03 | 1.45e-02 |
|  | 10 | 118765099 | 118765335 | PDFSTATSENSEMBLUCSC | 237 | 8 | 5387 | tss;island | 0.584996 | 0.014700 | KIAA1598 (margin: KIAA1598) | 6.76 | 1.55e-04 | 2.71e-03 |
|  | 17 | 40346279 | 40346486 | PDFSTATSENSEMBLUCSC | 208 | 5 | 16578 | gene | 0.069590 | 0.772510 | GHDC (margin: HCRT; GHDC; STAT5B) | 6.76 | 7.94e-03 | 3.19e-02 |
|  | 20 | 32307885 | 32308529 | PDFSTATSENSEMBLUCSC | 645 | 11 | 26102 | island | 0.513200 | 0.006186 | PXMP4 (margin: ZNF341; PXMP4; RP4-553F4.2) | 6.76 | 3.18e-03 | 2.01e-02 |
|  | 7 | 130126153 | 130126637 | PDFSTATSENSEMBLUCSC | 485 | 6 | 37223 | gene | 0.412190 | 0.005814 | MEST; RP11-2E11.5 (margin: MEST; MIR335; RP11-2E11.5; hsa-mir-335) | 6.76 | 8.66e-03 | 3.36e-02 |
|  | 16 | 8806966 | 8807043 | PDFSTATSENSEMBLUCSC | 78 | 5 | 14653 | gene | 0.075806 | 0.896051 | ABAT (margin: ABAT; RNU7-63P) | 6.76 | 7.94e-03 | 3.19e-02 |
|  | 3 | 186857284 | 186857593 | PDFSTATSENSEMBLUCSC | 310 | 7 | 28778 | tss;island | 0.004173 | 0.401838 | RPL39L (margin: RPL39L) | 6.75 | 5.83e-04 | 6.47e-03 |
|  | 1 | 12226913 | 12227131 | PDFSTATSENSEMBLUCSC | 219 | 5 | 2699 | island | 0.306616 | 0.970181 | TNFRSF1B (margin: TNFRSF1B) | 6.75 | 7.94e-03 | 3.19e-02 |
|  | 11 | 122852412 | 122852698 | PDFSTATSENSEMBLUCSC | 287 | 8 | 7775 | tss | 0.917900 | 0.161289 | BSX (margin: BSX) | 6.75 | 1.55e-04 | 2.71e-03 |
|  | 4 | 186435443 | 186435667 | PDFSTATSENSEMBLUCSC | 225 | 6 | 30210 | gene | 0.976491 | 0.284488 | PDLIM3 (margin: PDLIM3) | 6.75 | 2.16e-03 | 1.45e-02 |
|  | 1 | 211306986 | 211307408 | PDFSTATSENSEMBLUCSC | 423 | 5 | 993 | gene;island | 0.426752 | 0.006266 | KCNH1; KCNH1-IT1 (margin: KCNH1; KCNH1-IT1) | 6.75 | 7.94e-03 | 3.19e-02 |
|  | 1 | 210110998 | 210111162 | PDFSTATSENSEMBLUCSC | 165 | 5 | 2235 | tss;island | 0.336794 | 0.009198 | SYT14 (margin: SYT14) | 6.74 | 7.94e-03 | 3.19e-02 |
|  | 1 | 39874802 | 39875069 | PDFSTATSENSEMBLUCSC | 268 | 12 | 1521 | tss | 0.701414 | 0.019579 | MACF1; KIAA0754 (margin: MACF1; KIAA0754) | 6.74 | 2.22e-05 | 9.05e-04 |
|  | 7 | 93519855 | 93520566 | PDFSTATSENSEMBLUCSC | 712 | 19 | 38669 | island | 0.833285 | 0.039170 | GNGT1; AC002076.10; TFPI2 (margin: GNGT1; AC002076.10; TFPI2) | 6.74 | 2.26e-10 | 1.16e-07 |
|  | 6 | 3848634 | 3849818 | PDFSTATSENSEMBLUCSC | 1185 | 31 | 35389 | island | 0.009897 | 0.496856 | RP11-420L9.4; FAM50B (margin: RP11-420L9.4; FAM50B) | 6.74 | 2.03e-11 | 1.98e-08 |
|  | 14 | 75535757 | 75536217 | PDFSTATSENSEMBLUCSC | 461 | 6 | 12515 | tss | 0.010809 | 0.558942 | ZC2HC1C; ACYP1 (margin: ZC2HC1C; ACYP1) | 6.74 | 2.16e-03 | 1.45e-02 |
|  | 10 | 25464008 | 25464259 | PDFSTATSENSEMBLUCSC | 252 | 8 | 4991 | tss | 0.728679 | 0.026978 | GPR158; GPR158-AS1 (margin: GPR158; GPR158-AS1) | 6.73 | 1.55e-04 | 2.71e-03 |
|  | 1 | 92949447 | 92950836 | PDFSTATSENSEMBLUCSC | 1390 | 29 | 1751 | tss | 0.243322 | 0.971923 | GFI1 (margin: GFI1) | 6.73 | 1.05e-08 | 3.21e-06 |
|  | 10 | 135340445 | 135341025 | PDFSTATSENSEMBLUCSC | 581 | 9 | 6367 | island | 0.169381 | 0.892464 | CYP2E1; SPRN; AL161645.2 (margin: RP11-108K14.4; CYP2E1; SPRN; AL161645.2) | 6.73 | 4.11e-05 | 1.17e-03 |
|  | 8 | 1764878 | 1765477 | PDFSTATSENSEMBLUCSC | 600 | 12 | 40038 | island | 0.377028 | 0.985968 | MIR596 (margin: MIR596; ARHGEF10) | 6.72 | 7.40e-07 | 7.21e-05 |
|  | 8 | 1764878 | 1765387 | PDFSTATSENSEMBLUCSC | 510 | 10 | 39535 | tss | 0.377028 | 0.985968 | MIR596 (margin: MIR596; ARHGEF10) | 6.72 | 1.08e-05 | 4.82e-04 |
|  | 15 | 91497863 | 91498080 | PDFSTATSENSEMBLUCSC | 218 | 6 | 13908 | tss | 0.783728 | 0.037068 | UNC45A; RCCD1; AC068831.6 (margin: UNC45A; RCCD1; PRC1-AS1; AC068831.6; PRC1) | 6.72 | 2.16e-03 | 1.45e-02 |
|  | 1 | 244213358 | 244213618 | PDFSTATSENSEMBLUCSC | 261 | 5 | 2354 | tss;island | 0.941729 | 0.170774 | ZBTB18 (margin: RP11-278H7.1; ZBTB18) | 6.72 | 7.94e-03 | 3.19e-02 |
|  | 1 | 156675322 | 156675562 | PDFSTATSENSEMBLUCSC | 241 | 7 | 3863 | island | 0.650819 | 0.020391 | CRABP2 (margin: CRABP2; RP11-66D17.5) | 6.70 | 5.83e-04 | 6.47e-03 |
|  | 19 | 37329090 | 37329859 | PDFSTATSENSEMBLUCSC | 770 | 15 | 21947 | island | 0.593467 | 0.008594 | ZNF790 (margin: CTD-2162K18.5; ZNF345; ZNF790) | 6.70 | 1.29e-08 | 3.49e-06 |
|  | 20 | 44746392 | 44747006 | PDFSTATSENSEMBLUCSC | 615 | 11 | 26215 | island | 0.129910 | 0.934471 | CD40 (margin: CD40) | 6.70 | 2.84e-06 | 1.96e-04 |
|  | 20 | 44746392 | 44746902 | PDFSTATSENSEMBLUCSC | 511 | 9 | 25789 | tss | 0.129910 | 0.934471 | CD40 (margin: CD40) | 6.70 | 4.11e-05 | 1.17e-03 |
|  | 17 | 46671131 | 46671635 | PDFSTATSENSEMBLUCSC | 505 | 9 | 17464 | tss | 0.493760 | 0.013263 | HOXB-AS3; HOXB3; HOXB5; HOXB6 (margin: HOXB-AS3; HOXB3; MIR10A; HOXB5; HOXB6) | 6.69 | 1.23e-03 | 1.18e-02 |
|  | 16 | 21170927 | 21171239 | PDFSTATSENSEMBLUCSC | 313 | 5 | 14688 | gene;tss;island | 0.136289 | 0.002012 | TMEM159; DNAH3 (margin: TMEM159; DNAH3) | 6.68 | 7.94e-03 | 3.19e-02 |
|  | 13 | 113759771 | 113760286 | PDFSTATSENSEMBLUCSC | 516 | 6 | 11956 | island | 0.559401 | 0.019443 | F7 (margin: MCF2L; F7; AL137002.1) | 6.68 | 2.16e-03 | 1.45e-02 |
|  | 16 | 31159558 | 31159920 | PDFSTATSENSEMBLUCSC | 363 | 5 | 14733 | gene;island | 0.783503 | 0.123001 | PRSS36 (margin: PRSS36) | 6.68 | 7.94e-03 | 3.19e-02 |
|  | 13 | 36050844 | 36051073 | PDFSTATSENSEMBLUCSC | 230 | 7 | 11390 | tss | 0.074358 | 0.881816 | NBEA; MAB21L1 (margin: NBEA; MAB21L1) | 6.68 | 5.83e-04 | 6.47e-03 |
|  | 7 | 100769903 | 100770286 | PDFSTATSENSEMBLUCSC | 384 | 5 | 37798 | tss | 0.004803 | 0.401164 | SERPINE1 (margin: SERPINE1) | 6.67 | 7.94e-03 | 3.19e-02 |
|  | 1 | 10056837 | 10057168 | PDFSTATSENSEMBLUCSC | 332 | 7 | 1233 | tss | 0.361523 | 0.982024 | RBP7 (margin: NMNAT1; RBP7) | 6.67 | 5.83e-04 | 6.47e-03 |
|  | 8 | 110986685 | 110987019 | PDFSTATSENSEMBLUCSC | 335 | 5 | 40542 | island | 0.522314 | 0.027212 | RP11-696P8.2; KCNV1 (margin: RP11-696P8.2; KCNV1) | 6.66 | 7.94e-03 | 3.19e-02 |
|  | 17 | 6899085 | 6899888 | PDFSTATSENSEMBLUCSC | 804 | 14 | 17913 | island | 0.305123 | 0.979368 | ALOX12; AC027763.2; RP11-589P10.7; RP11-589P10.5 (margin: ALOX12; AC027763.2; RP11-589P10.7; RP11-589P10.5) | 6.66 | 4.99e-08 | 9.34e-06 |
|  | 1 | 10854205 | 10854560 | PDFSTATSENSEMBLUCSC | 356 | 5 | 217 | gene;island | 0.350352 | 0.006745 | CASZ1 (margin: CASZ1) | 6.66 | 7.94e-03 | 3.19e-02 |
|  | 4 | 48484997 | 48485328 | PDFSTATSENSEMBLUCSC | 332 | 8 | 30382 | tss;island | 0.206271 | 0.977545 | SLC10A4 (margin: SLC10A4; ZAR1) | 6.66 | 2.95e-03 | 1.88e-02 |
|  | 18 | 35145983 | 35146770 | PDFSTATSENSEMBLUCSC | 788 | 21 | 19477 | island | 0.717615 | 0.008448 | CELF4 (margin: CELF4) | 6.65 | 3.40e-09 | 1.10e-06 |
|  | 2 | 74730266 | 74730948 | PDFSTATSENSEMBLUCSC | 683 | 11 | 22745 | gene;island | 0.020739 | 0.557663 | RP11-523H20.3; LBX2-AS1; LBX2; PCGF1 (margin: TTC31; RP11-523H20.3; LBX2-AS1; TLX2; LBX2; AC005041.17; PCGF1) | 6.65 | 2.84e-06 | 1.96e-04 |
|  | 12 | 101603835 | 101604258 | PDFSTATSENSEMBLUCSC | 424 | 8 | 10717 | island | 0.701681 | 0.019187 | SLC5A8 (margin: SLC5A8; RNU6-768P) | 6.65 | 1.55e-04 | 2.71e-03 |
|  | 8 | 49647579 | 49648363 | PDFSTATSENSEMBLUCSC | 785 | 11 | 40284 | island | 0.139527 | 0.971559 | EFCAB1 (margin: EFCAB1) | 6.65 | 2.75e-04 | 4.46e-03 |
|  | 1 | 156675388 | 156675562 | PDFSTATSENSEMBLUCSC | 175 | 6 | 2020 | tss | 0.632415 | 0.021955 | CRABP2 (margin: CRABP2; RP11-66D17.5) | 6.65 | 2.16e-03 | 1.45e-02 |
|  | 19 | 11877661 | 11878036 | PDFSTATSENSEMBLUCSC | 376 | 7 | 21583 | island | 0.012621 | 0.552066 | ZNF441 (margin: ZNF441; CTC-499B15.1) | 6.64 | 5.83e-04 | 6.47e-03 |
|  | 15 | 41805868 | 41806234 | PDFSTATSENSEMBLUCSC | 367 | 5 | 14063 | island | 0.178321 | 0.937749 | LTK (margin: ITPKA; LTK; RPAP1) | 6.64 | 7.94e-03 | 3.19e-02 |
|  | 19 | 37406932 | 37407152 | PDFSTATSENSEMBLUCSC | 221 | 6 | 20005 | gene | 0.482800 | 0.008364 | ZNF568; ZNF829 (margin: ZNF345; ZNF568; ZNF829) | 6.64 | 2.16e-03 | 1.45e-02 |
|  | 19 | 19280969 | 19281559 | PDFSTATSENSEMBLUCSC | 591 | 11 | 19947 | gene;island | 0.518657 | 0.012012 | MEF2BNB-MEF2B; MEF2B (margin: MEF2BNB-MEF2B; MEF2B; MEF2BNB) | 6.64 | 3.94e-04 | 5.86e-03 |
|  | 15 | 63674108 | 63674276 | PDFSTATSENSEMBLUCSC | 169 | 6 | 13722 | tss;island | 0.488175 | 0.019645 | CA12 (margin: RP11-321G12.1; CA12) | 6.64 | 2.16e-03 | 1.45e-02 |
|  | 1 | 13840381 | 13840712 | PDFSTATSENSEMBLUCSC | 332 | 7 | 2703 | island | 0.424862 | 0.968490 | RP4-597A16.2; LRRC38 (margin: RP4-597A16.2; LRRC38) | 6.63 | 5.83e-04 | 6.47e-03 |
|  | 1 | 1141617 | 1141991 | PDFSTATSENSEMBLUCSC | 375 | 5 | 20 | gene | 0.667637 | 0.018728 | TNFRSF18 (margin: TTLL10; TNFRSF18; TNFRSF4; SDF4) | 6.63 | 7.94e-03 | 3.19e-02 |
|  | 14 | 36003074 | 36003211 | PDFSTATSENSEMBLUCSC | 138 | 6 | 12354 | tss | 0.523529 | 0.013804 | INSM2 (margin: INSM2; RALGAPA1) | 6.63 | 2.16e-03 | 1.45e-02 |
|  | 18 | 43652172 | 43652594 | PDFSTATSENSEMBLUCSC | 423 | 10 | 19484 | island | 0.744952 | 0.047956 | PSTPIP2 (margin: PSTPIP2; Y\_RNA; ATP5A1) | 6.62 | 1.08e-05 | 4.82e-04 |
|  | 6 | 151646540 | 151646651 | PDFSTATSENSEMBLUCSC | 112 | 5 | 35299 | tss | 0.515729 | 0.012495 | AKAP12 (margin: AKAP12; RN7SKP268) | 6.62 | 7.94e-03 | 3.19e-02 |
|  | 18 | 43652255 | 43652594 | PDFSTATSENSEMBLUCSC | 340 | 8 | 19251 | tss | 0.897404 | 0.079279 | PSTPIP2 (margin: PSTPIP2; Y\_RNA; ATP5A1) | 6.62 | 1.55e-04 | 2.71e-03 |
|  | 17 | 14204310 | 14204485 | PDFSTATSENSEMBLUCSC | 176 | 5 | 17146 | tss | 0.482895 | 0.005378 | HS3ST3B1 (margin: HS3ST3B1; RP11-214O1.2; RP11-214O1.1) | 6.62 | 7.94e-03 | 3.19e-02 |
|  | 16 | 1146194 | 1146545 | PDFSTATSENSEMBLUCSC | 352 | 5 | 15516 | island | 0.280982 | 0.982434 | C1QTNF8 (margin: C1QTNF8; LA16c-349E11.1) | 6.62 | 7.94e-03 | 3.19e-02 |
|  | 6 | 55444061 | 55444488 | PDFSTATSENSEMBLUCSC | 428 | 7 | 35043 | tss | 0.680507 | 0.052985 | HMGCLL1 (margin: HMGCLL1) | 6.62 | 1.17e-03 | 1.13e-02 |
|  | 7 | 12443478 | 12444290 | PDFSTATSENSEMBLUCSC | 813 | 11 | 38266 | island | 0.705256 | 0.066293 | VWDE (margin: VWDE) | 6.62 | 4.10e-03 | 2.48e-02 |
|  | 19 | 39522418 | 39523626 | PDFSTATSENSEMBLUCSC | 1209 | 11 | 21986 | island | 0.494865 | 0.006166 | CTB-189B5.3; FBXO27 (margin: CTB-189B5.3; FBXO27) | 6.62 | 3.94e-04 | 5.86e-03 |
|  | 6 | 36355488 | 36355825 | PDFSTATSENSEMBLUCSC | 338 | 6 | 34936 | tss | 0.114272 | 0.965613 | RP1-50J22.4; ETV7 (margin: RP1-50J22.4; ETV7; PXT1) | 6.61 | 8.66e-03 | 3.36e-02 |
|  | 7 | 37488162 | 37489005 | PDFSTATSENSEMBLUCSC | 844 | 15 | 38416 | island | 0.858881 | 0.052875 | ELMO1 (margin: ELMO1) | 6.61 | 1.55e-07 | 2.60e-05 |
|  | 7 | 37488564 | 37489005 | PDFSTATSENSEMBLUCSC | 442 | 11 | 37541 | tss | 0.843908 | 0.052875 | ELMO1 (margin: ELMO1) | 6.61 | 3.40e-05 | 1.17e-03 |
|  | 2 | 136875144 | 136875349 | PDFSTATSENSEMBLUCSC | 206 | 5 | 22886 | gene;island | 0.528429 | 0.020206 | CXCR4 (margin: CXCR4) | 6.60 | 7.94e-03 | 3.19e-02 |
|  | 15 | 35087506 | 35087773 | PDFSTATSENSEMBLUCSC | 268 | 5 | 13281 | gene | 0.132278 | 0.949119 | RP11-814P5.1; ACTC1 (margin: RP11-814P5.1; ACTC1) | 6.60 | 7.94e-03 | 3.19e-02 |
|  | 16 | 58548873 | 58549320 | PDFSTATSENSEMBLUCSC | 448 | 8 | 15275 | tss;island | 0.327962 | 0.013806 | NDRG4; SETD6 (margin: NDRG4; SETD6; CNOT1) | 6.60 | 1.09e-03 | 1.10e-02 |
|  | 1 | 92949337 | 92950836 | PDFSTATSENSEMBLUCSC | 1500 | 31 | 635 | gene;island | 0.245164 | 0.974474 | GFI1 (margin: GFI1) | 6.59 | 2.50e-09 | 8.71e-07 |
|  | 19 | 54023732 | 54024435 | PDFSTATSENSEMBLUCSC | 704 | 12 | 22380 | island | 0.727632 | 0.005698 | ZNF331 (margin: CTD-2224J9.8; ZNF331) | 6.59 | 1.11e-03 | 1.12e-02 |
|  | 19 | 54023732 | 54024110 | PDFSTATSENSEMBLUCSC | 379 | 8 | 21030 | tss | 0.861365 | 0.024017 | ZNF331 (margin: CTD-2224J9.8; ZNF331) | 6.59 | 1.04e-02 | 3.96e-02 |
|  | 6 | 29894619 | 29895260 | PDFSTATSENSEMBLUCSC | 642 | 25 | 35642 | island | 0.080932 | 0.920289 | HLA-K; HCG4B (margin: HLA-K; HLA-U; HCG4B) | 6.59 | 1.56e-09 | 6.31e-07 |
|  | 11 | 6440279 | 6440482 | PDFSTATSENSEMBLUCSC | 204 | 6 | 6490 | gene;island | 0.581947 | 0.017068 | APBB1 (margin: APBB1; HPX) | 6.59 | 2.16e-03 | 1.45e-02 |
|  | 7 | 3341524 | 3342048 | PDFSTATSENSEMBLUCSC | 525 | 6 | 36823 | gene;island | 0.547954 | 0.034528 | SDK1; AC073316.1 (margin: SDK1; AC073316.1) | 6.59 | 2.16e-03 | 1.45e-02 |
|  | 6 | 146136083 | 146136749 | PDFSTATSENSEMBLUCSC | 667 | 10 | 35273 | tss;island | 0.011085 | 0.503427 | RP11-545I5.3; FBXO30 (margin: RP11-545I5.3; FBXO30) | 6.59 | 2.09e-03 | 1.45e-02 |
|  | 10 | 17270087 | 17270250 | PDFSTATSENSEMBLUCSC | 164 | 5 | 4969 | tss | 0.323860 | 0.967403 | VIM; VIM-AS1 (margin: VIM; VIM-AS1; RP11-124N14.3) | 6.58 | 7.94e-03 | 3.19e-02 |
|  | 6 | 88875236 | 88875844 | PDFSTATSENSEMBLUCSC | 609 | 8 | 36315 | island | 0.506668 | 0.026711 | CNR1 (margin: CNR1) | 6.58 | 1.55e-04 | 2.71e-03 |
|  | 18 | 35146001 | 35146770 | PDFSTATSENSEMBLUCSC | 770 | 20 | 19246 | tss | 0.730509 | 0.007968 | CELF4 (margin: CELF4) | 6.58 | 1.33e-08 | 3.50e-06 |
|  | 17 | 75471193 | 75471491 | PDFSTATSENSEMBLUCSC | 299 | 6 | 16811 | gene | 0.347778 | 0.979823 | SEPT9 (margin: SEPT9; RP11-75C10.7; RP11-75C10.9) | 6.57 | 2.16e-03 | 1.45e-02 |
|  | 5 | 91988 | 92385 | PDFSTATSENSEMBLUCSC | 398 | 5 | 32571 | island | 0.107656 | 0.918667 | CTD-2231H16.1 (margin: CTD-2231H16.1) | 6.57 | 7.94e-03 | 3.19e-02 |
|  | 14 | 79744991 | 79745592 | PDFSTATSENSEMBLUCSC | 602 | 7 | 12159 | gene;tss;island | 0.602879 | 0.012765 | NRXN3 (margin: NRXN3) | 6.57 | 4.08e-03 | 2.47e-02 |
|  | 2 | 228582758 | 228582820 | PDFSTATSENSEMBLUCSC | 63 | 5 | 23931 | tss | 0.156773 | 0.001916 | SLC19A3 (margin: SLC19A3) | 6.55 | 7.94e-03 | 3.19e-02 |
|  | 1 | 65775467 | 65775772 | PDFSTATSENSEMBLUCSC | 306 | 5 | 570 | gene;island | 0.619651 | 0.010243 | DNAJC6 (margin: DNAJC6; RP5-1044H5.1; RP5-1044H5.2) | 6.55 | 7.94e-03 | 3.19e-02 |
|  | 1 | 203598573 | 203599089 | PDFSTATSENSEMBLUCSC | 517 | 6 | 953 | gene;island | 0.262317 | 0.981518 | ATP2B4 (margin: ATP2B4) | 6.54 | 2.16e-03 | 1.45e-02 |
|  | 13 | 113776873 | 113777109 | PDFSTATSENSEMBLUCSC | 237 | 8 | 11548 | tss | 0.115509 | 0.933082 | F7; F10 (margin: F7; F10; KARSP2; F10-AS1) | 6.54 | 1.55e-04 | 2.71e-03 |
|  | 2 | 31456964 | 31457061 | PDFSTATSENSEMBLUCSC | 98 | 5 | 22638 | gene | 0.090967 | 0.004553 | EHD3; CAPN14 (margin: RNA5SP90; EHD3; CAPN14) | 6.53 | 7.94e-03 | 3.19e-02 |
|  | 20 | 37230326 | 37230741 | PDFSTATSENSEMBLUCSC | 416 | 7 | 26158 | island | 0.037851 | 0.835436 | ARHGAP40 (margin: ARHGAP40) | 6.53 | 5.83e-04 | 6.47e-03 |
|  | 20 | 9819697 | 9819820 | PDFSTATSENSEMBLUCSC | 124 | 6 | 25593 | tss;island | 0.309722 | 0.006363 | PAK7 (margin: PAK7) | 6.53 | 2.16e-03 | 1.45e-02 |
|  | 2 | 210636350 | 210636844 | PDFSTATSENSEMBLUCSC | 495 | 8 | 25053 | island | 0.754735 | 0.036160 | UNC80 (margin: UNC80) | 6.53 | 3.11e-04 | 4.77e-03 |
|  | 13 | 20805094 | 20805679 | PDFSTATSENSEMBLUCSC | 586 | 10 | 11096 | gene;island | 0.152697 | 0.927213 | GJB6 (margin: GJB6) | 6.52 | 1.08e-05 | 4.82e-04 |
|  | 13 | 36920660 | 36921174 | PDFSTATSENSEMBLUCSC | 515 | 12 | 11394 | tss | 0.223460 | 0.005104 | SPG20OS; SPG20 (margin: SPG20OS; AL139377.1; SPG20) | 6.52 | 3.64e-03 | 2.29e-02 |
|  | 1 | 49242616 | 49242934 | PDFSTATSENSEMBLUCSC | 319 | 5 | 1612 | tss | 0.738194 | 0.015883 | AGBL4; BEND5 (margin: AGBL4; BEND5) | 6.52 | 7.94e-03 | 3.19e-02 |
|  | 3 | 48885189 | 48885665 | PDFSTATSENSEMBLUCSC | 477 | 10 | 29098 | island | 0.005880 | 0.386344 | PRKAR2A-AS1; PRKAR2A (margin: PRKAR2A-AS1; PRKAR2A; SLC25A20) | 6.52 | 2.06e-04 | 3.42e-03 |
|  | 1 | 2461820 | 2461929 | PDFSTATSENSEMBLUCSC | 110 | 6 | 1175 | tss;island | 0.557598 | 0.989228 | HES5 (margin: PANK4; HES5) | 6.52 | 2.16e-03 | 1.45e-02 |
|  | 21 | 39288661 | 39289266 | PDFSTATSENSEMBLUCSC | 606 | 8 | 26681 | island | 0.536400 | 0.030395 | KCNJ6 (margin: KCNJ6) | 6.51 | 3.11e-04 | 4.77e-03 |
|  | 1 | 177140126 | 177140609 | PDFSTATSENSEMBLUCSC | 484 | 7 | 2108 | tss | 0.957226 | 0.077755 | FAM5B (margin: FAM5B; ASTN1) | 6.51 | 6.99e-03 | 3.19e-02 |
|  | 3 | 161089871 | 161091175 | PDFSTATSENSEMBLUCSC | 1305 | 14 | 28698 | tss | 0.529137 | 0.014096 | SPTSSB (margin: SPTSSB) | 6.51 | 2.53e-05 | 1.02e-03 |
|  | 1 | 23751134 | 23751761 | PDFSTATSENSEMBLUCSC | 628 | 9 | 2835 | island | 0.700462 | 0.031916 | TCEA3 (margin: TCEA3; ASAP3) | 6.51 | 4.11e-05 | 1.17e-03 |
|  | 10 | 44069847 | 44070200 | PDFSTATSENSEMBLUCSC | 354 | 7 | 5700 | island | 0.480529 | 0.010686 | ZNF239 (margin: ZNF239) | 6.50 | 5.83e-04 | 6.47e-03 |
|  | 2 | 197457277 | 197457430 | PDFSTATSENSEMBLUCSC | 154 | 6 | 24975 | island | 0.594782 | 0.016642 | HECW2 (margin: HECW2) | 6.50 | 2.16e-03 | 1.45e-02 |
|  | 2 | 223916502 | 223916952 | PDFSTATSENSEMBLUCSC | 451 | 7 | 25157 | island | 0.266617 | 0.981798 | KCNE4 (margin: KCNE4) | 6.50 | 2.33e-03 | 1.52e-02 |
|  | 11 | 122852284 | 122852698 | PDFSTATSENSEMBLUCSC | 415 | 9 | 8904 | island | 0.936536 | 0.171709 | BSX (margin: BSX) | 6.50 | 2.88e-04 | 4.58e-03 |
|  | 3 | 49941376 | 49941613 | PDFSTATSENSEMBLUCSC | 238 | 7 | 28339 | tss;island | 0.011266 | 0.453635 | CTD-2330K9.2; CTD-2330K9.3; MST1R (margin: CTD-2330K9.2; CTD-2330K9.3; MST1R; MON1A) | 6.50 | 5.83e-04 | 6.47e-03 |
|  | 3 | 128336483 | 128337044 | PDFSTATSENSEMBLUCSC | 562 | 8 | 29456 | island | 0.543072 | 0.015749 | RPN1 (margin: RPN1) | 6.50 | 1.55e-04 | 2.71e-03 |
|  | 8 | 17433926 | 17434541 | PDFSTATSENSEMBLUCSC | 616 | 9 | 39567 | tss;island | 0.559855 | 0.011597 | PDGFRL (margin: SLC7A2; PDGFRL) | 6.49 | 1.23e-03 | 1.18e-02 |
|  | 6 | 31126599 | 31127527 | PDFSTATSENSEMBLUCSC | 929 | 22 | 33860 | gene;island | 0.009629 | 0.701390 | TCF19; CCHCR1 (margin: TCF19; CCHCR1; POU5F1) | 6.49 | 1.96e-07 | 2.62e-05 |
|  | 5 | 113697861 | 113698506 | PDFSTATSENSEMBLUCSC | 646 | 8 | 33017 | island | 0.918513 | 0.045542 | KCNN2 (margin: KCNN2) | 6.48 | 6.22e-04 | 6.71e-03 |
|  | 16 | 90114063 | 90114307 | PDFSTATSENSEMBLUCSC | 245 | 6 | 15437 | tss | 0.476131 | 0.015025 | URAHP (margin: GAS8; URAHP; PRDM7) | 6.48 | 2.16e-03 | 1.45e-02 |
|  | 19 | 37064156 | 37064886 | PDFSTATSENSEMBLUCSC | 731 | 13 | 20003 | gene;island | 0.009136 | 0.396197 | AC092295.7; ZNF529 (margin: AC092295.7; ZNF529) | 6.48 | 1.92e-07 | 2.60e-05 |
|  | 19 | 54668230 | 54668604 | PDFSTATSENSEMBLUCSC | 375 | 5 | 20218 | gene;island | 0.969556 | 0.394043 | TMC4 (margin: CNOT3; LENG1; TMC4; MBOAT7) | 6.48 | 7.94e-03 | 3.19e-02 |
|  | 2 | 223160102 | 223160569 | PDFSTATSENSEMBLUCSC | 468 | 5 | 23045 | gene;island | 0.458132 | 0.984497 | PAX3 (margin: CCDC140; PAX3) | 6.48 | 7.94e-03 | 3.19e-02 |
|  | 20 | 170364 | 170641 | PDFSTATSENSEMBLUCSC | 278 | 5 | 25521 | tss | 0.192470 | 0.952277 | DEFB128 (margin: DEFB128; AL360078.1) | 6.47 | 7.94e-03 | 3.19e-02 |
|  | 1 | 1141617 | 1142405 | PDFSTATSENSEMBLUCSC | 789 | 9 | 2414 | island | 0.627937 | 0.032601 | TNFRSF18 (margin: TTLL10; TNFRSF18; TNFRSF4; SDF4) | 6.47 | 1.65e-04 | 2.80e-03 |
|  | 2 | 17721431 | 17722068 | PDFSTATSENSEMBLUCSC | 638 | 11 | 24094 | island | 0.032765 | 0.624398 | VSNL1 (margin: VSNL1) | 6.46 | 2.84e-06 | 1.96e-04 |
|  | 1 | 39874802 | 39875238 | PDFSTATSENSEMBLUCSC | 437 | 13 | 446 | gene;island | 0.749083 | 0.019585 | MACF1; KIAA0754 (margin: MACF1; KIAA0754) | 6.46 | 1.29e-05 | 5.58e-04 |
|  | 10 | 77871958 | 77872458 | PDFSTATSENSEMBLUCSC | 501 | 5 | 4646 | gene;island | 0.823891 | 0.011326 | C10orf11 (margin: C10orf11) | 6.46 | 7.94e-03 | 3.19e-02 |
|  | 12 | 81471311 | 81472177 | PDFSTATSENSEMBLUCSC | 867 | 13 | 10650 | island | 0.536744 | 0.011255 | ACSS3 (margin: ACSS3) | 6.46 | 1.92e-07 | 2.60e-05 |
|  | 16 | 86370962 | 86371442 | PDFSTATSENSEMBLUCSC | 481 | 6 | 14883 | gene | 0.085211 | 0.901485 | LINC00917 (margin: RP11-158I3.3; LINC00917) | 6.46 | 2.16e-03 | 1.45e-02 |
|  | 3 | 47620439 | 47620545 | PDFSTATSENSEMBLUCSC | 107 | 6 | 28288 | tss;island | 0.761374 | 0.025960 | CSPG5 (margin: CSPG5; SMARCC1) | 6.45 | 2.16e-03 | 1.45e-02 |
|  | 12 | 64784483 | 64784686 | PDFSTATSENSEMBLUCSC | 204 | 5 | 9841 | tss;island | 0.379663 | 0.977796 | RPS11P6; C12orf56 (margin: RPS11P6; C12orf56) | 6.45 | 7.94e-03 | 3.19e-02 |
|  | 6 | 33160250 | 33160604 | PDFSTATSENSEMBLUCSC | 355 | 9 | 34889 | tss | 0.282673 | 0.947236 | COL11A2; RXRB (margin: RNY4P10; SLC39A7; HSD17B8; COL11A2; RXRB) | 6.45 | 4.11e-05 | 1.17e-03 |
|  | 5 | 52083947 | 52084055 | PDFSTATSENSEMBLUCSC | 109 | 6 | 31610 | gene | 0.012348 | 0.421759 | ITGA1; PELO; CTD-2288O8.1 (margin: ITGA1; PELO; CTD-2288O8.1) | 6.45 | 2.16e-03 | 1.45e-02 |
|  | 1 | 201508965 | 201509415 | PDFSTATSENSEMBLUCSC | 451 | 5 | 4078 | island | 0.910190 | 0.045115 | (margin: RP11-134G8.7; RPS10P7) | 6.44 | 7.94e-03 | 3.19e-02 |
|  | 3 | 44770851 | 44771316 | PDFSTATSENSEMBLUCSC | 466 | 11 | 29041 | island | 0.273772 | 0.929794 | ZNF501 (margin: ZNF502; ZNF501; KIAA1143) | 6.43 | 5.67e-06 | 3.54e-04 |
|  | 3 | 44770851 | 44771113 | PDFSTATSENSEMBLUCSC | 263 | 9 | 28261 | tss | 0.321675 | 0.976770 | ZNF501 (margin: ZNF502; ZNF501; KIAA1143) | 6.43 | 8.23e-05 | 2.06e-03 |
|  | 8 | 144660590 | 144660772 | PDFSTATSENSEMBLUCSC | 183 | 5 | 39953 | tss | 0.550473 | 0.990780 | RP11-661A12.9; RP11-661A12.7; NAPRT1; EEF1D (margin: RP11-661A12.9; RP11-661A12.7; MROH6; NAPRT1; EEF1D) | 6.43 | 7.94e-03 | 3.19e-02 |
|  | 4 | 25090198 | 25090665 | PDFSTATSENSEMBLUCSC | 468 | 6 | 30958 | island | 0.331168 | 0.972069 | (margin: ) | 6.42 | 2.16e-03 | 1.45e-02 |
|  | 1 | 37500195 | 37500508 | PDFSTATSENSEMBLUCSC | 314 | 5 | 1499 | tss;island | 0.891274 | 0.055756 | GRIK3 (margin: GRIK3) | 6.42 | 7.94e-03 | 3.19e-02 |
|  | 5 | 180230767 | 180231194 | PDFSTATSENSEMBLUCSC | 428 | 9 | 31947 | gene;tss;island | 0.136342 | 0.972816 | MGAT1 (margin: MGAT1) | 6.41 | 4.11e-05 | 1.17e-03 |
|  | 3 | 56502021 | 56502293 | PDFSTATSENSEMBLUCSC | 273 | 5 | 27840 | gene;island | 0.384032 | 0.010285 | ERC2 (margin: ERC2) | 6.40 | 7.94e-03 | 3.19e-02 |
|  | 15 | 52587751 | 52588279 | PDFSTATSENSEMBLUCSC | 529 | 8 | 14149 | island | 0.476469 | 0.011951 | MYO5C (margin: MYO5C; MYO5A) | 6.40 | 1.55e-04 | 2.71e-03 |
|  | 6 | 152126736 | 152126938 | PDFSTATSENSEMBLUCSC | 203 | 5 | 34512 | gene;island | 0.132970 | 0.866096 | ESR1 (margin: ESR1; RP3-443C4.2) | 6.40 | 7.94e-03 | 3.19e-02 |
|  | 19 | 37959744 | 37960107 | PDFSTATSENSEMBLUCSC | 364 | 8 | 21956 | island | 0.505132 | 0.011363 | ZNF570; ZNF569 (margin: ZNF570; ZNF569) | 6.39 | 6.99e-03 | 3.19e-02 |
|  | 6 | 42946962 | 42947317 | PDFSTATSENSEMBLUCSC | 356 | 9 | 36146 | island | 0.014483 | 0.635762 | PEX6 (margin: PPP2R5D; PEX6) | 6.39 | 4.11e-05 | 1.17e-03 |
|  | 7 | 27204663 | 27205114 | PDFSTATSENSEMBLUCSC | 452 | 7 | 36938 | gene | 0.293685 | 0.951591 | HOXA9; RP1-170O19.20 (margin: HOXA-AS3; RP1-170O19.21; HOXA-AS4; HOXA7; HOXA9; RP1-170O19.20; MIR196B; HOXA10) | 6.38 | 5.83e-04 | 6.47e-03 |
|  | 6 | 151814892 | 151814994 | PDFSTATSENSEMBLUCSC | 103 | 5 | 35301 | tss;island | 0.564496 | 0.019981 | CCDC170 (margin: CCDC170) | 6.38 | 7.94e-03 | 3.19e-02 |
|  | 4 | 122302226 | 122302421 | PDFSTATSENSEMBLUCSC | 196 | 7 | 30570 | tss;island | 0.315206 | 0.978108 | QRFPR (margin: QRFPR; RP11-364P2.2) | 6.38 | 5.83e-04 | 6.47e-03 |
|  | 6 | 55443757 | 55444488 | PDFSTATSENSEMBLUCSC | 732 | 10 | 36218 | island | 0.649865 | 0.037714 | HMGCLL1 (margin: HMGCLL1) | 6.38 | 4.33e-05 | 1.22e-03 |
|  | 2 | 12856626 | 12856970 | PDFSTATSENSEMBLUCSC | 345 | 9 | 23194 | tss | 0.180360 | 0.004155 | RP11-333O1.1; TRIB2 (margin: RP11-333O1.1; TRIB2) | 6.38 | 4.11e-05 | 1.17e-03 |
|  | 2 | 228582688 | 228582820 | PDFSTATSENSEMBLUCSC | 133 | 6 | 25168 | island | 0.309152 | 0.001938 | SLC19A3 (margin: SLC19A3) | 6.38 | 2.16e-03 | 1.45e-02 |
|  | 15 | 79383385 | 79383980 | PDFSTATSENSEMBLUCSC | 596 | 14 | 13842 | tss;island | 0.540917 | 0.008829 | RASGRF1 (margin: RASGRF1) | 6.37 | 4.99e-08 | 9.34e-06 |
|  | 6 | 71122912 | 71123290 | PDFSTATSENSEMBLUCSC | 379 | 8 | 36238 | island | 0.011426 | 0.508432 | FAM135A; RP11-462G2.2 (margin: FAM135A; RP11-462G2.2) | 6.37 | 1.55e-04 | 2.71e-03 |
|  | 17 | 6899085 | 6899380 | PDFSTATSENSEMBLUCSC | 296 | 9 | 17056 | tss | 0.325649 | 0.980788 | ALOX12; AC027763.2; RP11-589P10.7; RP11-589P10.5 (margin: ALOX12; AC027763.2; RP11-589P10.7; RP11-589P10.5) | 6.36 | 4.11e-05 | 1.17e-03 |
|  | 20 | 749264 | 749620 | PDFSTATSENSEMBLUCSC | 357 | 6 | 25529 | tss | 0.013551 | 0.627063 | SLC52A3 (margin: SLC52A3) | 6.36 | 2.16e-03 | 1.45e-02 |
|  | 6 | 33172442 | 33172572 | PDFSTATSENSEMBLUCSC | 131 | 6 | 34227 | gene | 0.010851 | 0.455195 | SLC39A7; HSD17B8 (margin: RNY4P10; SLC39A7; HSD17B8; MIR219-1; RING1; ZNF70P1; RXRB) | 6.36 | 2.16e-03 | 1.45e-02 |
|  | 11 | 63438586 | 63439313 | PDFSTATSENSEMBLUCSC | 728 | 13 | 8371 | island | 0.723416 | 0.013477 | ATL3 (margin: RTN3; ATL3) | 6.36 | 1.30e-04 | 2.71e-03 |
|  | 4 | 113970617 | 113970935 | PDFSTATSENSEMBLUCSC | 319 | 6 | 30103 | gene | 0.041525 | 0.823644 | ANK2; RP11-650J17.1 (margin: ANK2; RP11-650J17.1) | 6.36 | 4.33e-03 | 2.52e-02 |
|  | 8 | 67344553 | 67344665 | PDFSTATSENSEMBLUCSC | 113 | 6 | 39742 | tss | 0.413016 | 0.981792 | RRS1; ADHFE1 (margin: RRS1; ADHFE1; RP11-346I3.4) | 6.34 | 2.16e-03 | 1.45e-02 |
|  | 13 | 114880761 | 114881194 | PDFSTATSENSEMBLUCSC | 434 | 6 | 11316 | gene;island | 0.296170 | 0.970036 | RASA3 (margin: RASA3; RASA3-IT1) | 6.34 | 2.16e-03 | 1.45e-02 |
|  | 1 | 99729757 | 99729807 | PDFSTATSENSEMBLUCSC | 51 | 6 | 1773 | tss;island | 0.794715 | 0.067753 | LPPR4 (margin: LPPR4) | 6.34 | 2.16e-03 | 1.45e-02 |
|  | 11 | 111847692 | 111848400 | PDFSTATSENSEMBLUCSC | 709 | 10 | 6903 | gene;island | 0.702735 | 0.030183 | DIXDC1 (margin: DIXDC1) | 6.33 | 8.93e-03 | 3.45e-02 |
|  | 15 | 83953690 | 83954395 | PDFSTATSENSEMBLUCSC | 706 | 18 | 13865 | tss;island | 0.122262 | 0.926995 | RP11-382A20.4; BNC1 (margin: RP11-382A20.4; BNC1) | 6.33 | 2.20e-10 | 1.16e-07 |
|  | 7 | 12610355 | 12610833 | PDFSTATSENSEMBLUCSC | 479 | 6 | 36896 | gene | 0.684784 | 0.046006 | SCIN; AC005281.2 (margin: SCIN; AC005281.2) | 6.32 | 2.16e-03 | 1.45e-02 |
|  | 2 | 43903470 | 43904011 | PDFSTATSENSEMBLUCSC | 542 | 8 | 22660 | gene;tss | 0.207571 | 0.004602 | PLEKHH2; AC011242.6 (margin: PLEKHH2; AC011242.6; AC011242.5) | 6.32 | 6.99e-03 | 3.19e-02 |
|  | 5 | 139927007 | 139927242 | PDFSTATSENSEMBLUCSC | 236 | 8 | 32342 | tss | 0.010983 | 0.458789 | ANKHD1; ANKHD1-EIF4EBP3; EIF4EBP3; SRA1 (margin: ANKHD1; ANKHD1-EIF4EBP3; EIF4EBP3; SRA1; APBB3) | 6.32 | 1.55e-04 | 2.71e-03 |
|  | 6 | 32050905 | 32051108 | PDFSTATSENSEMBLUCSC | 204 | 5 | 34037 | gene;island | 0.024513 | 0.656600 | TNXB (margin: TNXB; RNA5SP206) | 6.31 | 7.94e-03 | 3.19e-02 |
|  | 17 | 42733527 | 42733729 | PDFSTATSENSEMBLUCSC | 203 | 8 | 17412 | tss;island | 0.920764 | 0.221093 | C17orf104 (margin: RP11-1072C15.3; C17orf104; RP11-1072C15.2) | 6.31 | 1.55e-04 | 2.71e-03 |
|  | 7 | 134576049 | 134576386 | PDFSTATSENSEMBLUCSC | 338 | 6 | 37233 | gene | 0.011827 | 0.474737 | CALD1 (margin: CALD1) | 6.31 | 2.16e-03 | 1.45e-02 |
|  | 3 | 44753865 | 44754131 | PDFSTATSENSEMBLUCSC | 267 | 8 | 28260 | tss | 0.395013 | 0.976595 | ZNF502; SOCS5P3 (margin: ZNF502; SOCS5P3) | 6.31 | 1.86e-03 | 1.45e-02 |
|  | 8 | 67344553 | 67344720 | PDFSTATSENSEMBLUCSC | 168 | 7 | 40354 | island | 0.435886 | 0.984524 | RRS1; ADHFE1 (margin: RRS1; ADHFE1; RP11-346I3.4) | 6.30 | 5.83e-04 | 6.47e-03 |
|  | 10 | 97667581 | 97667923 | PDFSTATSENSEMBLUCSC | 343 | 6 | 5969 | island | 0.546975 | 0.019100 | RP11-248J23.7; C10orf131; ENTPD1-AS1 (margin: RP11-248J23.7; C10orf131; ENTPD1-AS1) | 6.30 | 2.16e-03 | 1.45e-02 |
|  | 3 | 48885314 | 48885665 | PDFSTATSENSEMBLUCSC | 352 | 9 | 28308 | tss | 0.005971 | 0.373760 | PRKAR2A-AS1; PRKAR2A (margin: PRKAR2A-AS1; PRKAR2A; SLC25A20) | 6.30 | 7.82e-04 | 8.17e-03 |
|  | 2 | 12858298 | 12858655 | PDFSTATSENSEMBLUCSC | 358 | 5 | 22577 | gene;island | 0.225473 | 0.832890 | RP11-333O1.1; TRIB2 (margin: RP11-333O1.1; TRIB2) | 6.29 | 7.94e-03 | 3.19e-02 |
|  | 11 | 66824138 | 66824219 | PDFSTATSENSEMBLUCSC | 82 | 5 | 7483 | tss;island | 0.008882 | 0.371961 | RHOD (margin: SYT12; RHOD) | 6.29 | 7.94e-03 | 3.19e-02 |
|  | 13 | 70682525 | 70682771 | PDFSTATSENSEMBLUCSC | 247 | 5 | 11176 | gene;island | 0.130157 | 0.980361 | ATXN8OS; KLHL1 (margin: ATXN8OS; KLHL1) | 6.29 | 7.94e-03 | 3.19e-02 |
|  | 19 | 34111400 | 34111733 | PDFSTATSENSEMBLUCSC | 334 | 5 | 20686 | tss;island | 0.411950 | 0.979887 | CHST8 (margin: CHST8) | 6.29 | 7.94e-03 | 3.19e-02 |
|  | 18 | 47792917 | 47793175 | PDFSTATSENSEMBLUCSC | 259 | 6 | 19274 | tss | 0.281794 | 0.007149 | CCDC11; MBD1 (margin: CCDC11; MBD1) | 6.29 | 2.16e-03 | 1.45e-02 |
|  | 13 | 36050788 | 36051073 | PDFSTATSENSEMBLUCSC | 286 | 8 | 11127 | gene;island | 0.138508 | 0.902072 | NBEA; MAB21L1 (margin: NBEA; MAB21L1) | 6.29 | 1.55e-04 | 2.71e-03 |
|  | 8 | 145018010 | 145018300 | PDFSTATSENSEMBLUCSC | 291 | 5 | 39499 | gene;tss;island | 0.547107 | 0.018419 | PLEC; MIR661 (margin: PLEC; MIR661) | 6.28 | 7.94e-03 | 3.19e-02 |
|  | 7 | 12609907 | 12610833 | PDFSTATSENSEMBLUCSC | 927 | 15 | 38267 | island | 0.431974 | 0.009856 | SCIN; AC005281.2 (margin: SCIN; AC005281.2) | 6.28 | 3.87e-07 | 4.83e-05 |
|  | 7 | 12609907 | 12610196 | PDFSTATSENSEMBLUCSC | 290 | 9 | 37445 | tss | 0.228348 | 0.007662 | SCIN; AC005281.2 (margin: SCIN; AC005281.2) | 6.28 | 4.11e-05 | 1.17e-03 |
|  | 4 | 96468962 | 96469634 | PDFSTATSENSEMBLUCSC | 673 | 9 | 30063 | gene;island | 0.669987 | 0.010880 | RP11-710C12.1; UNC5C (margin: RP11-710C12.1; UNC5C) | 6.27 | 1.65e-04 | 2.80e-03 |
|  | 20 | 62680970 | 62681428 | PDFSTATSENSEMBLUCSC | 459 | 11 | 26380 | island | 0.815018 | 0.013399 | TCEA2; ZNF512B; SOX18 (margin: LINC00176; TCEA2; RP13-152O15.5; ZNF512B; SOX18) | 6.27 | 1.28e-04 | 2.71e-03 |
|  | 20 | 62680986 | 62681428 | PDFSTATSENSEMBLUCSC | 443 | 9 | 25909 | tss | 0.818539 | 0.013399 | TCEA2; ZNF512B; SOX18 (margin: LINC00176; TCEA2; RP13-152O15.5; ZNF512B; SOX18) | 6.27 | 7.82e-04 | 8.17e-03 |
|  | 12 | 45269519 | 45269790 | PDFSTATSENSEMBLUCSC | 272 | 5 | 9651 | tss | 0.601295 | 0.019498 | NELL2 (margin: NELL2; Y\_RNA) | 6.27 | 7.94e-03 | 3.19e-02 |
|  | 4 | 74964856 | 74965278 | PDFSTATSENSEMBLUCSC | 423 | 11 | 31102 | island | 0.011064 | 0.363721 | CXCL2 (margin: CXCL2) | 6.27 | 2.84e-06 | 1.96e-04 |
|  | 4 | 74965068 | 74965278 | PDFSTATSENSEMBLUCSC | 211 | 9 | 30434 | tss | 0.011064 | 0.350260 | CXCL2 (margin: CXCL2) | 6.27 | 4.11e-05 | 1.17e-03 |
|  | 3 | 168863860 | 168864313 | PDFSTATSENSEMBLUCSC | 454 | 7 | 28020 | gene;island | 0.009253 | 0.248934 | MECOM (margin: MECOM) | 6.27 | 5.83e-04 | 6.47e-03 |
|  | 11 | 65779192 | 65779343 | PDFSTATSENSEMBLUCSC | 152 | 6 | 7456 | tss | 0.237062 | 0.950759 | CST6 (margin: BANF1; CST6; EIF1AD; CATSPER1) | 6.26 | 2.16e-03 | 1.45e-02 |
|  | 2 | 189156874 | 189157566 | PDFSTATSENSEMBLUCSC | 693 | 11 | 24953 | island | 0.009169 | 0.441112 | GULP1 (margin: GULP1; MIR561; AC068718.1) | 6.26 | 8.51e-05 | 2.07e-03 |
|  | 2 | 189156874 | 189157361 | PDFSTATSENSEMBLUCSC | 488 | 9 | 23777 | tss | 0.009169 | 0.461662 | GULP1 (margin: GULP1; MIR561; AC068718.1) | 6.26 | 7.82e-04 | 8.17e-03 |
|  | 5 | 100238977 | 100239071 | PDFSTATSENSEMBLUCSC | 95 | 6 | 32196 | tss | 0.107932 | 0.004465 | ST8SIA4 (margin: ST8SIA4) | 6.26 | 2.16e-03 | 1.45e-02 |
|  | 3 | 138665654 | 138666473 | PDFSTATSENSEMBLUCSC | 820 | 13 | 28626 | tss;island | 0.445298 | 0.983005 | C3orf72; FOXL2 (margin: C3orf72; RP11-548O1.3; FOXL2) | 6.25 | 7.69e-07 | 7.42e-05 |
|  | 2 | 178937517 | 178937882 | PDFSTATSENSEMBLUCSC | 366 | 8 | 23751 | tss | 0.241980 | 0.011030 | PDE11A (margin: PDE11A) | 6.25 | 4.66e-03 | 2.68e-02 |
|  | 21 | 39288720 | 39289266 | PDFSTATSENSEMBLUCSC | 547 | 7 | 26544 | tss | 0.575433 | 0.033411 | KCNJ6 (margin: KCNJ6) | 6.25 | 1.17e-03 | 1.13e-02 |
|  | 16 | 30022548 | 30022725 | PDFSTATSENSEMBLUCSC | 178 | 7 | 15175 | tss;island | 0.279049 | 0.002162 | DOC2A (margin: INO80E; C16orf92; DOC2A) | 6.25 | 1.17e-03 | 1.13e-02 |
|  | 1 | 239549577 | 239549853 | PDFSTATSENSEMBLUCSC | 277 | 6 | 4370 | island | 0.786841 | 0.016996 | CHRM3 (margin: CHRM3) | 6.25 | 2.16e-03 | 1.45e-02 |
|  | 10 | 119304583 | 119304719 | PDFSTATSENSEMBLUCSC | 137 | 5 | 5392 | tss | 0.442560 | 0.984314 | EMX2; EMX2OS (margin: EMX2; EMX2OS) | 6.24 | 7.94e-03 | 3.19e-02 |
|  | 19 | 37329330 | 37329859 | PDFSTATSENSEMBLUCSC | 530 | 13 | 20730 | tss | 0.558811 | 0.011420 | ZNF790 (margin: CTD-2162K18.5; ZNF345; ZNF790) | 6.22 | 1.92e-07 | 2.60e-05 |
|  | 1 | 17308032 | 17308303 | PDFSTATSENSEMBLUCSC | 272 | 5 | 1288 | tss;island | 0.156133 | 0.950767 | RP1-37C10.3; MFAP2 (margin: CROCC; RP1-37C10.3; MFAP2; ATP13A2) | 6.22 | 7.94e-03 | 3.19e-02 |
|  | 8 | 63997977 | 63998778 | PDFSTATSENSEMBLUCSC | 802 | 11 | 40337 | island | 0.447284 | 0.022318 | TTPA (margin: TTPA) | 6.21 | 8.51e-05 | 2.07e-03 |
|  | 17 | 46670933 | 46671635 | PDFSTATSENSEMBLUCSC | 703 | 13 | 16655 | gene;island | 0.548156 | 0.029049 | HOXB-AS3; HOXB3; HOXB5; HOXB6 (margin: HOXB-AS3; HOXB3; MIR10A; HOXB5; HOXB6) | 6.21 | 2.28e-04 | 3.75e-03 |
|  | 3 | 161089712 | 161091175 | PDFSTATSENSEMBLUCSC | 1464 | 15 | 29638 | island | 0.487654 | 0.015737 | SPTSSB (margin: SPTSSB) | 6.21 | 8.82e-06 | 4.82e-04 |
|  | 2 | 201450323 | 201450743 | PDFSTATSENSEMBLUCSC | 421 | 11 | 25003 | island | 0.413673 | 0.980182 | SGOL2; AOX1 (margin: SGOL2; AOX1) | 6.20 | 2.84e-06 | 1.96e-04 |
|  | 2 | 201450323 | 201450690 | PDFSTATSENSEMBLUCSC | 368 | 9 | 23810 | tss | 0.457338 | 0.984032 | SGOL2; AOX1 (margin: SGOL2; AOX1) | 6.20 | 4.11e-05 | 1.17e-03 |
|  | 12 | 81331457 | 81331845 | PDFSTATSENSEMBLUCSC | 389 | 7 | 10649 | island | 0.971457 | 0.223871 | ACSS3; LIN7A; MIR618 (margin: ACSS3; LIN7A; MIR618) | 6.20 | 6.99e-03 | 3.19e-02 |
|  | 5 | 131592959 | 131593413 | PDFSTATSENSEMBLUCSC | 455 | 10 | 33086 | island | 0.277509 | 0.957158 | PDLIM4; P4HA2 (margin: PDLIM4; P4HA2) | 6.19 | 1.30e-04 | 2.71e-03 |
|  | 10 | 94833042 | 94833214 | PDFSTATSENSEMBLUCSC | 173 | 6 | 5234 | tss;island | 0.865288 | 0.050848 | CYP26A1 (margin: CYP26C1; CYP26A1) | 6.18 | 2.16e-03 | 1.45e-02 |
|  | 19 | 4769531 | 4769690 | PDFSTATSENSEMBLUCSC | 160 | 7 | 19739 | gene;tss | 0.407219 | 0.005552 | MIR7-3HG; MIR7-3 (margin: MIR7-3HG; MIR7-3) | 6.18 | 5.83e-04 | 6.47e-03 |
|  | 16 | 4421445 | 4421654 | PDFSTATSENSEMBLUCSC | 210 | 5 | 14638 | gene;tss;island | 0.838493 | 0.115543 | VASN; CORO7-PAM16; CORO7 (margin: VASN; CORO7-PAM16; CORO7) | 6.18 | 7.94e-03 | 3.19e-02 |
|  | 12 | 2800755 | 2801158 | PDFSTATSENSEMBLUCSC | 404 | 5 | 9007 | gene;island | 0.068173 | 0.959282 | CACNA1C; CACNA1C-AS1 (margin: CACNA1C; CACNA1C-AS1) | 6.18 | 7.94e-03 | 3.19e-02 |
|  | 11 | 69468789 | 69469175 | PDFSTATSENSEMBLUCSC | 387 | 6 | 6782 | gene | 0.932932 | 0.316453 | CCND1; ORAOV1 (margin: CCND1; ORAOV1) | 6.17 | 2.16e-03 | 1.45e-02 |
|  | 17 | 15244970 | 15245237 | PDFSTATSENSEMBLUCSC | 268 | 7 | 17150 | tss | 0.349483 | 0.980780 | TEKT3 (margin: TEKT3) | 6.16 | 5.83e-04 | 6.47e-03 |
|  | 19 | 45996372 | 45996644 | PDFSTATSENSEMBLUCSC | 273 | 6 | 20099 | gene;island | 0.587822 | 0.030170 | PPM1N; RTN2 (margin: PPM1N; RTN2) | 6.16 | 2.16e-03 | 1.45e-02 |
|  | 10 | 99531765 | 99531934 | PDFSTATSENSEMBLUCSC | 170 | 6 | 5272 | tss | 0.325937 | 0.970258 | SFRP5 (margin: ZFYVE27; SFRP5) | 6.16 | 8.66e-03 | 3.36e-02 |
|  | 11 | 1029029 | 1029336 | PDFSTATSENSEMBLUCSC | 308 | 5 | 6400 | gene;island | 0.374158 | 0.966743 | MUC6 (margin: MUC6) | 6.16 | 7.94e-03 | 3.19e-02 |
|  | 17 | 40346279 | 40346798 | PDFSTATSENSEMBLUCSC | 520 | 8 | 17358 | tss | 0.054685 | 0.714715 | GHDC (margin: HCRT; GHDC; STAT5B) | 6.15 | 3.11e-04 | 4.77e-03 |
|  | 10 | 118084109 | 118084587 | PDFSTATSENSEMBLUCSC | 479 | 6 | 4775 | gene | 0.087577 | 0.912214 | CCDC172 (margin: CCDC172) | 6.15 | 4.33e-03 | 2.52e-02 |
|  | 1 | 220921855 | 220922217 | PDFSTATSENSEMBLUCSC | 363 | 6 | 1020 | gene | 0.456907 | 0.011596 | MARC2 (margin: MARC2) | 6.14 | 2.16e-03 | 1.45e-02 |
|  | 17 | 71161157 | 71161502 | PDFSTATSENSEMBLUCSC | 346 | 7 | 18699 | island | 0.694264 | 0.031428 | RP11-143K11.5; SSTR2; POLR3KP2 (margin: RP11-143K11.5; SSTR2; RP11-143K11.1; POLR3KP2) | 6.13 | 5.83e-04 | 6.47e-03 |
|  | 3 | 10206254 | 10206537 | PDFSTATSENSEMBLUCSC | 284 | 7 | 28145 | tss | 0.028970 | 0.673477 | IRAK2 (margin: IRAK2) | 6.13 | 5.83e-04 | 6.47e-03 |
|  | 7 | 1607146 | 1607868 | PDFSTATSENSEMBLUCSC | 723 | 7 | 36762 | gene;island | 0.340174 | 0.960540 | PSMG3-AS1; PSMG3 (margin: PSMG3-AS1; TMEM184A; PSMG3) | 6.13 | 1.17e-03 | 1.13e-02 |
|  | 2 | 178937477 | 178937882 | PDFSTATSENSEMBLUCSC | 406 | 9 | 22944 | gene;island | 0.262155 | 0.011359 | PDE11A (margin: PDE11A) | 6.13 | 2.76e-03 | 1.78e-02 |
|  | 6 | 36355343 | 36355825 | PDFSTATSENSEMBLUCSC | 483 | 7 | 36081 | island | 0.129236 | 0.957947 | RP1-50J22.4; ETV7 (margin: RP1-50J22.4; ETV7; PXT1) | 6.13 | 2.33e-03 | 1.52e-02 |
|  | 6 | 29910755 | 29911558 | PDFSTATSENSEMBLUCSC | 804 | 22 | 33702 | gene;island | 0.052525 | 0.818446 | HLA-A; HCG4P5 (margin: HLA-U; HLA-A; HCG4P5) | 6.13 | 2.17e-08 | 5.43e-06 |
|  | 6 | 42947051 | 42947317 | PDFSTATSENSEMBLUCSC | 267 | 8 | 34986 | tss | 0.019655 | 0.617764 | PEX6 (margin: PPP2R5D; PEX6) | 6.13 | 1.55e-04 | 2.71e-03 |
|  | 1 | 6659205 | 6659504 | PDFSTATSENSEMBLUCSC | 300 | 5 | 181 | gene;island | 0.431185 | 0.978370 | KLHL21 (margin: ZBTB48; KLHL21) | 6.13 | 7.94e-03 | 3.19e-02 |
|  | 5 | 78985425 | 78985592 | PDFSTATSENSEMBLUCSC | 168 | 9 | 32147 | tss | 0.544246 | 0.021568 | CMYA5 (margin: PAPD4; CMYA5) | 6.12 | 4.11e-05 | 1.17e-03 |
|  | 15 | 52588020 | 52588279 | PDFSTATSENSEMBLUCSC | 260 | 6 | 13679 | tss | 0.449042 | 0.012765 | MYO5C (margin: MYO5C; MYO5A) | 6.12 | 2.16e-03 | 1.45e-02 |
|  | 17 | 21454528 | 21455116 | PDFSTATSENSEMBLUCSC | 589 | 6 | 18125 | island | 0.015745 | 0.460394 | RP11-822E23.3; C17orf51 (margin: RP11-822E23.2; RP11-822E23.3; C17orf51) | 6.12 | 2.16e-03 | 1.45e-02 |
|  | 7 | 28319343 | 28319676 | PDFSTATSENSEMBLUCSC | 334 | 6 | 37504 | tss | 0.105150 | 0.923947 | AC005017.2 (margin: AC005017.2) | 6.12 | 4.33e-03 | 2.52e-02 |
|  | 1 | 204120607 | 204120951 | PDFSTATSENSEMBLUCSC | 345 | 5 | 956 | gene;island | 0.863620 | 0.021578 | ETNK2 (margin: RP11-74C13.3; ETNK2; REN) | 6.12 | 7.94e-03 | 3.19e-02 |
|  | 4 | 7194841 | 7195206 | PDFSTATSENSEMBLUCSC | 366 | 5 | 29935 | gene;island | 0.002403 | 0.193058 | SORCS2 (margin: SORCS2) | 6.12 | 7.94e-03 | 3.19e-02 |
|  | 6 | 31744339 | 31744962 | PDFSTATSENSEMBLUCSC | 624 | 14 | 33954 | gene | 0.010125 | 0.615516 | VWA7; VARS; Y\_RNA (margin: MSH5; MSH5-SAPCD1; SAPCD1; SAPCD1-AS1; VWA7; VARS; Y\_RNA) | 6.11 | 7.94e-04 | 8.28e-03 |
|  | 14 | 89878562 | 89878733 | PDFSTATSENSEMBLUCSC | 172 | 6 | 12170 | gene;island | 0.163694 | 0.923881 | FOXN3; RP11-33N16.3 (margin: RP11-33N16.2; FOXN3-AS1; FOXN3; RP11-33N16.3) | 6.11 | 2.16e-03 | 1.45e-02 |
|  | 4 | 118006747 | 118006832 | PDFSTATSENSEMBLUCSC | 86 | 6 | 30559 | tss | 0.518175 | 0.022554 | TRAM1L1 (margin: TRAM1L1) | 6.10 | 2.16e-03 | 1.45e-02 |
|  | 12 | 16758692 | 16759029 | PDFSTATSENSEMBLUCSC | 338 | 6 | 9072 | gene | 0.637440 | 0.049020 | MGST1; LMO3 (margin: MGST1; LMO3) | 6.10 | 2.16e-03 | 1.45e-02 |
|  | 1 | 9884565 | 9884732 | PDFSTATSENSEMBLUCSC | 168 | 8 | 1230 | tss;island | 0.183542 | 0.007948 | CLSTN1 (margin: CLSTN1) | 6.09 | 1.55e-04 | 2.71e-03 |
|  | 5 | 142065671 | 142065819 | PDFSTATSENSEMBLUCSC | 149 | 5 | 32398 | tss | 0.031688 | 0.508175 | FGF1 (margin: FGF1) | 6.09 | 7.94e-03 | 3.19e-02 |
|  | 16 | 68482518 | 68483194 | PDFSTATSENSEMBLUCSC | 677 | 9 | 15322 | tss;island | 0.870023 | 0.039648 | SMPD3 (margin: SMPD3) | 6.09 | 8.23e-05 | 2.06e-03 |
|  | 17 | 41791038 | 41791475 | PDFSTATSENSEMBLUCSC | 438 | 5 | 18374 | island | 0.502205 | 0.014899 | (margin: CTC-501O10.1; RP11-209M4.1) | 6.09 | 7.94e-03 | 3.19e-02 |
|  | 6 | 111408745 | 111408765 | PDFSTATSENSEMBLUCSC | 21 | 5 | 35175 | tss | 0.467771 | 0.013681 | SLC16A10 (margin: SLC16A10; RNU6-960P) | 6.08 | 7.94e-03 | 3.19e-02 |
|  | 22 | 27053559 | 27053934 | PDFSTATSENSEMBLUCSC | 376 | 5 | 26826 | gene | 0.148714 | 0.988579 | MIAT (margin: MIAT; CTA-373H7.7) | 6.08 | 7.94e-03 | 3.19e-02 |
|  | 1 | 10056837 | 10057312 | PDFSTATSENSEMBLUCSC | 476 | 9 | 2662 | island | 0.474104 | 0.982024 | RBP7 (margin: NMNAT1; RBP7) | 6.08 | 4.11e-05 | 1.17e-03 |
|  | 13 | 72441404 | 72441711 | PDFSTATSENSEMBLUCSC | 308 | 5 | 11472 | tss;island | 0.666538 | 0.046586 | DACH1 (margin: DACH1) | 6.08 | 7.94e-03 | 3.19e-02 |
|  | 5 | 57878745 | 57878919 | PDFSTATSENSEMBLUCSC | 175 | 5 | 32078 | tss | 0.563468 | 0.027230 | RAB3C (margin: RAB3C) | 6.08 | 7.94e-03 | 3.19e-02 |
|  | 5 | 139927007 | 139927271 | PDFSTATSENSEMBLUCSC | 265 | 10 | 31785 | gene;island | 0.016379 | 0.474184 | ANKHD1; ANKHD1-EIF4EBP3; EIF4EBP3; SRA1 (margin: ANKHD1; ANKHD1-EIF4EBP3; EIF4EBP3; SRA1; APBB3) | 6.07 | 1.08e-05 | 4.82e-04 |
|  | 8 | 144660395 | 144660772 | PDFSTATSENSEMBLUCSC | 378 | 6 | 40673 | island | 0.546836 | 0.990304 | RP11-661A12.9; RP11-661A12.7; NAPRT1; EEF1D (margin: RP11-661A12.9; RP11-661A12.7; MROH6; NAPRT1; EEF1D) | 6.07 | 2.16e-03 | 1.45e-02 |
|  | 1 | 84326462 | 84326856 | PDFSTATSENSEMBLUCSC | 395 | 8 | 3406 | island | 0.506696 | 0.012575 | RP11-475O6.1 (margin: RP11-475O6.1; TTLL7) | 6.07 | 1.55e-04 | 2.71e-03 |
|  | 17 | 20811860 | 20812310 | PDFSTATSENSEMBLUCSC | 451 | 5 | 18113 | island | 0.011897 | 0.443634 | RP11-344E13.3 (margin: RP11-344E13.3; RNASEH1P1) | 6.07 | 7.94e-03 | 3.19e-02 |
|  | 6 | 152125861 | 152126441 | PDFSTATSENSEMBLUCSC | 581 | 8 | 34511 | gene;tss;island | 0.590549 | 0.991160 | ESR1 (margin: ESR1; RP3-443C4.2) | 6.07 | 1.55e-04 | 2.71e-03 |
|  | 6 | 26043546 | 26043990 | PDFSTATSENSEMBLUCSC | 445 | 5 | 35510 | island | 0.163920 | 0.936430 | U91328.2; HIST1H3C; HIST1H2BB (margin: U91328.2; HIST1H3C; HIST1H3B; HIST1H2AB; HIST1H2BB; HIST1H1C) | 6.06 | 7.94e-03 | 3.19e-02 |
|  | 10 | 54073047 | 54073642 | PDFSTATSENSEMBLUCSC | 596 | 8 | 5086 | tss;island | 0.356181 | 0.013192 | DKK1; PRKG1-AS1 (margin: DKK1; PRKG1-AS1) | 6.06 | 1.55e-04 | 2.71e-03 |
|  | 6 | 111408745 | 111408840 | PDFSTATSENSEMBLUCSC | 96 | 6 | 36418 | island | 0.470901 | 0.013862 | SLC16A10 (margin: SLC16A10; RNU6-960P) | 6.05 | 2.16e-03 | 1.45e-02 |
|  | 6 | 5085404 | 5085713 | PDFSTATSENSEMBLUCSC | 310 | 5 | 34608 | tss;island | 0.016225 | 0.530779 | PPP1R3G (margin: PPP1R3G) | 6.04 | 7.94e-03 | 3.19e-02 |
|  | 6 | 39016262 | 39016776 | PDFSTATSENSEMBLUCSC | 515 | 7 | 36105 | island | 0.471043 | 0.961741 | GLP1R (margin: GLP1R; RP1-202I21.3) | 6.04 | 2.33e-03 | 1.52e-02 |
|  | 17 | 6659164 | 6659562 | PDFSTATSENSEMBLUCSC | 399 | 7 | 16377 | gene | 0.182400 | 0.893293 | XAF1 (margin: RPL23AP73; XAF1; CTC-281F24.2) | 6.04 | 2.33e-03 | 1.52e-02 |
|  | 2 | 33359198 | 33359688 | PDFSTATSENSEMBLUCSC | 491 | 6 | 22641 | gene | 0.109078 | 0.918688 | LTBP1 (margin: LTBP1) | 6.04 | 2.16e-03 | 1.45e-02 |
|  | 1 | 115632236 | 115632727 | PDFSTATSENSEMBLUCSC | 492 | 8 | 1857 | tss | 0.013920 | 0.513893 | TSPAN2 (margin: RP4-666F24.3; TSPAN2) | 6.04 | 1.55e-04 | 2.71e-03 |
|  | 8 | 89340069 | 89340480 | PDFSTATSENSEMBLUCSC | 412 | 5 | 39802 | tss;island | 0.690120 | 0.068353 | RP11-586K2.1; MMP16 (margin: RP11-586K2.1; MMP16) | 6.04 | 7.94e-03 | 3.19e-02 |
|  | 11 | 35639492 | 35639686 | PDFSTATSENSEMBLUCSC | 195 | 6 | 7247 | tss;island | 0.018668 | 0.601576 | FJX1 (margin: FJX1) | 6.02 | 4.33e-03 | 2.52e-02 |
|  | 5 | 88179235 | 88179539 | PDFSTATSENSEMBLUCSC | 305 | 6 | 31682 | gene;island | 0.331203 | 0.009086 | MEF2C-AS1; MEF2C (margin: MEF2C-AS1; MEF2C) | 6.01 | 2.16e-03 | 1.45e-02 |
|  | 1 | 8002302 | 8002698 | PDFSTATSENSEMBLUCSC | 397 | 5 | 2629 | island | 0.848814 | 0.068610 | RP5-892F13.2; TNFRSF9 (margin: RP5-892F13.2; PARK7; TNFRSF9) | 6.01 | 7.94e-03 | 3.19e-02 |
|  | 6 | 88875236 | 88875651 | PDFSTATSENSEMBLUCSC | 416 | 5 | 34413 | gene | 0.506050 | 0.073838 | CNR1 (margin: CNR1) | 6.01 | 7.94e-03 | 3.19e-02 |
|  | 5 | 114938148 | 114938459 | PDFSTATSENSEMBLUCSC | 312 | 5 | 32228 | tss | 0.488307 | 0.980304 | AC010226.4; TICAM2; TMED7-TICAM2 (margin: AC010226.4; TICAM2; TMED7-TICAM2; TMED7) | 6.01 | 7.94e-03 | 3.19e-02 |
|  | 8 | 143535437 | 143535891 | PDFSTATSENSEMBLUCSC | 455 | 5 | 40634 | island | 0.384072 | 0.973533 | BAI1 (margin: BAI1) | 6.00 | 7.94e-03 | 3.19e-02 |
|  | 3 | 44753865 | 44754587 | PDFSTATSENSEMBLUCSC | 723 | 12 | 29040 | island | 0.395013 | 0.976294 | ZNF502; SOCS5P3 (margin: ZNF502; SOCS5P3) | 6.00 | 6.56e-04 | 7.02e-03 |
|  | 13 | 28493913 | 28494177 | PDFSTATSENSEMBLUCSC | 265 | 8 | 11633 | island | 0.392318 | 0.943998 | PDX1; PDX1-AS1 (margin: PDX1; PDX1-AS1) | 6.00 | 1.55e-04 | 2.71e-03 |
|  | 13 | 28493913 | 28494161 | PDFSTATSENSEMBLUCSC | 249 | 6 | 11365 | tss | 0.487884 | 0.943998 | PDX1; PDX1-AS1 (margin: PDX1; PDX1-AS1) | 6.00 | 2.16e-03 | 1.45e-02 |
|  | 6 | 33084420 | 33085063 | PDFSTATSENSEMBLUCSC | 644 | 20 | 34188 | gene;island | 0.090258 | 0.948950 | HLA-DPB2 (margin: HLA-DPB2; COL11A2P1) | 5.99 | 9.25e-07 | 8.75e-05 |
|  | 6 | 33095664 | 33096501 | PDFSTATSENSEMBLUCSC | 838 | 9 | 34192 | gene | 0.021820 | 0.596136 | HLA-DPB2 (margin: HLA-DPB2; HLA-DPA3) | 5.98 | 2.76e-03 | 1.78e-02 |
|  | 11 | 826837 | 827509 | PDFSTATSENSEMBLUCSC | 673 | 6 | 7031 | tss | 0.496828 | 0.018847 | PNPLA2; EFCAB4A; AP006621.8 (margin: PNPLA2; EFCAB4A; CD151; AP006621.8; POLR2L) | 5.98 | 2.16e-03 | 1.45e-02 |
|  | 4 | 155664941 | 155665249 | PDFSTATSENSEMBLUCSC | 309 | 7 | 31387 | island | 0.895922 | 0.095788 | LRAT (margin: LRAT; RP11-21G20.3) | 5.98 | 5.83e-04 | 6.47e-03 |
|  | 6 | 27342376 | 27342738 | PDFSTATSENSEMBLUCSC | 363 | 5 | 33619 | gene;island | 0.008442 | 0.349678 | ZNF391 (margin: ZNF391; ZNF204P) | 5.98 | 7.94e-03 | 3.19e-02 |
|  | 19 | 13915583 | 13915877 | PDFSTATSENSEMBLUCSC | 295 | 6 | 19883 | gene;island | 0.977923 | 0.437804 | ZSWIM4 (margin: ZSWIM4; CTD-3252C9.2; RN7SL619P) | 5.98 | 2.16e-03 | 1.45e-02 |
|  | 10 | 49893336 | 49893463 | PDFSTATSENSEMBLUCSC | 128 | 6 | 5069 | tss | 0.312916 | 0.932955 | WDFY4 (margin: WDFY4) | 5.98 | 2.16e-03 | 1.45e-02 |
|  | 7 | 4872926 | 4873549 | PDFSTATSENSEMBLUCSC | 624 | 6 | 36860 | gene;island | 0.267219 | 0.958084 | RADIL (margin: RADIL) | 5.98 | 2.16e-03 | 1.45e-02 |
|  | 3 | 36422059 | 36422406 | PDFSTATSENSEMBLUCSC | 348 | 7 | 28978 | island | 0.041633 | 0.619133 | STAC (margin: STAC) | 5.97 | 5.83e-04 | 6.47e-03 |
|  | 1 | 17445660 | 17446162 | PDFSTATSENSEMBLUCSC | 503 | 8 | 2757 | island | 0.436290 | 0.014641 | PADI2 (margin: PADI2) | 5.97 | 1.55e-04 | 2.71e-03 |
|  | 19 | 37663410 | 37663856 | PDFSTATSENSEMBLUCSC | 447 | 12 | 20734 | tss | 0.409742 | 0.010275 | CTC-454I21.3; ZNF585A (margin: CTC-454I21.3; ZNF585A; ZNF585B) | 5.97 | 7.40e-07 | 7.21e-05 |
|  | 6 | 24911204 | 24911615 | PDFSTATSENSEMBLUCSC | 412 | 10 | 34672 | tss | 0.662098 | 0.055435 | FAM65B (margin: FAM65B) | 5.96 | 2.17e-05 | 8.94e-04 |
|  | 1 | 248020632 | 248020812 | PDFSTATSENSEMBLUCSC | 181 | 5 | 1128 | gene | 0.471304 | 0.980558 | TRIM58 (margin: TRIM58; OR2W3) | 5.96 | 7.94e-03 | 3.19e-02 |
|  | 12 | 89746328 | 89746771 | PDFSTATSENSEMBLUCSC | 444 | 8 | 9906 | tss | 0.002736 | 0.199021 | DUSP6 (margin: DUSP6) | 5.95 | 1.55e-04 | 2.71e-03 |
|  | 14 | 92980026 | 92980204 | PDFSTATSENSEMBLUCSC | 179 | 5 | 13089 | island | 0.018268 | 0.513562 | RIN3 (margin: RIN3) | 5.95 | 7.94e-03 | 3.19e-02 |
|  | 10 | 29698152 | 29698470 | PDFSTATSENSEMBLUCSC | 319 | 8 | 5008 | tss;island | 0.008323 | 0.335828 | PTCHD3P1 (margin: PTCHD3P1) | 5.95 | 6.99e-03 | 3.19e-02 |
|  | 2 | 207139066 | 207139471 | PDFSTATSENSEMBLUCSC | 406 | 9 | 23835 | tss;island | 0.010014 | 0.425622 | ZDBF2 (margin: ZDBF2) | 5.94 | 4.11e-05 | 1.17e-03 |
|  | 3 | 113251979 | 113252350 | PDFSTATSENSEMBLUCSC | 372 | 5 | 27898 | gene;island | 0.472188 | 0.017230 | SIDT1 (margin: SIDT1) | 5.94 | 7.94e-03 | 3.19e-02 |
|  | 10 | 124894943 | 124895500 | PDFSTATSENSEMBLUCSC | 558 | 9 | 5415 | tss | 0.516571 | 0.986226 | HMX3 (margin: HMX3) | 5.94 | 1.65e-04 | 2.80e-03 |
|  | 2 | 121493807 | 121494043 | PDFSTATSENSEMBLUCSC | 237 | 5 | 24669 | island | 0.017600 | 0.451545 | GLI2 (margin: GLI2) | 5.94 | 7.94e-03 | 3.19e-02 |
|  | 11 | 79151719 | 79152112 | PDFSTATSENSEMBLUCSC | 394 | 7 | 7593 | tss | 0.630142 | 0.030519 | TENM4 (margin: TENM4) | 5.93 | 5.83e-04 | 6.47e-03 |
|  | 15 | 93199037 | 93199180 | PDFSTATSENSEMBLUCSC | 144 | 5 | 13912 | tss | 0.409325 | 0.015216 | FAM174B (margin: FAM174B) | 5.93 | 7.94e-03 | 3.19e-02 |
|  | 2 | 149634369 | 149634726 | PDFSTATSENSEMBLUCSC | 358 | 5 | 22894 | gene;island | 0.518422 | 0.017532 | KIF5C; AC105402.4 (margin: KIF5C; AC105402.4; AC105402.2; AC105402.3) | 5.93 | 7.94e-03 | 3.19e-02 |
|  | 2 | 182521874 | 182521926 | PDFSTATSENSEMBLUCSC | 53 | 6 | 23762 | tss;island | 0.459963 | 0.016813 | CERKL (margin: CERKL) | 5.93 | 2.16e-03 | 1.45e-02 |
|  | 2 | 136875897 | 136876188 | PDFSTATSENSEMBLUCSC | 292 | 7 | 23649 | tss;island | 0.589683 | 0.008708 | CXCR4 (margin: CXCR4) | 5.92 | 5.83e-04 | 6.47e-03 |
|  | 5 | 43037123 | 43037666 | PDFSTATSENSEMBLUCSC | 544 | 6 | 32766 | island | 0.509784 | 0.984734 | CTD-2201E18.3; ANXA2R (margin: CTD-2035E11.4; AC025171.1; CTD-2201E18.3; ANXA2R) | 5.92 | 2.16e-03 | 1.45e-02 |
|  | 4 | 10459128 | 10459435 | PDFSTATSENSEMBLUCSC | 308 | 9 | 30300 | tss;island | 0.209543 | 0.915958 | ZNF518B (margin: ZNF518B) | 5.92 | 4.11e-05 | 1.17e-03 |
|  | 4 | 53617076 | 53617448 | PDFSTATSENSEMBLUCSC | 373 | 5 | 31045 | island | 0.311470 | 0.740989 | ERVMER34-1; AC104066.1 (margin: SNORA26; ERVMER34-1; AC104066.1) | 5.92 | 7.94e-03 | 3.19e-02 |
|  | 1 | 26737005 | 26737470 | PDFSTATSENSEMBLUCSC | 466 | 8 | 2895 | island | 0.279963 | 0.974743 | LIN28A (margin: LIN28A) | 5.91 | 1.55e-04 | 2.71e-03 |
|  | 2 | 10830636 | 10830764 | PDFSTATSENSEMBLUCSC | 129 | 5 | 23183 | tss;island | 0.932419 | 0.050049 | RN7SL832P; NOL10 (margin: RN7SL832P; NOL10) | 5.91 | 7.94e-03 | 3.19e-02 |
|  | 17 | 2699021 | 2699718 | PDFSTATSENSEMBLUCSC | 698 | 10 | 17009 | tss | 0.308136 | 0.960232 | RAP1GAP2 (margin: RAP1GAP2) | 5.91 | 4.87e-04 | 6.47e-03 |
|  | 17 | 15244887 | 15245237 | PDFSTATSENSEMBLUCSC | 351 | 8 | 18040 | island | 0.435952 | 0.977951 | TEKT3 (margin: TEKT3) | 5.90 | 1.55e-04 | 2.71e-03 |
|  | 7 | 29519297 | 29519450 | PDFSTATSENSEMBLUCSC | 154 | 5 | 37511 | tss | 0.181840 | 0.927205 | CHN2 (margin: CHN2) | 5.90 | 7.94e-03 | 3.19e-02 |
|  | 13 | 93879303 | 93879769 | PDFSTATSENSEMBLUCSC | 467 | 5 | 11190 | gene;island | 0.912796 | 0.087961 | GPC6 (margin: GPC6) | 5.90 | 7.94e-03 | 3.19e-02 |
|  | 2 | 161126295 | 161126857 | PDFSTATSENSEMBLUCSC | 563 | 7 | 24814 | island | 0.013273 | 0.445791 | ITGB6; RBMS1 (margin: ITGB6; RBMS1) | 5.90 | 5.83e-04 | 6.47e-03 |
|  | 14 | 101525784 | 101526140 | PDFSTATSENSEMBLUCSC | 357 | 6 | 12630 | tss | 0.072769 | 0.882319 | MIR154; MIR496 (margin: MIR381HG; MIR889; MIR544A; MIR655; AL132709.3; MIR487A; MIR134; MIR485; MIR323B; MIR154; MIR496; MIR377; MIR541; MIR409; MIR412; MIR369; MIR410; MIR656; MEG9; MIR382) | 5.90 | 2.16e-03 | 1.45e-02 |
|  | 19 | 11998457 | 11999148 | PDFSTATSENSEMBLUCSC | 692 | 14 | 21586 | island | 0.170670 | 0.937151 | ZNF69 (margin: ZNF439; ZNF69) | 5.90 | 4.99e-08 | 9.34e-06 |
|  | 6 | 39290342 | 39290877 | PDFSTATSENSEMBLUCSC | 536 | 7 | 34956 | tss | 0.026917 | 0.733122 | KCNK16 (margin: KCNK17; KCNK16; KIF6) | 5.89 | 4.08e-03 | 2.47e-02 |
|  | 12 | 26986758 | 26987164 | PDFSTATSENSEMBLUCSC | 407 | 11 | 9615 | tss;island | 0.808322 | 0.098829 | ITPR2 (margin: ITPR2) | 5.89 | 5.53e-04 | 6.47e-03 |
|  | 1 | 870791 | 871057 | PDFSTATSENSEMBLUCSC | 267 | 5 | 2 | gene;island | 0.255959 | 0.973119 | SAMD11 (margin: SAMD11; AL645608.1; NOC2L) | 5.89 | 7.94e-03 | 3.19e-02 |
|  | 4 | 141347993 | 141348737 | PDFSTATSENSEMBLUCSC | 745 | 9 | 30138 | gene | 0.334080 | 0.006771 | CLGN (margin: CLGN) | 5.89 | 3.99e-03 | 2.47e-02 |
|  | 18 | 37421319 | 37421988 | PDFSTATSENSEMBLUCSC | 670 | 5 | 19479 | island | 0.628367 | 0.028092 | RP11-636O21.1 (margin: RP11-636O21.1) | 5.89 | 7.94e-03 | 3.19e-02 |
|  | 12 | 52545884 | 52546408 | PDFSTATSENSEMBLUCSC | 525 | 5 | 10436 | island | 0.028730 | 0.590420 | (margin: ) | 5.88 | 7.94e-03 | 3.19e-02 |
|  | 2 | 75426726 | 75427699 | PDFSTATSENSEMBLUCSC | 974 | 12 | 23468 | tss;island | 0.387107 | 0.013947 | TACR1 (margin: RP11-355F16.1; TACR1) | 5.88 | 7.40e-07 | 7.21e-05 |
|  | 22 | 44576869 | 44577240 | PDFSTATSENSEMBLUCSC | 372 | 8 | 27211 | tss | 0.226517 | 0.944394 | PARVG (margin: PARVB; PARVG) | 5.88 | 1.55e-04 | 2.71e-03 |
|  | 1 | 76262302 | 76262984 | PDFSTATSENSEMBLUCSC | 683 | 8 | 3390 | island | 0.384534 | 0.980276 | RABGGTB; MSH4 (margin: ACADM; RABGGTB; SNORD45C; SNORD45A; SNORD45B; MSH4) | 5.88 | 1.55e-04 | 2.71e-03 |
|  | 12 | 7260546 | 7260888 | PDFSTATSENSEMBLUCSC | 343 | 8 | 9044 | gene;tss | 0.019868 | 0.901568 | C1RL-AS1; C1RL (margin: C1RL-AS1; RNU6-485P; C1RL) | 5.88 | 1.55e-04 | 2.71e-03 |
|  | 2 | 113992762 | 113993313 | PDFSTATSENSEMBLUCSC | 552 | 8 | 22834 | gene;tss;island | 0.986977 | 0.563694 | AC016683.6; PAX8 (margin: AC016683.6; RP11-65I12.1; PAX8) | 5.88 | 1.55e-04 | 2.71e-03 |
|  | 7 | 93204046 | 93204478 | PDFSTATSENSEMBLUCSC | 433 | 9 | 37713 | tss;island | 0.777357 | 0.075143 | CALCR (margin: CALCR) | 5.87 | 4.11e-05 | 1.17e-03 |
|  | 3 | 62304470 | 62304779 | PDFSTATSENSEMBLUCSC | 310 | 5 | 28424 | tss;island | 0.943505 | 0.239458 | C3orf14; PTPRG-AS1 (margin: C3orf14; PTPRG-AS1) | 5.87 | 7.94e-03 | 3.19e-02 |
|  | 11 | 19736150 | 19736333 | PDFSTATSENSEMBLUCSC | 184 | 5 | 6535 | gene;tss;island | 0.292789 | 0.974156 | NAV2; RP11-359E10.1 (margin: NAV2; RP11-359E10.1) | 5.87 | 7.94e-03 | 3.19e-02 |
|  | 1 | 186649153 | 186649530 | PDFSTATSENSEMBLUCSC | 378 | 6 | 925 | gene | 0.157196 | 0.003652 | PTGS2 (margin: PTGS2) | 5.87 | 4.33e-03 | 2.52e-02 |
|  | 11 | 4629034 | 4629912 | PDFSTATSENSEMBLUCSC | 879 | 10 | 8007 | island | 0.017314 | 0.378816 | TRIM68 (margin: TRIM68) | 5.87 | 8.93e-03 | 3.45e-02 |
|  | 1 | 248020331 | 248020812 | PDFSTATSENSEMBLUCSC | 482 | 9 | 4421 | island | 0.465363 | 0.976431 | TRIM58 (margin: TRIM58; OR2W3) | 5.87 | 4.11e-05 | 1.17e-03 |
|  | 10 | 102289529 | 102289885 | PDFSTATSENSEMBLUCSC | 357 | 5 | 6027 | island | 0.013803 | 0.395162 | HIF1AN; SEC31B; NDUFB8 (margin: HIF1AN; SEC31B; NDUFB8) | 5.87 | 7.94e-03 | 3.19e-02 |
|  | 11 | 2884118 | 2884460 | PDFSTATSENSEMBLUCSC | 343 | 5 | 7975 | island | 0.549021 | 0.986063 | KCNQ1-AS1 (margin: KCNQ1DN; KCNQ1-AS1) | 5.87 | 7.94e-03 | 3.19e-02 |
|  | 6 | 29855401 | 29855636 | PDFSTATSENSEMBLUCSC | 236 | 9 | 33699 | gene | 0.008569 | 0.534975 | HLA-H; HCG4P7 (margin: HLA-H; HLA-T; HCG4P7) | 5.87 | 1.65e-04 | 2.80e-03 |
|  | 17 | 21279561 | 21279874 | PDFSTATSENSEMBLUCSC | 314 | 6 | 18118 | island | 0.547441 | 0.984027 | KCNJ12 (margin: KCNJ12) | 5.86 | 2.16e-03 | 1.45e-02 |
|  | 5 | 180018495 | 180018735 | PDFSTATSENSEMBLUCSC | 241 | 6 | 32560 | tss | 0.371413 | 0.968255 | SCGB3A1 (margin: AC122714.1; SCGB3A1; FLT4) | 5.86 | 2.16e-03 | 1.45e-02 |
|  | 7 | 39649224 | 39649443 | PDFSTATSENSEMBLUCSC | 220 | 5 | 36974 | gene;island | 0.364593 | 0.970401 | YAE1D1; AC004837.3; AC004837.4 (margin: YAE1D1; AC004837.3; AC004837.4) | 5.85 | 7.94e-03 | 3.19e-02 |
|  | 5 | 100238655 | 100239071 | PDFSTATSENSEMBLUCSC | 417 | 8 | 32984 | island | 0.113773 | 0.005165 | ST8SIA4 (margin: ST8SIA4) | 5.85 | 1.55e-04 | 2.71e-03 |
|  | 5 | 131592959 | 131593287 | PDFSTATSENSEMBLUCSC | 329 | 9 | 32268 | tss | 0.223401 | 0.950462 | PDLIM4; P4HA2 (margin: PDLIM4; P4HA2) | 5.85 | 4.94e-04 | 6.47e-03 |
|  | 6 | 30431563 | 30431860 | PDFSTATSENSEMBLUCSC | 298 | 15 | 35682 | island | 0.239452 | 0.945388 | (margin: TMPOP1; SUCLA2P1) | 5.85 | 1.29e-08 | 3.49e-06 |
|  | 2 | 197457393 | 197457430 | PDFSTATSENSEMBLUCSC | 38 | 5 | 23794 | tss | 0.570079 | 0.018206 | HECW2 (margin: HECW2) | 5.84 | 7.94e-03 | 3.19e-02 |
|  | 12 | 54422127 | 54422488 | PDFSTATSENSEMBLUCSC | 362 | 5 | 9159 | gene;island | 0.891134 | 0.332585 | HOXC5; HOXC6; HOXC4 (margin: HOXC5; HOXC6; HOXC4; AC012531.25; MIR615) | 5.84 | 7.94e-03 | 3.19e-02 |
|  | 2 | 179278332 | 179278855 | PDFSTATSENSEMBLUCSC | 524 | 6 | 22945 | gene;island | 0.013720 | 0.350643 | AC009948.5 (margin: RP11-65L3.1; AC009948.5) | 5.84 | 2.16e-03 | 1.45e-02 |
|  | 8 | 11059029 | 11059303 | PDFSTATSENSEMBLUCSC | 275 | 8 | 39549 | tss;island | 0.093767 | 0.703286 | AF131215.8; XKR6 (margin: AF131215.8; XKR6) | 5.84 | 6.99e-03 | 3.19e-02 |
|  | 3 | 96533511 | 96534005 | PDFSTATSENSEMBLUCSC | 495 | 6 | 27871 | gene;island | 0.920773 | 0.252637 | EPHA6 (margin: EPHA6) | 5.84 | 2.16e-03 | 1.45e-02 |
|  | 17 | 71161160 | 71161502 | PDFSTATSENSEMBLUCSC | 343 | 6 | 16745 | gene | 0.588200 | 0.020480 | RP11-143K11.5; SSTR2; POLR3KP2 (margin: RP11-143K11.5; SSTR2; RP11-143K11.1; POLR3KP2) | 5.83 | 2.16e-03 | 1.45e-02 |
|  | 11 | 10472001 | 10472161 | PDFSTATSENSEMBLUCSC | 161 | 7 | 7144 | tss;island | 0.026589 | 0.576345 | AMPD3 (margin: AMPD3) | 5.83 | 5.83e-04 | 6.47e-03 |
|  | 17 | 45772333 | 45772523 | PDFSTATSENSEMBLUCSC | 191 | 5 | 17443 | tss | 0.251027 | 0.972468 | TBKBP1 (margin: KPNB1; TBKBP1) | 5.83 | 7.94e-03 | 3.19e-02 |
|  | 13 | 36920644 | 36921174 | PDFSTATSENSEMBLUCSC | 531 | 13 | 11131 | gene;island | 0.117488 | 0.005981 | SPG20OS; SPG20 (margin: SPG20OS; AL139377.1; SPG20) | 5.83 | 1.91e-03 | 1.45e-02 |
|  | 7 | 8301971 | 8302301 | PDFSTATSENSEMBLUCSC | 331 | 8 | 37438 | tss;island | 0.657986 | 0.040590 | AC007009.1; AC007128.1; ICA1 (margin: AC007009.1; AC007128.1; ICA1) | 5.83 | 1.55e-04 | 2.71e-03 |
|  | 2 | 5836111 | 5836366 | PDFSTATSENSEMBLUCSC | 256 | 5 | 22558 | gene;island | 0.948911 | 0.287835 | SOX11; AC010729.1 (margin: SOX11; AC010729.1; AC107057.2; AC108025.2) | 5.83 | 7.94e-03 | 3.19e-02 |
|  | 10 | 124894943 | 124895886 | PDFSTATSENSEMBLUCSC | 944 | 13 | 6215 | island | 0.540449 | 0.984133 | HMX3 (margin: HMX3; HMX2) | 5.82 | 2.67e-05 | 1.07e-03 |
|  | 17 | 7197775 | 7198087 | PDFSTATSENSEMBLUCSC | 313 | 9 | 17933 | island | 0.691478 | 0.008007 | YBX2 (margin: SLC2A4; RP1-4G17.2; YBX2) | 5.82 | 1.23e-03 | 1.18e-02 |
|  | 2 | 27805536 | 27806117 | PDFSTATSENSEMBLUCSC | 582 | 11 | 24189 | island | 0.012305 | 0.379741 | C2orf16; ZNF512; RP11-158I13.2; AC074091.1 (margin: C2orf16; ZNF512; RP11-158I13.2; AC074091.1) | 5.82 | 3.94e-04 | 5.86e-03 |
|  | 6 | 83903419 | 83904091 | PDFSTATSENSEMBLUCSC | 673 | 8 | 34402 | gene;tss;island | 0.434121 | 0.011292 | RWDD2A; PGM3 (margin: RWDD2A; PGM3) | 5.82 | 1.04e-02 | 3.96e-02 |
|  | 11 | 65405268 | 65405525 | PDFSTATSENSEMBLUCSC | 258 | 9 | 7437 | tss | 0.016034 | 0.521291 | PCNXL3; MIR4690; SIPA1 (margin: PCNXL3; MIR4690; SIPA1; MIR4489) | 5.82 | 3.99e-03 | 2.47e-02 |
|  | 7 | 27187372 | 27187691 | PDFSTATSENSEMBLUCSC | 320 | 8 | 37493 | tss | 0.397123 | 0.977278 | HOXA-AS3; HOXA3; HOXA6 (margin: HOXA-AS3; RP1-170O19.21; HOXA3; HOXA5; HOXA6; HOXA7) | 5.81 | 1.55e-04 | 2.71e-03 |
|  | 3 | 44902933 | 44903730 | PDFSTATSENSEMBLUCSC | 798 | 13 | 29044 | island | 0.021293 | 0.490498 | KIF15; TMEM42; MIR564 (margin: KIF15; TMEM42; MIR564) | 5.81 | 1.92e-07 | 2.60e-05 |
|  | 4 | 155664941 | 155665106 | PDFSTATSENSEMBLUCSC | 166 | 6 | 30647 | tss | 0.885266 | 0.107460 | LRAT (margin: LRAT; RP11-21G20.3) | 5.80 | 2.16e-03 | 1.45e-02 |
|  | 2 | 131513608 | 131513927 | PDFSTATSENSEMBLUCSC | 320 | 6 | 22867 | gene;island | 0.470951 | 0.029929 | AMER3; AC140481.8 (margin: AMER3; AC140481.8) | 5.80 | 2.16e-03 | 1.45e-02 |
|  | 15 | 99644973 | 99645231 | PDFSTATSENSEMBLUCSC | 259 | 7 | 13923 | tss;island | 0.014917 | 0.375982 | SYNM; RP11-654A16.3 (margin: SYNM; RP11-654A16.3) | 5.80 | 5.83e-04 | 6.47e-03 |
|  | 11 | 65547778 | 65548028 | PDFSTATSENSEMBLUCSC | 251 | 7 | 8478 | island | 0.016321 | 0.567325 | AP5B1; AP001266.1 (margin: RP11-770G2.2; OVOL1; AP5B1; AP001266.1; RP11-770G2.4) | 5.80 | 5.83e-04 | 6.47e-03 |
|  | 6 | 110736772 | 110737053 | PDFSTATSENSEMBLUCSC | 282 | 5 | 35169 | tss | 0.120860 | 0.883547 | DDO (margin: DDO; SLC22A16) | 5.80 | 7.94e-03 | 3.19e-02 |
|  | 6 | 31759728 | 31760825 | PDFSTATSENSEMBLUCSC | 1098 | 20 | 33957 | gene;island | 0.190099 | 0.941294 | VARS (margin: VARS; LSM2) | 5.80 | 2.00e-06 | 1.82e-04 |
|  | 20 | 36793608 | 36794002 | PDFSTATSENSEMBLUCSC | 395 | 12 | 26152 | island | 0.682876 | 0.025361 | TGM2 (margin: TGM2) | 5.79 | 7.40e-07 | 7.21e-05 |
|  | 17 | 1958851 | 1959132 | PDFSTATSENSEMBLUCSC | 282 | 6 | 16327 | gene;tss;island | 0.208448 | 0.962238 | HIC1 (margin: HIC1; RP11-667K14.3; MIR132; MIR212; SMG6) | 5.79 | 2.16e-03 | 1.45e-02 |
|  | 7 | 112726396 | 112726869 | PDFSTATSENSEMBLUCSC | 474 | 10 | 37191 | gene;tss | 0.365289 | 0.955694 | GPR85 (margin: GPR85) | 5.79 | 2.17e-05 | 8.94e-04 |
|  | 1 | 239550177 | 239550578 | PDFSTATSENSEMBLUCSC | 402 | 6 | 4371 | island | 0.780983 | 0.100971 | CHRM3 (margin: CHRM3) | 5.79 | 2.16e-03 | 1.45e-02 |
|  | 4 | 148402399 | 148402899 | PDFSTATSENSEMBLUCSC | 501 | 5 | 30150 | gene;island | 0.499104 | 0.976841 | EDNRA (margin: EDNRA) | 5.78 | 7.94e-03 | 3.19e-02 |
|  | 21 | 46424715 | 46424976 | PDFSTATSENSEMBLUCSC | 262 | 6 | 26591 | tss;island | 0.024753 | 0.572840 | LINC00162 (margin: LINC00163; AP001505.9; LINC00162) | 5.78 | 2.16e-03 | 1.45e-02 |
|  | 11 | 79151611 | 79152112 | PDFSTATSENSEMBLUCSC | 502 | 8 | 8697 | island | 0.568076 | 0.033910 | TENM4 (margin: TENM4) | 5.77 | 3.11e-04 | 4.77e-03 |
|  | 14 | 104583632 | 104584279 | PDFSTATSENSEMBLUCSC | 648 | 6 | 13202 | island | 0.726304 | 0.035761 | MIR203 (margin: ASPG; MIR203) | 5.76 | 2.16e-03 | 1.45e-02 |
|  | 4 | 5712581 | 5712884 | PDFSTATSENSEMBLUCSC | 304 | 6 | 30276 | tss | 0.031829 | 0.630970 | EVC; EVC2 (margin: EVC; EVC2) | 5.76 | 4.33e-03 | 2.52e-02 |
|  | 17 | 29886672 | 29887049 | PDFSTATSENSEMBLUCSC | 378 | 5 | 18188 | island | 0.612420 | 0.029355 | MIR193A (margin: MIR193A; AC003101.1; RNU6ATAC7P) | 5.76 | 7.94e-03 | 3.19e-02 |
|  | 11 | 57519282 | 57519659 | PDFSTATSENSEMBLUCSC | 378 | 8 | 7310 | tss;island | 0.834741 | 0.076773 | TMX2-CTNND1; RP11-691N7.6; CTNND1; BTBD18 (margin: TMX2; TMX2-CTNND1; C11orf31; RP11-691N7.6; CTNND1; BTBD18) | 5.76 | 6.99e-03 | 3.19e-02 |
|  | 22 | 18593294 | 18593609 | PDFSTATSENSEMBLUCSC | 316 | 7 | 27284 | island | 0.513269 | 0.016596 | PEX26; TUBA8 (margin: PEX26; XXbac-B476C20.10; TUBA8; ARL2BPP10) | 5.75 | 5.83e-04 | 6.47e-03 |
|  | 18 | 47792708 | 47793175 | PDFSTATSENSEMBLUCSC | 468 | 8 | 19508 | island | 0.281794 | 0.019063 | CCDC11; MBD1 (margin: CCDC11; MBD1) | 5.75 | 1.55e-04 | 2.71e-03 |
|  | 10 | 35930436 | 35930534 | PDFSTATSENSEMBLUCSC | 99 | 6 | 5029 | tss;island | 0.364800 | 0.008761 | FZD8; MIR4683 (margin: FZD8; MIR4683) | 5.74 | 2.16e-03 | 1.45e-02 |
|  | 13 | 97646639 | 97646811 | PDFSTATSENSEMBLUCSC | 173 | 7 | 11506 | tss;island | 0.340892 | 0.013738 | OXGR1 (margin: LINC00359; OXGR1) | 5.74 | 5.83e-04 | 6.47e-03 |
|  | 7 | 31232532 | 31232861 | PDFSTATSENSEMBLUCSC | 330 | 6 | 38391 | island | 0.469134 | 0.008210 | (margin: ) | 5.74 | 2.16e-03 | 1.45e-02 |
|  | 22 | 44576869 | 44577265 | PDFSTATSENSEMBLUCSC | 397 | 9 | 26891 | gene | 0.246176 | 0.945574 | PARVG (margin: PARVB; PARVG) | 5.74 | 1.65e-04 | 2.80e-03 |
|  | 6 | 151646540 | 151647133 | PDFSTATSENSEMBLUCSC | 594 | 9 | 34509 | gene;island | 0.483203 | 0.020852 | AKAP12 (margin: AKAP12; RN7SKP268) | 5.74 | 2.76e-03 | 1.78e-02 |
|  | 2 | 119532041 | 119532574 | PDFSTATSENSEMBLUCSC | 534 | 7 | 24643 | island | 0.008394 | 0.296952 | (margin: ) | 5.73 | 1.17e-03 | 1.13e-02 |
|  | 16 | 433439 | 433825 | PDFSTATSENSEMBLUCSC | 387 | 5 | 14530 | gene;island | 0.563754 | 0.023778 | Z97634.5; TMEM8A (margin: Z97634.5; Z97634.3; TMEM8A) | 5.73 | 7.94e-03 | 3.19e-02 |
|  | 4 | 110223830 | 110224288 | PDFSTATSENSEMBLUCSC | 459 | 10 | 30542 | tss | 0.765990 | 0.059767 | AC004051.2; COL25A1 (margin: AC004051.2; COL25A1) | 5.72 | 1.50e-03 | 1.41e-02 |
|  | 6 | 108487063 | 108487183 | PDFSTATSENSEMBLUCSC | 121 | 6 | 35158 | tss;island | 0.381778 | 0.969383 | NR2E1; OSTM1 (margin: OSTM1-AS1; NR2E1; OSTM1) | 5.72 | 2.16e-03 | 1.45e-02 |
|  | 20 | 43029468 | 43029895 | PDFSTATSENSEMBLUCSC | 428 | 5 | 25759 | tss | 0.534753 | 0.889968 | HNF4A (margin: HNF4A; MIR3646; RP5-881L22.4; RP5-881L22.6) | 5.72 | 7.94e-03 | 3.19e-02 |
|  | 2 | 111875749 | 111876008 | PDFSTATSENSEMBLUCSC | 260 | 5 | 24611 | island | 0.242861 | 0.947520 | ACOXL; BCL2L11 (margin: ACOXL; BCL2L11; AC096670.3) | 5.72 | 7.94e-03 | 3.19e-02 |
|  | 1 | 49242359 | 49242934 | PDFSTATSENSEMBLUCSC | 576 | 8 | 520 | gene;island | 0.786971 | 0.053277 | AGBL4; BEND5 (margin: AGBL4; BEND5) | 5.72 | 2.95e-03 | 1.88e-02 |
|  | 17 | 80797881 | 80798078 | PDFSTATSENSEMBLUCSC | 198 | 8 | 16959 | gene | 0.368091 | 0.012338 | TBCD; ZNF750 (margin: TBCD; ZNF750) | 5.72 | 1.55e-04 | 2.71e-03 |
|  | 11 | 67806337 | 67806668 | PDFSTATSENSEMBLUCSC | 332 | 8 | 8564 | island | 0.009860 | 0.412339 | TCIRG1 (margin: ALDH3B1; NDUFS8; MIR4691; TCIRG1; RP11-802E16.3; RP5-901A4.1) | 5.71 | 1.55e-04 | 2.71e-03 |
|  | 11 | 67806337 | 67806437 | PDFSTATSENSEMBLUCSC | 101 | 6 | 7505 | tss | 0.009860 | 0.373438 | TCIRG1 (margin: ALDH3B1; NDUFS8; MIR4691; TCIRG1; RP11-802E16.3; RP5-901A4.1) | 5.71 | 2.16e-03 | 1.45e-02 |
|  | 7 | 150549175 | 150549560 | PDFSTATSENSEMBLUCSC | 386 | 5 | 37983 | tss | 0.039005 | 0.586068 | AOC1 (margin: AOC1) | 5.71 | 7.94e-03 | 3.19e-02 |
|  | 10 | 119304379 | 119304719 | PDFSTATSENSEMBLUCSC | 341 | 7 | 4786 | gene;island | 0.458746 | 0.984314 | EMX2; EMX2OS (margin: EMX2; EMX2OS) | 5.70 | 5.83e-04 | 6.47e-03 |
|  | 15 | 60296138 | 60296469 | PDFSTATSENSEMBLUCSC | 332 | 6 | 14189 | island | 0.621428 | 0.027204 | FOXB1 (margin: FOXB1) | 5.70 | 2.16e-03 | 1.45e-02 |
|  | 6 | 100062488 | 100062855 | PDFSTATSENSEMBLUCSC | 368 | 5 | 34430 | gene;island | 0.356666 | 0.960362 | PRDM13 (margin: PRDM13) | 5.69 | 7.94e-03 | 3.19e-02 |
|  | 11 | 1037640 | 1037923 | PDFSTATSENSEMBLUCSC | 284 | 5 | 7037 | tss;island | 0.665481 | 0.976247 | MUC6 (margin: RP13-870H17.3; MUC6) | 5.69 | 7.94e-03 | 3.19e-02 |
|  | 19 | 12476027 | 12476577 | PDFSTATSENSEMBLUCSC | 551 | 8 | 21600 | island | 0.017804 | 0.437119 | ZNF442 (margin: CTD-3105H18.5; ZNF442) | 5.69 | 1.04e-02 | 3.96e-02 |
|  | 8 | 41166990 | 41167278 | PDFSTATSENSEMBLUCSC | 289 | 6 | 39667 | tss;island | 0.599309 | 0.986692 | SFRP1 (margin: RNU6-895P; SFRP1) | 5.69 | 2.16e-03 | 1.45e-02 |
|  | 13 | 113676726 | 113677325 | PDFSTATSENSEMBLUCSC | 600 | 6 | 11269 | gene;island | 0.331854 | 0.961549 | MCF2L (margin: MCF2L) | 5.68 | 2.16e-03 | 1.45e-02 |
|  | 1 | 60539362 | 60539675 | PDFSTATSENSEMBLUCSC | 314 | 6 | 3327 | island | 0.636488 | 0.982084 | C1orf87 (margin: C1orf87) | 5.68 | 4.33e-03 | 2.52e-02 |
|  | 6 | 149806235 | 149806732 | PDFSTATSENSEMBLUCSC | 498 | 7 | 35283 | tss | 0.163840 | 0.906720 | ZC3H12D (margin: ZC3H12D; RP1-281H8.3) | 5.68 | 1.11e-02 | 4.14e-02 |
|  | 7 | 149570987 | 149571373 | PDFSTATSENSEMBLUCSC | 387 | 11 | 37973 | tss | 0.503624 | 0.014069 | ATP6V0E2; ATP6V0E2-AS1 (margin: ZNF862; ATP6V0E2; ATP6V0E2-AS1) | 5.67 | 2.84e-06 | 1.96e-04 |
|  | 15 | 48470205 | 48470973 | PDFSTATSENSEMBLUCSC | 769 | 10 | 14119 | island | 0.926747 | 0.025624 | MYEF2 (margin: MYEF2; RP11-605F22.2) | 5.67 | 3.89e-03 | 2.43e-02 |
|  | 14 | 55034288 | 55034666 | PDFSTATSENSEMBLUCSC | 379 | 8 | 12859 | island | 0.004207 | 0.230822 | SAMD4A (margin: SAMD4A) | 5.67 | 1.55e-04 | 2.71e-03 |
|  | 5 | 142065539 | 142065819 | PDFSTATSENSEMBLUCSC | 281 | 6 | 31843 | gene | 0.036642 | 0.577866 | FGF1 (margin: FGF1) | 5.67 | 2.16e-03 | 1.45e-02 |
|  | 19 | 19976569 | 19976679 | PDFSTATSENSEMBLUCSC | 111 | 6 | 20654 | tss | 0.684678 | 0.020332 | ZNF253 (margin: CTC-559E9.12; ZNF253; CTC-559E9.10) | 5.67 | 2.16e-03 | 1.45e-02 |
|  | 2 | 176971820 | 176972068 | PDFSTATSENSEMBLUCSC | 249 | 7 | 23734 | tss | 0.017044 | 0.384279 | HOXD11; AC009336.1; HOXD10 (margin: HOXD13; HOXD12; HOXD11; AC009336.1; HOXD10) | 5.67 | 5.83e-04 | 6.47e-03 |
|  | 6 | 166401464 | 166401700 | PDFSTATSENSEMBLUCSC | 237 | 6 | 34542 | gene;island | 0.294165 | 0.003217 | PDE10A; LINC00473 (margin: PDE10A; LINC00473) | 5.66 | 2.16e-03 | 1.45e-02 |
|  | 1 | 201476290 | 201476371 | PDFSTATSENSEMBLUCSC | 82 | 5 | 939 | gene | 0.017048 | 0.405215 | RP11-134G8.7; CSRP1 (margin: RP11-134G8.7; RPS10P7; CSRP1) | 5.66 | 7.94e-03 | 3.19e-02 |
|  | 1 | 26737005 | 26737262 | PDFSTATSENSEMBLUCSC | 258 | 5 | 1392 | tss | 0.182421 | 0.972274 | LIN28A (margin: LIN28A) | 5.66 | 7.94e-03 | 3.19e-02 |
|  | 17 | 54671073 | 54671575 | PDFSTATSENSEMBLUCSC | 503 | 5 | 16702 | gene | 0.496751 | 0.018319 | NOG (margin: NOG) | 5.66 | 7.94e-03 | 3.19e-02 |
|  | 17 | 33416834 | 33417113 | PDFSTATSENSEMBLUCSC | 280 | 5 | 17264 | tss;island | 0.056238 | 0.841096 | RFFL; RAD51L3-RFFL (margin: RNU6-840P; RFFL; RAD51L3-RFFL; Vault; RAD51D) | 5.66 | 7.94e-03 | 3.19e-02 |
|  | 19 | 40324746 | 40325128 | PDFSTATSENSEMBLUCSC | 383 | 11 | 22006 | island | 0.437254 | 0.010936 | DYRK1B; FBL (margin: DYRK1B; FBL) | 5.65 | 1.04e-02 | 3.96e-02 |
|  | 5 | 14405632 | 14406021 | PDFSTATSENSEMBLUCSC | 390 | 5 | 31563 | gene | 0.545394 | 0.985757 | TRIO (margin: TRIO) | 5.65 | 7.94e-03 | 3.19e-02 |
|  | 4 | 57976556 | 57976944 | PDFSTATSENSEMBLUCSC | 389 | 8 | 30416 | tss | 0.146426 | 0.649772 | RP11-12A1.1; IGFBP7 (margin: RP11-12A1.1; IGFBP7; snoU13) | 5.64 | 1.55e-04 | 2.71e-03 |
|  | 19 | 51522454 | 51522921 | PDFSTATSENSEMBLUCSC | 468 | 7 | 20188 | gene;island | 0.013525 | 0.205710 | KLK10 (margin: CTB-147C22.9; CTC-518B2.12; CTC-518B2.9; KLK9; KLK10; KLK11; KLK12) | 5.64 | 6.99e-03 | 3.19e-02 |
|  | 5 | 128300967 | 128301488 | PDFSTATSENSEMBLUCSC | 522 | 10 | 33072 | island | 0.358096 | 0.012953 | SLC27A6 (margin: SLC27A6) | 5.64 | 3.25e-04 | 4.91e-03 |
|  | 6 | 43211208 | 43211491 | PDFSTATSENSEMBLUCSC | 284 | 5 | 36158 | island | 0.800057 | 0.988217 | TTBK1 (margin: TTBK1) | 5.64 | 7.94e-03 | 3.19e-02 |
|  | 3 | 9993731 | 9994061 | PDFSTATSENSEMBLUCSC | 331 | 5 | 27657 | gene | 0.415501 | 0.008481 | PRRT3-AS1; PRRT3 (margin: CRELD1; PRRT3-AS1; RP11-1020A11.2; PRRT3; EMC3) | 5.64 | 7.94e-03 | 3.19e-02 |
|  | 16 | 10970801 | 10971250 | PDFSTATSENSEMBLUCSC | 450 | 5 | 15697 | island | 0.017922 | 0.413516 | CIITA; RP11-876N24.2 (margin: CIITA; RP11-876N24.2) | 5.64 | 7.94e-03 | 3.19e-02 |
|  | 1 | 25943790 | 25943891 | PDFSTATSENSEMBLUCSC | 102 | 5 | 1373 | tss;island | 0.491869 | 0.016764 | MAN1C1 (margin: MAN1C1) | 5.64 | 7.94e-03 | 3.19e-02 |
|  | 17 | 40715222 | 40715281 | PDFSTATSENSEMBLUCSC | 60 | 5 | 16580 | gene;island | 0.021112 | 0.518758 | RP11-400F19.8; COASY (margin: RP11-400F19.8; HSD17B1; COASY; MLX; RP11-400F19.6; PSMC3IP) | 5.63 | 7.94e-03 | 3.19e-02 |
|  | 21 | 43638893 | 43639122 | PDFSTATSENSEMBLUCSC | 230 | 9 | 26437 | gene;tss;island | 0.475864 | 0.017986 | ABCG1 (margin: ABCG1; RNA5SP492) | 5.63 | 1.65e-04 | 2.80e-03 |
|  | 10 | 134859687 | 134860210 | PDFSTATSENSEMBLUCSC | 524 | 7 | 6329 | island | 0.312062 | 0.831134 | (margin: ) | 5.63 | 2.33e-03 | 1.52e-02 |
|  | 5 | 150284302 | 150284796 | PDFSTATSENSEMBLUCSC | 495 | 9 | 33313 | island | 0.292191 | 0.900417 | ZNF300 (margin: IRGM; ZNF300) | 5.62 | 1.65e-04 | 2.80e-03 |
|  | 15 | 41952235 | 41952827 | PDFSTATSENSEMBLUCSC | 593 | 8 | 14066 | island | 0.523476 | 0.019538 | MGA (margin: MGA) | 5.62 | 1.04e-02 | 3.96e-02 |
|  | 13 | 113698408 | 113698688 | PDFSTATSENSEMBLUCSC | 281 | 6 | 11270 | gene;island | 0.172284 | 0.822952 | MCF2L (margin: MCF2L) | 5.62 | 8.66e-03 | 3.36e-02 |
|  | 5 | 150284416 | 150284796 | PDFSTATSENSEMBLUCSC | 381 | 8 | 32435 | tss | 0.332093 | 0.924752 | ZNF300 (margin: IRGM; ZNF300) | 5.61 | 6.22e-04 | 6.71e-03 |
|  | 7 | 50800837 | 50801423 | PDFSTATSENSEMBLUCSC | 587 | 8 | 37014 | gene;tss | 0.158946 | 0.937695 | GRB10 (margin: GRB10) | 5.61 | 4.66e-03 | 2.68e-02 |
|  | 5 | 175792454 | 175792510 | PDFSTATSENSEMBLUCSC | 57 | 5 | 33425 | island | 0.530436 | 0.985608 | ARL10 (margin: RP11-843P14.2; RP11-843P14.1; ARL10; MIR1271; KIAA1191) | 5.60 | 7.94e-03 | 3.19e-02 |
|  | 10 | 103603563 | 103604019 | PDFSTATSENSEMBLUCSC | 457 | 7 | 6074 | island | 0.940615 | 0.215024 | KCNIP2; C10orf76 (margin: KCNIP2; C10orf76) | 5.60 | 5.83e-04 | 6.47e-03 |
|  | 19 | 7767075 | 7767348 | PDFSTATSENSEMBLUCSC | 274 | 5 | 20405 | tss | 0.311366 | 0.917696 | FCER2 (margin: FCER2) | 5.60 | 7.94e-03 | 3.19e-02 |
|  | 11 | 101000566 | 101000717 | PDFSTATSENSEMBLUCSC | 152 | 5 | 7642 | tss | 0.444469 | 0.015088 | PGR (margin: PGR) | 5.60 | 7.94e-03 | 3.19e-02 |
|  | 6 | 50813551 | 50814089 | PDFSTATSENSEMBLUCSC | 539 | 5 | 34368 | gene;island | 0.206636 | 0.986261 | TFAP2B (margin: TFAP2B; RPS17P5) | 5.59 | 7.94e-03 | 3.19e-02 |
|  | 4 | 154125208 | 154125594 | PDFSTATSENSEMBLUCSC | 387 | 6 | 30640 | tss | 0.878636 | 0.276944 | TRIM2 (margin: TRIM2) | 5.59 | 4.33e-03 | 2.52e-02 |
|  | 20 | 30457834 | 30458267 | PDFSTATSENSEMBLUCSC | 434 | 9 | 25384 | gene;tss;island | 0.522422 | 0.020716 | TTLL9; DUSP15 (margin: TTLL9; DUSP15) | 5.59 | 4.11e-05 | 1.17e-03 |
|  | 15 | 86313834 | 86313973 | PDFSTATSENSEMBLUCSC | 140 | 7 | 13463 | gene;tss;island | 0.452417 | 0.978538 | RP11-158M2.6; KLHL25; MIR1276 (margin: RP11-158M2.6; KLHL25; MIR1276) | 5.59 | 5.83e-04 | 6.47e-03 |
|  | 6 | 118973021 | 118973353 | PDFSTATSENSEMBLUCSC | 333 | 8 | 35200 | tss | 0.529956 | 0.034077 | CEP85L (margin: CEP85L) | 5.59 | 6.99e-03 | 3.19e-02 |
|  | 8 | 144640306 | 144640403 | PDFSTATSENSEMBLUCSC | 98 | 6 | 39952 | tss | 0.032462 | 0.611651 | GSDMD (margin: GSDMD; RP11-661A12.5; MROH6) | 5.58 | 2.16e-03 | 1.45e-02 |
|  | 6 | 32410690 | 32411185 | PDFSTATSENSEMBLUCSC | 496 | 6 | 34114 | gene | 0.031762 | 0.604327 | HLA-DRA (margin: HLA-DRA) | 5.58 | 2.16e-03 | 1.45e-02 |
|  | 6 | 33140660 | 33141305 | PDFSTATSENSEMBLUCSC | 646 | 9 | 34201 | gene | 0.029186 | 0.611682 | COL11A2 (margin: COL11A2) | 5.58 | 1.23e-03 | 1.18e-02 |
|  | 20 | 1206682 | 1206830 | PDFSTATSENSEMBLUCSC | 149 | 8 | 25934 | island | 0.232500 | 0.006555 | RAD21L1 (margin: RAD21L1) | 5.58 | 1.09e-03 | 1.10e-02 |
|  | 11 | 2019436 | 2020560 | PDFSTATSENSEMBLUCSC | 1125 | 36 | 7055 | tss;island | 0.601164 | 0.031832 | H19 (margin: AC051649.6; MRPL23-AS1; H19) | 5.57 | 4.70e-13 | 9.15e-10 |
|  | 12 | 85673860 | 85674022 | PDFSTATSENSEMBLUCSC | 163 | 6 | 9901 | tss | 0.561979 | 0.983125 | ALX1 (margin: ALX1) | 5.57 | 2.16e-03 | 1.45e-02 |
|  | 4 | 183061830 | 183062004 | PDFSTATSENSEMBLUCSC | 175 | 5 | 30188 | gene;island | 0.609397 | 0.981871 | AC108142.1 (margin: TENM3; RP11-402C9.1; AC108142.1) | 5.57 | 7.94e-03 | 3.19e-02 |
|  | 19 | 37064219 | 37064886 | PDFSTATSENSEMBLUCSC | 668 | 10 | 20727 | tss | 0.014667 | 0.410684 | AC092295.7; ZNF529 (margin: AC092295.7; ZNF529) | 5.57 | 1.08e-05 | 4.82e-04 |
|  | 7 | 94284258 | 94285004 | PDFSTATSENSEMBLUCSC | 747 | 27 | 37092 | gene;tss;island | 0.810916 | 0.082725 | PEG10; SGCE (margin: PEG10; SGCE) | 5.56 | 2.94e-11 | 2.60e-08 |
|  | 7 | 29519297 | 29519656 | PDFSTATSENSEMBLUCSC | 360 | 8 | 36949 | gene | 0.231188 | 0.907870 | CHN2 (margin: CHN2) | 5.56 | 3.11e-04 | 4.77e-03 |
|  | 4 | 41867415 | 41867562 | PDFSTATSENSEMBLUCSC | 148 | 5 | 31005 | island | 0.455622 | 0.985565 | (margin: LINC00682) | 5.56 | 7.94e-03 | 3.19e-02 |
|  | 10 | 180188 | 180755 | PDFSTATSENSEMBLUCSC | 568 | 12 | 5468 | island | 0.038165 | 0.003573 | ZMYND11 (margin: ZMYND11) | 5.56 | 8.29e-03 | 3.33e-02 |
|  | 7 | 24797102 | 24797884 | PDFSTATSENSEMBLUCSC | 783 | 11 | 37478 | tss | 0.392099 | 0.967413 | DFNA5 (margin: DFNA5) | 5.55 | 8.33e-03 | 3.34e-02 |
|  | 10 | 131265059 | 131265796 | PDFSTATSENSEMBLUCSC | 738 | 15 | 6258 | island | 0.406370 | 0.007612 | MGMT (margin: MGMT) | 5.55 | 4.40e-05 | 1.23e-03 |
|  | 19 | 9545862 | 9546377 | PDFSTATSENSEMBLUCSC | 516 | 12 | 21498 | island | 0.013176 | 0.385001 | ZNF266 (margin: ZNF266) | 5.55 | 7.40e-07 | 7.21e-05 |
|  | 19 | 9546252 | 9546377 | PDFSTATSENSEMBLUCSC | 126 | 8 | 20424 | tss | 0.013176 | 0.385001 | ZNF266 (margin: ZNF266) | 5.55 | 1.55e-04 | 2.71e-03 |
|  | 17 | 16256937 | 16257223 | PDFSTATSENSEMBLUCSC | 287 | 9 | 17156 | tss;island | 0.171990 | 0.936737 | CENPV (margin: PIGL; CENPV) | 5.55 | 8.23e-05 | 2.06e-03 |
|  | 3 | 170746029 | 170746265 | PDFSTATSENSEMBLUCSC | 237 | 5 | 28717 | tss | 0.330673 | 0.974284 | SLC2A2 (margin: SLC2A2) | 5.54 | 7.94e-03 | 3.19e-02 |
|  | 7 | 101005832 | 101006089 | PDFSTATSENSEMBLUCSC | 258 | 9 | 37802 | tss;island | 0.583787 | 0.988591 | COL26A1 (margin: COL26A1) | 5.53 | 2.88e-04 | 4.58e-03 |
|  | 3 | 170746029 | 170746291 | PDFSTATSENSEMBLUCSC | 263 | 6 | 29661 | island | 0.379421 | 0.972852 | SLC2A2 (margin: SLC2A2) | 5.53 | 2.16e-03 | 1.45e-02 |
|  | 1 | 3044741 | 3045166 | PDFSTATSENSEMBLUCSC | 426 | 6 | 101 | gene | 0.308099 | 0.968146 | PRDM16 (margin: PRDM16; RP1-163G9.2) | 5.53 | 2.16e-03 | 1.45e-02 |
|  | 11 | 65547881 | 65548028 | PDFSTATSENSEMBLUCSC | 148 | 6 | 7440 | tss | 0.018850 | 0.531819 | AP5B1; AP001266.1 (margin: RP11-770G2.2; OVOL1; AP5B1; AP001266.1; RP11-770G2.4) | 5.52 | 2.16e-03 | 1.45e-02 |
|  | 10 | 86001154 | 86001418 | PDFSTATSENSEMBLUCSC | 265 | 6 | 5896 | island | 0.080456 | 0.797022 | LRIT1 (margin: RGR; LRIT1) | 5.52 | 8.66e-03 | 3.36e-02 |
|  | 10 | 135090098 | 135090565 | PDFSTATSENSEMBLUCSC | 468 | 5 | 6350 | island | 0.321387 | 0.958079 | ADAM8 (margin: ADAM8; TUBGCP2) | 5.52 | 7.94e-03 | 3.19e-02 |
|  | 14 | 36003074 | 36003528 | PDFSTATSENSEMBLUCSC | 455 | 10 | 12781 | island | 0.452536 | 0.013810 | INSM2 (margin: INSM2; RALGAPA1) | 5.51 | 1.08e-05 | 4.82e-04 |
|  | 16 | 82044738 | 82045078 | PDFSTATSENSEMBLUCSC | 341 | 5 | 14857 | gene | 0.034422 | 0.651977 | SDR42E1 (margin: SDR42E1) | 5.51 | 7.94e-03 | 3.19e-02 |
|  | 11 | 69468789 | 69469403 | PDFSTATSENSEMBLUCSC | 615 | 9 | 8593 | island | 0.895978 | 0.303387 | CCND1; ORAOV1 (margin: CCND1; ORAOV1) | 5.51 | 8.23e-05 | 2.06e-03 |
|  | 7 | 149570931 | 149571373 | PDFSTATSENSEMBLUCSC | 443 | 12 | 37269 | gene;island | 0.503133 | 0.017461 | ATP6V0E2; ATP6V0E2-AS1 (margin: ZNF862; ATP6V0E2; ATP6V0E2-AS1) | 5.51 | 7.40e-07 | 7.21e-05 |
|  | 18 | 60987770 | 60988155 | PDFSTATSENSEMBLUCSC | 386 | 11 | 19554 | island | 0.202685 | 0.008352 | BCL2 (margin: BCL2; RP11-28F1.2; KDSR) | 5.50 | 6.63e-03 | 3.19e-02 |
|  | 2 | 152955498 | 152955783 | PDFSTATSENSEMBLUCSC | 286 | 5 | 23662 | tss;island | 0.541982 | 0.025987 | AC079790.2; CACNB4 (margin: AC079790.2; CACNB4) | 5.50 | 7.94e-03 | 3.19e-02 |
|  | 7 | 156802150 | 156802502 | PDFSTATSENSEMBLUCSC | 353 | 7 | 37317 | gene;tss;island | 0.671111 | 0.036629 | RP5-1121A15.1; MNX1 (margin: RP5-1121A15.4; RP5-1121A15.1; MNX1) | 5.50 | 5.83e-04 | 6.47e-03 |
|  | 14 | 69951211 | 69951495 | PDFSTATSENSEMBLUCSC | 285 | 7 | 12967 | island | 0.527078 | 0.018484 | PLEKHD1 (margin: PLEKHD1) | 5.50 | 5.83e-04 | 6.47e-03 |
|  | 15 | 83378637 | 83378895 | PDFSTATSENSEMBLUCSC | 259 | 8 | 13859 | tss | 0.136691 | 0.006341 | AP3B2; AC105339.1 (margin: AP3B2; AC105339.1) | 5.49 | 6.22e-04 | 6.71e-03 |
|  | 17 | 72889708 | 72890001 | PDFSTATSENSEMBLUCSC | 294 | 8 | 17634 | tss | 0.468382 | 0.954638 | FADS6 (margin: FADS6) | 5.49 | 6.22e-04 | 6.71e-03 |
|  | 20 | 55841869 | 55842071 | PDFSTATSENSEMBLUCSC | 203 | 8 | 25840 | tss;island | 0.863226 | 0.075758 | RP4-813D12.3; BMP7 (margin: RP4-813D12.3; BMP7) | 5.49 | 3.11e-04 | 4.77e-03 |
|  | 8 | 19540210 | 19540734 | PDFSTATSENSEMBLUCSC | 525 | 8 | 39572 | tss | 0.137751 | 0.892700 | RP11-1105O14.1; CSGALNACT1 (margin: RP11-1105O14.1; CSGALNACT1) | 5.49 | 6.99e-03 | 3.19e-02 |
|  | 10 | 18549378 | 18549778 | PDFSTATSENSEMBLUCSC | 401 | 7 | 4521 | gene | 0.486116 | 0.963709 | CACNB2; RP11-109I13.2 (margin: CACNB2; RP11-109I13.2) | 5.49 | 6.99e-03 | 3.19e-02 |
|  | 8 | 145654565 | 145654854 | PDFSTATSENSEMBLUCSC | 290 | 5 | 39523 | gene;tss;island | 0.493492 | 0.980491 | VPS28; TONSL (margin: AC084125.4; VPS28; TONSL) | 5.49 | 7.94e-03 | 3.19e-02 |
|  | 7 | 151553471 | 151553868 | PDFSTATSENSEMBLUCSC | 398 | 6 | 37297 | gene | 0.881688 | 0.030597 | PRKAG2 (margin: PRKAG2) | 5.48 | 8.66e-03 | 3.36e-02 |
|  | 1 | 6546574 | 6546839 | PDFSTATSENSEMBLUCSC | 266 | 5 | 176 | gene;tss;island | 0.617785 | 0.037988 | PLEKHG5 (margin: PLEKHG5) | 5.47 | 7.94e-03 | 3.19e-02 |
|  | 1 | 244624521 | 244624883 | PDFSTATSENSEMBLUCSC | 363 | 6 | 4394 | island | 0.418953 | 0.011095 | C1orf101 (margin: C1orf101; ADSS) | 5.47 | 2.16e-03 | 1.45e-02 |
|  | 13 | 113759771 | 113760101 | PDFSTATSENSEMBLUCSC | 331 | 5 | 11547 | tss | 0.539466 | 0.030111 | F7 (margin: MCF2L; F7; AL137002.1) | 5.47 | 7.94e-03 | 3.19e-02 |
|  | 11 | 615945 | 616112 | PDFSTATSENSEMBLUCSC | 168 | 6 | 7020 | tss;island | 0.479860 | 0.011663 | IRF7; CDHR5 (margin: PHRF1; IRF7; CDHR5; SCT) | 5.47 | 4.33e-03 | 2.52e-02 |
|  | 2 | 174219209 | 174219534 | PDFSTATSENSEMBLUCSC | 326 | 5 | 23722 | tss;island | 0.012701 | 0.448256 | CDCA7; AC092573.2 (margin: CDCA7; AC092573.2) | 5.47 | 7.94e-03 | 3.19e-02 |
|  | 6 | 6007259 | 6007769 | PDFSTATSENSEMBLUCSC | 511 | 6 | 35407 | island | 0.401251 | 0.976119 | NRN1 (margin: NRN1) | 5.47 | 2.16e-03 | 1.45e-02 |
|  | 5 | 57878745 | 57879417 | PDFSTATSENSEMBLUCSC | 673 | 12 | 32817 | island | 0.681921 | 0.027506 | RAB3C (margin: RAB3C) | 5.46 | 2.01e-04 | 3.38e-03 |
|  | 8 | 75896645 | 75897310 | PDFSTATSENSEMBLUCSC | 666 | 9 | 40400 | island | 0.418945 | 0.013349 | CRISPLD1 (margin: CRISPLD1) | 5.46 | 4.11e-05 | 1.17e-03 |
|  | 2 | 30454146 | 30454643 | PDFSTATSENSEMBLUCSC | 498 | 8 | 24201 | island | 0.211085 | 0.715719 | LBH (margin: LBH) | 5.46 | 6.22e-04 | 6.71e-03 |
|  | 2 | 30454146 | 30454363 | PDFSTATSENSEMBLUCSC | 218 | 6 | 23285 | tss | 0.213201 | 0.715719 | LBH (margin: LBH) | 5.46 | 4.33e-03 | 2.52e-02 |
|  | 2 | 160761085 | 160761622 | PDFSTATSENSEMBLUCSC | 538 | 8 | 24812 | island | 0.357431 | 0.961751 | LY75; LY75-CD302 (margin: LY75; LY75-CD302) | 5.46 | 1.55e-04 | 2.71e-03 |
|  | 2 | 133428502 | 133429299 | PDFSTATSENSEMBLUCSC | 798 | 9 | 23640 | tss | 0.008623 | 0.132120 | LYPD1; NCKAP5 (margin: AC010974.3; LYPD1; NCKAP5) | 5.46 | 7.77e-03 | 3.19e-02 |
|  | 3 | 9993731 | 9994280 | PDFSTATSENSEMBLUCSC | 550 | 9 | 28868 | island | 0.381345 | 0.009662 | PRRT3-AS1; PRRT3 (margin: CRELD1; PRRT3-AS1; RP11-1020A11.2; PRRT3; EMC3) | 5.45 | 4.11e-05 | 1.17e-03 |
|  | 2 | 135476601 | 135477056 | PDFSTATSENSEMBLUCSC | 456 | 8 | 23642 | tss;island | 0.666534 | 0.053036 | TMEM163 (margin: TMEM163) | 5.45 | 1.04e-02 | 3.96e-02 |
|  | 8 | 75896645 | 75896836 | PDFSTATSENSEMBLUCSC | 192 | 5 | 39775 | tss | 0.380480 | 0.019352 | CRISPLD1 (margin: CRISPLD1) | 5.45 | 7.94e-03 | 3.19e-02 |
|  | 2 | 242805838 | 242806119 | PDFSTATSENSEMBLUCSC | 282 | 5 | 25311 | island | 0.499163 | 0.976368 | (margin: CXXC11; PDCD1) | 5.45 | 7.94e-03 | 3.19e-02 |
|  | 17 | 43922122 | 43922423 | PDFSTATSENSEMBLUCSC | 302 | 6 | 16638 | gene | 0.091335 | 0.828997 | SPPL2C; MAPT-AS1 (margin: CRHR1; SPPL2C; MAPT-AS1) | 5.44 | 2.16e-03 | 1.45e-02 |
|  | 2 | 20866107 | 20866500 | PDFSTATSENSEMBLUCSC | 394 | 9 | 24115 | island | 0.294031 | 0.983572 | GDF7 (margin: GDF7; AC012065.7; RP11-130L8.1) | 5.44 | 4.11e-05 | 1.17e-03 |
|  | 16 | 215410 | 215960 | PDFSTATSENSEMBLUCSC | 551 | 7 | 14962 | tss | 0.544603 | 0.981374 | HBM; HBZP1 (margin: HBZ; HBM; HBZP1; Z84721.4; HBA2; HBA1) | 5.44 | 5.83e-04 | 6.47e-03 |
|  | 6 | 112375186 | 112375333 | PDFSTATSENSEMBLUCSC | 148 | 6 | 35180 | tss | 0.152571 | 0.927626 | WISP3 (margin: WISP3) | 5.43 | 2.16e-03 | 1.45e-02 |
|  | 6 | 31148332 | 31148748 | PDFSTATSENSEMBLUCSC | 417 | 16 | 35754 | island | 0.973725 | 0.506941 | POU5F1 (margin: POU5F1; PSORS1C3) | 5.43 | 3.33e-09 | 1.10e-06 |
|  | 10 | 26504950 | 26505156 | PDFSTATSENSEMBLUCSC | 207 | 5 | 4993 | tss | 0.649864 | 0.033092 | GAD2 (margin: MYO3A; GAD2) | 5.43 | 7.94e-03 | 3.19e-02 |
|  | 22 | 18593294 | 18593441 | PDFSTATSENSEMBLUCSC | 148 | 6 | 26948 | tss | 0.551085 | 0.017932 | PEX26; TUBA8 (margin: PEX26; XXbac-B476C20.10; TUBA8; ARL2BPP10) | 5.42 | 2.16e-03 | 1.45e-02 |
|  | 8 | 60031823 | 60031944 | PDFSTATSENSEMBLUCSC | 122 | 6 | 39722 | tss;island | 0.284781 | 0.925134 | RP11-25K19.1; TOX (margin: RP11-25K19.1; TOX) | 5.42 | 2.16e-03 | 1.45e-02 |
|  | 2 | 176971820 | 176972113 | PDFSTATSENSEMBLUCSC | 294 | 8 | 24896 | island | 0.020965 | 0.473734 | HOXD11; AC009336.1; HOXD10 (margin: HOXD13; HOXD12; HOXD11; AC009336.1; HOXD10) | 5.41 | 1.55e-04 | 2.71e-03 |
|  | 3 | 61547104 | 61547287 | PDFSTATSENSEMBLUCSC | 184 | 7 | 29243 | island | 0.431322 | 0.019209 | PTPRG (margin: PTPRG) | 5.41 | 5.83e-04 | 6.47e-03 |
|  | 11 | 35160330 | 35160892 | PDFSTATSENSEMBLUCSC | 563 | 8 | 8205 | island | 0.008193 | 0.335136 | CD44; RP4-607I7.1 (margin: CD44; AL356215.1; RP4-607I7.1) | 5.41 | 1.09e-03 | 1.10e-02 |
|  | 14 | 55595666 | 55595958 | PDFSTATSENSEMBLUCSC | 293 | 5 | 12864 | island | 0.013638 | 0.428839 | LGALS3 (margin: LGALS3) | 5.41 | 7.94e-03 | 3.19e-02 |
|  | 5 | 172661803 | 172662463 | PDFSTATSENSEMBLUCSC | 661 | 8 | 33407 | island | 0.279337 | 0.934953 | NKX2-5 (margin: RPL7AP33; NKX2-5) | 5.41 | 4.66e-03 | 2.68e-02 |
|  | 17 | 7452289 | 7452644 | PDFSTATSENSEMBLUCSC | 356 | 5 | 17955 | island | 0.219343 | 0.945058 | TNFSF12; TNFSF12-TNFSF13 (margin: TNFSF12; TNFSF12-TNFSF13; TNFSF13; Y\_RNA) | 5.41 | 7.94e-03 | 3.19e-02 |
|  | 17 | 71640253 | 71640369 | PDFSTATSENSEMBLUCSC | 117 | 6 | 17618 | tss | 0.419848 | 0.016548 | RP11-277J6.2; SDK2 (margin: RP11-277J6.2; SDK2) | 5.40 | 4.33e-03 | 2.52e-02 |
|  | 10 | 99531645 | 99531934 | PDFSTATSENSEMBLUCSC | 290 | 8 | 5996 | island | 0.223994 | 0.945905 | SFRP5 (margin: ZFYVE27; SFRP5) | 5.39 | 2.95e-03 | 1.88e-02 |
|  | 5 | 1930801 | 1931781 | PDFSTATSENSEMBLUCSC | 981 | 8 | 32646 | island | 0.428493 | 0.938438 | (margin: RP11-259O2.1) | 5.39 | 1.86e-03 | 1.45e-02 |
|  | 4 | 90757351 | 90757814 | PDFSTATSENSEMBLUCSC | 464 | 7 | 30057 | gene;island | 0.264788 | 0.962328 | RP11-67M1.1; SNCA (margin: RP11-67M1.1; SNCA) | 5.38 | 5.83e-04 | 6.47e-03 |
|  | 6 | 71122912 | 71123041 | PDFSTATSENSEMBLUCSC | 130 | 5 | 35057 | tss | 0.016466 | 0.489987 | FAM135A; RP11-462G2.2 (margin: FAM135A; RP11-462G2.2) | 5.38 | 7.94e-03 | 3.19e-02 |
|  | 21 | 22369802 | 22370444 | PDFSTATSENSEMBLUCSC | 643 | 6 | 26495 | tss;island | 0.817345 | 0.279340 | NCAM2 (margin: NCAM2) | 5.38 | 2.16e-03 | 1.45e-02 |
|  | 16 | 67312928 | 67313043 | PDFSTATSENSEMBLUCSC | 116 | 7 | 14797 | gene;tss;island | 0.372309 | 0.034200 | PLEKHG4 (margin: SLC9A5; PLEKHG4; KCTD19) | 5.38 | 4.08e-03 | 2.47e-02 |
|  | 3 | 192958383 | 192958968 | PDFSTATSENSEMBLUCSC | 586 | 7 | 28794 | tss;island | 0.413652 | 0.972772 | HRASLS (margin: HRASLS) | 5.38 | 5.83e-04 | 6.47e-03 |
|  | 11 | 119455041 | 119455905 | PDFSTATSENSEMBLUCSC | 865 | 8 | 8885 | island | 0.012682 | 0.381139 | (margin: ) | 5.38 | 1.55e-04 | 2.71e-03 |
|  | 17 | 45772333 | 45772800 | PDFSTATSENSEMBLUCSC | 468 | 7 | 18457 | island | 0.345098 | 0.972495 | TBKBP1 (margin: KPNB1; TBKBP1) | 5.38 | 1.17e-03 | 1.13e-02 |
|  | 6 | 29601398 | 29601705 | PDFSTATSENSEMBLUCSC | 308 | 6 | 34750 | tss;island | 0.940755 | 0.227902 | GABBR1 (margin: GABBR1; SUMO2P1) | 5.38 | 2.16e-03 | 1.45e-02 |
|  | 20 | 47896769 | 47897451 | PDFSTATSENSEMBLUCSC | 683 | 7 | 25447 | gene;island | 0.009393 | 0.381828 | ZFAS1; SNORD12C; SNORD12B; SNORD12; ZNFX1 (margin: ZFAS1; SNORD12C; SNORD12B; SNORD12; ZNFX1) | 5.37 | 5.83e-04 | 6.47e-03 |
|  | 20 | 47896769 | 47897207 | PDFSTATSENSEMBLUCSC | 439 | 5 | 25807 | tss | 0.011800 | 0.381828 | ZFAS1; SNORD12C; SNORD12B; SNORD12; ZNFX1 (margin: ZFAS1; SNORD12C; SNORD12B; SNORD12; ZNFX1) | 5.37 | 7.94e-03 | 3.19e-02 |
|  | 17 | 54670875 | 54671575 | PDFSTATSENSEMBLUCSC | 701 | 6 | 18577 | island | 0.485699 | 0.024918 | NOG (margin: NOG) | 5.37 | 2.16e-03 | 1.45e-02 |
|  | 19 | 40724308 | 40724388 | PDFSTATSENSEMBLUCSC | 81 | 5 | 20783 | tss;island | 0.308146 | 0.955682 | TTC9B (margin: MAP3K10; TTC9B; CNTD2; AKT2) | 5.37 | 7.94e-03 | 3.19e-02 |
|  | 6 | 32525813 | 32526027 | PDFSTATSENSEMBLUCSC | 215 | 5 | 34117 | gene | 0.136934 | 0.760662 | HLA-DRB6 (margin: RNU1-61P; HLA-DRB6) | 5.37 | 7.94e-03 | 3.19e-02 |
|  | 11 | 133827056 | 133827256 | PDFSTATSENSEMBLUCSC | 201 | 5 | 7826 | tss;island | 0.346179 | 0.010533 | IGSF9B; AP000911.1 (margin: IGSF9B; AP000911.1) | 5.37 | 7.94e-03 | 3.19e-02 |
|  | 11 | 46366643 | 46366884 | PDFSTATSENSEMBLUCSC | 242 | 6 | 7276 | tss | 0.169284 | 0.901455 | DGKZ (margin: DGKZ) | 5.37 | 2.16e-03 | 1.45e-02 |
|  | 6 | 71666682 | 71666858 | PDFSTATSENSEMBLUCSC | 177 | 5 | 36242 | island | 0.446170 | 0.969011 | B3GAT2 (margin: B3GAT2) | 5.35 | 7.94e-03 | 3.19e-02 |
|  | 7 | 87104724 | 87105416 | PDFSTATSENSEMBLUCSC | 693 | 8 | 38635 | island | 0.471797 | 0.976440 | ABCB4 (margin: ABCB4) | 5.35 | 1.55e-04 | 2.71e-03 |
|  | 12 | 132293329 | 132293702 | PDFSTATSENSEMBLUCSC | 374 | 5 | 11015 | island | 0.524169 | 0.984082 | (margin: SFSWAP; RNA5SP378; RNU6-1017P) | 5.35 | 7.94e-03 | 3.19e-02 |
|  | 5 | 63461216 | 63461930 | PDFSTATSENSEMBLUCSC | 715 | 10 | 32828 | island | 0.665439 | 0.039022 | RNF180 (margin: RNF180) | 5.34 | 2.06e-04 | 3.42e-03 |
|  | 3 | 187870621 | 187871034 | PDFSTATSENSEMBLUCSC | 414 | 7 | 28780 | tss;island | 0.195855 | 0.002931 | LPP; LPP-AS2 (margin: RP11-430L16.1; LPP; LPP-AS2) | 5.34 | 5.83e-04 | 6.47e-03 |
|  | 17 | 27045043 | 27045302 | PDFSTATSENSEMBLUCSC | 260 | 6 | 17218 | tss;island | 0.008018 | 0.338559 | RPL23A; RAB34 (margin: RPL23A; SNORD42B; SNORD4A; SNORD42A; SNORD4B; NEK8; AC010761.14; PROCA1; RAB34; AC010761.8; TLCD1) | 5.34 | 2.16e-03 | 1.45e-02 |
|  | 2 | 160761262 | 160761622 | PDFSTATSENSEMBLUCSC | 361 | 6 | 23683 | tss | 0.357431 | 0.960949 | LY75; LY75-CD302 (margin: LY75; LY75-CD302) | 5.33 | 2.16e-03 | 1.45e-02 |
|  | 2 | 131513608 | 131513735 | PDFSTATSENSEMBLUCSC | 128 | 5 | 23630 | tss | 0.424751 | 0.025977 | AMER3; AC140481.8 (margin: AMER3; AC140481.8) | 5.33 | 7.94e-03 | 3.19e-02 |
|  | 19 | 58446745 | 58446988 | PDFSTATSENSEMBLUCSC | 244 | 8 | 21129 | tss | 0.952230 | 0.361115 | ZNF418 (margin: ZNF418; ZNF256) | 5.33 | 1.55e-04 | 2.71e-03 |
|  | 1 | 115631930 | 115632727 | PDFSTATSENSEMBLUCSC | 798 | 10 | 3628 | island | 0.015525 | 0.513893 | TSPAN2 (margin: RP4-666F24.3; TSPAN2) | 5.32 | 1.08e-05 | 4.82e-04 |
|  | 10 | 18629497 | 18629677 | PDFSTATSENSEMBLUCSC | 181 | 6 | 4522 | gene | 0.440669 | 0.959524 | CACNB2 (margin: CACNB2) | 5.32 | 2.16e-03 | 1.45e-02 |
|  | 19 | 37825307 | 37825446 | PDFSTATSENSEMBLUCSC | 140 | 6 | 20736 | tss;island | 0.883959 | 0.465372 | HKR1 (margin: HKR1; CTD-3220F14.2) | 5.32 | 2.16e-03 | 1.45e-02 |
|  | 13 | 20767498 | 20768560 | PDFSTATSENSEMBLUCSC | 1063 | 13 | 11329 | tss;island | 0.238638 | 0.959495 | GJB2 (margin: LINC00556; GJB2) | 5.32 | 5.77e-06 | 3.56e-04 |
|  | 5 | 59189649 | 59189934 | PDFSTATSENSEMBLUCSC | 286 | 8 | 32079 | tss | 0.726217 | 0.054985 | PDE4D (margin: PDE4D) | 5.31 | 1.55e-04 | 2.71e-03 |
|  | 10 | 7409524 | 7409783 | PDFSTATSENSEMBLUCSC | 260 | 5 | 4481 | gene;island | 0.662286 | 0.983266 | SFMBT2 (margin: SFMBT2) | 5.31 | 7.94e-03 | 3.19e-02 |
|  | 11 | 64937570 | 64937697 | PDFSTATSENSEMBLUCSC | 128 | 6 | 7419 | tss | 0.935295 | 0.243261 | SPDYC (margin: SPDYC; CAPN1; AP003068.17; AP003068.18; PGAM1P8; AP003068.23) | 5.31 | 2.16e-03 | 1.45e-02 |
|  | 11 | 65405268 | 65405903 | PDFSTATSENSEMBLUCSC | 636 | 12 | 8472 | island | 0.029097 | 0.538585 | PCNXL3; MIR4690; SIPA1 (margin: PCNXL3; MIR4690; SIPA1; MIR4489) | 5.31 | 4.96e-04 | 6.47e-03 |
|  | 2 | 109745210 | 109745841 | PDFSTATSENSEMBLUCSC | 632 | 8 | 22822 | gene;tss;island | 0.268846 | 0.921915 | SH3RF3; SH3RF3-AS1 (margin: SH3RF3; SH3RF3-AS1) | 5.30 | 1.09e-03 | 1.10e-02 |
|  | 12 | 45269318 | 45269790 | PDFSTATSENSEMBLUCSC | 473 | 7 | 9100 | gene;island | 0.289309 | 0.020361 | NELL2 (margin: NELL2; Y\_RNA) | 5.30 | 1.11e-02 | 4.14e-02 |
|  | 4 | 8271196 | 8271507 | PDFSTATSENSEMBLUCSC | 312 | 7 | 30917 | island | 0.451292 | 0.964515 | HTRA3 (margin: HTRA3) | 5.30 | 5.83e-04 | 6.47e-03 |
|  | 11 | 128775513 | 128775750 | PDFSTATSENSEMBLUCSC | 238 | 7 | 6969 | gene | 0.007703 | 0.158108 | KCNJ5; C11orf45 (margin: KCNJ5; C11orf45) | 5.30 | 5.83e-04 | 6.47e-03 |
|  | 20 | 30639705 | 30640041 | PDFSTATSENSEMBLUCSC | 337 | 8 | 25671 | tss | 0.355977 | 0.947828 | RNA5SP482; HCK (margin: RNA5SP482; HCK) | 5.30 | 1.09e-03 | 1.10e-02 |
|  | 15 | 65341703 | 65341995 | PDFSTATSENSEMBLUCSC | 293 | 5 | 13378 | gene | 0.240295 | 0.005140 | SLC51B (margin: SLC51B; RASL12) | 5.29 | 7.94e-03 | 3.19e-02 |
|  | 6 | 166401536 | 166401700 | PDFSTATSENSEMBLUCSC | 165 | 5 | 35333 | tss | 0.159742 | 0.003473 | PDE10A; LINC00473 (margin: PDE10A; LINC00473) | 5.29 | 7.94e-03 | 3.19e-02 |
|  | 7 | 27199726 | 27199956 | PDFSTATSENSEMBLUCSC | 231 | 5 | 38355 | island | 0.698292 | 0.989795 | RP1-170O19.21 (margin: HOXA-AS3; RP1-170O19.21; HOXA-AS4; HOXA3; HOXA6; HOXA7; HOXA9; RP1-170O19.20; MIR196B; HOXA10) | 5.29 | 7.94e-03 | 3.19e-02 |
|  | 6 | 127664674 | 127664950 | PDFSTATSENSEMBLUCSC | 277 | 6 | 35216 | tss;island | 0.027107 | 0.464196 | ECHDC1 (margin: YWHAZP4; ECHDC1) | 5.29 | 2.16e-03 | 1.45e-02 |
|  | 7 | 130125200 | 130126637 | PDFSTATSENSEMBLUCSC | 1438 | 18 | 38921 | island | 0.512617 | 0.020161 | MEST; RP11-2E11.5 (margin: MEST; MIR335; RP11-2E11.5; hsa-mir-335) | 5.29 | 2.11e-04 | 3.49e-03 |
|  | 11 | 3239953 | 3240399 | PDFSTATSENSEMBLUCSC | 447 | 6 | 6478 | gene;island | 0.046947 | 0.794759 | MRGPRG-AS1; MRGPRG (margin: MRGPRG-AS1; AC109309.4; MRGPRG; MRGPRE) | 5.28 | 2.16e-03 | 1.45e-02 |
|  | 19 | 418899 | 419190 | PDFSTATSENSEMBLUCSC | 292 | 5 | 19626 | gene;island | 0.440726 | 0.971018 | SHC2 (margin: C2CD4C; SHC2) | 5.28 | 7.94e-03 | 3.19e-02 |
|  | 17 | 33775295 | 33775779 | PDFSTATSENSEMBLUCSC | 485 | 5 | 16520 | gene | 0.314494 | 0.966579 | SLFN13 (margin: SLFN13; RP11-1094M14.7) | 5.28 | 7.94e-03 | 3.19e-02 |
|  | 4 | 77355817 | 77356250 | PDFSTATSENSEMBLUCSC | 434 | 5 | 30449 | tss | 0.281378 | 0.012750 | SHROOM3 (margin: SHROOM3) | 5.27 | 7.94e-03 | 3.19e-02 |
|  | 4 | 185940923 | 185941304 | PDFSTATSENSEMBLUCSC | 382 | 5 | 30207 | gene;island | 0.397556 | 0.951253 | HELT (margin: HELT) | 5.27 | 7.94e-03 | 3.19e-02 |
|  | 1 | 17446027 | 17446162 | PDFSTATSENSEMBLUCSC | 136 | 5 | 1291 | tss | 0.434895 | 0.020577 | PADI2 (margin: PADI2) | 5.27 | 7.94e-03 | 3.19e-02 |
|  | 15 | 64443650 | 64444055 | PDFSTATSENSEMBLUCSC | 406 | 10 | 14212 | island | 0.506168 | 0.019056 | SNX22 (margin: SNX1; SNX22; PPIB) | 5.26 | 8.93e-03 | 3.45e-02 |
|  | 2 | 154333481 | 154334077 | PDFSTATSENSEMBLUCSC | 597 | 9 | 24791 | island | 0.245991 | 0.005185 | AC012501.2; RPRM (margin: AC012501.2; RPRM) | 5.26 | 8.23e-05 | 2.06e-03 |
|  | 11 | 826837 | 827613 | PDFSTATSENSEMBLUCSC | 777 | 7 | 7885 | island | 0.434926 | 0.019704 | PNPLA2; EFCAB4A; AP006621.8 (margin: PNPLA2; EFCAB4A; CD151; AP006621.8; POLR2L) | 5.26 | 5.83e-04 | 6.47e-03 |
|  | 20 | 57462798 | 57464129 | PDFSTATSENSEMBLUCSC | 1332 | 37 | 25473 | gene;tss;island | 0.577175 | 0.978901 | GNAS; RP1-309F20.3 (margin: GNAS; RP1-309F20.3; RP1-309F20.4) | 5.26 | 1.34e-17 | 6.55e-14 |
|  | 4 | 5021084 | 5021328 | PDFSTATSENSEMBLUCSC | 245 | 8 | 30875 | island | 0.385327 | 0.974312 | CYTL1 (margin: CYTL1) | 5.25 | 3.11e-04 | 4.77e-03 |
|  | 12 | 114838567 | 114839007 | PDFSTATSENSEMBLUCSC | 441 | 5 | 9324 | gene;island | 0.313676 | 0.968563 | TBX5 (margin: TBX5-AS1; TBX5) | 5.24 | 7.94e-03 | 3.19e-02 |
|  | 8 | 81397601 | 81397876 | PDFSTATSENSEMBLUCSC | 276 | 5 | 39782 | tss;island | 0.048556 | 0.003771 | ZBTB10; Y\_RNA (margin: ZBTB10; Y\_RNA) | 5.24 | 7.94e-03 | 3.19e-02 |
|  | 6 | 150311305 | 150312368 | PDFSTATSENSEMBLUCSC | 1064 | 9 | 36564 | island | 0.184427 | 0.962426 | (margin: RP11-472G23.3; BTF3P10; RAET1K) | 5.24 | 4.11e-05 | 1.17e-03 |
|  | 8 | 24772137 | 24772435 | PDFSTATSENSEMBLUCSC | 299 | 7 | 39605 | tss | 0.667441 | 0.981603 | NEFM; GS1-72M22.1 (margin: NEFM; RP11-624C23.1; GS1-72M22.1) | 5.23 | 5.83e-04 | 6.47e-03 |
|  | 11 | 3240068 | 3240399 | PDFSTATSENSEMBLUCSC | 332 | 5 | 7093 | tss | 0.053077 | 0.678098 | MRGPRG-AS1; MRGPRG (margin: MRGPRG-AS1; AC109309.4; MRGPRG; MRGPRE) | 5.23 | 7.94e-03 | 3.19e-02 |
|  | 4 | 165304259 | 165304540 | PDFSTATSENSEMBLUCSC | 282 | 8 | 31404 | island | 0.287069 | 0.952757 | MARCH1 (margin: MARCH1) | 5.23 | 3.11e-04 | 4.77e-03 |
|  | 4 | 165304443 | 165304540 | PDFSTATSENSEMBLUCSC | 98 | 6 | 30663 | tss | 0.318970 | 0.952757 | MARCH1 (margin: MARCH1) | 5.23 | 2.16e-03 | 1.45e-02 |
|  | 3 | 55521351 | 55521529 | PDFSTATSENSEMBLUCSC | 179 | 5 | 28402 | tss;island | 0.234347 | 0.008240 | WNT5A-AS1; WNT5A (margin: WNT5A-AS1; WNT5A; RP11-875H7.5) | 5.23 | 7.94e-03 | 3.19e-02 |
|  | 5 | 59189375 | 59189934 | PDFSTATSENSEMBLUCSC | 560 | 10 | 31625 | gene;island | 0.726217 | 0.069314 | PDE4D (margin: PDE4D) | 5.23 | 3.25e-04 | 4.91e-03 |
|  | 17 | 80797948 | 80798078 | PDFSTATSENSEMBLUCSC | 131 | 7 | 17781 | tss | 0.336913 | 0.015865 | TBCD; ZNF750 (margin: TBCD; ZNF750) | 5.23 | 5.83e-04 | 6.47e-03 |
|  | 11 | 46366643 | 46367100 | PDFSTATSENSEMBLUCSC | 458 | 8 | 6598 | gene;island | 0.185126 | 0.901455 | DGKZ (margin: DGKZ) | 5.22 | 1.55e-04 | 2.71e-03 |
|  | 7 | 94953653 | 94954202 | PDFSTATSENSEMBLUCSC | 550 | 9 | 38677 | island | 0.491455 | 0.977226 | PON1 (margin: PON1) | 5.22 | 4.11e-05 | 1.17e-03 |
|  | 3 | 145968620 | 145968726 | PDFSTATSENSEMBLUCSC | 107 | 6 | 27983 | gene;island | 0.171166 | 0.007655 | PLSCR4 (margin: PLSCR4) | 5.22 | 8.66e-03 | 3.36e-02 |
|  | 7 | 98972104 | 98972197 | PDFSTATSENSEMBLUCSC | 94 | 6 | 37752 | tss;island | 0.442184 | 0.030038 | ARPC1A; ARPC1B (margin: ARPC1A; ARPC1B) | 5.22 | 2.16e-03 | 1.45e-02 |
|  | 12 | 9227173 | 9227320 | PDFSTATSENSEMBLUCSC | 148 | 5 | 9051 | gene | 0.044872 | 0.490089 | A2M (margin: A2M-AS1; LINC00612; A2M) | 5.22 | 7.94e-03 | 3.19e-02 |
|  | 19 | 39989234 | 39989592 | PDFSTATSENSEMBLUCSC | 359 | 8 | 21999 | island | 0.070824 | 0.610580 | DLL3 (margin: TIMM50; DLL3) | 5.21 | 6.22e-04 | 6.71e-03 |
|  | 22 | 45809596 | 45810043 | PDFSTATSENSEMBLUCSC | 448 | 8 | 26904 | gene | 0.017408 | 0.288738 | RIBC2; SMC1B (margin: RIBC2; SMC1B) | 5.21 | 3.11e-04 | 4.77e-03 |
|  | 15 | 65203834 | 65204146 | PDFSTATSENSEMBLUCSC | 313 | 6 | 14222 | island | 0.409145 | 0.019701 | AC069368.3; ANKDD1A (margin: AC069368.3; ANKDD1A; RN7SL348P) | 5.21 | 2.16e-03 | 1.45e-02 |
|  | 10 | 5566543 | 5566908 | PDFSTATSENSEMBLUCSC | 366 | 6 | 4926 | tss;island | 0.197042 | 0.886065 | CALML3; CALML3-AS1; RP11-116G8.5 (margin: CALML3; CALML3-AS1; RP11-116G8.5) | 5.21 | 2.16e-03 | 1.45e-02 |
|  | 4 | 154125208 | 154125720 | PDFSTATSENSEMBLUCSC | 513 | 9 | 30152 | gene | 0.970704 | 0.366064 | TRIM2 (margin: TRIM2) | 5.20 | 7.82e-04 | 8.17e-03 |
|  | 19 | 53662261 | 53662723 | PDFSTATSENSEMBLUCSC | 463 | 9 | 22369 | island | 0.892812 | 0.281867 | ZNF347; ZNF665 (margin: CTD-2245F17.2; ZNF347; ZNF665) | 5.20 | 8.23e-05 | 2.06e-03 |
|  | 1 | 201915757 | 201915999 | PDFSTATSENSEMBLUCSC | 243 | 7 | 2170 | tss | 0.453973 | 0.021186 | LMOD1 (margin: TIMM17A; LMOD1) | 5.19 | 1.17e-03 | 1.13e-02 |
|  | 11 | 843897 | 844400 | PDFSTATSENSEMBLUCSC | 504 | 8 | 7033 | tss | 0.676259 | 0.056119 | TSPAN4; POLR2L (margin: EFCAB4A; CD151; TSPAN4; AP006621.8; POLR2L) | 5.19 | 1.55e-04 | 2.71e-03 |
|  | 16 | 68481813 | 68482277 | PDFSTATSENSEMBLUCSC | 465 | 5 | 14816 | gene;island | 0.647321 | 0.048241 | SMPD3 (margin: SMPD3) | 5.19 | 7.94e-03 | 3.19e-02 |
|  | 6 | 125684510 | 125684960 | PDFSTATSENSEMBLUCSC | 451 | 8 | 36460 | island | 0.427742 | 0.023079 | (margin: RP11-735G4.1) | 5.19 | 1.55e-04 | 2.71e-03 |
|  | 14 | 69951211 | 69951422 | PDFSTATSENSEMBLUCSC | 212 | 6 | 12481 | tss | 0.480368 | 0.024134 | PLEKHD1 (margin: PLEKHD1) | 5.19 | 2.16e-03 | 1.45e-02 |
|  | 11 | 26353484 | 26353630 | PDFSTATSENSEMBLUCSC | 147 | 5 | 7206 | tss | 0.594606 | 0.025228 | ANO3 (margin: ANO3) | 5.19 | 7.94e-03 | 3.19e-02 |
|  | 1 | 111746873 | 111747324 | PDFSTATSENSEMBLUCSC | 452 | 8 | 3589 | island | 0.440277 | 0.025789 | CHI3L2; DENND2D (margin: CHI3L2; DENND2D) | 5.18 | 1.55e-04 | 2.71e-03 |
|  | 10 | 131354782 | 131355302 | PDFSTATSENSEMBLUCSC | 521 | 9 | 4826 | gene | 0.712452 | 0.985817 | MGMT (margin: MGMT) | 5.18 | 4.11e-05 | 1.17e-03 |
|  | 15 | 73661642 | 73662002 | PDFSTATSENSEMBLUCSC | 361 | 6 | 13781 | tss | 0.413589 | 0.960301 | HCN4 (margin: HCN4) | 5.18 | 4.33e-03 | 2.52e-02 |
|  | 20 | 50419082 | 50419348 | PDFSTATSENSEMBLUCSC | 267 | 7 | 25826 | tss | 0.972177 | 0.523511 | SALL4 (margin: SALL4) | 5.17 | 5.83e-04 | 6.47e-03 |
|  | 1 | 19615303 | 19615702 | PDFSTATSENSEMBLUCSC | 400 | 11 | 1302 | tss;island | 0.609638 | 0.977349 | AKR7A3 (margin: RP4-657E11.10; AKR7A3) | 5.17 | 8.51e-05 | 2.07e-03 |
|  | 12 | 108523395 | 108523597 | PDFSTATSENSEMBLUCSC | 203 | 6 | 10767 | island | 0.795360 | 0.034562 | WSCD2 (margin: WSCD2) | 5.17 | 8.66e-03 | 3.36e-02 |
|  | 18 | 43913565 | 43913755 | PDFSTATSENSEMBLUCSC | 191 | 5 | 19256 | tss;island | 0.337040 | 0.011847 | RNF165 (margin: RNF165) | 5.17 | 7.94e-03 | 3.19e-02 |
|  | 19 | 2702863 | 2703086 | PDFSTATSENSEMBLUCSC | 224 | 10 | 20329 | tss;island | 0.742810 | 0.008144 | GNG7 (margin: GNG7; DIRAS1) | 5.16 | 8.93e-03 | 3.45e-02 |
|  | 13 | 27335082 | 27335542 | PDFSTATSENSEMBLUCSC | 461 | 9 | 11356 | tss;island | 0.351129 | 0.955254 | GPR12 (margin: GPR12) | 5.16 | 4.11e-05 | 1.17e-03 |
|  | 8 | 30890108 | 30890632 | PDFSTATSENSEMBLUCSC | 525 | 11 | 39271 | gene;tss | 0.553844 | 0.028674 | WRN; PURG (margin: WRN; PURG) | 5.16 | 1.28e-02 | 4.76e-02 |
|  | 5 | 65891878 | 65892109 | PDFSTATSENSEMBLUCSC | 232 | 7 | 32101 | tss | 0.316111 | 0.009449 | MAST4 (margin: MAST4) | 5.16 | 1.17e-03 | 1.13e-02 |
|  | 19 | 7852051 | 7852238 | PDFSTATSENSEMBLUCSC | 188 | 6 | 20407 | tss;island | 0.169891 | 0.931435 | CLEC4GP1 (margin: CLEC4GP1) | 5.16 | 2.16e-03 | 1.45e-02 |
|  | 16 | 89044523 | 89044882 | PDFSTATSENSEMBLUCSC | 360 | 5 | 15418 | tss | 0.052894 | 0.710303 | CBFA2T3 (margin: CTD-2555A7.1; CBFA2T3) | 5.16 | 7.94e-03 | 3.19e-02 |
|  | 6 | 124124875 | 124125371 | PDFSTATSENSEMBLUCSC | 497 | 6 | 36455 | island | 0.577329 | 0.046300 | NKAIN2 (margin: NKAIN2) | 5.15 | 8.66e-03 | 3.36e-02 |
|  | 13 | 44361019 | 44361220 | PDFSTATSENSEMBLUCSC | 202 | 5 | 11699 | island | 0.113119 | 0.006501 | RP11-168P13.1; ENOX1 (margin: RP11-168P13.1; ENOX1) | 5.15 | 7.94e-03 | 3.19e-02 |
|  | 3 | 168864101 | 168864313 | PDFSTATSENSEMBLUCSC | 213 | 5 | 28706 | tss | 0.009253 | 0.248934 | MECOM (margin: MECOM) | 5.15 | 7.94e-03 | 3.19e-02 |
|  | 2 | 33359198 | 33359550 | PDFSTATSENSEMBLUCSC | 353 | 5 | 23298 | tss | 0.127685 | 0.916879 | LTBP1 (margin: LTBP1) | 5.15 | 7.94e-03 | 3.19e-02 |
|  | 1 | 77747875 | 77748221 | PDFSTATSENSEMBLUCSC | 347 | 6 | 1705 | tss | 0.459902 | 0.020755 | AK5 (margin: AK5) | 5.15 | 4.33e-03 | 2.52e-02 |
|  | 20 | 1246703 | 1247058 | PDFSTATSENSEMBLUCSC | 356 | 8 | 25935 | island | 0.317867 | 0.929687 | RAD21L1; SNPH (margin: RAD21L1; SNPH) | 5.15 | 1.55e-04 | 2.71e-03 |
|  | 20 | 1246703 | 1246934 | PDFSTATSENSEMBLUCSC | 232 | 6 | 25536 | tss | 0.429381 | 0.935216 | RAD21L1; SNPH (margin: RAD21L1; SNPH) | 5.15 | 2.16e-03 | 1.45e-02 |
|  | 2 | 20866107 | 20866414 | PDFSTATSENSEMBLUCSC | 308 | 8 | 23215 | tss | 0.419811 | 0.983403 | GDF7 (margin: GDF7; AC012065.7; RP11-130L8.1) | 5.14 | 1.55e-04 | 2.71e-03 |
|  | 1 | 1238436 | 1238797 | PDFSTATSENSEMBLUCSC | 362 | 5 | 27 | gene;island | 0.311613 | 0.960958 | ACAP3 (margin: SCNN1D; PUSL1; ACAP3; CPSF3L) | 5.14 | 7.94e-03 | 3.19e-02 |
|  | 1 | 236559257 | 236559675 | PDFSTATSENSEMBLUCSC | 419 | 7 | 1097 | gene;island | 0.027803 | 0.597609 | EDARADD (margin: EDARADD) | 5.14 | 6.99e-03 | 3.19e-02 |
|  | 10 | 128077307 | 128077376 | PDFSTATSENSEMBLUCSC | 70 | 6 | 5433 | tss;island | 0.587569 | 0.979567 | ADAM12 (margin: ADAM12) | 5.14 | 2.16e-03 | 1.45e-02 |
|  | 21 | 46493156 | 46493436 | PDFSTATSENSEMBLUCSC | 281 | 5 | 26592 | tss;island | 0.856554 | 0.151464 | ADARB1; SSR4P1; AP001579.1 (margin: ADARB1; SSR4P1; AP001579.1) | 5.14 | 7.94e-03 | 3.19e-02 |
|  | 2 | 25391505 | 25391911 | PDFSTATSENSEMBLUCSC | 407 | 11 | 24139 | island | 0.503666 | 0.967084 | POMC (margin: RP11-509E16.1; POMC) | 5.14 | 2.84e-06 | 1.96e-04 |
|  | 4 | 108852496 | 108852651 | PDFSTATSENSEMBLUCSC | 156 | 7 | 30535 | tss;island | 0.227892 | 0.011491 | CYP2U1; RP11-286E11.1 (margin: CYP2U1; RP11-286E11.1; RP11-286E11.2) | 5.14 | 5.83e-04 | 6.47e-03 |
|  | 6 | 74161911 | 74162142 | PDFSTATSENSEMBLUCSC | 232 | 7 | 36256 | island | 0.023750 | 0.412442 | MB21D1 (margin: MTO1; RNU6-975P; MB21D1) | 5.13 | 5.83e-04 | 6.47e-03 |
|  | 3 | 44903463 | 44903730 | PDFSTATSENSEMBLUCSC | 268 | 5 | 27745 | gene | 0.021293 | 0.428484 | KIF15; TMEM42; MIR564 (margin: KIF15; TMEM42; MIR564) | 5.13 | 7.94e-03 | 3.19e-02 |
|  | 8 | 33457483 | 33457822 | PDFSTATSENSEMBLUCSC | 340 | 6 | 39642 | tss | 0.394121 | 0.965104 | DUSP26 (margin: RP11-317N12.1; DUSP26) | 5.13 | 2.16e-03 | 1.45e-02 |
|  | 17 | 80185943 | 80186336 | PDFSTATSENSEMBLUCSC | 394 | 6 | 17769 | tss;island | 0.471860 | 0.974856 | SLC16A3 (margin: RP13-516M14.2; SLC16A3; CSNK1D) | 5.12 | 8.66e-03 | 3.36e-02 |
|  | 12 | 12502846 | 12503544 | PDFSTATSENSEMBLUCSC | 699 | 10 | 10246 | island | 0.387336 | 0.019286 | MANSC1 (margin: RP11-757G14.1; LOH12CR1; MANSC1; LOH12CR2) | 5.12 | 2.17e-05 | 8.94e-04 |
|  | 12 | 12503186 | 12503544 | PDFSTATSENSEMBLUCSC | 359 | 8 | 9569 | tss | 0.365928 | 0.019286 | MANSC1 (margin: RP11-757G14.1; LOH12CR1; MANSC1; LOH12CR2) | 5.12 | 1.55e-04 | 2.71e-03 |
|  | 4 | 176986621 | 176986950 | PDFSTATSENSEMBLUCSC | 330 | 7 | 30687 | tss | 0.493372 | 0.010285 | WDR17 (margin: WDR17) | 5.12 | 2.33e-03 | 1.52e-02 |
|  | 8 | 70378380 | 70378760 | PDFSTATSENSEMBLUCSC | 381 | 6 | 39752 | tss | 0.039381 | 0.567749 | SULF1 (margin: SULF1) | 5.12 | 8.66e-03 | 3.36e-02 |
|  | 4 | 980604 | 980676 | PDFSTATSENSEMBLUCSC | 73 | 5 | 29852 | gene;tss;island | 0.246837 | 0.011616 | IDUA; DGKQ; SLC26A1 (margin: IDUA; DGKQ; SLC26A1) | 5.12 | 7.94e-03 | 3.19e-02 |
|  | 20 | 30639705 | 30640256 | PDFSTATSENSEMBLUCSC | 552 | 11 | 26089 | island | 0.374674 | 0.945993 | RNA5SP482; HCK (margin: RNA5SP482; HCK) | 5.12 | 5.39e-05 | 1.47e-03 |
|  | 19 | 52490101 | 52490339 | PDFSTATSENSEMBLUCSC | 239 | 9 | 21002 | tss | 0.518917 | 0.014081 | ZNF350 (margin: HCCAT3; ZNF350; ZNF615) | 5.11 | 4.11e-05 | 1.17e-03 |
|  | 19 | 12624679 | 12624832 | PDFSTATSENSEMBLUCSC | 154 | 6 | 20498 | tss | 0.579203 | 0.982915 | ZNF709 (margin: CTD-3105H18.8; CTD-3105H18.10; CTD-3105H18.9; CTD-3105H18.11; PPIAP20; ZNF709; ZNF564; CTD-2192J16.20) | 5.11 | 2.16e-03 | 1.45e-02 |
|  | 17 | 32907636 | 32907997 | PDFSTATSENSEMBLUCSC | 362 | 7 | 18202 | island | 0.573578 | 0.185917 | TMEM132E; C17orf102 (margin: TMEM132E; C17orf102) | 5.10 | 1.11e-02 | 4.14e-02 |
|  | 5 | 65891878 | 65892187 | PDFSTATSENSEMBLUCSC | 310 | 8 | 32843 | island | 0.285027 | 0.015871 | MAST4 (margin: MAST4) | 5.10 | 6.22e-04 | 6.71e-03 |
|  | 7 | 30810852 | 30810882 | PDFSTATSENSEMBLUCSC | 31 | 5 | 37521 | tss;island | 0.601242 | 0.044448 | INMT-FAM188B; FAM188B (margin: INMT-FAM188B; FAM188B) | 5.10 | 7.94e-03 | 3.19e-02 |
|  | 15 | 75470777 | 75471218 | PDFSTATSENSEMBLUCSC | 442 | 6 | 14325 | island | 0.967945 | 0.643665 | (margin: RPL36AP45) | 5.10 | 2.16e-03 | 1.45e-02 |
|  | 1 | 247275326 | 247276096 | PDFSTATSENSEMBLUCSC | 771 | 10 | 4414 | island | 0.694229 | 0.049019 | C1orf229 (margin: FGFR3P6; ZNF669; C1orf229; ZNF124) | 5.08 | 5.20e-03 | 2.96e-02 |
|  | 7 | 51384371 | 51384676 | PDFSTATSENSEMBLUCSC | 306 | 7 | 38495 | island | 0.374087 | 0.017654 | COBL (margin: COBL) | 5.08 | 5.83e-04 | 6.47e-03 |
|  | 3 | 10857688 | 10857800 | PDFSTATSENSEMBLUCSC | 113 | 8 | 28153 | tss;island | 0.410025 | 0.960746 | SLC6A11 (margin: SLC6A11) | 5.08 | 1.55e-04 | 2.71e-03 |
|  | 14 | 102026230 | 102026555 | PDFSTATSENSEMBLUCSC | 326 | 10 | 12635 | tss | 0.517699 | 0.974135 | DIO3; DIO3OS (margin: DIO3; DIO3OS) | 5.08 | 1.08e-05 | 4.82e-04 |
|  | 4 | 93224895 | 93225466 | PDFSTATSENSEMBLUCSC | 572 | 7 | 30499 | tss | 0.525546 | 0.025623 | GRID2; RP11-9B6.1 (margin: GRID2; RP11-9B6.1) | 5.08 | 2.33e-03 | 1.52e-02 |
|  | 6 | 166139905 | 166140706 | PDFSTATSENSEMBLUCSC | 802 | 8 | 36621 | island | 0.263519 | 0.948228 | PDE10A (margin: PDE10A; RNU6-730P) | 5.08 | 1.55e-04 | 2.71e-03 |
|  | 6 | 49681299 | 49681391 | PDFSTATSENSEMBLUCSC | 93 | 5 | 35027 | tss | 0.585130 | 0.982850 | CRISP2 (margin: CRISP2) | 5.07 | 7.94e-03 | 3.19e-02 |
|  | 2 | 154335014 | 154335386 | PDFSTATSENSEMBLUCSC | 373 | 9 | 24792 | island | 0.115355 | 0.011100 | AC012501.2; RPRM (margin: AC012501.2; RPRM) | 5.07 | 4.11e-05 | 1.17e-03 |
|  | 6 | 32365491 | 32366096 | PDFSTATSENSEMBLUCSC | 606 | 7 | 34107 | gene | 0.445272 | 0.971855 | BTNL2 (margin: HCG23; BTNL2) | 5.07 | 6.99e-03 | 3.19e-02 |
|  | 20 | 3776212 | 3776483 | PDFSTATSENSEMBLUCSC | 272 | 8 | 25977 | island | 0.632599 | 0.050435 | CDC25B (margin: CDC25B; CENPB) | 5.07 | 3.11e-04 | 4.77e-03 |
|  | 19 | 3932951 | 3933195 | PDFSTATSENSEMBLUCSC | 245 | 5 | 21354 | island | 0.122736 | 0.675741 | NMRK2 (margin: ATCAY; NMRK2) | 5.07 | 7.94e-03 | 3.19e-02 |
|  | 7 | 122526408 | 122526940 | PDFSTATSENSEMBLUCSC | 533 | 9 | 38875 | island | 0.453150 | 0.020894 | CADPS2 (margin: CADPS2) | 5.07 | 4.11e-05 | 1.17e-03 |
|  | 6 | 46702683 | 46703422 | PDFSTATSENSEMBLUCSC | 740 | 12 | 34360 | gene | 0.443560 | 0.966600 | PLA2G7 (margin: ANKRD66; PLA2G7) | 5.07 | 2.22e-05 | 9.05e-04 |
|  | 8 | 144640306 | 144640755 | PDFSTATSENSEMBLUCSC | 450 | 9 | 39483 | gene;island | 0.041254 | 0.602544 | GSDMD (margin: GSDMD; RP11-661A12.5; MROH6) | 5.07 | 4.11e-05 | 1.17e-03 |
|  | 5 | 68787696 | 68788173 | PDFSTATSENSEMBLUCSC | 478 | 9 | 32854 | island | 0.338818 | 0.015133 | OCLN; RP11-241G9.3; GUSBP3 (margin: OCLN; RP11-241G9.3; GUSBP3) | 5.06 | 1.06e-02 | 4.01e-02 |
|  | 6 | 134638742 | 134639020 | PDFSTATSENSEMBLUCSC | 279 | 5 | 34483 | gene | 0.424915 | 0.958450 | SGK1 (margin: SGK1) | 5.06 | 7.94e-03 | 3.19e-02 |
|  | 20 | 50418952 | 50419348 | PDFSTATSENSEMBLUCSC | 397 | 9 | 26257 | island | 0.983176 | 0.560413 | SALL4 (margin: SALL4) | 5.06 | 8.23e-05 | 2.06e-03 |
|  | 4 | 20255061 | 20255343 | PDFSTATSENSEMBLUCSC | 283 | 5 | 30953 | island | 0.799443 | 0.970024 | SLIT2 (margin: SLIT2) | 5.05 | 7.94e-03 | 3.19e-02 |
|  | 22 | 21400059 | 21400184 | PDFSTATSENSEMBLUCSC | 126 | 5 | 26993 | tss;island | 0.439903 | 0.017089 | AC002472.11; AC002472.13; P2RX6P (margin: AC002472.11; AC002472.13; MIR649; P2RX6P) | 5.05 | 7.94e-03 | 3.19e-02 |
|  | 20 | 44098223 | 44098387 | PDFSTATSENSEMBLUCSC | 165 | 6 | 25776 | tss | 0.016406 | 0.389711 | WFDC2; AL031663.1 (margin: WFDC2; AL031663.1) | 5.04 | 2.16e-03 | 1.45e-02 |
|  | 19 | 51225848 | 51226557 | PDFSTATSENSEMBLUCSC | 710 | 6 | 20984 | tss;island | 0.522912 | 0.960149 | CLEC11A (margin: CLEC11A; SHANK1) | 5.04 | 2.16e-03 | 1.45e-02 |
|  | 7 | 27186993 | 27187691 | PDFSTATSENSEMBLUCSC | 699 | 11 | 38349 | island | 0.454791 | 0.975381 | HOXA-AS3; HOXA3; HOXA6 (margin: HOXA-AS3; RP1-170O19.21; HOXA3; HOXA5; HOXA6; HOXA7) | 5.04 | 8.51e-05 | 2.07e-03 |
|  | 7 | 1022471 | 1022806 | PDFSTATSENSEMBLUCSC | 336 | 6 | 37392 | tss | 0.489814 | 0.971559 | CYP2W1 (margin: CYP2W1; COX19) | 5.04 | 2.16e-03 | 1.45e-02 |
|  | 11 | 128775679 | 128775750 | PDFSTATSENSEMBLUCSC | 72 | 6 | 7808 | tss | 0.006616 | 0.127950 | KCNJ5; C11orf45 (margin: KCNJ5; C11orf45) | 5.03 | 2.16e-03 | 1.45e-02 |
|  | 1 | 236228598 | 236228837 | PDFSTATSENSEMBLUCSC | 240 | 6 | 2339 | tss;island | 0.737854 | 0.982001 | NID1 (margin: Y\_RNA; NID1) | 5.02 | 2.16e-03 | 1.45e-02 |
|  | 7 | 35077704 | 35077807 | PDFSTATSENSEMBLUCSC | 104 | 7 | 37534 | tss | 0.198062 | 0.005963 | DPY19L1 (margin: DPY19L1) | 5.02 | 1.17e-03 | 1.13e-02 |
|  | 8 | 12990898 | 12991196 | PDFSTATSENSEMBLUCSC | 299 | 7 | 39560 | tss | 0.162281 | 0.006757 | DLC1 (margin: DLC1) | 5.01 | 5.83e-04 | 6.47e-03 |
|  | 19 | 37341052 | 37341373 | PDFSTATSENSEMBLUCSC | 322 | 8 | 21948 | island | 0.390930 | 0.019365 | ZNF345; ZNF790 (margin: ZNF345; ZNF790; Y\_RNA) | 5.01 | 6.22e-04 | 6.71e-03 |
|  | 22 | 43253517 | 43253559 | PDFSTATSENSEMBLUCSC | 43 | 5 | 27198 | tss;island | 0.913136 | 0.456486 | ARFGAP3; PACSIN2 (margin: ARFGAP3; PACSIN2; AL049758.2) | 5.01 | 7.94e-03 | 3.19e-02 |
|  | 8 | 28560467 | 28560723 | PDFSTATSENSEMBLUCSC | 257 | 5 | 39262 | gene;island | 0.354185 | 0.963616 | EXTL3; EXTL3-AS1 (margin: EXTL3; EXTL3-AS1) | 5.01 | 7.94e-03 | 3.19e-02 |
|  | 5 | 180018465 | 180018735 | PDFSTATSENSEMBLUCSC | 271 | 7 | 33504 | island | 0.381737 | 0.976943 | SCGB3A1 (margin: AC122714.1; SCGB3A1; FLT4) | 5.00 | 5.83e-04 | 6.47e-03 |
|  | 7 | 87105022 | 87105416 | PDFSTATSENSEMBLUCSC | 395 | 5 | 37686 | tss | 0.400445 | 0.960192 | ABCB4 (margin: ABCB4) | 5.00 | 7.94e-03 | 3.19e-02 |
|  | 4 | 95678703 | 95678939 | PDFSTATSENSEMBLUCSC | 237 | 5 | 30504 | tss;island | 0.214372 | 0.021672 | BMPR1B; RP11-168E14.1 (margin: BMPR1B; RP11-168E14.1) | 5.00 | 7.94e-03 | 3.19e-02 |
|  | 8 | 24772137 | 24772513 | PDFSTATSENSEMBLUCSC | 377 | 8 | 39246 | gene;island | 0.673926 | 0.982568 | NEFM; GS1-72M22.1 (margin: NEFM; RP11-624C23.1; GS1-72M22.1) | 5.00 | 1.55e-04 | 2.71e-03 |
|  | 10 | 122216135 | 122216378 | PDFSTATSENSEMBLUCSC | 244 | 7 | 5403 | tss;island | 0.279159 | 0.898233 | PPAPDC1A (margin: PPAPDC1A; AC073587.1) | 4.99 | 1.17e-03 | 1.13e-02 |
|  | 19 | 39989234 | 39989542 | PDFSTATSENSEMBLUCSC | 309 | 6 | 20774 | tss | 0.087637 | 0.668656 | DLL3 (margin: TIMM50; DLL3) | 4.99 | 8.66e-03 | 3.36e-02 |
|  | 11 | 2182457 | 2182795 | PDFSTATSENSEMBLUCSC | 339 | 8 | 7061 | tss | 0.212626 | 0.938469 | INS-IGF2; INS (margin: MIR4686; IGF2; INS-IGF2; INS; TH) | 4.99 | 3.11e-04 | 4.77e-03 |
|  | 1 | 43919949 | 43920264 | PDFSTATSENSEMBLUCSC | 316 | 8 | 1563 | tss | 0.440478 | 0.944286 | SZT2; HYI-AS1; HYI (margin: SZT2; HYI-AS1; SZT2-AS1; HYI) | 4.99 | 4.66e-03 | 2.68e-02 |
|  | 12 | 111284527 | 111284806 | PDFSTATSENSEMBLUCSC | 280 | 8 | 10011 | tss | 0.324939 | 0.926879 | RP1-74B13.2; CCDC63 (margin: RP1-74B13.2; CCDC63; RPL29P25) | 4.98 | 1.55e-04 | 2.71e-03 |
|  | 5 | 140624869 | 140625130 | PDFSTATSENSEMBLUCSC | 262 | 7 | 32369 | tss | 0.428592 | 0.962029 | PCDHB15 (margin: PCDHB18; PCDHB19P; PCDHB15) | 4.98 | 5.83e-04 | 6.47e-03 |
|  | 5 | 174151043 | 174151779 | PDFSTATSENSEMBLUCSC | 737 | 9 | 33416 | island | 0.355032 | 0.006026 | MSX2 (margin: MSX2) | 4.98 | 2.88e-04 | 4.58e-03 |
|  | 2 | 97652129 | 97652491 | PDFSTATSENSEMBLUCSC | 363 | 5 | 24544 | island | 0.409352 | 0.964038 | FAM178B (margin: FAM178B; snoU13) | 4.97 | 7.94e-03 | 3.19e-02 |
|  | 6 | 167535764 | 167536184 | PDFSTATSENSEMBLUCSC | 421 | 7 | 34556 | gene;tss | 0.522759 | 0.952910 | CCR6 (margin: CCR6) | 4.97 | 2.33e-03 | 1.52e-02 |
|  | 20 | 44098223 | 44098409 | PDFSTATSENSEMBLUCSC | 187 | 7 | 26200 | island | 0.019264 | 0.398475 | WFDC2; AL031663.1 (margin: WFDC2; AL031663.1) | 4.97 | 5.83e-04 | 6.47e-03 |
|  | 8 | 40755165 | 40755559 | PDFSTATSENSEMBLUCSC | 395 | 7 | 40240 | island | 0.494995 | 0.017408 | ZMAT4; RP11-465K16.1 (margin: ZMAT4; RP11-465K16.1) | 4.97 | 5.83e-04 | 6.47e-03 |
|  | 4 | 52917271 | 52917567 | PDFSTATSENSEMBLUCSC | 297 | 9 | 30390 | tss | 0.017628 | 0.352360 | SPATA18 (margin: RP11-535C7.1; SPATA18) | 4.97 | 3.99e-03 | 2.47e-02 |
|  | 11 | 843897 | 844686 | PDFSTATSENSEMBLUCSC | 790 | 11 | 6393 | gene;island | 0.450766 | 0.067105 | TSPAN4; POLR2L (margin: EFCAB4A; CD151; TSPAN4; AP006621.8; POLR2L) | 4.97 | 5.39e-05 | 1.47e-03 |
|  | 2 | 223164459 | 223164925 | PDFSTATSENSEMBLUCSC | 467 | 8 | 23049 | gene;tss;island | 0.696724 | 0.980626 | CCDC140; PAX3 (margin: CCDC140; PAX3) | 4.97 | 6.22e-04 | 6.71e-03 |
|  | 18 | 48086162 | 48086680 | PDFSTATSENSEMBLUCSC | 519 | 7 | 19514 | island | 0.371146 | 0.029344 | MAPK4 (margin: MAPK4) | 4.97 | 2.33e-03 | 1.52e-02 |
|  | 6 | 32811495 | 32812708 | PDFSTATSENSEMBLUCSC | 1214 | 44 | 34151 | gene;island | 0.007943 | 0.334668 | XXbac-BPG246D15.8; PSMB9; PSMB8; TAP1 (margin: XXbac-BPG246D15.8; PSMB9; TAP2; PSMB8; TAP1) | 4.97 | 1.30e-09 | 5.51e-07 |
|  | 6 | 134638742 | 134639482 | PDFSTATSENSEMBLUCSC | 741 | 10 | 36495 | island | 0.425329 | 0.951037 | SGK1 (margin: SGK1) | 4.97 | 1.08e-05 | 4.82e-04 |
|  | 11 | 2481857 | 2482929 | PDFSTATSENSEMBLUCSC | 1073 | 17 | 6460 | gene | 0.476189 | 0.954665 | KCNQ1 (margin: KCNQ1) | 4.97 | 4.35e-07 | 5.37e-05 |
|  | 3 | 112359488 | 112359652 | PDFSTATSENSEMBLUCSC | 165 | 6 | 27896 | gene | 0.008889 | 0.196253 | CCDC80 (margin: CCDC80) | 4.97 | 2.16e-03 | 1.45e-02 |
|  | 14 | 101505891 | 101506579 | PDFSTATSENSEMBLUCSC | 689 | 11 | 12625 | tss | 0.322106 | 0.958752 | AL132709.2; MIR376C; MIR654; MIR376A1; MIR300 (margin: MIR494; MIR1193; MIR543; MIR495; AL132709.2; MIR376C; MIR654; MIR376A1; MIR300; MIR1185-1; MIR1185-2; MIR381HG; MIR381; MIR487B; MIR539; MIR889; MIR544A; MIR655; AL132709.3) | 4.97 | 5.24e-03 | 2.98e-02 |
|  | 4 | 40858965 | 40859344 | PDFSTATSENSEMBLUCSC | 380 | 9 | 29990 | gene;island | 0.975364 | 0.598613 | APBB2 (margin: APBB2; snoU13) | 4.97 | 4.11e-05 | 1.17e-03 |
|  | 1 | 236686564 | 236686804 | PDFSTATSENSEMBLUCSC | 241 | 5 | 1098 | gene;tss;island | 0.474351 | 0.013877 | LGALS8; LGALS8-AS1 (margin: LGALS8; LGALS8-AS1) | 4.96 | 7.94e-03 | 3.19e-02 |
|  | 14 | 55034288 | 55034616 | PDFSTATSENSEMBLUCSC | 329 | 6 | 12409 | tss | 0.004361 | 0.163211 | SAMD4A (margin: SAMD4A) | 4.96 | 2.16e-03 | 1.45e-02 |
|  | 13 | 33589962 | 33590495 | PDFSTATSENSEMBLUCSC | 534 | 11 | 11385 | tss;island | 0.485445 | 0.977110 | KL (margin: KL) | 4.96 | 3.40e-05 | 1.17e-03 |
|  | 20 | 57464970 | 57465175 | PDFSTATSENSEMBLUCSC | 206 | 7 | 25474 | gene;tss;island | 0.573327 | 0.969520 | GNAS; RP1-309F20.3 (margin: GNAS; RP1-309F20.3) | 4.95 | 1.17e-03 | 1.13e-02 |
|  | 6 | 94129234 | 94129697 | PDFSTATSENSEMBLUCSC | 464 | 10 | 36331 | island | 0.865728 | 0.077628 | EPHA7 (margin: EPHA7) | 4.95 | 1.50e-03 | 1.41e-02 |
|  | 3 | 61547104 | 61547211 | PDFSTATSENSEMBLUCSC | 108 | 5 | 28423 | tss | 0.131795 | 0.019209 | PTPRG (margin: PTPRG) | 4.95 | 7.94e-03 | 3.19e-02 |
|  | 2 | 17721431 | 17721803 | PDFSTATSENSEMBLUCSC | 373 | 8 | 23200 | tss | 0.071875 | 0.637873 | VSNL1 (margin: VSNL1) | 4.95 | 1.55e-04 | 2.71e-03 |
|  | 6 | 152702330 | 152702660 | PDFSTATSENSEMBLUCSC | 331 | 6 | 34516 | gene | 0.340864 | 0.920874 | SYNE1-AS1; SYNE1 (margin: SYNE1-AS1; SYNE1) | 4.95 | 2.16e-03 | 1.45e-02 |
|  | 11 | 6947353 | 6947773 | PDFSTATSENSEMBLUCSC | 421 | 11 | 8023 | island | 0.314585 | 0.801923 | ZNF215 (margin: OR2D3; ZNF215) | 4.95 | 6.63e-03 | 3.19e-02 |
|  | 15 | 83348985 | 83349864 | PDFSTATSENSEMBLUCSC | 880 | 7 | 13454 | gene;island | 0.560507 | 0.066622 | RP11-752G15.3; AP3B2 (margin: RP11-752G15.3; AP3B2; RP11-752G15.4) | 4.94 | 6.99e-03 | 3.19e-02 |
|  | 11 | 65779192 | 65779847 | PDFSTATSENSEMBLUCSC | 656 | 10 | 8495 | island | 0.439260 | 0.958825 | CST6 (margin: BANF1; CST6; EIF1AD; CATSPER1) | 4.94 | 5.20e-03 | 2.96e-02 |
|  | 16 | 215410 | 216244 | PDFSTATSENSEMBLUCSC | 835 | 9 | 15443 | island | 0.673505 | 0.981374 | HBM; HBZP1 (margin: HBZ; HBM; HBZP1; Z84721.4; HBA2; HBA1) | 4.94 | 8.23e-05 | 2.06e-03 |
|  | 7 | 94953653 | 94953877 | PDFSTATSENSEMBLUCSC | 225 | 5 | 37096 | gene | 0.491455 | 0.962794 | PON1 (margin: PON1) | 4.94 | 7.94e-03 | 3.19e-02 |
|  | 8 | 39441968 | 39442089 | PDFSTATSENSEMBLUCSC | 122 | 5 | 39664 | tss | 0.684996 | 0.982794 | ADAM18 (margin: CTD-2024D23.1; ADAM18) | 4.94 | 7.94e-03 | 3.19e-02 |
|  | 6 | 166876490 | 166877038 | PDFSTATSENSEMBLUCSC | 549 | 8 | 34545 | gene | 0.434865 | 0.976870 | RPS6KA2; RPS6KA2-IT1 (margin: RPS6KA2; RPS6KA2-IT1) | 4.93 | 2.95e-03 | 1.88e-02 |
|  | 5 | 137474700 | 137475115 | PDFSTATSENSEMBLUCSC | 416 | 5 | 31764 | gene | 0.943178 | 0.354188 | NME5; BRD8 (margin: NME5; BRD8) | 4.93 | 7.94e-03 | 3.19e-02 |
|  | 5 | 149669414 | 149669813 | PDFSTATSENSEMBLUCSC | 400 | 5 | 32427 | tss | 0.850101 | 0.258092 | CAMK2A (margin: CAMK2A; ARSI) | 4.92 | 7.94e-03 | 3.19e-02 |
|  | 12 | 75728104 | 75728458 | PDFSTATSENSEMBLUCSC | 355 | 5 | 9880 | tss | 0.027242 | 0.001966 | GLIPR1L1; CAPS2 (margin: GLIPR1L1; CAPS2) | 4.92 | 7.94e-03 | 3.19e-02 |
|  | 12 | 107711872 | 107712210 | PDFSTATSENSEMBLUCSC | 339 | 6 | 10761 | island | 0.143342 | 0.004099 | BTBD11 (margin: BTBD11) | 4.92 | 8.66e-03 | 3.36e-02 |
|  | 3 | 128720242 | 128720433 | PDFSTATSENSEMBLUCSC | 192 | 5 | 28575 | tss;island | 0.560640 | 0.944336 | EFCC1; KIAA1257 (margin: EFCC1; KIAA1257) | 4.91 | 7.94e-03 | 3.19e-02 |
|  | 17 | 40713676 | 40714406 | PDFSTATSENSEMBLUCSC | 731 | 11 | 18340 | island | 0.019914 | 0.373920 | RP11-400F19.8; COASY (margin: RP11-400F19.8; HSD17B1; COASY; MLX; RP11-400F19.6; PSMC3IP) | 4.91 | 3.94e-04 | 5.86e-03 |
|  | 14 | 97058777 | 97059467 | PDFSTATSENSEMBLUCSC | 691 | 8 | 13119 | island | 0.656276 | 0.116143 | RP11-433J8.1 (margin: RP11-433J8.1) | 4.91 | 1.04e-02 | 3.96e-02 |
|  | 8 | 143869138 | 143869473 | PDFSTATSENSEMBLUCSC | 336 | 5 | 39940 | tss | 0.113818 | 0.880452 | RP11-706C16.8; LY6D (margin: RP11-706C16.8; LYNX1; LY6D) | 4.91 | 7.94e-03 | 3.19e-02 |
|  | 5 | 140624869 | 140625389 | PDFSTATSENSEMBLUCSC | 521 | 10 | 33228 | island | 0.429667 | 0.958687 | PCDHB15 (margin: PCDHB18; PCDHB19P; PCDHB15) | 4.91 | 1.08e-05 | 4.82e-04 |
|  | 14 | 101359793 | 101360236 | PDFSTATSENSEMBLUCSC | 444 | 11 | 12615 | tss | 0.395317 | 0.959555 | AL117190.3; MEG8 (margin: MIR433; MIR127; MIR432; MIR136; AL117190.3; MEG8; AL117190.1; RTL1) | 4.90 | 1.40e-03 | 1.33e-02 |
|  | 10 | 22726610 | 22726885 | PDFSTATSENSEMBLUCSC | 276 | 6 | 5605 | island | 0.008728 | 0.246909 | SPAG6 (margin: SPAG6) | 4.90 | 2.16e-03 | 1.45e-02 |
|  | 1 | 236227410 | 236227686 | PDFSTATSENSEMBLUCSC | 277 | 6 | 1096 | gene;island | 0.219113 | 0.970203 | NID1 (margin: Y\_RNA; NID1) | 4.90 | 4.33e-03 | 2.52e-02 |
|  | 1 | 6085799 | 6086275 | PDFSTATSENSEMBLUCSC | 477 | 7 | 1196 | tss | 0.027194 | 0.510438 | KCNAB2 (margin: KCNAB2) | 4.89 | 5.83e-04 | 6.47e-03 |
|  | 6 | 17282284 | 17282859 | PDFSTATSENSEMBLUCSC | 576 | 10 | 33587 | gene;tss;island | 0.903507 | 0.487784 | RBM24 (margin: RBM24) | 4.89 | 3.25e-04 | 4.91e-03 |
|  | 10 | 102090297 | 102090589 | PDFSTATSENSEMBLUCSC | 293 | 5 | 5291 | tss | 0.174052 | 0.855414 | PKD2L1 (margin: PKD2L1; RP11-34D15.2) | 4.89 | 7.94e-03 | 3.19e-02 |
|  | 4 | 46391414 | 46391929 | PDFSTATSENSEMBLUCSC | 516 | 11 | 30372 | tss | 0.910914 | 0.150960 | RP11-436F23.1; GABRA2 (margin: RP11-436F23.1; GABRA2) | 4.89 | 2.45e-03 | 1.59e-02 |
|  | 20 | 21685834 | 21686728 | PDFSTATSENSEMBLUCSC | 895 | 14 | 26042 | island | 0.614267 | 0.973968 | PAX1 (margin: PAX1) | 4.88 | 3.34e-06 | 2.26e-04 |
|  | 20 | 21685834 | 21686293 | PDFSTATSENSEMBLUCSC | 460 | 10 | 25631 | tss | 0.645460 | 0.981050 | PAX1 (margin: PAX1) | 4.88 | 1.30e-04 | 2.71e-03 |
|  | 8 | 97247865 | 97248129 | PDFSTATSENSEMBLUCSC | 265 | 6 | 39825 | tss | 0.015536 | 0.353544 | KB-1043D8.6; UQCRB (margin: KB-1043D8.6; UQCRB; MTERFD1) | 4.88 | 8.66e-03 | 3.36e-02 |
|  | 1 | 31846397 | 31846873 | PDFSTATSENSEMBLUCSC | 477 | 5 | 1446 | tss | 0.375931 | 0.957897 | FABP3 (margin: ZCCHC17; FABP3) | 4.88 | 7.94e-03 | 3.19e-02 |
|  | 11 | 45687263 | 45687494 | PDFSTATSENSEMBLUCSC | 232 | 7 | 7265 | tss;island | 0.256939 | 0.013035 | CHST1 (margin: CHST1) | 4.88 | 1.17e-03 | 1.13e-02 |
|  | 8 | 97505593 | 97505868 | PDFSTATSENSEMBLUCSC | 276 | 7 | 39827 | tss;island | 0.549832 | 0.032320 | SDC2 (margin: SDC2) | 4.88 | 2.33e-03 | 1.52e-02 |
|  | 1 | 224804053 | 224804396 | PDFSTATSENSEMBLUCSC | 344 | 7 | 4244 | island | 0.569051 | 0.028501 | CNIH3; RP11-100E13.1 (margin: CNIH3; RP11-100E13.1) | 4.88 | 1.17e-03 | 1.13e-02 |
|  | 6 | 31685392 | 31685575 | PDFSTATSENSEMBLUCSC | 184 | 7 | 33940 | gene | 0.607218 | 0.027866 | LY6G6F; MEGT1; LY6G6D; C6orf25; LY6G6C (margin: LY6G6F; MEGT1; LY6G6D; C6orf25; XXbac-BPG32J3.20; LY6G6E; LY6G6C; DDAH2) | 4.87 | 5.83e-04 | 6.47e-03 |
|  | 4 | 4387697 | 4387917 | PDFSTATSENSEMBLUCSC | 221 | 6 | 30267 | tss;island | 0.660379 | 0.964272 | NSG1 (margin: NSG1) | 4.87 | 2.16e-03 | 1.45e-02 |
|  | 10 | 132909113 | 132909470 | PDFSTATSENSEMBLUCSC | 358 | 5 | 4840 | gene | 0.251516 | 0.919686 | TCERG1L (margin: TCERG1L) | 4.87 | 7.94e-03 | 3.19e-02 |
|  | 4 | 52917271 | 52917660 | PDFSTATSENSEMBLUCSC | 390 | 10 | 31040 | island | 0.018575 | 0.371187 | SPATA18 (margin: RP11-535C7.1; SPATA18) | 4.86 | 8.93e-03 | 3.45e-02 |
|  | 2 | 145275197 | 145275441 | PDFSTATSENSEMBLUCSC | 245 | 5 | 22890 | gene;island | 0.003127 | 0.101742 | ZEB2-AS1; ZEB2 (margin: ZEB2-AS1; ZEB2) | 4.86 | 7.94e-03 | 3.19e-02 |
|  | 2 | 119599459 | 119600002 | PDFSTATSENSEMBLUCSC | 544 | 6 | 24644 | island | 0.636856 | 0.979329 | EN1 (margin: RP11-19E11.1; EN1) | 4.86 | 2.16e-03 | 1.45e-02 |
|  | 1 | 44870946 | 44871634 | PDFSTATSENSEMBLUCSC | 689 | 8 | 3181 | island | 0.219642 | 0.009174 | RNF220 (margin: RNF220) | 4.86 | 6.22e-04 | 6.71e-03 |
|  | 17 | 46703854 | 46704004 | PDFSTATSENSEMBLUCSC | 151 | 6 | 17468 | tss;island | 0.008080 | 0.131316 | HOXB7; HOXB9 (margin: HOXB-AS4; HOXB7; HOXB8; HOXB9; MIR196A1) | 4.86 | 4.33e-03 | 2.52e-02 |
|  | 5 | 1268644 | 1269309 | PDFSTATSENSEMBLUCSC | 666 | 8 | 31531 | gene;island | 0.401198 | 0.977865 | TERT (margin: TERT) | 4.86 | 2.95e-03 | 1.88e-02 |
|  | 12 | 115130855 | 115131549 | PDFSTATSENSEMBLUCSC | 695 | 8 | 10849 | island | 0.549805 | 0.971863 | (margin: TBX3) | 4.86 | 6.99e-03 | 3.19e-02 |
|  | 1 | 182584068 | 182584578 | PDFSTATSENSEMBLUCSC | 511 | 6 | 4024 | island | 0.661253 | 0.059513 | RP11-317P15.4 (margin: RP11-317P15.4; RGS16) | 4.85 | 2.16e-03 | 1.45e-02 |
|  | 19 | 4534986 | 4535582 | PDFSTATSENSEMBLUCSC | 597 | 11 | 21379 | island | 0.143006 | 0.009940 | PLIN5; CTB-50L17.14; LRG1 (margin: RN7SL121P; PLIN5; CTB-50L17.14; LRG1; SEMA6B) | 4.85 | 7.65e-04 | 8.13e-03 |
|  | 5 | 153857789 | 153858102 | PDFSTATSENSEMBLUCSC | 314 | 6 | 33329 | island | 0.401517 | 0.950425 | HAND1 (margin: HAND1; CTB-158E9.1) | 4.84 | 4.33e-03 | 2.52e-02 |
|  | 7 | 64734093 | 64734749 | PDFSTATSENSEMBLUCSC | 657 | 7 | 38527 | island | 0.275941 | 0.013319 | (margin: AC092685.1) | 4.84 | 1.17e-03 | 1.13e-02 |
|  | 17 | 27045043 | 27045176 | PDFSTATSENSEMBLUCSC | 134 | 5 | 16500 | gene | 0.009760 | 0.319314 | RPL23A; RAB34 (margin: RPL23A; SNORD42B; SNORD4A; SNORD42A; SNORD4B; NEK8; AC010761.14; PROCA1; RAB34; AC010761.8; TLCD1) | 4.84 | 7.94e-03 | 3.19e-02 |
|  | 14 | 102026086 | 102026555 | PDFSTATSENSEMBLUCSC | 470 | 11 | 13158 | island | 0.518049 | 0.977254 | DIO3; DIO3OS (margin: DIO3; DIO3OS) | 4.84 | 5.67e-06 | 3.54e-04 |
|  | 19 | 52511285 | 52511900 | PDFSTATSENSEMBLUCSC | 616 | 12 | 22346 | island | 0.009672 | 0.290116 | ZNF615 (margin: ZNF615; ZNF614) | 4.84 | 3.64e-03 | 2.29e-02 |
|  | 19 | 52511502 | 52511900 | PDFSTATSENSEMBLUCSC | 399 | 10 | 21003 | tss | 0.009672 | 0.302971 | ZNF615 (margin: ZNF615; ZNF614) | 4.84 | 6.84e-03 | 3.19e-02 |
|  | 8 | 99076526 | 99076837 | PDFSTATSENSEMBLUCSC | 312 | 6 | 40483 | island | 0.610609 | 0.965373 | C8orf47 (margin: C8orf47) | 4.84 | 2.16e-03 | 1.45e-02 |
|  | 11 | 2721207 | 2722713 | PDFSTATSENSEMBLUCSC | 1507 | 41 | 6466 | gene;island | 0.467848 | 0.027733 | KCNQ1; KCNQ1OT1 (margin: KCNQ1; KCNQ1OT1) | 4.83 | 3.24e-05 | 1.17e-03 |
|  | 7 | 150945869 | 150946199 | PDFSTATSENSEMBLUCSC | 331 | 5 | 37289 | gene;tss;island | 0.358613 | 0.012071 | RP5-1070G24.2; SMARCD3 (margin: CHPF2; MIR671; RP5-1070G24.2; SMARCD3) | 4.83 | 7.94e-03 | 3.19e-02 |
|  | 6 | 74162051 | 74162142 | PDFSTATSENSEMBLUCSC | 92 | 6 | 35070 | tss | 0.032792 | 0.404815 | MB21D1 (margin: MTO1; RNU6-975P; MB21D1) | 4.83 | 2.16e-03 | 1.45e-02 |
|  | 2 | 19558368 | 19558569 | PDFSTATSENSEMBLUCSC | 202 | 5 | 24102 | island | 0.428487 | 0.955676 | OSR1 (margin: MIR4757; AC092594.1; OSR1) | 4.82 | 7.94e-03 | 3.19e-02 |
|  | 4 | 175443243 | 175443867 | PDFSTATSENSEMBLUCSC | 625 | 6 | 30184 | gene;island | 0.244689 | 0.006125 | HPGD; RP11-440I14.2 (margin: HPGD; RP11-440I14.2) | 4.82 | 2.16e-03 | 1.45e-02 |
|  | 8 | 22926478 | 22926911 | PDFSTATSENSEMBLUCSC | 434 | 10 | 40144 | island | 0.010998 | 0.381637 | RP11-875O11.2; TNFRSF10B; RP11-875O11.3 (margin: RP11-875O11.2; TNFRSF10B; RP11-875O11.3) | 4.82 | 3.89e-03 | 2.43e-02 |
|  | 2 | 122042939 | 122043074 | PDFSTATSENSEMBLUCSC | 136 | 5 | 23601 | tss;island | 0.055975 | 0.660437 | TFCP2L1 (margin: TFCP2L1) | 4.82 | 7.94e-03 | 3.19e-02 |
|  | 20 | 825050 | 825268 | PDFSTATSENSEMBLUCSC | 219 | 5 | 25530 | tss | 0.307883 | 0.012493 | FAM110A; RP11-276A18.2 (margin: FAM110A; RP11-276A18.2; RPS10L) | 4.81 | 7.94e-03 | 3.19e-02 |
|  | 1 | 24513473 | 24514013 | PDFSTATSENSEMBLUCSC | 541 | 8 | 2856 | island | 0.027792 | 0.500168 | IFNLR1 (margin: IFNLR1) | 4.81 | 1.55e-04 | 2.71e-03 |
|  | 17 | 47307954 | 47308460 | PDFSTATSENSEMBLUCSC | 507 | 7 | 18521 | island | 0.287656 | 0.007478 | PHOSPHO1 (margin: ABI3; PHOSPHO1) | 4.81 | 4.08e-03 | 2.47e-02 |
|  | 13 | 79183424 | 79184101 | PDFSTATSENSEMBLUCSC | 678 | 6 | 11796 | island | 0.567422 | 0.971021 | RNF219-AS1 (margin: RNF219-AS1; RP11-52L5.6; POU4F1; RNF219) | 4.81 | 2.16e-03 | 1.45e-02 |
|  | 19 | 36347276 | 36347698 | PDFSTATSENSEMBLUCSC | 423 | 6 | 20716 | tss;island | 0.057667 | 0.656609 | KIRREL2; NPHS1 (margin: KIRREL2; APLP1; NPHS1) | 4.81 | 8.66e-03 | 3.36e-02 |
|  | 10 | 61122307 | 61122570 | PDFSTATSENSEMBLUCSC | 264 | 9 | 4596 | gene | 0.142931 | 0.006156 | FAM13C (margin: FAM13C) | 4.80 | 2.76e-03 | 1.78e-02 |
|  | 10 | 131265059 | 131265435 | PDFSTATSENSEMBLUCSC | 377 | 11 | 5440 | tss | 0.495232 | 0.018989 | MGMT (margin: MGMT) | 4.80 | 3.18e-03 | 2.01e-02 |
|  | 7 | 51384519 | 51384676 | PDFSTATSENSEMBLUCSC | 158 | 6 | 37599 | tss | 0.338481 | 0.023004 | COBL (margin: COBL) | 4.79 | 2.16e-03 | 1.45e-02 |
|  | 1 | 40105664 | 40105764 | PDFSTATSENSEMBLUCSC | 101 | 5 | 1524 | tss;island | 0.813202 | 0.137318 | HEYL (margin: RP1-144F13.3; HEYL) | 4.78 | 7.94e-03 | 3.19e-02 |
|  | 7 | 74020711 | 74021054 | PDFSTATSENSEMBLUCSC | 344 | 5 | 38580 | island | 0.778451 | 0.091797 | (margin: GTF2IRD1) | 4.78 | 7.94e-03 | 3.19e-02 |
|  | 19 | 50861774 | 50862121 | PDFSTATSENSEMBLUCSC | 348 | 5 | 20174 | gene;island | 0.575607 | 0.971541 | NR1H2; NAPSA (margin: NR1H2; NAPSA; CTB-191K22.6) | 4.78 | 7.94e-03 | 3.19e-02 |
|  | 20 | 43374239 | 43374406 | PDFSTATSENSEMBLUCSC | 168 | 8 | 25763 | tss;island | 0.418571 | 0.044646 | KCNK15; RP11-445H22.4 (margin: KCNK15; RP11-445H22.4; RIMS4) | 4.78 | 3.11e-04 | 4.77e-03 |
|  | 15 | 83378614 | 83378895 | PDFSTATSENSEMBLUCSC | 282 | 9 | 14385 | island | 0.101681 | 0.007237 | AP3B2; AC105339.1 (margin: AP3B2; AC105339.1) | 4.78 | 4.94e-04 | 6.47e-03 |
|  | 11 | 2444462 | 2445442 | PDFSTATSENSEMBLUCSC | 981 | 17 | 7072 | tss;island | 0.281698 | 0.881357 | TRPM5 (margin: TRPM5) | 4.78 | 1.67e-07 | 2.60e-05 |
|  | 18 | 580188 | 580334 | PDFSTATSENSEMBLUCSC | 147 | 6 | 19151 | tss | 0.466927 | 0.963835 | CETN1 (margin: CETN1; RN7SKP146) | 4.78 | 4.33e-03 | 2.52e-02 |
|  | 8 | 22014425 | 22014724 | PDFSTATSENSEMBLUCSC | 300 | 8 | 39582 | tss;island | 0.462715 | 0.046217 | SFTPC; LGI3 (margin: SFTPC; BMP1; LGI3) | 4.78 | 1.55e-04 | 2.71e-03 |
|  | 3 | 46853850 | 46854311 | PDFSTATSENSEMBLUCSC | 462 | 7 | 29061 | island | 0.361665 | 0.966916 | PRSS50; PRSS45; PRSS44 (margin: PRSS50; PRSS45; PRSS44) | 4.77 | 1.17e-03 | 1.13e-02 |
|  | 7 | 140773422 | 140774161 | PDFSTATSENSEMBLUCSC | 740 | 6 | 38967 | island | 0.700704 | 0.049542 | TMEM178B (margin: TMEM178B) | 4.77 | 2.16e-03 | 1.45e-02 |
|  | 16 | 30124803 | 30125403 | PDFSTATSENSEMBLUCSC | 601 | 8 | 15180 | tss | 0.098766 | 0.779381 | GDPD3; MAPK3 (margin: RP11-455F5.3; RP11-455F5.4; GDPD3; MAPK3) | 4.77 | 6.99e-03 | 3.19e-02 |
|  | 17 | 25798741 | 25799018 | PDFSTATSENSEMBLUCSC | 278 | 5 | 17205 | tss | 0.140731 | 0.819446 | KSR1 (margin: KSR1; RP11-720N19.2) | 4.77 | 7.94e-03 | 3.19e-02 |
|  | 6 | 94129412 | 94129697 | PDFSTATSENSEMBLUCSC | 286 | 8 | 35125 | tss | 0.814441 | 0.127732 | EPHA7 (margin: EPHA7) | 4.77 | 1.04e-02 | 3.96e-02 |
|  | 3 | 134369919 | 134370241 | PDFSTATSENSEMBLUCSC | 323 | 9 | 28607 | tss | 0.274664 | 0.896400 | EPHB1; KY (margin: EPHB1; KY) | 4.76 | 4.11e-05 | 1.17e-03 |
|  | 13 | 74708078 | 74708194 | PDFSTATSENSEMBLUCSC | 117 | 6 | 11477 | tss | 0.116825 | 0.006948 | KLF12 (margin: KLF12) | 4.76 | 2.16e-03 | 1.45e-02 |
|  | 4 | 141489963 | 141490428 | PDFSTATSENSEMBLUCSC | 466 | 10 | 30608 | tss | 0.624971 | 0.978035 | UCP1 (margin: RN7SL152P; UCP1) | 4.75 | 1.08e-05 | 4.82e-04 |
|  | 10 | 31609882 | 31610093 | PDFSTATSENSEMBLUCSC | 212 | 8 | 4554 | gene;island | 0.049984 | 0.642516 | ZEB1; ZEB1-AS1 (margin: ZEB1; ZEB1-AS1) | 4.75 | 1.55e-04 | 2.71e-03 |
|  | 6 | 27840105 | 27840257 | PDFSTATSENSEMBLUCSC | 153 | 6 | 34727 | tss | 0.014174 | 0.000516 | HIST1H3I; HIST1H4L (margin: HIST1H2AL; HIST1H2BPS2; HIST1H1B; HIST1H3I; HIST1H4L) | 4.75 | 2.16e-03 | 1.45e-02 |
|  | 2 | 145147129 | 145147558 | PDFSTATSENSEMBLUCSC | 430 | 5 | 22889 | gene;island | 0.338227 | 0.821686 | ZEB2 (margin: ZEB2) | 4.75 | 7.94e-03 | 3.19e-02 |
|  | 11 | 2721243 | 2722713 | PDFSTATSENSEMBLUCSC | 1471 | 40 | 7077 | tss | 0.477564 | 0.027740 | KCNQ1; KCNQ1OT1 (margin: KCNQ1; KCNQ1OT1) | 4.75 | 5.98e-05 | 1.62e-03 |
|  | 14 | 104195181 | 104195666 | PDFSTATSENSEMBLUCSC | 486 | 5 | 12243 | gene;island | 0.960675 | 0.590539 | ZFYVE21 (margin: ZFYVE21; Y\_RNA; PPP1R13B) | 4.75 | 7.94e-03 | 3.19e-02 |
|  | 6 | 167263025 | 167263454 | PDFSTATSENSEMBLUCSC | 430 | 6 | 34555 | gene | 0.308054 | 0.906135 | RPS6KA2 (margin: RPS6KA2; RP11-514O12.4) | 4.73 | 2.16e-03 | 1.45e-02 |
|  | 12 | 12848977 | 12849748 | PDFSTATSENSEMBLUCSC | 772 | 11 | 10250 | island | 0.074570 | 0.008318 | GPR19 (margin: RP11-180M15.3; GPR19) | 4.73 | 5.53e-04 | 6.47e-03 |
|  | 13 | 111972854 | 111972997 | PDFSTATSENSEMBLUCSC | 144 | 6 | 11542 | tss | 0.583758 | 0.953972 | TEX29 (margin: TEX29) | 4.73 | 2.16e-03 | 1.45e-02 |
|  | 10 | 50323706 | 50323832 | PDFSTATSENSEMBLUCSC | 127 | 5 | 5070 | tss;island | 0.435328 | 0.951864 | VSTM4 (margin: FAM170B-AS1; VSTM4) | 4.73 | 7.94e-03 | 3.19e-02 |
|  | 8 | 49468684 | 49468988 | PDFSTATSENSEMBLUCSC | 305 | 6 | 40283 | island | 0.552412 | 0.969892 | RP11-770E5.1; RP11-567J20.2 (margin: RP11-770E5.1; RP11-567J20.2) | 4.72 | 2.16e-03 | 1.45e-02 |
|  | 6 | 43276478 | 43276781 | PDFSTATSENSEMBLUCSC | 304 | 9 | 36161 | island | 0.419961 | 0.962723 | RP11-480N24.3; CRIP3; ZNF318 (margin: SLC22A7; RP11-480N24.3; CRIP3; ZNF318) | 4.72 | 4.11e-05 | 1.17e-03 |
|  | 11 | 14913752 | 14914012 | PDFSTATSENSEMBLUCSC | 261 | 8 | 7166 | tss | 0.149965 | 0.005475 | CYP2R1 (margin: CYP2R1) | 4.72 | 1.09e-03 | 1.10e-02 |
|  | 5 | 133451237 | 133451474 | PDFSTATSENSEMBLUCSC | 238 | 7 | 32287 | tss | 0.090258 | 0.814876 | TCF7 (margin: TCF7) | 4.72 | 1.17e-03 | 1.13e-02 |
|  | 19 | 7741454 | 7741838 | PDFSTATSENSEMBLUCSC | 385 | 5 | 20404 | tss | 0.055868 | 0.831206 | C19orf59; CTD-3214H19.16 (margin: CTD-3214H19.12; RETN; C19orf59; CTD-3214H19.16; TRAPPC5; FCER2) | 4.71 | 7.94e-03 | 3.19e-02 |
|  | 19 | 7741602 | 7741955 | PDFSTATSENSEMBLUCSC | 354 | 5 | 21462 | island | 0.055868 | 0.908037 | C19orf59; CTD-3214H19.16 (margin: CTD-3214H19.12; RETN; C19orf59; CTD-3214H19.16; TRAPPC5; FCER2) | 4.71 | 7.94e-03 | 3.19e-02 |
|  | 2 | 233497957 | 233498164 | PDFSTATSENSEMBLUCSC | 208 | 6 | 23953 | tss | 0.378354 | 0.024925 | EFHD1 (margin: EFHD1) | 4.71 | 2.16e-03 | 1.45e-02 |
|  | 7 | 128828120 | 128828765 | PDFSTATSENSEMBLUCSC | 646 | 9 | 38907 | island | 0.537283 | 0.968194 | SMO (margin: SMO) | 4.71 | 2.88e-04 | 4.58e-03 |
|  | 12 | 118407009 | 118407487 | PDFSTATSENSEMBLUCSC | 479 | 6 | 10045 | tss;island | 0.432803 | 0.027135 | KSR2 (margin: KSR2) | 4.71 | 8.66e-03 | 3.36e-02 |
|  | 11 | 94134446 | 94135029 | PDFSTATSENSEMBLUCSC | 584 | 10 | 8737 | island | 0.593387 | 0.981610 | GPR83 (margin: GPR83) | 4.71 | 1.05e-03 | 1.08e-02 |
|  | 20 | 30605656 | 30606095 | PDFSTATSENSEMBLUCSC | 440 | 5 | 25387 | gene;island | 0.368351 | 0.024602 | CCM2L (margin: RNA5SP481; CCM2L; RP1-310O13.7) | 4.71 | 7.94e-03 | 3.19e-02 |
|  | 13 | 20806477 | 20806813 | PDFSTATSENSEMBLUCSC | 337 | 8 | 11331 | tss;island | 0.556864 | 0.984305 | GJB6 (margin: GJB6) | 4.71 | 1.55e-04 | 2.71e-03 |
|  | 2 | 233497957 | 233498223 | PDFSTATSENSEMBLUCSC | 267 | 7 | 23068 | gene;island | 0.369572 | 0.026663 | EFHD1 (margin: EFHD1) | 4.70 | 5.83e-04 | 6.47e-03 |
|  | 11 | 63258509 | 63258803 | PDFSTATSENSEMBLUCSC | 295 | 8 | 8369 | island | 0.557900 | 0.966761 | HRASLS5 (margin: HRASLS5) | 4.70 | 6.99e-03 | 3.19e-02 |
|  | 15 | 90039409 | 90039913 | PDFSTATSENSEMBLUCSC | 505 | 10 | 14437 | island | 0.594427 | 0.056155 | RHCG (margin: RHCG; LINC00928) | 4.70 | 1.08e-05 | 4.82e-04 |
|  | 15 | 90039805 | 90039913 | PDFSTATSENSEMBLUCSC | 109 | 6 | 13888 | tss | 0.617174 | 0.058457 | RHCG (margin: RHCG; LINC00928) | 4.70 | 2.16e-03 | 1.45e-02 |
|  | 5 | 1257034 | 1257221 | PDFSTATSENSEMBLUCSC | 188 | 5 | 31530 | gene;island | 0.493279 | 0.964162 | TERT (margin: SLC6A18; TERT) | 4.70 | 7.94e-03 | 3.19e-02 |
|  | 1 | 20878760 | 20879029 | PDFSTATSENSEMBLUCSC | 270 | 5 | 2795 | island | 0.709544 | 0.983762 | FAM43B (margin: FAM43B) | 4.70 | 7.94e-03 | 3.19e-02 |
|  | 21 | 42218932 | 42219299 | PDFSTATSENSEMBLUCSC | 368 | 8 | 26686 | island | 0.591361 | 0.986267 | DSCAM (margin: DSCAM) | 4.70 | 2.95e-03 | 1.88e-02 |
|  | 2 | 1926366 | 1926907 | PDFSTATSENSEMBLUCSC | 542 | 6 | 22541 | gene | 0.094606 | 0.649657 | MYT1L (margin: MYT1L) | 4.70 | 8.66e-03 | 3.36e-02 |
|  | 1 | 161067636 | 161068387 | PDFSTATSENSEMBLUCSC | 752 | 12 | 3898 | island | 0.318415 | 0.006583 | KLHDC9; PFDN2 (margin: RP11-544M22.8; KLHDC9; PVRL4; PFDN2) | 4.70 | 5.56e-03 | 3.16e-02 |
|  | 17 | 62777648 | 62777777 | PDFSTATSENSEMBLUCSC | 130 | 6 | 16734 | gene;tss;island | 0.060473 | 0.609135 | hsa-mir-6080; PLEKHM1P (margin: hsa-mir-6080; RP11-927P21.4; PLEKHM1P) | 4.70 | 2.16e-03 | 1.45e-02 |
|  | 18 | 580188 | 580435 | PDFSTATSENSEMBLUCSC | 248 | 7 | 19347 | island | 0.480626 | 0.963675 | CETN1 (margin: CETN1; RN7SKP146) | 4.69 | 2.33e-03 | 1.52e-02 |
|  | 5 | 128300967 | 128301185 | PDFSTATSENSEMBLUCSC | 219 | 6 | 32260 | tss | 0.155037 | 0.011585 | SLC27A6 (margin: SLC27A6) | 4.69 | 2.16e-03 | 1.45e-02 |
|  | 1 | 43389643 | 43389888 | PDFSTATSENSEMBLUCSC | 246 | 5 | 3154 | island | 0.449313 | 0.030480 | SLC2A1 (margin: SLC2A1) | 4.69 | 7.94e-03 | 3.19e-02 |
|  | 13 | 113633379 | 113634042 | PDFSTATSENSEMBLUCSC | 664 | 8 | 11263 | gene;island | 0.208671 | 0.896625 | MCF2L (margin: MCF2L; MCF2L-AS1) | 4.69 | 6.22e-04 | 6.71e-03 |
|  | 6 | 24911001 | 24911615 | PDFSTATSENSEMBLUCSC | 615 | 12 | 35497 | island | 0.522011 | 0.055435 | FAM65B (margin: FAM65B) | 4.68 | 3.33e-05 | 1.17e-03 |
|  | 4 | 141489799 | 141490428 | PDFSTATSENSEMBLUCSC | 630 | 12 | 31327 | island | 0.624971 | 0.977642 | UCP1 (margin: RN7SL152P; UCP1) | 4.68 | 7.40e-07 | 7.21e-05 |
|  | 4 | 8271196 | 8271414 | PDFSTATSENSEMBLUCSC | 219 | 6 | 30296 | tss | 0.430225 | 0.946298 | HTRA3 (margin: HTRA3) | 4.68 | 2.16e-03 | 1.45e-02 |
|  | 13 | 74707860 | 74708194 | PDFSTATSENSEMBLUCSC | 335 | 8 | 11779 | island | 0.143541 | 0.006948 | KLF12 (margin: KLF12) | 4.68 | 4.66e-03 | 2.68e-02 |
|  | 1 | 197880384 | 197880923 | PDFSTATSENSEMBLUCSC | 540 | 6 | 2156 | tss;island | 0.418197 | 0.897991 | LHX9 (margin: C1orf53; LHX9) | 4.68 | 2.16e-03 | 1.45e-02 |
|  | 4 | 141347993 | 141349096 | PDFSTATSENSEMBLUCSC | 1104 | 16 | 31325 | island | 0.175649 | 0.006070 | CLGN (margin: CLGN) | 4.68 | 3.92e-04 | 5.86e-03 |
|  | 7 | 1250038 | 1251125 | PDFSTATSENSEMBLUCSC | 1088 | 17 | 38089 | island | 0.112095 | 0.773256 | (margin: ) | 4.68 | 1.21e-05 | 5.36e-04 |
|  | 3 | 120626881 | 120627170 | PDFSTATSENSEMBLUCSC | 290 | 6 | 29392 | island | 0.616596 | 0.046476 | STXBP5L (margin: STXBP5L) | 4.67 | 2.16e-03 | 1.45e-02 |
|  | 6 | 36237982 | 36238230 | PDFSTATSENSEMBLUCSC | 249 | 7 | 34935 | tss | 0.032196 | 0.459046 | PNPLA1 (margin: PNPLA1) | 4.67 | 5.83e-04 | 6.47e-03 |
|  | 11 | 66034681 | 66035276 | PDFSTATSENSEMBLUCSC | 596 | 17 | 6727 | gene | 0.480074 | 0.050703 | KLC2; RAB1B; RP11-867G23.1; RP11-867G23.2 (margin: KLC2; RAB1B; CNIH2; RP11-755F10.3; RP11-867G23.1; RP11-867G23.2; RP11-867G23.3; RP11-867G23.4) | 4.67 | 2.52e-04 | 4.14e-03 |
|  | 1 | 149144820 | 149145243 | PDFSTATSENSEMBLUCSC | 424 | 5 | 3704 | island | 0.228465 | 0.893290 | RNU1-114P (margin: RNU1-114P) | 4.67 | 7.94e-03 | 3.19e-02 |
|  | 1 | 68515788 | 68516453 | PDFSTATSENSEMBLUCSC | 666 | 11 | 574 | gene | 0.674010 | 0.978243 | GNG12-AS1; DIRAS3; ARL5AP3 (margin: GNG12-AS1; RP4-609E1.2; AL157407.1; DIRAS3; ARL5AP3) | 4.67 | 1.04e-03 | 1.08e-02 |
|  | 21 | 45705175 | 45705742 | PDFSTATSENSEMBLUCSC | 568 | 10 | 26582 | tss;island | 0.569780 | 0.984970 | AIRE (margin: AIRE) | 4.67 | 1.05e-03 | 1.08e-02 |
|  | 4 | 46391259 | 46391929 | PDFSTATSENSEMBLUCSC | 671 | 13 | 29995 | gene;island | 0.910914 | 0.215073 | RP11-436F23.1; GABRA2 (margin: RP11-436F23.1; GABRA2) | 4.67 | 1.91e-03 | 1.45e-02 |
|  | 11 | 46354562 | 46354635 | PDFSTATSENSEMBLUCSC | 74 | 6 | 7275 | tss | 0.771264 | 0.987943 | DGKZ (margin: CREB3L1; DGKZ) | 4.66 | 2.16e-03 | 1.45e-02 |
|  | 6 | 43276570 | 43276781 | PDFSTATSENSEMBLUCSC | 212 | 8 | 34996 | tss | 0.405866 | 0.955916 | RP11-480N24.3; CRIP3; ZNF318 (margin: SLC22A7; RP11-480N24.3; CRIP3; ZNF318) | 4.66 | 1.55e-04 | 2.71e-03 |
|  | 15 | 41056021 | 41056252 | PDFSTATSENSEMBLUCSC | 232 | 5 | 13594 | tss | 0.200461 | 0.011103 | GCHFR (margin: RP11-532F12.6; GCHFR; C15orf62; RMDN3; DNAJC17) | 4.66 | 7.94e-03 | 3.19e-02 |
|  | 10 | 115998843 | 115999174 | PDFSTATSENSEMBLUCSC | 332 | 6 | 6152 | island | 0.151883 | 0.009628 | VWA2 (margin: TDRD1; VWA2; CTB-1144G6.5) | 4.66 | 8.66e-03 | 3.36e-02 |
|  | 17 | 40805728 | 40805956 | PDFSTATSENSEMBLUCSC | 229 | 5 | 18346 | island | 0.919291 | 0.314589 | (margin: ATP5LP7; HMGB3P27; TUBG2) | 4.66 | 7.94e-03 | 3.19e-02 |
|  | 10 | 114135847 | 114136073 | PDFSTATSENSEMBLUCSC | 227 | 6 | 4762 | gene | 0.157526 | 0.794079 | ACSL5 (margin: ACSL5) | 4.65 | 2.16e-03 | 1.45e-02 |
|  | 6 | 21666391 | 21666666 | PDFSTATSENSEMBLUCSC | 276 | 5 | 34664 | tss | 0.280060 | 0.917560 | LINC00340 (margin: LINC00340) | 4.65 | 7.94e-03 | 3.19e-02 |
|  | 10 | 134977981 | 134978147 | PDFSTATSENSEMBLUCSC | 167 | 5 | 4887 | gene;island | 0.484765 | 0.963840 | KNDC1 (margin: KNDC1) | 4.65 | 7.94e-03 | 3.19e-02 |
|  | 6 | 150463775 | 150464265 | PDFSTATSENSEMBLUCSC | 491 | 8 | 36568 | island | 0.040524 | 0.550569 | PPP1R14C (margin: PPP1R14C) | 4.65 | 1.86e-03 | 1.45e-02 |
|  | 10 | 123357993 | 123358317 | PDFSTATSENSEMBLUCSC | 325 | 6 | 5406 | tss;island | 0.043592 | 0.486375 | FGFR2 (margin: FGFR2) | 4.64 | 2.16e-03 | 1.45e-02 |
|  | 8 | 15397637 | 15398333 | PDFSTATSENSEMBLUCSC | 697 | 8 | 40099 | island | 0.314539 | 0.009705 | TUSC3 (margin: TUSC3) | 4.64 | 1.04e-02 | 3.96e-02 |
|  | 17 | 72889323 | 72890001 | PDFSTATSENSEMBLUCSC | 679 | 11 | 18728 | island | 0.513969 | 0.953032 | FADS6 (margin: FADS6) | 4.64 | 8.51e-05 | 2.07e-03 |
|  | 17 | 73874443 | 73874790 | PDFSTATSENSEMBLUCSC | 348 | 7 | 18765 | island | 0.012213 | 0.389366 | RP11-552F3.9; TRIM47; TRIM65 (margin: RP11-552F3.9; TRIM47; TRIM65) | 4.64 | 2.33e-03 | 1.52e-02 |
|  | 4 | 156297854 | 156298427 | PDFSTATSENSEMBLUCSC | 574 | 12 | 31390 | island | 0.736511 | 0.039893 | AC097467.2; MAP9 (margin: AC097467.2; RP11-27G13.5; MAP9; RP11-27G13.3; RP11-27G13.4) | 4.64 | 2.32e-03 | 1.52e-02 |
|  | 7 | 82792053 | 82792411 | PDFSTATSENSEMBLUCSC | 359 | 9 | 38625 | island | 0.148853 | 0.010428 | PCLO (margin: PCLO) | 4.63 | 1.65e-04 | 2.80e-03 |
|  | 10 | 129533998 | 129534411 | PDFSTATSENSEMBLUCSC | 414 | 5 | 6248 | island | 0.453016 | 0.893488 | FOXI2; AL391005.1 (margin: FOXI2; AL391005.1) | 4.63 | 7.94e-03 | 3.19e-02 |
|  | 6 | 168197410 | 168198163 | PDFSTATSENSEMBLUCSC | 754 | 9 | 36648 | island | 0.573375 | 0.976432 | AL009178.1; C6orf123 (margin: AL009178.1; C6orf123) | 4.63 | 7.82e-04 | 8.17e-03 |
|  | 6 | 168197625 | 168198163 | PDFSTATSENSEMBLUCSC | 539 | 7 | 35343 | tss | 0.573375 | 0.963023 | AL009178.1; C6orf123 (margin: AL009178.1; C6orf123) | 4.63 | 6.99e-03 | 3.19e-02 |
|  | 2 | 192015279 | 192015918 | PDFSTATSENSEMBLUCSC | 640 | 10 | 22962 | gene | 0.294069 | 0.016357 | STAT4 (margin: STAT4) | 4.63 | 1.08e-05 | 4.82e-04 |
|  | 6 | 10886946 | 10887047 | PDFSTATSENSEMBLUCSC | 102 | 7 | 34633 | tss | 0.351650 | 0.971862 | RP11-637O19.3; SYCP2L (margin: RP11-637O19.3; SYCP2L; RP11-637O19.2; GCM2) | 4.63 | 1.17e-03 | 1.13e-02 |
|  | 5 | 140305947 | 140306249 | PDFSTATSENSEMBLUCSC | 303 | 8 | 32357 | tss | 0.957368 | 0.507255 | PCDHA1; PCDHA2; PCDHA3; PCDHA4; PCDHA5; PCDHA6; PCDHA7; PCDHA8; PCDHA9; PCDHA10; PCDHA11; PCDHA12; PCDHA13; PCDHAC1 (margin: PCDHA1; PCDHA2; PCDHA3; PCDHA4; PCDHA5; PCDHA6; PCDHA7; PCDHA8; PCDHA9; PCDHA10; PCDHA11; PCDHA12; PCDHA13; PCDHAC1) | 4.62 | 1.55e-04 | 2.71e-03 |
|  | 10 | 135049991 | 135050355 | PDFSTATSENSEMBLUCSC | 365 | 13 | 5455 | tss;island | 0.656817 | 0.984329 | VENTX (margin: KNDC1; UTF1; VENTX; MIR202) | 4.62 | 3.65e-06 | 2.42e-04 |
|  | 7 | 93519855 | 93520036 | PDFSTATSENSEMBLUCSC | 182 | 6 | 37091 | gene | 0.787939 | 0.218208 | GNGT1; AC002076.10; TFPI2 (margin: GNGT1; AC002076.10; TFPI2) | 4.62 | 2.16e-03 | 1.45e-02 |
|  | 17 | 66194978 | 66195485 | PDFSTATSENSEMBLUCSC | 508 | 5 | 16740 | gene | 0.609403 | 0.978534 | LOC440461 (margin: RP11-147L13.8; LOC440461) | 4.61 | 7.94e-03 | 3.19e-02 |
|  | 8 | 33457226 | 33457822 | PDFSTATSENSEMBLUCSC | 597 | 8 | 40215 | island | 0.498978 | 0.965104 | DUSP26 (margin: RP11-317N12.1; DUSP26) | 4.61 | 1.55e-04 | 2.71e-03 |
|  | 3 | 45837898 | 45838338 | PDFSTATSENSEMBLUCSC | 441 | 10 | 29051 | island | 0.671566 | 0.981258 | SLC6A20 (margin: SLC6A20) | 4.61 | 1.50e-03 | 1.41e-02 |
|  | 3 | 45838037 | 45838338 | PDFSTATSENSEMBLUCSC | 302 | 8 | 28271 | tss | 0.632896 | 0.979962 | SLC6A20 (margin: SLC6A20) | 4.61 | 1.04e-02 | 3.96e-02 |
|  | 6 | 32297615 | 32297861 | PDFSTATSENSEMBLUCSC | 247 | 5 | 34101 | gene | 0.780550 | 0.973407 | C6orf10 (margin: HNRNPA1P2; C6orf10) | 4.61 | 7.94e-03 | 3.19e-02 |
|  | 15 | 41056021 | 41056369 | PDFSTATSENSEMBLUCSC | 349 | 7 | 14045 | island | 0.223257 | 0.016246 | GCHFR (margin: RP11-532F12.6; GCHFR; C15orf62; RMDN3; DNAJC17) | 4.61 | 5.83e-04 | 6.47e-03 |
|  | 16 | 15595854 | 15596423 | PDFSTATSENSEMBLUCSC | 570 | 9 | 14675 | gene | 0.395222 | 0.938668 | RP11-1021N1.1; C16orf45 (margin: RP11-1021N1.1; C16orf45) | 4.60 | 2.76e-03 | 1.78e-02 |
|  | 11 | 30607888 | 30608198 | PDFSTATSENSEMBLUCSC | 311 | 10 | 8155 | island | 0.352776 | 0.019465 | RP5-1024C24.1; MPPED2 (margin: RP5-1024C24.1; MPPED2) | 4.60 | 1.08e-05 | 4.82e-04 |
|  | 7 | 45961074 | 45961569 | PDFSTATSENSEMBLUCSC | 496 | 17 | 37587 | tss;island | 0.971996 | 0.540501 | IGFBP3 (margin: IGFBP3) | 4.60 | 6.06e-03 | 3.19e-02 |
|  | 10 | 134918671 | 134918974 | PDFSTATSENSEMBLUCSC | 304 | 5 | 4885 | gene;island | 0.083218 | 0.813265 | GPR123 (margin: GPR123) | 4.60 | 7.94e-03 | 3.19e-02 |
|  | 2 | 25391656 | 25391911 | PDFSTATSENSEMBLUCSC | 256 | 10 | 23231 | tss | 0.510038 | 0.969985 | POMC (margin: RP11-509E16.1; POMC) | 4.60 | 1.08e-05 | 4.82e-04 |
|  | 13 | 114875001 | 114875412 | PDFSTATSENSEMBLUCSC | 412 | 5 | 11314 | gene;island | 0.896972 | 0.385201 | RASA3; RASA3-IT1 (margin: RASA3; RASA3-IT1) | 4.60 | 7.94e-03 | 3.19e-02 |
|  | 11 | 134145889 | 134146324 | PDFSTATSENSEMBLUCSC | 436 | 9 | 8981 | island | 0.496692 | 0.972447 | GLB1L3 (margin: ACAD8; GLB1L3) | 4.59 | 2.88e-04 | 4.58e-03 |
|  | 16 | 56622951 | 56623549 | PDFSTATSENSEMBLUCSC | 599 | 10 | 15946 | island | 0.636101 | 0.983984 | MT3 (margin: MT3) | 4.59 | 1.08e-05 | 4.82e-04 |
|  | 4 | 114683176 | 114683375 | PDFSTATSENSEMBLUCSC | 200 | 7 | 30556 | tss;island | 0.054651 | 0.582743 | CAMK2D (margin: CAMK2D) | 4.59 | 5.83e-04 | 6.47e-03 |
|  | 20 | 3776212 | 3776357 | PDFSTATSENSEMBLUCSC | 146 | 7 | 25568 | tss | 0.618304 | 0.063233 | CDC25B (margin: CDC25B; CENPB) | 4.59 | 1.17e-03 | 1.13e-02 |
|  | 4 | 25031951 | 25032564 | PDFSTATSENSEMBLUCSC | 614 | 9 | 30957 | island | 0.406595 | 0.010702 | LGI2 (margin: LGI2) | 4.59 | 1.23e-03 | 1.18e-02 |
|  | 12 | 130646022 | 130646497 | PDFSTATSENSEMBLUCSC | 476 | 6 | 10118 | tss;island | 0.108050 | 0.594564 | FZD10; FZD10-AS1 (margin: FZD10; FZD10-AS1) | 4.58 | 2.16e-03 | 1.45e-02 |
|  | 3 | 32859377 | 32859587 | PDFSTATSENSEMBLUCSC | 211 | 7 | 28968 | island | 0.550407 | 0.978075 | TRIM71 (margin: TRIM71) | 4.58 | 5.83e-04 | 6.47e-03 |
|  | 12 | 24715478 | 24715564 | PDFSTATSENSEMBLUCSC | 87 | 6 | 9605 | tss;island | 0.529605 | 0.041805 | RP11-444D3.1 (margin: RP11-444D3.1; LINC00477) | 4.58 | 2.16e-03 | 1.45e-02 |
|  | 6 | 84233917 | 84234463 | PDFSTATSENSEMBLUCSC | 547 | 8 | 34403 | gene | 0.497537 | 0.950523 | PRSS35 (margin: PRSS35) | 4.58 | 1.04e-02 | 3.96e-02 |
|  | 2 | 84743142 | 84743743 | PDFSTATSENSEMBLUCSC | 602 | 12 | 24469 | island | 0.443367 | 0.027236 | DNAH6 (margin: DNAH6) | 4.58 | 6.81e-03 | 3.19e-02 |
|  | 5 | 135415693 | 135416613 | PDFSTATSENSEMBLUCSC | 921 | 16 | 33146 | island | 0.538887 | 0.970870 | VTRNA2-1 (margin: VTRNA2-1) | 4.57 | 2.33e-08 | 5.67e-06 |
|  | 13 | 36944433 | 36944649 | PDFSTATSENSEMBLUCSC | 217 | 7 | 11395 | tss | 0.734431 | 0.984903 | SPG20OS; SPG20 (margin: SPG20OS; SPG20) | 4.57 | 4.08e-03 | 2.47e-02 |
|  | 6 | 28984234 | 28985069 | PDFSTATSENSEMBLUCSC | 836 | 24 | 35610 | island | 0.014891 | 0.325226 | (margin: ZNF311; OR2AD1P) | 4.57 | 1.25e-05 | 5.44e-04 |
|  | 4 | 619162 | 619392 | PDFSTATSENSEMBLUCSC | 231 | 6 | 30723 | island | 0.257760 | 0.874224 | PDE6B (margin: PDE6B) | 4.57 | 2.16e-03 | 1.45e-02 |
|  | 7 | 128828120 | 128828599 | PDFSTATSENSEMBLUCSC | 480 | 8 | 37891 | tss | 0.541563 | 0.966394 | SMO (margin: SMO) | 4.57 | 1.09e-03 | 1.10e-02 |
|  | 10 | 30026015 | 30026143 | PDFSTATSENSEMBLUCSC | 129 | 5 | 5010 | tss;island | 0.345416 | 0.020275 | SVIL (margin: SVIL) | 4.57 | 7.94e-03 | 3.19e-02 |
|  | 10 | 88126273 | 88126306 | PDFSTATSENSEMBLUCSC | 34 | 6 | 5197 | tss | 0.562582 | 0.050001 | GRID1 (margin: GRID1) | 4.57 | 2.16e-03 | 1.45e-02 |
|  | 19 | 58446312 | 58446988 | PDFSTATSENSEMBLUCSC | 677 | 12 | 22487 | island | 0.929211 | 0.368589 | ZNF418 (margin: ZNF418; ZNF256) | 4.56 | 2.96e-06 | 2.02e-04 |
|  | 10 | 103603722 | 103604019 | PDFSTATSENSEMBLUCSC | 298 | 5 | 5312 | tss | 0.929895 | 0.402403 | KCNIP2; C10orf76 (margin: KCNIP2; C10orf76) | 4.56 | 7.94e-03 | 3.19e-02 |
|  | 7 | 4174939 | 4175403 | PDFSTATSENSEMBLUCSC | 465 | 5 | 36830 | gene | 0.407087 | 0.861989 | SDK1 (margin: SDK1) | 4.56 | 7.94e-03 | 3.19e-02 |
|  | 6 | 29454557 | 29455532 | PDFSTATSENSEMBLUCSC | 976 | 15 | 33659 | gene | 0.106273 | 0.745605 | MAS1L; RPS17P1 (margin: XXbac-BPG13B8.10; MAS1LP1; MAS1L; RPS17P1) | 4.55 | 5.62e-05 | 1.53e-03 |
|  | 14 | 65005951 | 65006359 | PDFSTATSENSEMBLUCSC | 409 | 10 | 12458 | tss;island | 0.015115 | 0.312320 | HSPA2; RP11-973N13.4 (margin: ZBTB1; HSPA2; PPP1R36; RP11-973N13.4; RP11-973N13.5) | 4.55 | 1.15e-02 | 4.29e-02 |
|  | 3 | 192444985 | 192445380 | PDFSTATSENSEMBLUCSC | 396 | 5 | 28087 | gene | 0.442110 | 0.034672 | FGF12; RNU1-20P (margin: FGF12; RNU1-20P) | 4.55 | 7.94e-03 | 3.19e-02 |
|  | 1 | 55007670 | 55008516 | PDFSTATSENSEMBLUCSC | 847 | 11 | 3299 | island | 0.013112 | 0.105780 | ACOT11 (margin: HNRNPA1P63; ACOT11) | 4.54 | 2.84e-06 | 1.96e-04 |
|  | 3 | 123813191 | 123813504 | PDFSTATSENSEMBLUCSC | 314 | 8 | 28548 | tss | 0.391695 | 0.923100 | KALRN (margin: KALRN) | 4.54 | 4.66e-03 | 2.68e-02 |
|  | 6 | 117197952 | 117198743 | PDFSTATSENSEMBLUCSC | 792 | 12 | 36434 | island | 0.496516 | 0.042537 | RFX6 (margin: RFX6) | 4.54 | 7.17e-05 | 1.92e-03 |
|  | 18 | 32556626 | 32557027 | PDFSTATSENSEMBLUCSC | 402 | 7 | 19231 | tss;island | 0.147140 | 0.009193 | MAPRE2 (margin: MAPRE2) | 4.53 | 1.17e-03 | 1.13e-02 |
|  | 7 | 107302255 | 107302510 | PDFSTATSENSEMBLUCSC | 256 | 7 | 37183 | gene;tss;island | 0.434857 | 0.025667 | SLC26A4; SLC26A4-AS1 (margin: SLC26A4; SLC26A4-AS1) | 4.53 | 1.17e-03 | 1.13e-02 |
|  | 19 | 53662353 | 53662723 | PDFSTATSENSEMBLUCSC | 371 | 8 | 21024 | tss | 0.857039 | 0.261367 | ZNF347; ZNF665 (margin: CTD-2245F17.2; ZNF347; ZNF665) | 4.53 | 3.11e-04 | 4.77e-03 |
|  | 6 | 36237982 | 36238410 | PDFSTATSENSEMBLUCSC | 429 | 8 | 34313 | gene | 0.101206 | 0.588040 | PNPLA1 (margin: PNPLA1) | 4.53 | 1.04e-02 | 3.96e-02 |
|  | 17 | 43662488 | 43662979 | PDFSTATSENSEMBLUCSC | 492 | 6 | 18441 | island | 0.033586 | 0.411225 | DND1P1 (margin: DND1P1) | 4.53 | 2.16e-03 | 1.45e-02 |
|  | 11 | 44337982 | 44338421 | PDFSTATSENSEMBLUCSC | 440 | 5 | 8226 | island | 0.431226 | 0.935887 | (margin: ALX4) | 4.53 | 7.94e-03 | 3.19e-02 |
|  | 1 | 3331987 | 3332264 | PDFSTATSENSEMBLUCSC | 278 | 6 | 125 | gene;island | 0.285106 | 0.896736 | PRDM16 (margin: PRDM16) | 4.53 | 8.66e-03 | 3.36e-02 |
|  | 4 | 66536186 | 66536772 | PDFSTATSENSEMBLUCSC | 587 | 7 | 30417 | tss;island | 0.780437 | 0.240590 | RP11-807H7.1; EPHA5 (margin: RP11-807H7.1; EPHA5) | 4.52 | 2.33e-03 | 1.52e-02 |
|  | 15 | 73661340 | 73662002 | PDFSTATSENSEMBLUCSC | 663 | 8 | 14288 | island | 0.413589 | 0.940603 | HCN4 (margin: HCN4) | 4.52 | 6.99e-03 | 3.19e-02 |
|  | 3 | 112931126 | 112931354 | PDFSTATSENSEMBLUCSC | 229 | 6 | 28505 | tss | 0.410678 | 0.032573 | BOC (margin: BOC) | 4.51 | 8.66e-03 | 3.36e-02 |
|  | 2 | 223166909 | 223167617 | PDFSTATSENSEMBLUCSC | 709 | 8 | 23050 | gene;island | 0.421325 | 0.951898 | CCDC140 (margin: CCDC140; PAX3) | 4.51 | 1.55e-04 | 2.71e-03 |
|  | 2 | 133428422 | 133429299 | PDFSTATSENSEMBLUCSC | 878 | 10 | 24746 | island | 0.010720 | 0.108930 | LYPD1; NCKAP5 (margin: AC010974.3; LYPD1; NCKAP5) | 4.51 | 8.93e-03 | 3.45e-02 |
|  | 3 | 134369339 | 134370241 | PDFSTATSENSEMBLUCSC | 903 | 13 | 29500 | island | 0.415460 | 0.899791 | EPHB1; KY (margin: EPHB1; KY) | 4.50 | 8.65e-06 | 4.82e-04 |
|  | 11 | 94134646 | 94135029 | PDFSTATSENSEMBLUCSC | 384 | 8 | 7629 | tss | 0.684159 | 0.984266 | GPR83 (margin: GPR83) | 4.50 | 4.66e-03 | 2.68e-02 |
|  | 19 | 37341052 | 37341261 | PDFSTATSENSEMBLUCSC | 210 | 7 | 20731 | tss | 0.436574 | 0.020782 | ZNF345; ZNF790 (margin: ZNF345; ZNF790; Y\_RNA) | 4.50 | 2.33e-03 | 1.52e-02 |
|  | 14 | 61789466 | 61789733 | PDFSTATSENSEMBLUCSC | 268 | 5 | 12107 | gene;island | 0.011036 | 0.200907 | PRKCH; RP11-902B17.1 (margin: PRKCH; RP11-902B17.1) | 4.50 | 7.94e-03 | 3.19e-02 |
|  | 1 | 1713944 | 1714271 | PDFSTATSENSEMBLUCSC | 328 | 5 | 2462 | island | 0.638812 | 0.985566 | (margin: NADK; GNB1) | 4.50 | 7.94e-03 | 3.19e-02 |
|  | 2 | 242794854 | 242795082 | PDFSTATSENSEMBLUCSC | 229 | 5 | 23148 | gene;island | 0.691629 | 0.980015 | PDCD1 (margin: AC114730.2; PDCD1) | 4.48 | 7.94e-03 | 3.19e-02 |
|  | 3 | 183959000 | 183959171 | PDFSTATSENSEMBLUCSC | 172 | 7 | 28057 | gene;tss;island | 0.516725 | 0.925824 | EIF2B5; VWA5B2; MIR1224; ALG3 (margin: EIF2B5; VWA5B2; MIR1224; ECE2; ALG3) | 4.48 | 5.83e-04 | 6.47e-03 |
|  | 2 | 37571677 | 37571732 | PDFSTATSENSEMBLUCSC | 56 | 5 | 23306 | tss | 0.978153 | 0.662337 | QPCT (margin: QPCT) | 4.48 | 7.94e-03 | 3.19e-02 |
|  | 4 | 77172617 | 77173197 | PDFSTATSENSEMBLUCSC | 581 | 10 | 31115 | island | 0.112784 | 0.005193 | FAM47E; FAM47E-STBD1 (margin: FAM47E; FAM47E-STBD1) | 4.48 | 8.93e-03 | 3.45e-02 |
|  | 2 | 119600348 | 119600627 | PDFSTATSENSEMBLUCSC | 280 | 5 | 22840 | gene;island | 0.621834 | 0.973412 | EN1 (margin: RP11-19E11.1; EN1) | 4.48 | 7.94e-03 | 3.19e-02 |
|  | 3 | 100120235 | 100120961 | PDFSTATSENSEMBLUCSC | 727 | 11 | 27878 | gene;tss;island | 0.111505 | 0.010183 | LNP1; TOMM70A (margin: LNP1; TOMM70A) | 4.48 | 1.86e-03 | 1.45e-02 |
|  | 7 | 29603121 | 29603407 | PDFSTATSENSEMBLUCSC | 287 | 7 | 37512 | tss | 0.116620 | 0.010820 | PRR15; AC007255.8 (margin: RP11-550A18.1; PRR15; AC007255.8) | 4.48 | 6.99e-03 | 3.19e-02 |
|  | 6 | 33160067 | 33160604 | PDFSTATSENSEMBLUCSC | 538 | 15 | 35969 | island | 0.471990 | 0.934988 | COL11A2; RXRB (margin: RNY4P10; SLC39A7; HSD17B8; COL11A2; RXRB) | 4.47 | 4.40e-05 | 1.23e-03 |
|  | 3 | 145879430 | 145879710 | PDFSTATSENSEMBLUCSC | 281 | 6 | 28647 | tss | 0.048617 | 0.541253 | PLOD2 (margin: PLOD2) | 4.47 | 2.16e-03 | 1.45e-02 |
|  | 3 | 155394109 | 155394428 | PDFSTATSENSEMBLUCSC | 320 | 5 | 28004 | gene;tss | 0.530451 | 0.966149 | PLCH1 (margin: PLCH1) | 4.47 | 7.94e-03 | 3.19e-02 |
|  | 19 | 18496756 | 18497201 | PDFSTATSENSEMBLUCSC | 446 | 8 | 21778 | island | 0.017220 | 0.190306 | GDF15; MIR3189 (margin: GDF15; MIR3189; LRRC25) | 4.47 | 1.86e-03 | 1.45e-02 |
|  | 17 | 71640181 | 71640369 | PDFSTATSENSEMBLUCSC | 189 | 7 | 18706 | island | 0.408269 | 0.024002 | RP11-277J6.2; SDK2 (margin: RP11-277J6.2; SDK2) | 4.46 | 4.08e-03 | 2.47e-02 |
|  | 7 | 27213893 | 27214383 | PDFSTATSENSEMBLUCSC | 491 | 10 | 36939 | gene;island | 0.870379 | 0.987948 | RP1-170O19.20; HOXA10 (margin: HOXA-AS4; HOXA11-AS; HOXA9; RP1-170O19.20; MIR196B; HOXA10; HOXA11; RP1-170O19.14) | 4.46 | 4.33e-05 | 1.22e-03 |
|  | 8 | 39695788 | 39696062 | PDFSTATSENSEMBLUCSC | 275 | 6 | 39665 | tss | 0.646368 | 0.964748 | ADAM2 (margin: ADAM2) | 4.46 | 2.16e-03 | 1.45e-02 |
|  | 7 | 108095719 | 108096041 | PDFSTATSENSEMBLUCSC | 323 | 5 | 37185 | gene;island | 0.275370 | 0.035546 | NRCAM (margin: NRCAM) | 4.45 | 7.94e-03 | 3.19e-02 |
|  | 16 | 8806359 | 8806756 | PDFSTATSENSEMBLUCSC | 398 | 8 | 14652 | gene;tss | 0.358623 | 0.981840 | ABAT (margin: ABAT; RNU7-63P) | 4.45 | 1.09e-03 | 1.10e-02 |
|  | 1 | 156338639 | 156339286 | PDFSTATSENSEMBLUCSC | 648 | 8 | 3853 | island | 0.690837 | 0.110200 | RHBG; CCT3 (margin: RHBG; CCT3) | 4.44 | 1.55e-04 | 2.71e-03 |
|  | 15 | 45406327 | 45406939 | PDFSTATSENSEMBLUCSC | 613 | 13 | 13645 | tss;island | 0.204804 | 0.848229 | DUOXA2; DUOX2 (margin: DUOXA2; DUOX2; DUOXA1) | 4.44 | 2.31e-06 | 1.96e-04 |
|  | 12 | 114843188 | 114843955 | PDFSTATSENSEMBLUCSC | 768 | 10 | 9326 | gene;island | 0.365114 | 0.850980 | TBX5 (margin: TBX5-AS1; TBX5) | 4.44 | 4.87e-04 | 6.47e-03 |
|  | 2 | 71205052 | 71205564 | PDFSTATSENSEMBLUCSC | 513 | 6 | 23424 | tss | 0.228566 | 0.886898 | ANKRD53; AC007040.11 (margin: ANKRD53; AC007040.11; TEX261) | 4.44 | 4.33e-03 | 2.52e-02 |
|  | 19 | 12624466 | 12624832 | PDFSTATSENSEMBLUCSC | 367 | 8 | 21604 | island | 0.579203 | 0.981001 | ZNF709 (margin: CTD-3105H18.8; CTD-3105H18.10; CTD-3105H18.9; CTD-3105H18.11; PPIAP20; ZNF709; ZNF564; CTD-2192J16.20) | 4.44 | 4.66e-03 | 2.68e-02 |
|  | 15 | 65203834 | 65204034 | PDFSTATSENSEMBLUCSC | 201 | 5 | 13735 | tss | 0.387776 | 0.020814 | AC069368.3; ANKDD1A (margin: AC069368.3; ANKDD1A; RN7SL348P) | 4.43 | 7.94e-03 | 3.19e-02 |
|  | 8 | 70745466 | 70745926 | PDFSTATSENSEMBLUCSC | 461 | 6 | 39754 | tss | 0.723531 | 0.149375 | RP11-159H10.3; SLCO5A1 (margin: RP11-159H10.3; SLCO5A1) | 4.43 | 8.66e-03 | 3.36e-02 |
|  | 17 | 6679254 | 6679781 | PDFSTATSENSEMBLUCSC | 528 | 12 | 17910 | island | 0.614425 | 0.976909 | XAF1; FBXO39 (margin: XAF1; FBXO39) | 4.43 | 2.22e-05 | 9.05e-04 |
|  | 10 | 115938729 | 115939284 | PDFSTATSENSEMBLUCSC | 556 | 8 | 6151 | island | 0.959261 | 0.583104 | TDRD1 (margin: TDRD1; C10orf118; MIR2110) | 4.43 | 6.22e-04 | 6.71e-03 |
|  | 6 | 117197952 | 117198281 | PDFSTATSENSEMBLUCSC | 330 | 8 | 35194 | tss | 0.394681 | 0.064825 | RFX6 (margin: RFX6) | 4.43 | 1.04e-02 | 3.96e-02 |
|  | 11 | 14913124 | 14914012 | PDFSTATSENSEMBLUCSC | 889 | 13 | 8080 | island | 0.192657 | 0.007621 | CYP2R1 (margin: CYP2R1) | 4.43 | 2.31e-06 | 1.96e-04 |
|  | 3 | 50275394 | 50275694 | PDFSTATSENSEMBLUCSC | 301 | 5 | 27796 | gene;island | 0.008213 | 0.198314 | GNAI2; U73169.1 (margin: GNAI2; U73169.1) | 4.43 | 7.94e-03 | 3.19e-02 |
|  | 7 | 149569715 | 149570367 | PDFSTATSENSEMBLUCSC | 653 | 13 | 37268 | gene;island | 0.508493 | 0.040794 | ATP6V0E2; ATP6V0E2-AS1 (margin: ZNF862; ATP6V0E2; ATP6V0E2-AS1) | 4.43 | 7.95e-04 | 8.28e-03 |
|  | 2 | 50201382 | 50201511 | PDFSTATSENSEMBLUCSC | 130 | 5 | 22675 | gene | 0.610327 | 0.049076 | NRXN1 (margin: NRXN1) | 4.42 | 7.94e-03 | 3.19e-02 |
|  | 5 | 8457548 | 8458089 | PDFSTATSENSEMBLUCSC | 542 | 6 | 32684 | island | 0.043443 | 0.348590 | MIR4458; RP11-480D4.1; RP11-480D4.2; RP11-480D4.4 (margin: MIR4458; RP11-480D4.1; RP11-480D4.2; RP11-480D4.6; RP11-480D4.4) | 4.42 | 2.16e-03 | 1.45e-02 |
|  | 14 | 24808987 | 24809759 | PDFSTATSENSEMBLUCSC | 773 | 8 | 12747 | island | 0.557549 | 0.957387 | RIPK3 (margin: ADCY4; RP11-934B9.3; RIPK3; RNU6-802P) | 4.42 | 1.04e-02 | 3.96e-02 |
|  | 10 | 7453329 | 7453594 | PDFSTATSENSEMBLUCSC | 266 | 7 | 5533 | island | 0.527821 | 0.047732 | SFMBT2 (margin: SFMBT2) | 4.42 | 1.11e-02 | 4.14e-02 |
|  | 10 | 95326033 | 95326409 | PDFSTATSENSEMBLUCSC | 377 | 9 | 5238 | tss;island | 0.473957 | 0.964960 | FFAR4 (margin: FFAR4) | 4.42 | 4.11e-05 | 1.17e-03 |
|  | 3 | 194406190 | 194406572 | PDFSTATSENSEMBLUCSC | 383 | 9 | 28802 | tss;island | 0.422638 | 0.054896 | FAM43A (margin: FAM43A) | 4.41 | 1.06e-02 | 4.01e-02 |
|  | 3 | 180319597 | 180320159 | PDFSTATSENSEMBLUCSC | 563 | 9 | 29684 | island | 0.025245 | 0.331409 | TTC14; RP11-496B10.3; CCDC39 (margin: TTC14; RP11-496B10.3; RP11-496B10.1; CCDC39) | 4.41 | 4.94e-04 | 6.47e-03 |
|  | 20 | 50158996 | 50159653 | PDFSTATSENSEMBLUCSC | 658 | 6 | 25454 | gene;island | 0.817214 | 0.192707 | NFATC2 (margin: NFATC2) | 4.41 | 4.33e-03 | 2.52e-02 |
|  | 3 | 32859377 | 32859445 | PDFSTATSENSEMBLUCSC | 69 | 5 | 28208 | tss | 0.752612 | 0.984359 | TRIM71 (margin: TRIM71) | 4.40 | 7.94e-03 | 3.19e-02 |
|  | 6 | 112575705 | 112576119 | PDFSTATSENSEMBLUCSC | 415 | 6 | 36423 | island | 0.482311 | 0.958969 | RP11-506B6.6; LAMA4 (margin: RP11-506B6.6; LAMA4) | 4.40 | 2.16e-03 | 1.45e-02 |
|  | 1 | 201476290 | 201476960 | PDFSTATSENSEMBLUCSC | 671 | 11 | 4077 | island | 0.019877 | 0.433694 | RP11-134G8.7; CSRP1 (margin: RP11-134G8.7; RPS10P7; CSRP1) | 4.40 | 1.04e-02 | 3.96e-02 |
|  | 19 | 10397429 | 10397642 | PDFSTATSENSEMBLUCSC | 214 | 5 | 20443 | tss | 0.314858 | 0.931560 | ICAM1; ICAM4; CTD-2369P2.5; CTD-2369P2.8 (margin: ICAM1; ICAM4; ICAM5; CTD-2369P2.5; CTD-2369P2.8) | 4.40 | 7.94e-03 | 3.19e-02 |
|  | 10 | 31609882 | 31609960 | PDFSTATSENSEMBLUCSC | 79 | 6 | 5018 | tss | 0.082845 | 0.658260 | ZEB1; ZEB1-AS1 (margin: ZEB1; ZEB1-AS1) | 4.40 | 2.16e-03 | 1.45e-02 |
|  | 17 | 75446431 | 75446661 | PDFSTATSENSEMBLUCSC | 231 | 6 | 16808 | gene;island | 0.156153 | 0.801430 | SEPT9 (margin: SEPT9; Y\_RNA) | 4.40 | 2.16e-03 | 1.45e-02 |
|  | 7 | 156796967 | 156797410 | PDFSTATSENSEMBLUCSC | 444 | 6 | 39091 | island | 0.755968 | 0.992195 | RP5-1121A15.4; MNX1 (margin: RP5-1121A15.4; RP5-1121A15.1; MNX1) | 4.39 | 8.66e-03 | 3.36e-02 |
|  | 11 | 134145889 | 134146266 | PDFSTATSENSEMBLUCSC | 378 | 8 | 7832 | tss | 0.501605 | 0.974617 | GLB1L3 (margin: ACAD8; GLB1L3) | 4.39 | 1.09e-03 | 1.10e-02 |
|  | 16 | 54965084 | 54965142 | PDFSTATSENSEMBLUCSC | 59 | 5 | 15931 | island | 0.141191 | 0.007540 | IRX5; CRNDE (margin: IRX5; CTD-3032H12.2; CRNDE; CTD-3032H12.1) | 4.39 | 7.94e-03 | 3.19e-02 |
|  | 20 | 35972924 | 35973098 | PDFSTATSENSEMBLUCSC | 175 | 5 | 25726 | tss;island | 0.154581 | 0.756332 | SRC (margin: SRC) | 4.39 | 7.94e-03 | 3.19e-02 |
|  | 10 | 88126089 | 88126306 | PDFSTATSENSEMBLUCSC | 218 | 8 | 5902 | island | 0.562582 | 0.054792 | GRID1 (margin: GRID1) | 4.39 | 1.55e-04 | 2.71e-03 |
|  | 17 | 75446431 | 75446592 | PDFSTATSENSEMBLUCSC | 162 | 5 | 17698 | tss | 0.208314 | 0.846268 | SEPT9 (margin: SEPT9; Y\_RNA) | 4.39 | 7.94e-03 | 3.19e-02 |
|  | 17 | 40713676 | 40714003 | PDFSTATSENSEMBLUCSC | 328 | 8 | 17365 | tss | 0.031568 | 0.397819 | RP11-400F19.8; COASY (margin: RP11-400F19.8; HSD17B1; COASY; MLX; RP11-400F19.6; PSMC3IP) | 4.39 | 6.99e-03 | 3.19e-02 |
|  | 3 | 173115252 | 173116248 | PDFSTATSENSEMBLUCSC | 997 | 13 | 29671 | island | 0.545143 | 0.948845 | NLGN1 (margin: NLGN1) | 4.38 | 8.65e-06 | 4.82e-04 |
|  | 2 | 264120 | 264204 | PDFSTATSENSEMBLUCSC | 85 | 6 | 23152 | tss;island | 0.109763 | 0.004911 | ACP1; SH3YL1 (margin: ACP1; SH3YL1) | 4.38 | 4.33e-03 | 2.52e-02 |
|  | 5 | 140562562 | 140563400 | PDFSTATSENSEMBLUCSC | 839 | 8 | 31813 | gene;island | 0.662667 | 0.977164 | PCDHB16 (margin: PCDHB7; PCDHB8; PCDHB16; PCDHB9; PCDHB10) | 4.37 | 3.11e-04 | 4.77e-03 |
|  | 3 | 142839837 | 142840240 | PDFSTATSENSEMBLUCSC | 404 | 5 | 27980 | gene;island | 0.769801 | 0.974507 | CHST2; RP11-80H8.4 (margin: RP11-80H8.3; CHST2; RP11-80H8.4) | 4.37 | 7.94e-03 | 3.19e-02 |
|  | 10 | 26726877 | 26727261 | PDFSTATSENSEMBLUCSC | 385 | 7 | 4994 | tss | 0.489592 | 0.902020 | APBB1IP (margin: APBB1IP) | 4.37 | 5.83e-04 | 6.47e-03 |
|  | 11 | 114069864 | 114070210 | PDFSTATSENSEMBLUCSC | 347 | 6 | 6911 | gene;island | 0.589264 | 0.972092 | ZBTB16 (margin: ZBTB16; RP11-64D24.2) | 4.37 | 2.16e-03 | 1.45e-02 |
|  | 17 | 1082526 | 1083491 | PDFSTATSENSEMBLUCSC | 966 | 9 | 16310 | gene;island | 0.085581 | 0.009407 | AC016292.1; ABR (margin: AC016292.1; ABR) | 4.36 | 5.64e-03 | 3.18e-02 |
|  | 7 | 100493365 | 100493849 | PDFSTATSENSEMBLUCSC | 485 | 7 | 38785 | island | 0.216535 | 0.008756 | ACHE (margin: SRRT; UFSP1; ACHE; RN7SL549P) | 4.36 | 4.08e-03 | 2.47e-02 |
|  | 8 | 144635260 | 144635547 | PDFSTATSENSEMBLUCSC | 288 | 9 | 39950 | tss | 0.532168 | 0.970844 | GSDMD (margin: 7SK; GSDMD; ZC3H3; RP11-661A12.5) | 4.36 | 4.11e-05 | 1.17e-03 |
|  | 4 | 73434487 | 73434856 | PDFSTATSENSEMBLUCSC | 370 | 5 | 31092 | island | 0.027031 | 0.591170 | ADAMTS3 (margin: ADAMTS3) | 4.36 | 7.94e-03 | 3.19e-02 |
|  | 11 | 14913124 | 14913620 | PDFSTATSENSEMBLUCSC | 497 | 5 | 6519 | gene | 0.233310 | 0.014899 | CYP2R1 (margin: CYP2R1) | 4.35 | 7.94e-03 | 3.19e-02 |
|  | 11 | 30607950 | 30608198 | PDFSTATSENSEMBLUCSC | 249 | 9 | 7220 | tss | 0.343962 | 0.021375 | RP5-1024C24.1; MPPED2 (margin: RP5-1024C24.1; MPPED2) | 4.35 | 4.11e-05 | 1.17e-03 |
|  | 6 | 168615184 | 168615500 | PDFSTATSENSEMBLUCSC | 317 | 6 | 36661 | island | 0.437395 | 0.957606 | (margin: RP11-503C24.1; RP11-503C24.2) | 4.35 | 8.66e-03 | 3.36e-02 |
|  | 5 | 80529067 | 80529340 | PDFSTATSENSEMBLUCSC | 274 | 6 | 31666 | gene | 0.974666 | 0.663692 | CKMT2; CTC-281B15.1 (margin: RASGRF2; CKMT2; CTC-281B15.1; CTD-2248H3.1) | 4.34 | 2.16e-03 | 1.45e-02 |
|  | 12 | 6420020 | 6420052 | PDFSTATSENSEMBLUCSC | 33 | 6 | 9023 | gene;tss;island | 0.938658 | 0.435392 | PLEKHG6 (margin: PLEKHG6) | 4.34 | 2.16e-03 | 1.45e-02 |
|  | 5 | 170736021 | 170736572 | PDFSTATSENSEMBLUCSC | 552 | 10 | 33379 | island | 0.760427 | 0.981002 | TLX3 (margin: RANBP17; TLX3) | 4.34 | 1.08e-05 | 4.82e-04 |
|  | 12 | 15475116 | 15475342 | PDFSTATSENSEMBLUCSC | 227 | 6 | 9591 | tss | 0.200676 | 0.804411 | PTPRO; RERG (margin: PTPRO; RERG) | 4.34 | 2.16e-03 | 1.45e-02 |
|  | 11 | 2481206 | 2481641 | PDFSTATSENSEMBLUCSC | 436 | 7 | 6459 | gene;tss | 0.407075 | 0.967831 | KCNQ1 (margin: KCNQ1) | 4.34 | 6.99e-03 | 3.19e-02 |
|  | 10 | 102986525 | 102986973 | PDFSTATSENSEMBLUCSC | 449 | 6 | 6059 | island | 0.614839 | 0.977380 | LBX1 (margin: LBX1-AS1; LBX1) | 4.33 | 8.66e-03 | 3.36e-02 |
|  | 7 | 55516724 | 55517132 | PDFSTATSENSEMBLUCSC | 409 | 5 | 38505 | island | 0.702857 | 0.968149 | VOPP1 (margin: VOPP1) | 4.33 | 7.94e-03 | 3.19e-02 |
|  | 13 | 98829311 | 98829666 | PDFSTATSENSEMBLUCSC | 356 | 8 | 11197 | gene;island | 0.669895 | 0.978884 | FARP1; RNF113B (margin: FARP1; RNF113B) | 4.33 | 6.22e-04 | 6.71e-03 |
|  | 5 | 115697214 | 115697696 | PDFSTATSENSEMBLUCSC | 483 | 5 | 33031 | island | 0.645404 | 0.973916 | COMMD10 (margin: COMMD10) | 4.33 | 7.94e-03 | 3.19e-02 |
|  | 6 | 21666391 | 21666939 | PDFSTATSENSEMBLUCSC | 549 | 8 | 35486 | island | 0.477496 | 0.921616 | LINC00340 (margin: LINC00340) | 4.33 | 6.22e-04 | 6.71e-03 |
|  | 7 | 130125200 | 130126024 | PDFSTATSENSEMBLUCSC | 825 | 12 | 37900 | tss | 0.815743 | 0.102061 | MEST; RP11-2E11.5 (margin: MEST; MIR335; RP11-2E11.5; hsa-mir-335) | 4.32 | 2.32e-03 | 1.52e-02 |
|  | 1 | 6085799 | 6086652 | PDFSTATSENSEMBLUCSC | 854 | 10 | 2589 | island | 0.049736 | 0.547228 | KCNAB2 (margin: KCNAB2) | 4.32 | 7.58e-05 | 1.98e-03 |
|  | 15 | 83240550 | 83240791 | PDFSTATSENSEMBLUCSC | 242 | 7 | 13453 | gene | 0.487301 | 0.844147 | CPEB1 (margin: RP11-379H8.1; CPEB1) | 4.32 | 5.83e-04 | 6.47e-03 |
|  | 5 | 65892993 | 65893538 | PDFSTATSENSEMBLUCSC | 546 | 5 | 31635 | gene;island | 0.088883 | 0.010228 | MAST4 (margin: MAST4) | 4.31 | 7.94e-03 | 3.19e-02 |
|  | 14 | 104940229 | 104940658 | PDFSTATSENSEMBLUCSC | 430 | 5 | 13206 | island | 0.505771 | 0.971277 | TMEM179 (margin: TMEM179) | 4.31 | 7.94e-03 | 3.19e-02 |
|  | 1 | 59247425 | 59247797 | PDFSTATSENSEMBLUCSC | 373 | 5 | 558 | gene;island | 0.004355 | 0.041325 | JUN (margin: RP4-794H19.2; JUN) | 4.31 | 7.94e-03 | 3.19e-02 |
|  | 5 | 1445354 | 1445593 | PDFSTATSENSEMBLUCSC | 240 | 6 | 32629 | island | 0.442529 | 0.966869 | SLC6A3 (margin: SLC6A3; LPCAT1) | 4.31 | 2.16e-03 | 1.45e-02 |
|  | 19 | 12978611 | 12978805 | PDFSTATSENSEMBLUCSC | 195 | 5 | 19871 | gene;island | 0.687689 | 0.976858 | MAST1; HOOK2 (margin: MAST1; HOOK2; AC020934.1; DNASE2) | 4.31 | 7.94e-03 | 3.19e-02 |
|  | 6 | 30881112 | 30881842 | PDFSTATSENSEMBLUCSC | 731 | 23 | 33835 | gene | 0.916063 | 0.106615 | GTF2H4; VARS2 (margin: RN7SL175P; GTF2H4; VARS2) | 4.31 | 9.28e-06 | 4.82e-04 |
|  | 13 | 108922032 | 108922504 | PDFSTATSENSEMBLUCSC | 473 | 7 | 11224 | gene | 0.096754 | 0.656477 | TNFSF13B (margin: TNFSF13B) | 4.31 | 2.33e-03 | 1.52e-02 |
|  | 3 | 173115252 | 173116238 | PDFSTATSENSEMBLUCSC | 987 | 12 | 28723 | tss | 0.547077 | 0.952922 | NLGN1 (margin: NLGN1) | 4.30 | 3.33e-05 | 1.17e-03 |
|  | 5 | 170736021 | 170736277 | PDFSTATSENSEMBLUCSC | 257 | 7 | 32486 | tss | 0.775874 | 0.975876 | TLX3 (margin: RANBP17; TLX3) | 4.30 | 5.83e-04 | 6.47e-03 |
|  | 2 | 71205052 | 71205685 | PDFSTATSENSEMBLUCSC | 634 | 7 | 24391 | island | 0.389157 | 0.920247 | ANKRD53; AC007040.11 (margin: ANKRD53; AC007040.11; TEX261) | 4.30 | 2.33e-03 | 1.52e-02 |
|  | 1 | 102462445 | 102462682 | PDFSTATSENSEMBLUCSC | 238 | 5 | 656 | gene | 0.502977 | 0.049070 | OLFM3 (margin: OLFM3) | 4.29 | 7.94e-03 | 3.19e-02 |
|  | 12 | 26348331 | 26348529 | PDFSTATSENSEMBLUCSC | 199 | 6 | 9086 | gene;island | 0.282266 | 0.021869 | SSPN (margin: SSPN) | 4.29 | 4.33e-03 | 2.52e-02 |
|  | 10 | 28034669 | 28034856 | PDFSTATSENSEMBLUCSC | 188 | 7 | 5634 | island | 0.739197 | 0.977454 | RP11-360I20.2; MKX (margin: RP11-360I20.2; MKX) | 4.29 | 5.83e-04 | 6.47e-03 |
|  | 4 | 186732837 | 186733060 | PDFSTATSENSEMBLUCSC | 224 | 7 | 30212 | gene;tss | 0.755083 | 0.983242 | SORBS2 (margin: SORBS2) | 4.29 | 5.83e-04 | 6.47e-03 |
|  | 12 | 114846313 | 114846503 | PDFSTATSENSEMBLUCSC | 191 | 7 | 10033 | tss | 0.638526 | 0.969805 | TBX5-AS1; TBX5 (margin: TBX5-AS1; TBX5) | 4.29 | 5.83e-04 | 6.47e-03 |
|  | 14 | 101347044 | 101347358 | PDFSTATSENSEMBLUCSC | 315 | 6 | 12210 | gene;tss;island | 0.161891 | 0.860214 | MIR431; MIR433; MIR127; RTL1 (margin: MIR493; MIR337; MIR665; MIR431; MIR433; MIR127; MIR432; MIR136; AL117190.3; RTL1) | 4.29 | 2.16e-03 | 1.45e-02 |
|  | 4 | 93224895 | 93225565 | PDFSTATSENSEMBLUCSC | 671 | 8 | 31183 | island | 0.493817 | 0.047642 | GRID2; RP11-9B6.1 (margin: GRID2; RP11-9B6.1) | 4.28 | 1.86e-03 | 1.45e-02 |
|  | 3 | 112931126 | 112931496 | PDFSTATSENSEMBLUCSC | 371 | 8 | 29363 | island | 0.410678 | 0.042093 | BOC (margin: BOC) | 4.28 | 1.86e-03 | 1.45e-02 |
|  | 11 | 69590282 | 69590580 | PDFSTATSENSEMBLUCSC | 299 | 7 | 7522 | tss | 0.517153 | 0.954422 | FGF4 (margin: AP001888.1; FGF4) | 4.28 | 5.83e-04 | 6.47e-03 |
|  | 10 | 23216637 | 23216847 | PDFSTATSENSEMBLUCSC | 211 | 6 | 4986 | tss | 0.647070 | 0.106642 | ARMC3 (margin: ARMC3) | 4.28 | 2.16e-03 | 1.45e-02 |
|  | 5 | 135416331 | 135416613 | PDFSTATSENSEMBLUCSC | 283 | 9 | 32310 | tss | 0.536402 | 0.954643 | VTRNA2-1 (margin: VTRNA2-1) | 4.28 | 8.23e-05 | 2.06e-03 |
|  | 10 | 133508246 | 133508866 | PDFSTATSENSEMBLUCSC | 621 | 7 | 6278 | island | 0.297049 | 0.944735 | (margin: ) | 4.27 | 5.83e-04 | 6.47e-03 |
|  | 4 | 142054660 | 142054878 | PDFSTATSENSEMBLUCSC | 219 | 8 | 30609 | tss;island | 0.137613 | 0.005399 | RNF150 (margin: RNF150) | 4.27 | 4.66e-03 | 2.68e-02 |
|  | 20 | 61151391 | 61151480 | PDFSTATSENSEMBLUCSC | 90 | 6 | 25490 | gene;tss;island | 0.116732 | 0.728127 | C20orf166; MIR1-1 (margin: C20orf166; MIR1-1; MIR133A2; C20orf166-AS1) | 4.27 | 2.16e-03 | 1.45e-02 |
|  | 15 | 37403088 | 37403304 | PDFSTATSENSEMBLUCSC | 217 | 5 | 14015 | island | 0.423686 | 0.932819 | (margin: RP11-128A17.1; MEIS2) | 4.27 | 7.94e-03 | 3.19e-02 |
|  | 22 | 38071455 | 38071607 | PDFSTATSENSEMBLUCSC | 153 | 5 | 27121 | tss | 0.014990 | 0.219301 | LGALS1 (margin: SH3BP1; PDXP; LGALS1; NOL12; RP1-37E16.12; RN7SL385P) | 4.27 | 7.94e-03 | 3.19e-02 |
|  | 5 | 133451237 | 133451625 | PDFSTATSENSEMBLUCSC | 389 | 8 | 31750 | gene;island | 0.191375 | 0.848365 | TCF7 (margin: TCF7) | 4.26 | 1.09e-03 | 1.10e-02 |
|  | 5 | 148520669 | 148521226 | PDFSTATSENSEMBLUCSC | 558 | 11 | 33293 | island | 0.033761 | 0.506360 | ABLIM3 (margin: ABLIM3) | 4.26 | 8.33e-03 | 3.34e-02 |
|  | 4 | 40859249 | 40859344 | PDFSTATSENSEMBLUCSC | 96 | 6 | 30361 | tss | 0.967730 | 0.605984 | APBB2 (margin: APBB2; snoU13) | 4.26 | 2.16e-03 | 1.45e-02 |
|  | 7 | 82073339 | 82073810 | PDFSTATSENSEMBLUCSC | 472 | 7 | 37677 | tss;island | 0.438566 | 0.026865 | CACNA2D1 (margin: CACNA2D1) | 4.26 | 5.83e-04 | 6.47e-03 |
|  | 5 | 1243714 | 1243949 | PDFSTATSENSEMBLUCSC | 236 | 5 | 31528 | gene;island | 0.404799 | 0.946942 | SLC6A18 (margin: SLC6A18; TERT) | 4.26 | 7.94e-03 | 3.19e-02 |
|  | 1 | 43533796 | 43534324 | PDFSTATSENSEMBLUCSC | 529 | 6 | 3157 | island | 0.955390 | 0.534578 | (margin: ) | 4.25 | 2.16e-03 | 1.45e-02 |
|  | 11 | 134201447 | 134201640 | PDFSTATSENSEMBLUCSC | 194 | 6 | 7833 | tss;island | 0.319516 | 0.028015 | GLB1L2 (margin: GLB1L3; GLB1L2) | 4.25 | 4.33e-03 | 2.52e-02 |
|  | 6 | 28601269 | 28601519 | PDFSTATSENSEMBLUCSC | 251 | 14 | 35592 | island | 0.969866 | 0.626514 | RP11-373N24.2 (margin: RP11-373N24.2) | 4.25 | 4.99e-08 | 9.34e-06 |
|  | 19 | 4535211 | 4535582 | PDFSTATSENSEMBLUCSC | 372 | 8 | 20366 | tss | 0.090342 | 0.008659 | PLIN5; CTB-50L17.14; LRG1 (margin: RN7SL121P; PLIN5; CTB-50L17.14; LRG1; SEMA6B) | 4.25 | 1.04e-02 | 3.96e-02 |
|  | 6 | 166891617 | 166891959 | PDFSTATSENSEMBLUCSC | 343 | 5 | 34546 | gene | 0.740611 | 0.978701 | RPS6KA2 (margin: RPS6KA2) | 4.25 | 7.94e-03 | 3.19e-02 |
|  | 5 | 1225074 | 1225434 | PDFSTATSENSEMBLUCSC | 361 | 8 | 31968 | tss | 0.180399 | 0.908712 | SLC6A19; SLC6A18 (margin: SLC6A19; SLC6A18) | 4.24 | 6.99e-03 | 3.19e-02 |
|  | 4 | 144621898 | 144621971 | PDFSTATSENSEMBLUCSC | 74 | 5 | 30615 | tss;island | 0.956013 | 0.489243 | RP13-578N3.3; FREM3 (margin: RP13-578N3.3; FREM3) | 4.23 | 7.94e-03 | 3.19e-02 |
|  | 12 | 85673860 | 85674694 | PDFSTATSENSEMBLUCSC | 835 | 11 | 10658 | island | 0.630367 | 0.973145 | ALX1 (margin: ALX1) | 4.23 | 5.24e-03 | 2.98e-02 |
|  | 4 | 164253207 | 164254230 | PDFSTATSENSEMBLUCSC | 1024 | 11 | 31400 | island | 0.054112 | 0.006540 | NPY1R (margin: NPY5R; NPY1R) | 4.23 | 5.24e-03 | 2.98e-02 |
|  | 3 | 118753610 | 118753871 | PDFSTATSENSEMBLUCSC | 262 | 7 | 27904 | gene;island | 0.154896 | 0.007173 | IGSF11 (margin: IGSF11) | 4.23 | 2.33e-03 | 1.52e-02 |
|  | 8 | 25902284 | 25902611 | PDFSTATSENSEMBLUCSC | 328 | 6 | 40170 | island | 0.511699 | 0.087066 | EBF2 (margin: EBF2) | 4.22 | 2.16e-03 | 1.45e-02 |
|  | 6 | 30043727 | 30044259 | PDFSTATSENSEMBLUCSC | 533 | 10 | 34763 | tss | 0.044666 | 0.531996 | RNF39 (margin: ZNRD1; PPP1R11; RNF39) | 4.22 | 1.50e-03 | 1.41e-02 |
|  | 16 | 54228169 | 54228582 | PDFSTATSENSEMBLUCSC | 414 | 5 | 15929 | island | 0.807668 | 0.215833 | (margin: ) | 4.22 | 7.94e-03 | 3.19e-02 |
|  | 7 | 1280040 | 1280640 | PDFSTATSENSEMBLUCSC | 601 | 5 | 38094 | island | 0.312065 | 0.898665 | (margin: UNCX) | 4.22 | 7.94e-03 | 3.19e-02 |
|  | 1 | 26233332 | 26233709 | PDFSTATSENSEMBLUCSC | 378 | 11 | 1380 | tss;island | 0.503164 | 0.952445 | STMN1; MIR3917 (margin: AL033528.1; STMN1; MIR3917) | 4.21 | 1.28e-04 | 2.71e-03 |
|  | 11 | 66034681 | 66036038 | PDFSTATSENSEMBLUCSC | 1358 | 55 | 7460 | tss | 0.292714 | 0.009635 | KLC2; RAB1B; RP11-867G23.1; RP11-867G23.2; RP11-867G23.3 (margin: KLC2; RAB1B; CNIH2; RP11-755F10.3; RP11-867G23.1; RP11-867G23.2; RP11-867G23.3; RP11-867G23.4) | 4.21 | 7.14e-06 | 4.37e-04 |
|  | 17 | 6679254 | 6679541 | PDFSTATSENSEMBLUCSC | 288 | 9 | 17053 | tss | 0.613975 | 0.976445 | XAF1; FBXO39 (margin: XAF1; FBXO39) | 4.21 | 2.88e-04 | 4.58e-03 |
|  | 1 | 201857840 | 201857955 | PDFSTATSENSEMBLUCSC | 116 | 6 | 942 | gene | 0.437680 | 0.044005 | SHISA4 (margin: IPO9; SHISA4; RP11-307B6.3; LMOD1) | 4.21 | 2.16e-03 | 1.45e-02 |
|  | 15 | 73735098 | 73735476 | PDFSTATSENSEMBLUCSC | 379 | 8 | 13782 | tss | 0.394118 | 0.930991 | C15orf60 (margin: C15orf60) | 4.20 | 6.99e-03 | 3.19e-02 |
|  | 14 | 101528123 | 101528671 | PDFSTATSENSEMBLUCSC | 549 | 11 | 13151 | island | 0.312633 | 0.826818 | MIR154; MIR496; MIR377 (margin: MIR381HG; AL132709.3; MIR487A; MIR134; MIR485; MIR323B; MIR154; MIR496; MIR377; MIR541; MIR409; MIR412; MIR369; MIR410; MIR656; MEG9; MIR382; AL132709.1) | 4.20 | 1.04e-02 | 3.96e-02 |
|  | 19 | 22816896 | 22817496 | PDFSTATSENSEMBLUCSC | 601 | 10 | 21843 | island | 0.484423 | 0.018276 | ZNF492 (margin: ZNF492; AC011516.2) | 4.20 | 3.25e-04 | 4.91e-03 |
|  | 19 | 22816896 | 22817096 | PDFSTATSENSEMBLUCSC | 201 | 6 | 20664 | tss | 0.484423 | 0.018276 | ZNF492 (margin: ZNF492; AC011516.2) | 4.20 | 8.66e-03 | 3.36e-02 |
|  | 21 | 45148694 | 45149141 | PDFSTATSENSEMBLUCSC | 448 | 5 | 26447 | gene;island | 0.499797 | 0.939262 | PDXK; AP001052.9 (margin: PDXK; AP001052.9) | 4.19 | 7.94e-03 | 3.19e-02 |
|  | 11 | 85565852 | 85565989 | PDFSTATSENSEMBLUCSC | 138 | 7 | 7606 | tss;island | 0.006200 | 0.114151 | CCDC83; AP000974.1 (margin: CCDC83; AP000974.1) | 4.19 | 5.83e-04 | 6.47e-03 |
|  | 15 | 73735098 | 73735694 | PDFSTATSENSEMBLUCSC | 597 | 12 | 14289 | island | 0.463388 | 0.957353 | C15orf60 (margin: C15orf60) | 4.19 | 2.74e-04 | 4.46e-03 |
|  | 1 | 45082704 | 45083278 | PDFSTATSENSEMBLUCSC | 575 | 7 | 491 | gene;island | 0.846733 | 0.983987 | RNF220 (margin: RNF220) | 4.19 | 4.08e-03 | 2.47e-02 |
|  | 1 | 26372718 | 26373407 | PDFSTATSENSEMBLUCSC | 690 | 7 | 1381 | tss;island | 0.592055 | 0.974815 | SLC30A2 (margin: EXTL1; SLC30A2; TRIM63) | 4.19 | 6.99e-03 | 3.19e-02 |
|  | 11 | 3663491 | 3663842 | PDFSTATSENSEMBLUCSC | 352 | 9 | 7096 | tss | 0.320098 | 0.023018 | ART5 (margin: TRPC2; ART1; ART5) | 4.19 | 4.11e-05 | 1.17e-03 |
|  | 11 | 3663198 | 3663842 | PDFSTATSENSEMBLUCSC | 645 | 11 | 8000 | island | 0.327204 | 0.023018 | ART5 (margin: TRPC2; ART1; ART5) | 4.19 | 2.75e-04 | 4.46e-03 |
|  | 8 | 144069457 | 144069888 | PDFSTATSENSEMBLUCSC | 432 | 6 | 39470 | gene | 0.820375 | 0.137814 | RP11-273G15.2 (margin: CDC42P3; RP11-273G15.2) | 4.18 | 4.33e-03 | 2.52e-02 |
|  | 4 | 81186795 | 81187198 | PDFSTATSENSEMBLUCSC | 404 | 5 | 30461 | tss;island | 0.641858 | 0.970005 | FGF5 (margin: FGF5) | 4.18 | 7.94e-03 | 3.19e-02 |
|  | 1 | 156211409 | 156211896 | PDFSTATSENSEMBLUCSC | 488 | 10 | 2011 | tss | 0.427327 | 0.891505 | PMF1-BGLAP; PMF1; BGLAP; PAQR6 (margin: PMF1-BGLAP; PMF1; BGLAP; PAQR6; SMG5) | 4.18 | 1.05e-03 | 1.08e-02 |
|  | 14 | 38068553 | 38069206 | PDFSTATSENSEMBLUCSC | 654 | 9 | 12798 | island | 0.918635 | 0.286835 | TTC6; FOXA1 (margin: TTC6; FOXA1) | 4.18 | 4.11e-05 | 1.17e-03 |
|  | 12 | 45270207 | 45270896 | PDFSTATSENSEMBLUCSC | 690 | 18 | 9101 | gene;tss;island | 0.247954 | 0.019714 | NELL2 (margin: NELL2; Y\_RNA) | 4.18 | 8.22e-08 | 1.43e-05 |
|  | 10 | 135020656 | 135021100 | PDFSTATSENSEMBLUCSC | 445 | 5 | 4891 | gene;island | 0.392066 | 0.920561 | KNDC1 (margin: KNDC1) | 4.17 | 7.94e-03 | 3.19e-02 |
|  | 12 | 118313657 | 118313990 | PDFSTATSENSEMBLUCSC | 334 | 5 | 9339 | gene | 0.639577 | 0.980522 | KSR2 (margin: KSR2) | 4.17 | 7.94e-03 | 3.19e-02 |
|  | 5 | 1225074 | 1225719 | PDFSTATSENSEMBLUCSC | 646 | 10 | 32616 | island | 0.253550 | 0.900277 | SLC6A19; SLC6A18 (margin: SLC6A19; SLC6A18) | 4.17 | 4.87e-04 | 6.47e-03 |
|  | 7 | 30029717 | 30029938 | PDFSTATSENSEMBLUCSC | 222 | 7 | 37514 | tss;island | 0.085127 | 0.543670 | AC007285.6; SCRN1 (margin: AC007285.6; SCRN1) | 4.17 | 1.11e-02 | 4.14e-02 |
|  | 5 | 140305947 | 140306458 | PDFSTATSENSEMBLUCSC | 512 | 10 | 31804 | gene;island | 0.957368 | 0.508699 | PCDHA1; PCDHA2; PCDHA3; PCDHA4; PCDHA5; PCDHA6; PCDHA7; PCDHA8; PCDHA9; PCDHA10; PCDHA11; PCDHA12; PCDHA13; PCDHAC1 (margin: PCDHA1; PCDHA2; PCDHA3; PCDHA4; PCDHA5; PCDHA6; PCDHA7; PCDHA8; PCDHA9; PCDHA10; PCDHA11; PCDHA12; PCDHA13; PCDHAC1) | 4.16 | 7.58e-05 | 1.98e-03 |
|  | 11 | 76381687 | 76382149 | PDFSTATSENSEMBLUCSC | 463 | 7 | 7577 | tss;island | 0.584332 | 0.960984 | LRRC32 (margin: AP001189.4; RP11-672A2.3; LRRC32) | 4.16 | 2.33e-03 | 1.52e-02 |
|  | 2 | 14774786 | 14775199 | PDFSTATSENSEMBLUCSC | 414 | 6 | 22579 | gene;island | 0.566965 | 0.036494 | FAM84A; AC011897.1 (margin: AC011897.2; FAM84A; AC011897.1) | 4.16 | 8.66e-03 | 3.36e-02 |
|  | 19 | 19383613 | 19384165 | PDFSTATSENSEMBLUCSC | 553 | 5 | 21810 | island | 0.626232 | 0.969111 | AC138430.4; TM6SF2 (margin: HAPLN4; AC138430.4; TM6SF2; SUGP1) | 4.16 | 7.94e-03 | 3.19e-02 |
|  | 7 | 120968877 | 120969079 | PDFSTATSENSEMBLUCSC | 203 | 7 | 37861 | tss | 0.839002 | 0.198532 | WNT16 (margin: WNT16) | 4.16 | 5.83e-04 | 6.47e-03 |
|  | 7 | 87229552 | 87230344 | PDFSTATSENSEMBLUCSC | 793 | 9 | 37072 | gene;island | 0.376842 | 0.922394 | ABCB1 (margin: ABCB1) | 4.16 | 8.23e-05 | 2.06e-03 |
|  | 12 | 49582842 | 49583112 | PDFSTATSENSEMBLUCSC | 271 | 8 | 10388 | island | 0.128358 | 0.009218 | TUBA1C; TUBA1A (margin: TUBA1C; TUBA1A) | 4.15 | 3.11e-04 | 4.77e-03 |
|  | 1 | 2537547 | 2537838 | PDFSTATSENSEMBLUCSC | 292 | 6 | 92 | gene;island | 0.456664 | 0.944579 | MMEL1 (margin: MMEL1) | 4.14 | 4.33e-03 | 2.52e-02 |
|  | 11 | 2481857 | 2482594 | PDFSTATSENSEMBLUCSC | 738 | 15 | 7076 | tss | 0.392645 | 0.943668 | KCNQ1 (margin: KCNQ1) | 4.14 | 3.51e-06 | 2.34e-04 |
|  | 3 | 179754521 | 179754761 | PDFSTATSENSEMBLUCSC | 241 | 7 | 28734 | tss | 0.932774 | 0.395108 | PEX5L (margin: PEX5L) | 4.13 | 6.99e-03 | 3.19e-02 |
|  | 8 | 10261641 | 10262221 | PDFSTATSENSEMBLUCSC | 581 | 6 | 39195 | gene;island | 0.470947 | 0.933013 | MSRA (margin: MSRA) | 4.13 | 4.33e-03 | 2.52e-02 |
|  | 5 | 41510325 | 41510851 | PDFSTATSENSEMBLUCSC | 527 | 7 | 32754 | island | 0.401197 | 0.036869 | PLCXD3 (margin: PLCXD3) | 4.13 | 5.83e-04 | 6.47e-03 |
|  | 6 | 150463775 | 150464124 | PDFSTATSENSEMBLUCSC | 350 | 7 | 35296 | tss | 0.050007 | 0.566628 | PPP1R14C (margin: PPP1R14C) | 4.13 | 4.08e-03 | 2.47e-02 |
|  | 6 | 156718177 | 156718546 | PDFSTATSENSEMBLUCSC | 370 | 5 | 36589 | island | 0.587032 | 0.965090 | (margin: ) | 4.12 | 7.94e-03 | 3.19e-02 |
|  | 2 | 154335348 | 154335386 | PDFSTATSENSEMBLUCSC | 39 | 5 | 23667 | tss | 0.081115 | 0.004012 | AC012501.2; RPRM (margin: AC012501.2; RPRM) | 4.12 | 7.94e-03 | 3.19e-02 |
|  | 10 | 28034792 | 28034856 | PDFSTATSENSEMBLUCSC | 65 | 6 | 5004 | tss | 0.720930 | 0.975219 | RP11-360I20.2; MKX (margin: RP11-360I20.2; MKX) | 4.12 | 2.16e-03 | 1.45e-02 |
|  | 6 | 147829799 | 147830055 | PDFSTATSENSEMBLUCSC | 257 | 7 | 35279 | tss | 0.724120 | 0.984634 | SAMD5 (margin: SAMD5) | 4.12 | 5.83e-04 | 6.47e-03 |
|  | 6 | 28602543 | 28603437 | PDFSTATSENSEMBLUCSC | 895 | 35 | 35593 | island | 0.554668 | 0.977224 | RP11-373N24.2 (margin: RP11-373N24.2) | 4.11 | 5.28e-12 | 6.43e-09 |
|  | 19 | 18548845 | 18549274 | PDFSTATSENSEMBLUCSC | 430 | 8 | 21779 | island | 0.461350 | 0.044994 | ISYNA1 (margin: SSBP4; CTD-3137H5.1; ISYNA1; ELL; AC010335.1) | 4.11 | 1.86e-03 | 1.45e-02 |
|  | 8 | 144635260 | 144635610 | PDFSTATSENSEMBLUCSC | 351 | 10 | 40668 | island | 0.546792 | 0.954589 | GSDMD (margin: 7SK; GSDMD; ZC3H3; RP11-661A12.5) | 4.11 | 1.08e-05 | 4.82e-04 |
|  | 7 | 145813413 | 145813439 | PDFSTATSENSEMBLUCSC | 27 | 6 | 37959 | tss | 0.946067 | 0.518228 | CNTNAP2 (margin: CNTNAP2) | 4.11 | 2.16e-03 | 1.45e-02 |
|  | 19 | 47217554 | 47218031 | PDFSTATSENSEMBLUCSC | 478 | 6 | 20112 | gene;island | 0.013729 | 0.206649 | PRKD2 (margin: PRKD2; MIR320E; STRN4) | 4.11 | 8.66e-03 | 3.36e-02 |
|  | 6 | 137241923 | 137242316 | PDFSTATSENSEMBLUCSC | 394 | 5 | 35245 | tss;island | 0.795848 | 0.983993 | SLC35D3 (margin: PEX7; SLC35D3) | 4.11 | 7.94e-03 | 3.19e-02 |
|  | 10 | 61122307 | 61122716 | PDFSTATSENSEMBLUCSC | 410 | 10 | 5762 | island | 0.112351 | 0.006552 | FAM13C (margin: FAM13C) | 4.11 | 1.05e-03 | 1.08e-02 |
|  | 20 | 33146263 | 33146543 | PDFSTATSENSEMBLUCSC | 281 | 6 | 25400 | gene;island | 0.714251 | 0.975221 | MAP1LC3A; PIGU (margin: MAP1LC3A; PIGU) | 4.11 | 2.16e-03 | 1.45e-02 |
|  | 1 | 27113508 | 27113753 | PDFSTATSENSEMBLUCSC | 246 | 6 | 1396 | tss;island | 0.025221 | 0.319160 | PIGV (margin: ARID1A; PIGV) | 4.10 | 2.16e-03 | 1.45e-02 |
|  | 6 | 29394438 | 29395425 | PDFSTATSENSEMBLUCSC | 988 | 12 | 33655 | gene | 0.083188 | 0.704719 | OR5V1; OR11A1 (margin: OR12D1P; OR10C1; OR5V1; OR11A1) | 4.10 | 3.71e-04 | 5.59e-03 |
|  | 13 | 112760898 | 112761328 | PDFSTATSENSEMBLUCSC | 431 | 6 | 11916 | island | 0.566720 | 0.969552 | LINC00403; LINC00404 (margin: LINC00403; LINC00404) | 4.10 | 2.16e-03 | 1.45e-02 |
|  | 20 | 30060822 | 30061044 | PDFSTATSENSEMBLUCSC | 223 | 5 | 25658 | tss;island | 0.282652 | 0.870410 | DEFB124 (margin: REM1; DEFB124) | 4.10 | 7.94e-03 | 3.19e-02 |
|  | 22 | 38071455 | 38071677 | PDFSTATSENSEMBLUCSC | 223 | 7 | 27468 | island | 0.019314 | 0.225449 | LGALS1 (margin: SH3BP1; PDXP; LGALS1; NOL12; RP1-37E16.12; RN7SL385P) | 4.10 | 5.83e-04 | 6.47e-03 |
|  | 10 | 13933996 | 13934438 | PDFSTATSENSEMBLUCSC | 443 | 5 | 4505 | gene;island | 0.557331 | 0.950449 | FRMD4A (margin: FRMD4A) | 4.10 | 7.94e-03 | 3.19e-02 |
|  | 12 | 12849136 | 12849748 | PDFSTATSENSEMBLUCSC | 613 | 8 | 9573 | tss | 0.091486 | 0.006824 | GPR19 (margin: RP11-180M15.3; GPR19) | 4.09 | 1.04e-02 | 3.96e-02 |
|  | 12 | 115134148 | 115136308 | PDFSTATSENSEMBLUCSC | 2161 | 54 | 10850 | island | 0.614152 | 0.980693 | (margin: ) | 4.09 | 1.12e-13 | 2.74e-10 |
|  | 10 | 105212056 | 105212813 | PDFSTATSENSEMBLUCSC | 758 | 12 | 6102 | island | 0.598580 | 0.197777 | RP11-225H22.4; CALHM2; CALHM1 (margin: PDCD11; RP11-225H22.4; CALHM2; CALHM1) | 4.09 | 2.01e-04 | 3.38e-03 |
|  | 19 | 18548929 | 18549274 | PDFSTATSENSEMBLUCSC | 346 | 7 | 20619 | tss | 0.511873 | 0.047746 | ISYNA1 (margin: SSBP4; CTD-3137H5.1; ISYNA1; ELL; AC010335.1) | 4.09 | 6.99e-03 | 3.19e-02 |
|  | 11 | 66034681 | 66036138 | PDFSTATSENSEMBLUCSC | 1458 | 58 | 8500 | island | 0.282802 | 0.009742 | KLC2; RAB1B; RP11-867G23.1; RP11-867G23.2; RP11-867G23.3 (margin: KLC2; RAB1B; CNIH2; RP11-755F10.3; RP11-867G23.1; RP11-867G23.2; RP11-867G23.3; RP11-867G23.4) | 4.08 | 4.18e-06 | 2.75e-04 |
|  | 7 | 73062083 | 73062573 | PDFSTATSENSEMBLUCSC | 491 | 5 | 38562 | island | 0.028042 | 0.365765 | (margin: ) | 4.08 | 7.94e-03 | 3.19e-02 |
|  | 6 | 6546777 | 6547273 | PDFSTATSENSEMBLUCSC | 497 | 5 | 33547 | gene;island | 0.497510 | 0.940942 | LY86-AS1 (margin: CNN3P1; LY86-AS1) | 4.08 | 7.94e-03 | 3.19e-02 |
|  | 22 | 39712956 | 39713086 | PDFSTATSENSEMBLUCSC | 131 | 6 | 26876 | gene;tss;island | 0.063532 | 0.530907 | RPL3; SNORD83A; SNORD43 (margin: RPL3; SNORD83B; SNORD83A; SNORD43) | 4.08 | 2.16e-03 | 1.45e-02 |
|  | 20 | 30073209 | 30073576 | PDFSTATSENSEMBLUCSC | 368 | 6 | 25660 | tss;island | 0.496932 | 0.852025 | REM1; LINC00028 (margin: REM1; LINC00028; DEFB124) | 4.07 | 4.33e-03 | 2.52e-02 |
|  | 1 | 23504146 | 23504632 | PDFSTATSENSEMBLUCSC | 487 | 6 | 2830 | island | 0.086265 | 0.478604 | LUZP1 (margin: LUZP1) | 4.07 | 2.16e-03 | 1.45e-02 |
|  | 20 | 42875645 | 42876091 | PDFSTATSENSEMBLUCSC | 447 | 9 | 26180 | island | 0.579934 | 0.912764 | GDAP1L1 (margin: GDAP1L1) | 4.07 | 1.23e-03 | 1.18e-02 |
|  | 2 | 37571677 | 37571901 | PDFSTATSENSEMBLUCSC | 225 | 6 | 24223 | island | 0.973969 | 0.653280 | QPCT (margin: QPCT) | 4.06 | 2.16e-03 | 1.45e-02 |
|  | 10 | 124713989 | 124714384 | PDFSTATSENSEMBLUCSC | 396 | 8 | 5412 | tss | 0.619593 | 0.153328 | PSTK; C10orf88 (margin: PSTK; C10orf88) | 4.06 | 1.04e-02 | 3.96e-02 |
|  | 19 | 53636226 | 53636398 | PDFSTATSENSEMBLUCSC | 173 | 5 | 21023 | tss | 0.217989 | 0.951707 | ZNF415; ZNF347; CTD-2620I22.7 (margin: ZNF415; ZNF347; CTD-2620I22.7) | 4.06 | 7.94e-03 | 3.19e-02 |
|  | 22 | 29711916 | 29712139 | PDFSTATSENSEMBLUCSC | 224 | 6 | 27047 | tss;island | 0.696767 | 0.983027 | RASL10A (margin: GAS2L1; RASL10A; AP1B1) | 4.06 | 8.66e-03 | 3.36e-02 |
|  | 1 | 165204745 | 165205506 | PDFSTATSENSEMBLUCSC | 762 | 12 | 863 | gene;island | 0.558146 | 0.956989 | LMX1A (margin: LMX1A) | 4.06 | 1.03e-04 | 2.47e-03 |
|  | 10 | 26726877 | 26727318 | PDFSTATSENSEMBLUCSC | 442 | 8 | 5619 | island | 0.491990 | 0.933286 | APBB1IP (margin: APBB1IP) | 4.05 | 6.22e-04 | 6.71e-03 |
|  | 20 | 57425979 | 57428032 | PDFSTATSENSEMBLUCSC | 2054 | 66 | 25471 | gene;tss;island | 0.490360 | 0.950384 | GNAS; GNAS-AS1 (margin: GNAS; GNAS-AS1; RP1-309F20.3) | 4.05 | 3.02e-20 | 2.94e-16 |
|  | 12 | 79257496 | 79257853 | PDFSTATSENSEMBLUCSC | 358 | 6 | 9890 | tss | 0.284925 | 0.016648 | SYT1 (margin: SYT1) | 4.05 | 4.33e-03 | 2.52e-02 |
|  | 13 | 113652069 | 113652368 | PDFSTATSENSEMBLUCSC | 300 | 5 | 11266 | gene;island | 0.601177 | 0.954141 | MCF2L (margin: MCF2L; RP11-120K24.4; RP11-120K24.5) | 4.05 | 7.94e-03 | 3.19e-02 |
|  | 3 | 8543014 | 8543732 | PDFSTATSENSEMBLUCSC | 719 | 10 | 28849 | island | 0.132628 | 0.007899 | LMCD1; LMCD1-AS1 (margin: LMCD1; LMCD1-AS1) | 4.05 | 8.93e-03 | 3.45e-02 |
|  | 17 | 1956958 | 1958478 | PDFSTATSENSEMBLUCSC | 1521 | 45 | 17001 | tss;island | 0.423165 | 0.020299 | HIC1 (margin: DPH1; OVCA2; HIC1; RP11-667K14.4; RP11-667K14.3; MIR132; MIR212; SMG6) | 4.04 | 9.96e-11 | 6.93e-08 |
|  | 12 | 4554295 | 4554756 | PDFSTATSENSEMBLUCSC | 462 | 6 | 9016 | gene | 0.179440 | 0.793615 | FGF6 (margin: FGF6) | 4.04 | 8.66e-03 | 3.36e-02 |
|  | 7 | 18535072 | 18535499 | PDFSTATSENSEMBLUCSC | 428 | 6 | 37458 | tss | 0.597630 | 0.197771 | HDAC9 (margin: HDAC9) | 4.04 | 4.33e-03 | 2.52e-02 |
|  | 8 | 11560299 | 11560851 | PDFSTATSENSEMBLUCSC | 553 | 12 | 39552 | tss;island | 0.607764 | 0.964762 | GATA4 (margin: GATA4) | 4.04 | 6.56e-04 | 7.02e-03 |
|  | 10 | 25464008 | 25464513 | PDFSTATSENSEMBLUCSC | 506 | 12 | 4533 | gene;island | 0.728679 | 0.097295 | GPR158; GPR158-AS1 (margin: GPR158; GPR158-AS1) | 4.03 | 5.56e-03 | 3.16e-02 |
|  | 1 | 11919018 | 11919699 | PDFSTATSENSEMBLUCSC | 682 | 9 | 1252 | tss | 0.126322 | 0.034986 | NPPB (margin: NPPA-AS1; NPPA; NPPB) | 4.03 | 2.76e-03 | 1.78e-02 |
|  | 6 | 80656586 | 80656973 | PDFSTATSENSEMBLUCSC | 388 | 5 | 34399 | gene;island | 0.224159 | 0.021972 | ELOVL4 (margin: ELOVL4; GAPDHP63) | 4.03 | 7.94e-03 | 3.19e-02 |
|  | 11 | 35160462 | 35160892 | PDFSTATSENSEMBLUCSC | 431 | 6 | 6583 | gene | 0.008193 | 0.086509 | CD44; RP4-607I7.1 (margin: CD44; AL356215.1; RP4-607I7.1) | 4.03 | 8.66e-03 | 3.36e-02 |
|  | 15 | 81426347 | 81426820 | PDFSTATSENSEMBLUCSC | 474 | 10 | 14378 | island | 0.830843 | 0.980661 | C15orf26 (margin: C15orf26) | 4.03 | 7.25e-04 | 7.74e-03 |
|  | 15 | 81426347 | 81426610 | PDFSTATSENSEMBLUCSC | 264 | 8 | 13852 | tss | 0.831292 | 0.981286 | C15orf26 (margin: C15orf26) | 4.03 | 2.95e-03 | 1.88e-02 |
|  | 12 | 57634549 | 57634855 | PDFSTATSENSEMBLUCSC | 307 | 7 | 9814 | tss | 0.109171 | 0.017123 | NDUFA4L2 (margin: SHMT2; NDUFA4L2; STAC3; RP11-123K3.4; R3HDM2) | 4.03 | 6.99e-03 | 3.19e-02 |
|  | 1 | 68515788 | 68516713 | PDFSTATSENSEMBLUCSC | 926 | 17 | 3369 | island | 0.614361 | 0.955184 | GNG12-AS1; DIRAS3; ARL5AP3 (margin: GNG12-AS1; RP4-609E1.2; AL157407.1; DIRAS3; ARL5AP3) | 4.03 | 2.52e-04 | 4.14e-03 |
|  | 1 | 1935048 | 1935561 | PDFSTATSENSEMBLUCSC | 514 | 6 | 2467 | island | 0.521313 | 0.957298 | C1orf222 (margin: C1orf222; RP11-547D24.1) | 4.02 | 2.16e-03 | 1.45e-02 |
|  | 7 | 30029717 | 30029808 | PDFSTATSENSEMBLUCSC | 92 | 6 | 36952 | gene | 0.080215 | 0.506810 | AC007285.6; SCRN1 (margin: AC007285.6; SCRN1) | 4.02 | 2.16e-03 | 1.45e-02 |
|  | 7 | 120968877 | 120969174 | PDFSTATSENSEMBLUCSC | 298 | 8 | 37203 | gene;island | 0.827127 | 0.216850 | WNT16 (margin: WNT16) | 4.02 | 1.55e-04 | 2.71e-03 |
|  | 11 | 93583672 | 93583973 | PDFSTATSENSEMBLUCSC | 302 | 6 | 7626 | tss | 0.042885 | 0.418688 | VSTM5 (margin: VSTM5) | 4.01 | 2.16e-03 | 1.45e-02 |
|  | 6 | 33171765 | 33172572 | PDFSTATSENSEMBLUCSC | 808 | 16 | 35978 | island | 0.042213 | 0.427258 | SLC39A7; HSD17B8 (margin: RNY4P10; SLC39A7; HSD17B8; MIR219-1; RING1; ZNF70P1; COL11A2; RXRB) | 4.01 | 1.94e-03 | 1.45e-02 |
|  | 8 | 140716495 | 140716766 | PDFSTATSENSEMBLUCSC | 272 | 6 | 39924 | tss;island | 0.319875 | 0.039138 | KCNK9 (margin: KCNK9) | 4.01 | 2.16e-03 | 1.45e-02 |
|  | 5 | 87974369 | 87974547 | PDFSTATSENSEMBLUCSC | 179 | 7 | 31680 | gene;tss;island | 0.816910 | 0.989267 | CTC-467M3.1; LINC00461 (margin: CTC-467M3.1; LINC00461) | 4.01 | 2.33e-03 | 1.52e-02 |
|  | 19 | 41510026 | 41510285 | PDFSTATSENSEMBLUCSC | 260 | 7 | 20052 | gene | 0.701181 | 0.968617 | CYP2B6 (margin: CYP2B6) | 4.00 | 6.99e-03 | 3.19e-02 |
|  | 14 | 54421381 | 54421578 | PDFSTATSENSEMBLUCSC | 198 | 7 | 12083 | gene;tss;island | 0.612948 | 0.955923 | BMP4 (margin: MIR5580; BMP4) | 4.00 | 5.83e-04 | 6.47e-03 |
|  | 7 | 27213966 | 27214383 | PDFSTATSENSEMBLUCSC | 418 | 8 | 37498 | tss | 0.889836 | 0.989316 | RP1-170O19.20; HOXA10 (margin: HOXA-AS4; HOXA11-AS; HOXA9; RP1-170O19.20; MIR196B; HOXA10; HOXA11; RP1-170O19.14) | 4.00 | 6.22e-04 | 6.71e-03 |
|  | 1 | 204966000 | 204966512 | PDFSTATSENSEMBLUCSC | 513 | 7 | 962 | gene | 0.498554 | 0.943485 | NFASC (margin: NFASC) | 4.00 | 4.08e-03 | 2.47e-02 |
|  | 11 | 70508022 | 70508659 | PDFSTATSENSEMBLUCSC | 638 | 10 | 7529 | tss | 0.600272 | 0.949345 | SHANK2 (margin: SHANK2) | 4.00 | 7.58e-05 | 1.98e-03 |
|  | 8 | 119963842 | 119964729 | PDFSTATSENSEMBLUCSC | 888 | 11 | 40556 | island | 0.645056 | 0.043316 | TNFRSF11B (margin: TNFRSF11B) | 4.00 | 1.28e-02 | 4.76e-02 |
|  | 7 | 4264980 | 4265548 | PDFSTATSENSEMBLUCSC | 569 | 6 | 36836 | gene | 0.433500 | 0.950254 | SDK1 (margin: SDK1) | 4.00 | 2.16e-03 | 1.45e-02 |
|  | 11 | 70507825 | 70508659 | PDFSTATSENSEMBLUCSC | 835 | 11 | 6793 | gene;island | 0.620673 | 0.957428 | SHANK2 (margin: SHANK2) | 3.99 | 1.98e-05 | 8.44e-04 |
|  | 1 | 36038468 | 36038950 | PDFSTATSENSEMBLUCSC | 483 | 7 | 1485 | tss;island | 0.654418 | 0.985138 | TFAP2E; RP4-728D4.2 (margin: NCDN; TFAP2E; RP4-728D4.2) | 3.99 | 6.99e-03 | 3.19e-02 |
|  | 3 | 153838788 | 153839142 | PDFSTATSENSEMBLUCSC | 355 | 7 | 28667 | tss | 0.256371 | 0.010669 | ARHGEF26; ARHGEF26-AS1 (margin: ARHGEF26; ARHGEF26-AS1) | 3.98 | 6.99e-03 | 3.19e-02 |
|  | 8 | 40755380 | 40755559 | PDFSTATSENSEMBLUCSC | 180 | 5 | 39666 | tss | 0.218149 | 0.017408 | ZMAT4; RP11-465K16.1 (margin: ZMAT4; RP11-465K16.1) | 3.98 | 7.94e-03 | 3.19e-02 |
|  | 17 | 7348316 | 7348490 | PDFSTATSENSEMBLUCSC | 175 | 8 | 17950 | island | 0.957797 | 0.619744 | RP11-104H15.7; FGF11; CHRNB1; RP11-104H15.8 (margin: RP11-104H15.7; TMEM102; FGF11; CHRNB1; RP11-104H15.9; RP11-104H15.8) | 3.98 | 1.55e-04 | 2.71e-03 |
|  | 17 | 7348316 | 7348399 | PDFSTATSENSEMBLUCSC | 84 | 6 | 17084 | tss | 0.957797 | 0.619744 | RP11-104H15.7; FGF11; CHRNB1; RP11-104H15.8 (margin: RP11-104H15.7; TMEM102; FGF11; CHRNB1; RP11-104H15.9; RP11-104H15.8) | 3.98 | 2.16e-03 | 1.45e-02 |
|  | 18 | 61143926 | 61143993 | PDFSTATSENSEMBLUCSC | 68 | 5 | 19312 | tss | 0.001991 | 0.025353 | SERPINB5 (margin: SERPINB5; RP11-635N19.3) | 3.98 | 7.94e-03 | 3.19e-02 |
|  | 14 | 24505537 | 24505898 | PDFSTATSENSEMBLUCSC | 362 | 7 | 12724 | island | 0.008592 | 0.339748 | DHRS4L1 (margin: DHRS4L1) | 3.98 | 1.11e-02 | 4.14e-02 |
|  | 11 | 1948933 | 1949113 | PDFSTATSENSEMBLUCSC | 181 | 5 | 6439 | gene;island | 0.649414 | 0.967303 | TNNT3 (margin: TNNT3; AC051649.1) | 3.97 | 7.94e-03 | 3.19e-02 |
|  | 7 | 82792239 | 82792411 | PDFSTATSENSEMBLUCSC | 173 | 7 | 37678 | tss | 0.146606 | 0.007479 | PCLO (margin: PCLO) | 3.97 | 2.33e-03 | 1.52e-02 |
|  | 2 | 496713 | 497417 | PDFSTATSENSEMBLUCSC | 705 | 7 | 24004 | island | 0.561086 | 0.909061 | (margin: AC093326.1) | 3.97 | 4.08e-03 | 2.47e-02 |
|  | 14 | 76044591 | 76045025 | PDFSTATSENSEMBLUCSC | 435 | 6 | 13018 | island | 0.208654 | 0.036531 | FLVCR2; AC007182.6 (margin: FLVCR2; AC007182.6) | 3.97 | 8.66e-03 | 3.36e-02 |
|  | 3 | 9811714 | 9811990 | PDFSTATSENSEMBLUCSC | 277 | 7 | 28130 | tss;island | 0.702927 | 0.164571 | OGG1; CAMK1 (margin: OGG1; CAMK1; TADA3) | 3.96 | 5.83e-04 | 6.47e-03 |
|  | 17 | 65040527 | 65040616 | PDFSTATSENSEMBLUCSC | 90 | 6 | 17596 | tss | 0.595661 | 0.972321 | CACNG1 (margin: CACNG4; CACNG1; RP11-74H8.1) | 3.96 | 2.16e-03 | 1.45e-02 |
|  | 1 | 3207371 | 3207588 | PDFSTATSENSEMBLUCSC | 218 | 5 | 117 | gene;island | 0.558802 | 0.960104 | PRDM16 (margin: PRDM16) | 3.96 | 7.94e-03 | 3.19e-02 |
|  | 6 | 112575850 | 112576119 | PDFSTATSENSEMBLUCSC | 270 | 5 | 35183 | tss | 0.468805 | 0.942590 | RP11-506B6.6; LAMA4 (margin: RP11-506B6.6; LAMA4) | 3.95 | 7.94e-03 | 3.19e-02 |
|  | 11 | 8615506 | 8616172 | PDFSTATSENSEMBLUCSC | 667 | 14 | 7126 | tss | 0.802201 | 0.975519 | STK33 (margin: STK33) | 3.95 | 1.36e-05 | 5.85e-04 |
|  | 2 | 241938159 | 241938193 | PDFSTATSENSEMBLUCSC | 35 | 5 | 23983 | tss | 0.114432 | 0.008232 | SNED1 (margin: AC104809.3; SNED1; AC093585.6) | 3.94 | 7.94e-03 | 3.19e-02 |
|  | 11 | 61159602 | 61159837 | PDFSTATSENSEMBLUCSC | 236 | 7 | 7334 | tss;island | 0.048030 | 0.437402 | TMEM216 (margin: TMEM216; CPSF7) | 3.94 | 1.11e-02 | 4.14e-02 |
|  | 11 | 2321770 | 2322050 | PDFSTATSENSEMBLUCSC | 281 | 9 | 6453 | gene;tss | 0.599768 | 0.946469 | TSPAN32; C11orf21 (margin: TSPAN32; C11orf21) | 3.93 | 4.11e-05 | 1.17e-03 |
|  | 10 | 47151300 | 47151589 | PDFSTATSENSEMBLUCSC | 290 | 5 | 5720 | island | 0.019695 | 0.239619 | LINC00842 (margin: LINC00842; ANXA8L1) | 3.93 | 7.94e-03 | 3.19e-02 |
|  | 19 | 19648890 | 19649054 | PDFSTATSENSEMBLUCSC | 165 | 7 | 20645 | tss | 0.283654 | 0.785204 | YJEFN3; CILP2 (margin: NDUFA13; CTC-260F20.3; YJEFN3; CILP2) | 3.93 | 5.83e-04 | 6.47e-03 |
|  | 11 | 69519088 | 69519437 | PDFSTATSENSEMBLUCSC | 350 | 8 | 8596 | island | 0.284265 | 0.673886 | FGF19 (margin: FGF19) | 3.93 | 1.09e-03 | 1.10e-02 |
|  | 12 | 72666976 | 72667707 | PDFSTATSENSEMBLUCSC | 732 | 10 | 9218 | gene;island | 0.281286 | 0.854783 | TRHDE; TRHDE-AS1 (margin: TRHDE; TRHDE-AS1) | 3.93 | 2.17e-05 | 8.94e-04 |
|  | 19 | 23941207 | 23941575 | PDFSTATSENSEMBLUCSC | 369 | 5 | 19966 | gene | 0.517815 | 0.016040 | ZNF681 (margin: RP11-255H23.2; RPSAP58; ZNF681) | 3.93 | 7.94e-03 | 3.19e-02 |
|  | 12 | 103889516 | 103890274 | PDFSTATSENSEMBLUCSC | 759 | 11 | 10731 | island | 0.443048 | 0.046486 | C12orf42 (margin: RP11-626I20.1; C12orf42; RP11-626I20.2) | 3.93 | 1.40e-03 | 1.33e-02 |
|  | 3 | 152552410 | 152552615 | PDFSTATSENSEMBLUCSC | 206 | 7 | 28665 | tss;island | 0.223008 | 0.017734 | P2RY1 (margin: P2RY1; RP11-38P22.2) | 3.92 | 4.08e-03 | 2.47e-02 |
|  | 4 | 1504989 | 1505559 | PDFSTATSENSEMBLUCSC | 571 | 6 | 30788 | island | 0.438145 | 0.921064 | (margin: ) | 3.92 | 4.33e-03 | 2.52e-02 |
|  | 10 | 135039948 | 135040250 | PDFSTATSENSEMBLUCSC | 303 | 5 | 6345 | island | 0.386666 | 0.883825 | KNDC1 (margin: KNDC1; UTF1; VENTX) | 3.92 | 7.94e-03 | 3.19e-02 |
|  | 15 | 50474069 | 50474388 | PDFSTATSENSEMBLUCSC | 320 | 7 | 13665 | tss;island | 0.839302 | 0.291634 | SLC27A2; ATP8B4 (margin: SLC27A2; ATP8B4) | 3.92 | 5.83e-04 | 6.47e-03 |
|  | 16 | 88937115 | 88937386 | PDFSTATSENSEMBLUCSC | 272 | 5 | 16226 | island | 0.402063 | 0.884683 | (margin: TRAPPC2L; RP11-830F9.5; PABPN1L; CBFA2T3) | 3.91 | 7.94e-03 | 3.19e-02 |
|  | 1 | 109204168 | 109204325 | PDFSTATSENSEMBLUCSC | 158 | 5 | 1790 | tss;island | 0.814424 | 0.225785 | HENMT1 (margin: HENMT1) | 3.91 | 7.94e-03 | 3.19e-02 |
|  | 12 | 15475116 | 15475767 | PDFSTATSENSEMBLUCSC | 652 | 10 | 10269 | island | 0.202319 | 0.804411 | PTPRO; RERG (margin: PTPRO; RERG) | 3.91 | 7.58e-05 | 1.98e-03 |
|  | 11 | 110583377 | 110583662 | PDFSTATSENSEMBLUCSC | 286 | 7 | 8794 | island | 0.458990 | 0.906887 | ARHGAP20 (margin: ARHGAP20) | 3.91 | 1.17e-03 | 1.13e-02 |
|  | 16 | 67184918 | 67185194 | PDFSTATSENSEMBLUCSC | 277 | 6 | 15292 | tss;island | 0.672650 | 0.947783 | B3GNT9 (margin: C16orf70; FBXL8; B3GNT9; TRADD) | 3.91 | 2.16e-03 | 1.45e-02 |
|  | 12 | 2800055 | 2800500 | PDFSTATSENSEMBLUCSC | 446 | 8 | 9006 | gene;island | 0.648254 | 0.926792 | CACNA1C; CACNA1C-AS1 (margin: CACNA1C; CACNA1C-AS1) | 3.91 | 6.22e-04 | 6.71e-03 |
|  | 19 | 19648890 | 19649144 | PDFSTATSENSEMBLUCSC | 255 | 8 | 21821 | island | 0.291595 | 0.818622 | YJEFN3; CILP2 (margin: NDUFA13; CTC-260F20.3; YJEFN3; CILP2) | 3.90 | 1.55e-04 | 2.71e-03 |
|  | 2 | 131010423 | 131010871 | PDFSTATSENSEMBLUCSC | 449 | 5 | 24717 | island | 0.624089 | 0.961102 | MED15P5 (margin: AC134915.1; MED15P5) | 3.90 | 7.94e-03 | 3.19e-02 |
|  | 10 | 134095646 | 134096014 | PDFSTATSENSEMBLUCSC | 369 | 5 | 4860 | gene;island | 0.183742 | 0.769052 | STK32C (margin: STK32C) | 3.90 | 7.94e-03 | 3.19e-02 |
|  | 6 | 147829799 | 147830172 | PDFSTATSENSEMBLUCSC | 374 | 8 | 36549 | island | 0.736613 | 0.977233 | SAMD5 (margin: SAMD5) | 3.90 | 1.55e-04 | 2.71e-03 |
|  | 1 | 32827707 | 32828191 | PDFSTATSENSEMBLUCSC | 485 | 9 | 395 | gene;tss;island | 0.660730 | 0.115467 | TSSK3; FAM229A (margin: TSSK3; RP4-811H24.9; FAM229A; BSDC1) | 3.90 | 3.99e-03 | 2.47e-02 |
|  | 4 | 174451141 | 174451564 | PDFSTATSENSEMBLUCSC | 424 | 11 | 30682 | tss;island | 0.432426 | 0.954492 | HAND2-AS1; HAND2 (margin: HAND2-AS1; HAND2) | 3.89 | 8.51e-05 | 2.07e-03 |
|  | 11 | 8615356 | 8616172 | PDFSTATSENSEMBLUCSC | 817 | 16 | 8033 | island | 0.817690 | 0.970300 | STK33 (margin: STK33) | 3.89 | 8.94e-06 | 4.82e-04 |
|  | 20 | 45985495 | 45985741 | PDFSTATSENSEMBLUCSC | 247 | 7 | 25799 | tss | 0.097296 | 0.614412 | ZMYND8 (margin: RP4-569M23.2; ZMYND8) | 3.89 | 5.83e-04 | 6.47e-03 |
|  | 6 | 24358236 | 24358327 | PDFSTATSENSEMBLUCSC | 92 | 5 | 33590 | gene;island | 0.897507 | 0.348200 | KAAG1; DCDC2 (margin: KAAG1; RNU6-391P; DCDC2) | 3.88 | 7.94e-03 | 3.19e-02 |
|  | 1 | 44870976 | 44871634 | PDFSTATSENSEMBLUCSC | 659 | 7 | 489 | gene | 0.266203 | 0.015093 | RNF220 (margin: RNF220) | 3.88 | 2.33e-03 | 1.52e-02 |
|  | 11 | 75379432 | 75379624 | PDFSTATSENSEMBLUCSC | 193 | 7 | 8665 | island | 0.559345 | 0.941002 | MAP6 (margin: MAP6) | 3.88 | 5.83e-04 | 6.47e-03 |
|  | 2 | 175199259 | 175199828 | PDFSTATSENSEMBLUCSC | 570 | 12 | 24874 | island | 0.601333 | 0.976828 | SP9; AC018470.1 (margin: AC018470.4; SP9; AC018470.1) | 3.88 | 7.17e-05 | 1.92e-03 |
|  | 17 | 12569089 | 12569487 | PDFSTATSENSEMBLUCSC | 399 | 11 | 18028 | island | 0.371737 | 0.861079 | MYOCD (margin: MYOCD; AC005358.3) | 3.87 | 1.90e-04 | 3.21e-03 |
|  | 17 | 12569089 | 12569204 | PDFSTATSENSEMBLUCSC | 116 | 7 | 17141 | tss | 0.579174 | 0.928174 | MYOCD (margin: MYOCD; AC005358.3) | 3.87 | 5.83e-04 | 6.47e-03 |
|  | 12 | 85306323 | 85307152 | PDFSTATSENSEMBLUCSC | 830 | 13 | 10656 | island | 0.357884 | 0.945460 | SLC6A15 (margin: SLC6A15) | 3.87 | 5.23e-05 | 1.45e-03 |
|  | 7 | 4118583 | 4118868 | PDFSTATSENSEMBLUCSC | 286 | 5 | 36826 | gene | 0.393886 | 0.876023 | SDK1 (margin: SDK1) | 3.87 | 7.94e-03 | 3.19e-02 |
|  | 2 | 176987161 | 176987605 | PDFSTATSENSEMBLUCSC | 445 | 8 | 24902 | island | 0.843578 | 0.986353 | HOXD9; HOXD-AS2 (margin: HOXD10; HOXD9; HOXD8; HOXD-AS2) | 3.87 | 3.11e-04 | 4.77e-03 |
|  | 11 | 69590113 | 69590580 | PDFSTATSENSEMBLUCSC | 468 | 8 | 8597 | island | 0.518482 | 0.955893 | FGF4 (margin: AP001888.1; FGF4) | 3.86 | 1.55e-04 | 2.71e-03 |
|  | 11 | 36422377 | 36422615 | PDFSTATSENSEMBLUCSC | 239 | 5 | 6585 | gene | 0.644785 | 0.968642 | PRR5L (margin: PRR5L) | 3.86 | 7.94e-03 | 3.19e-02 |
|  | 3 | 13114343 | 13114803 | PDFSTATSENSEMBLUCSC | 461 | 6 | 28901 | island | 0.530338 | 0.954589 | IQSEC1 (margin: IQSEC1) | 3.86 | 8.66e-03 | 3.36e-02 |
|  | 3 | 48697409 | 48697823 | PDFSTATSENSEMBLUCSC | 415 | 8 | 27769 | gene;island | 0.667706 | 0.939987 | CELSR3 (margin: RP11-148G20.1; CELSR3; NCKIPSD) | 3.85 | 4.66e-03 | 2.68e-02 |
|  | 6 | 32942063 | 32942808 | PDFSTATSENSEMBLUCSC | 746 | 10 | 34171 | gene;island | 0.004822 | 0.058971 | BRD2; XXbac-BPG181M17.6 (margin: BRD2; BRD2-IT1; HLA-DMA; XXbac-BPG181M17.6) | 3.85 | 3.89e-03 | 2.43e-02 |
|  | 3 | 196366191 | 196366669 | PDFSTATSENSEMBLUCSC | 479 | 8 | 29810 | island | 0.638609 | 0.942516 | NRROS; PIGX (margin: NRROS; PIGX; AC023797.1) | 3.84 | 2.95e-03 | 1.88e-02 |
|  | 3 | 179754483 | 179754761 | PDFSTATSENSEMBLUCSC | 279 | 8 | 29683 | island | 0.906395 | 0.387603 | PEX5L (margin: PEX5L) | 3.84 | 1.86e-03 | 1.45e-02 |
|  | 21 | 42688468 | 42689051 | PDFSTATSENSEMBLUCSC | 584 | 9 | 26687 | island | 0.839102 | 0.986997 | FAM3B (margin: FAM3B) | 3.84 | 8.23e-05 | 2.06e-03 |
|  | 21 | 42688468 | 42688650 | PDFSTATSENSEMBLUCSC | 183 | 5 | 26555 | tss | 0.790298 | 0.986997 | FAM3B (margin: FAM3B) | 3.84 | 7.94e-03 | 3.19e-02 |
|  | 10 | 135050619 | 135051351 | PDFSTATSENSEMBLUCSC | 733 | 25 | 5456 | tss | 0.548626 | 0.958874 | VENTX (margin: KNDC1; UTF1; VENTX; MIR202) | 3.83 | 1.86e-10 | 1.13e-07 |
|  | 7 | 75157456 | 75157765 | PDFSTATSENSEMBLUCSC | 310 | 7 | 37654 | tss | 0.007502 | 0.047012 | PMS2P3 (margin: PMS2P3; HIP1) | 3.83 | 2.33e-03 | 1.52e-02 |
|  | 1 | 228473704 | 228473980 | PDFSTATSENSEMBLUCSC | 277 | 5 | 1054 | gene;island | 0.491288 | 0.961735 | OBSCN (margin: OBSCN; RP5-1139B12.2; RP5-1139B12.3; RP5-1139B12.4) | 3.83 | 7.94e-03 | 3.19e-02 |
|  | 11 | 93583509 | 93583973 | PDFSTATSENSEMBLUCSC | 465 | 8 | 8733 | island | 0.048085 | 0.390377 | VSTM5 (margin: VSTM5) | 3.83 | 1.55e-04 | 2.71e-03 |
|  | 12 | 85306648 | 85307152 | PDFSTATSENSEMBLUCSC | 505 | 9 | 9898 | tss | 0.357884 | 0.880173 | SLC6A15 (margin: SLC6A15) | 3.83 | 3.99e-03 | 2.47e-02 |
|  | 11 | 75379491 | 75379624 | PDFSTATSENSEMBLUCSC | 134 | 6 | 7570 | tss | 0.551989 | 0.922910 | MAP6 (margin: MAP6) | 3.82 | 2.16e-03 | 1.45e-02 |
|  | 3 | 111717682 | 111718245 | PDFSTATSENSEMBLUCSC | 564 | 8 | 27893 | gene | 0.520577 | 0.957080 | TAGLN3 (margin: ABHD10; TAGLN3) | 3.82 | 1.55e-04 | 2.71e-03 |
|  | 1 | 43751270 | 43751563 | PDFSTATSENSEMBLUCSC | 294 | 8 | 1558 | tss | 0.104932 | 0.006292 | C1orf210 (margin: TMEM125; C1orf210) | 3.82 | 1.04e-02 | 3.96e-02 |
|  | 13 | 84456127 | 84456722 | PDFSTATSENSEMBLUCSC | 596 | 9 | 11806 | island | 0.894082 | 0.257688 | SLITRK1 (margin: SLITRK1) | 3.82 | 2.76e-03 | 1.78e-02 |
|  | 4 | 156587884 | 156588387 | PDFSTATSENSEMBLUCSC | 504 | 8 | 30159 | gene;tss;island | 0.383802 | 0.023016 | GUCY1A3 (margin: GUCY1A3) | 3.82 | 1.55e-04 | 2.71e-03 |
|  | 1 | 220101698 | 220102043 | PDFSTATSENSEMBLUCSC | 346 | 8 | 4211 | island | 0.589472 | 0.949138 | SLC30A10 (margin: SLC30A10) | 3.81 | 6.22e-04 | 6.71e-03 |
|  | 1 | 220101698 | 220101962 | PDFSTATSENSEMBLUCSC | 265 | 6 | 1014 | gene | 0.656775 | 0.956432 | SLC30A10 (margin: SLC30A10) | 3.81 | 2.16e-03 | 1.45e-02 |
|  | 6 | 30923241 | 30924004 | PDFSTATSENSEMBLUCSC | 764 | 17 | 35746 | island | 0.493822 | 0.909337 | DPCR1; HCG21 (margin: DPCR1; HCG21) | 3.81 | 9.73e-06 | 4.82e-04 |
|  | 17 | 44896147 | 44896223 | PDFSTATSENSEMBLUCSC | 77 | 6 | 17435 | tss | 0.267941 | 0.039237 | WNT3 (margin: WNT3) | 3.81 | 2.16e-03 | 1.45e-02 |
|  | 11 | 44341361 | 44341831 | PDFSTATSENSEMBLUCSC | 471 | 5 | 8227 | island | 0.771222 | 0.979388 | (margin: ALX4) | 3.81 | 7.94e-03 | 3.19e-02 |
|  | 6 | 32171786 | 32172151 | PDFSTATSENSEMBLUCSC | 366 | 8 | 34088 | gene | 0.173519 | 0.674541 | NOTCH4 (margin: GPSM3; NOTCH4) | 3.80 | 6.22e-04 | 6.71e-03 |
|  | 7 | 121784102 | 121784596 | PDFSTATSENSEMBLUCSC | 495 | 9 | 38868 | island | 0.050096 | 0.493100 | AASS (margin: AASS) | 3.80 | 2.88e-04 | 4.58e-03 |
|  | 1 | 3568669 | 3568722 | PDFSTATSENSEMBLUCSC | 54 | 6 | 1183 | tss;island | 0.356578 | 0.039575 | TP73; WRAP73 (margin: TP73; WRAP73; RP5-1092A11.5) | 3.80 | 2.16e-03 | 1.45e-02 |
|  | 20 | 2781316 | 2781685 | PDFSTATSENSEMBLUCSC | 370 | 8 | 25551 | tss | 0.736049 | 0.975281 | CPXM1 (margin: CPXM1) | 3.80 | 3.11e-04 | 4.77e-03 |
|  | 15 | 29499226 | 29499526 | PDFSTATSENSEMBLUCSC | 301 | 5 | 13270 | gene | 0.694966 | 0.983560 | FAM189A1 (margin: FAM189A1) | 3.80 | 7.94e-03 | 3.19e-02 |
|  | 11 | 30605411 | 30605797 | PDFSTATSENSEMBLUCSC | 387 | 5 | 6551 | gene;island | 0.630225 | 0.929240 | RP5-1024C24.1; MPPED2 (margin: RP5-1024C24.1; MPPED2) | 3.80 | 7.94e-03 | 3.19e-02 |
|  | 2 | 39893078 | 39893520 | PDFSTATSENSEMBLUCSC | 443 | 6 | 22654 | gene;island | 0.782332 | 0.986842 | AC007246.3; TMEM178A (margin: AC007246.3; TMEM178A) | 3.80 | 4.33e-03 | 2.52e-02 |
|  | 6 | 32363084 | 32363325 | PDFSTATSENSEMBLUCSC | 242 | 9 | 34105 | gene | 0.312437 | 0.963162 | HCG23; BTNL2 (margin: HCG23; BTNL2) | 3.79 | 7.77e-03 | 3.19e-02 |
|  | 16 | 2261069 | 2261237 | PDFSTATSENSEMBLUCSC | 169 | 7 | 15021 | tss | 0.603656 | 0.954671 | MLST8; RP11-304L19.8; BRICD5; PGP (margin: MLST8; RP11-304L19.8; AC009065.3; BRICD5; PGP) | 3.79 | 5.83e-04 | 6.47e-03 |
|  | 11 | 78387190 | 78387569 | PDFSTATSENSEMBLUCSC | 380 | 7 | 6846 | gene;island | 0.629494 | 0.964665 | TENM4 (margin: TENM4) | 3.79 | 6.99e-03 | 3.19e-02 |
|  | 15 | 78556834 | 78557094 | PDFSTATSENSEMBLUCSC | 261 | 6 | 13437 | gene;tss;island | 0.079483 | 0.542311 | DNAJA4; RP11-762H8.3 (margin: DNAJA4; RP11-762H8.3) | 3.79 | 2.16e-03 | 1.45e-02 |
|  | 7 | 50132903 | 50133212 | PDFSTATSENSEMBLUCSC | 310 | 8 | 37593 | tss | 0.765775 | 0.967611 | ZPBP (margin: C7orf72; ZPBP) | 3.79 | 2.95e-03 | 1.88e-02 |
|  | 11 | 67141628 | 67142030 | PDFSTATSENSEMBLUCSC | 403 | 8 | 6749 | gene;tss;island | 0.006607 | 0.098417 | AP003419.11; CLCF1 (margin: RN7SKP239; AP003419.11; CLCF1) | 3.79 | 2.95e-03 | 1.88e-02 |
|  | 11 | 85522279 | 85522637 | PDFSTATSENSEMBLUCSC | 359 | 8 | 7605 | tss | 0.520712 | 0.091491 | SYTL2 (margin: SYTL2) | 3.78 | 2.95e-03 | 1.88e-02 |
|  | 15 | 100882020 | 100882647 | PDFSTATSENSEMBLUCSC | 628 | 11 | 14507 | island | 0.230900 | 0.015438 | ADAMTS17 (margin: ADAMTS17; SPATA41) | 3.78 | 5.53e-04 | 6.47e-03 |
|  | 2 | 190445477 | 190446181 | PDFSTATSENSEMBLUCSC | 705 | 7 | 24955 | island | 0.702217 | 0.974688 | SLC40A1 (margin: SLC40A1) | 3.77 | 1.11e-02 | 4.14e-02 |
|  | 2 | 240279976 | 240280294 | PDFSTATSENSEMBLUCSC | 319 | 5 | 23117 | gene | 0.986121 | 0.885821 | HDAC4 (margin: HDAC4; MIR2467) | 3.77 | 7.94e-03 | 3.19e-02 |
|  | 20 | 2361431 | 2361548 | PDFSTATSENSEMBLUCSC | 118 | 5 | 25543 | tss | 0.744947 | 0.985329 | TGM6 (margin: TGM6) | 3.77 | 7.94e-03 | 3.19e-02 |
|  | 8 | 104310606 | 104310886 | PDFSTATSENSEMBLUCSC | 281 | 6 | 39857 | tss | 0.004542 | 0.036168 | FZD6; RP11-318M2.2 (margin: FZD6; RP11-318M2.2) | 3.77 | 2.16e-03 | 1.45e-02 |
|  | 4 | 5053085 | 5053596 | PDFSTATSENSEMBLUCSC | 512 | 7 | 30877 | island | 0.486009 | 0.975514 | STK32B (margin: STK32B) | 3.77 | 1.17e-03 | 1.13e-02 |
|  | 22 | 24180995 | 24181270 | PDFSTATSENSEMBLUCSC | 276 | 6 | 27354 | island | 0.789792 | 0.983213 | DERL3 (margin: SMARCB1; DERL3) | 3.77 | 2.16e-03 | 1.45e-02 |
|  | 10 | 125651370 | 125651726 | PDFSTATSENSEMBLUCSC | 357 | 8 | 6221 | island | 0.634302 | 0.962387 | CPXM2 (margin: CPXM2) | 3.76 | 6.99e-03 | 3.19e-02 |
|  | 10 | 61469748 | 61469980 | PDFSTATSENSEMBLUCSC | 233 | 8 | 5094 | tss | 0.596866 | 0.040963 | SLC16A9 (margin: SLC16A9) | 3.76 | 6.99e-03 | 3.19e-02 |
|  | 10 | 14051636 | 14052028 | PDFSTATSENSEMBLUCSC | 393 | 6 | 4508 | gene | 0.603613 | 0.965023 | FRMD4A (margin: RP11-142M10.2; FRMD4A) | 3.76 | 4.33e-03 | 2.52e-02 |
|  | 19 | 2588241 | 2588629 | PDFSTATSENSEMBLUCSC | 389 | 5 | 19695 | gene;island | 0.882797 | 0.403795 | GNG7 (margin: GNG7) | 3.75 | 7.94e-03 | 3.19e-02 |
|  | 11 | 85521839 | 85522637 | PDFSTATSENSEMBLUCSC | 799 | 12 | 8707 | island | 0.258890 | 0.044898 | SYTL2 (margin: SYTL2) | 3.75 | 2.74e-04 | 4.46e-03 |
|  | 6 | 30418494 | 30419612 | PDFSTATSENSEMBLUCSC | 1119 | 32 | 35680 | island | 0.463818 | 0.912848 | (margin: ) | 3.74 | 3.51e-10 | 1.71e-07 |
|  | 11 | 31832710 | 31833264 | PDFSTATSENSEMBLUCSC | 555 | 11 | 6562 | gene;island | 0.752034 | 0.973280 | RCN1; PAX6 (margin: RCN1; PAX6) | 3.74 | 2.84e-06 | 1.96e-04 |
|  | 7 | 27143046 | 27143806 | PDFSTATSENSEMBLUCSC | 761 | 12 | 37488 | tss;island | 0.959201 | 0.612951 | HOXA2; HOXA3 (margin: HOTAIRM1; HOXA-AS2; HOXA1; HOXA2; HOXA3) | 3.74 | 1.03e-04 | 2.47e-03 |
|  | 12 | 114846162 | 114846503 | PDFSTATSENSEMBLUCSC | 342 | 8 | 10841 | island | 0.585134 | 0.942764 | TBX5-AS1; TBX5 (margin: TBX5-AS1; TBX5) | 3.74 | 6.22e-04 | 6.71e-03 |
|  | 5 | 77268331 | 77268888 | PDFSTATSENSEMBLUCSC | 558 | 5 | 32907 | island | 0.821990 | 0.984573 | (margin: ) | 3.73 | 7.94e-03 | 3.19e-02 |
|  | 2 | 175199259 | 175199803 | PDFSTATSENSEMBLUCSC | 545 | 11 | 23723 | tss | 0.603495 | 0.975612 | SP9; AC018470.1 (margin: AC018470.4; SP9; AC018470.1) | 3.73 | 2.75e-04 | 4.46e-03 |
|  | 10 | 115938729 | 115939024 | PDFSTATSENSEMBLUCSC | 296 | 6 | 5375 | tss | 0.957167 | 0.641491 | TDRD1 (margin: TDRD1; C10orf118; MIR2110) | 3.73 | 8.66e-03 | 3.36e-02 |
|  | 15 | 45670865 | 45671347 | PDFSTATSENSEMBLUCSC | 483 | 13 | 13333 | gene;tss;island | 0.869863 | 0.986491 | GATM (margin: GATM) | 3.73 | 2.31e-06 | 1.96e-04 |
|  | 20 | 42875645 | 42875779 | PDFSTATSENSEMBLUCSC | 135 | 6 | 25756 | tss | 0.375155 | 0.880633 | GDAP1L1 (margin: GDAP1L1) | 3.73 | 2.16e-03 | 1.45e-02 |
|  | 10 | 91295317 | 91295421 | PDFSTATSENSEMBLUCSC | 105 | 6 | 5215 | tss | 0.832077 | 0.987465 | SLC16A12 (margin: SLC16A12) | 3.73 | 2.16e-03 | 1.45e-02 |
|  | 19 | 11998457 | 11998660 | PDFSTATSENSEMBLUCSC | 204 | 7 | 20484 | tss | 0.407806 | 0.868448 | ZNF69 (margin: ZNF439; ZNF69) | 3.72 | 5.83e-04 | 6.47e-03 |
|  | 6 | 39693159 | 39693479 | PDFSTATSENSEMBLUCSC | 321 | 7 | 36110 | island | 0.140376 | 0.012292 | KIF6 (margin: KIF6) | 3.72 | 4.08e-03 | 2.47e-02 |
|  | 4 | 122301573 | 122302007 | PDFSTATSENSEMBLUCSC | 435 | 6 | 30112 | gene;island | 0.646415 | 0.967748 | QRFPR (margin: QRFPR; RP11-364P2.2) | 3.72 | 4.33e-03 | 2.52e-02 |
|  | 5 | 63461216 | 63461654 | PDFSTATSENSEMBLUCSC | 439 | 8 | 32088 | tss | 0.665439 | 0.097552 | RNF180 (margin: RNF180) | 3.72 | 2.95e-03 | 1.88e-02 |
|  | 7 | 50132541 | 50133212 | PDFSTATSENSEMBLUCSC | 672 | 12 | 38489 | island | 0.765775 | 0.967611 | ZPBP (margin: C7orf72; ZPBP) | 3.72 | 1.83e-03 | 1.45e-02 |
|  | 10 | 105212236 | 105212813 | PDFSTATSENSEMBLUCSC | 578 | 11 | 5338 | tss | 0.609000 | 0.347970 | RP11-225H22.4; CALHM2; CALHM1 (margin: PDCD11; RP11-225H22.4; CALHM2; CALHM1) | 3.72 | 3.94e-04 | 5.86e-03 |
|  | 3 | 27754391 | 27754675 | PDFSTATSENSEMBLUCSC | 285 | 5 | 28947 | island | 0.355507 | 0.878219 | RP11-222K16.2 (margin: RP11-222K16.2; AC098614.3; EOMES) | 3.72 | 7.94e-03 | 3.19e-02 |
|  | 10 | 50887472 | 50887934 | PDFSTATSENSEMBLUCSC | 463 | 10 | 5738 | island | 0.497170 | 0.946012 | CHAT; C10orf53 (margin: CHAT; C10orf53) | 3.71 | 3.89e-03 | 2.43e-02 |
|  | 7 | 150778201 | 150778340 | PDFSTATSENSEMBLUCSC | 140 | 5 | 37286 | gene | 0.025940 | 0.356509 | FASTK; TMUB1 (margin: SLC4A2; AGAP3; RP11-148K1.12; FASTK; TMUB1) | 3.71 | 7.94e-03 | 3.19e-02 |
|  | 10 | 131761150 | 131761386 | PDFSTATSENSEMBLUCSC | 237 | 6 | 4838 | gene;island | 0.679958 | 0.962685 | EBF3 (margin: EBF3) | 3.71 | 2.16e-03 | 1.45e-02 |
|  | 12 | 322514 | 322983 | PDFSTATSENSEMBLUCSC | 470 | 10 | 8991 | gene;tss | 0.337324 | 0.869478 | SLC6A12 (margin: RP11-283I3.2; SLC6A12; SLC6A13) | 3.71 | 4.87e-04 | 6.47e-03 |
|  | 8 | 65492528 | 65492936 | PDFSTATSENSEMBLUCSC | 409 | 10 | 40347 | island | 0.175875 | 0.738905 | BHLHE22; RP11-21C4.1 (margin: BHLHE22; RP11-21C4.5; RP11-21C4.1; CYP7B1) | 3.71 | 3.25e-04 | 4.91e-03 |
|  | 8 | 65492528 | 65492792 | PDFSTATSENSEMBLUCSC | 265 | 8 | 39736 | tss | 0.164728 | 0.738905 | BHLHE22; RP11-21C4.1 (margin: BHLHE22; RP11-21C4.5; RP11-21C4.1; CYP7B1) | 3.71 | 6.22e-04 | 6.71e-03 |
|  | 20 | 61162000 | 61162216 | PDFSTATSENSEMBLUCSC | 217 | 7 | 25491 | gene | 0.341657 | 0.968412 | C20orf166; MIR133A2 (margin: C20orf166; MIR1-1; MIR133A2; RPL7P3; WI2-87327B8.1) | 3.71 | 6.99e-03 | 3.19e-02 |
|  | 22 | 19137874 | 19138267 | PDFSTATSENSEMBLUCSC | 394 | 5 | 26955 | tss;island | 0.788035 | 0.978832 | GSC2 (margin: DGCR14; GSC2) | 3.70 | 7.94e-03 | 3.19e-02 |
|  | 7 | 105221826 | 105222180 | PDFSTATSENSEMBLUCSC | 355 | 8 | 38821 | island | 0.921773 | 0.471501 | EFCAB10; YBX1P2 (margin: EFCAB10; YBX1P2) | 3.70 | 1.04e-02 | 3.96e-02 |
|  | 11 | 46354562 | 46355283 | PDFSTATSENSEMBLUCSC | 722 | 10 | 8246 | island | 0.771264 | 0.984161 | DGKZ (margin: CREB3L1; DGKZ) | 3.70 | 1.50e-03 | 1.41e-02 |
|  | 13 | 33002010 | 33002269 | PDFSTATSENSEMBLUCSC | 260 | 5 | 11124 | gene | 0.130875 | 0.011906 | N4BP2L1; RP11-298P3.4 (margin: N4BP2L1; SNORA16; RP11-298P3.4; N4BP2L2) | 3.70 | 7.94e-03 | 3.19e-02 |
|  | 17 | 33700745 | 33700817 | PDFSTATSENSEMBLUCSC | 73 | 6 | 17267 | tss;island | 0.107058 | 0.530248 | SLFN11 (margin: SLFN11; RP11-686D22.6) | 3.70 | 2.16e-03 | 1.45e-02 |
|  | 16 | 57318603 | 57319093 | PDFSTATSENSEMBLUCSC | 491 | 11 | 15260 | tss;island | 0.008169 | 0.066675 | PLLP (margin: PLLP) | 3.69 | 5.24e-03 | 2.98e-02 |
|  | 1 | 3056542 | 3056951 | PDFSTATSENSEMBLUCSC | 410 | 7 | 102 | gene;island | 0.133823 | 0.732067 | PRDM16 (margin: PRDM16; RP1-163G9.2) | 3.69 | 1.17e-03 | 1.13e-02 |
|  | 20 | 2781122 | 2781685 | PDFSTATSENSEMBLUCSC | 564 | 11 | 25954 | island | 0.740995 | 0.976379 | CPXM1 (margin: CPXM1) | 3.69 | 8.51e-05 | 2.07e-03 |
|  | 13 | 112721712 | 112721789 | PDFSTATSENSEMBLUCSC | 78 | 6 | 11543 | tss | 0.634855 | 0.949551 | SOX1 (margin: SOX1) | 3.68 | 2.16e-03 | 1.45e-02 |
|  | 10 | 61469599 | 61469980 | PDFSTATSENSEMBLUCSC | 382 | 9 | 5764 | island | 0.593549 | 0.049942 | SLC16A9 (margin: SLC16A9) | 3.68 | 5.64e-03 | 3.18e-02 |
|  | 4 | 42400738 | 42401242 | PDFSTATSENSEMBLUCSC | 505 | 6 | 29994 | gene;island | 0.384687 | 0.893546 | SHISA3 (margin: SHISA3; RP11-63A11.1; ATP8A1) | 3.68 | 8.66e-03 | 3.36e-02 |
|  | 10 | 102821427 | 102822508 | PDFSTATSENSEMBLUCSC | 1082 | 11 | 4722 | gene;island | 0.356010 | 0.040526 | KAZALD1 (margin: KAZALD1) | 3.68 | 7.65e-04 | 8.13e-03 |
|  | 8 | 54164051 | 54164442 | PDFSTATSENSEMBLUCSC | 392 | 8 | 40296 | island | 0.712925 | 0.971749 | OPRK1 (margin: RP11-162D9.3; OPRK1) | 3.67 | 2.95e-03 | 1.88e-02 |
|  | 12 | 122356316 | 122356852 | PDFSTATSENSEMBLUCSC | 537 | 6 | 10925 | island | 0.958229 | 0.637917 | PSMD9; RP11-87C12.2; WDR66 (margin: PSMD9; RP11-87C12.2; RNU7-170P; WDR66) | 3.67 | 2.16e-03 | 1.45e-02 |
|  | 7 | 1022471 | 1023156 | PDFSTATSENSEMBLUCSC | 686 | 11 | 38063 | island | 0.527375 | 0.955195 | CYP2W1 (margin: CYP2W1; COX19) | 3.67 | 8.51e-05 | 2.07e-03 |
|  | 11 | 69519128 | 69519437 | PDFSTATSENSEMBLUCSC | 310 | 7 | 7521 | tss | 0.330090 | 0.691431 | FGF19 (margin: FGF19) | 3.67 | 2.33e-03 | 1.52e-02 |
|  | 2 | 177053274 | 177053568 | PDFSTATSENSEMBLUCSC | 295 | 10 | 24915 | island | 0.074999 | 0.494011 | HOXD1; HOXD-AS1 (margin: AC009336.24; HOXD1; HOXD-AS1) | 3.67 | 1.08e-05 | 4.82e-04 |
|  | 11 | 67418140 | 67418405 | PDFSTATSENSEMBLUCSC | 266 | 9 | 7500 | tss | 0.073856 | 0.666909 | ACY3; AP003385.2 (margin: TBX10; ACY3; AP003385.2; ALDH3B2) | 3.66 | 7.82e-04 | 8.17e-03 |
|  | 3 | 125899370 | 125900108 | PDFSTATSENSEMBLUCSC | 739 | 13 | 29427 | island | 0.771005 | 0.978300 | ALDH1L1-AS2; ALDH1L1 (margin: ALDH1L1-AS2; ALDH1L1) | 3.66 | 2.67e-05 | 1.07e-03 |
|  | 3 | 125899547 | 125900108 | PDFSTATSENSEMBLUCSC | 562 | 11 | 28556 | tss | 0.771005 | 0.978300 | ALDH1L1-AS2; ALDH1L1 (margin: ALDH1L1-AS2; ALDH1L1) | 3.66 | 5.39e-05 | 1.47e-03 |
|  | 6 | 42927940 | 42928497 | PDFSTATSENSEMBLUCSC | 558 | 22 | 34985 | tss | 0.253324 | 0.015977 | GNMT (margin: RP3-475N16.1; GNMT; RPL24P4; PEX6) | 3.66 | 2.35e-07 | 3.02e-05 |
|  | 10 | 134043618 | 134043897 | PDFSTATSENSEMBLUCSC | 280 | 5 | 4858 | gene;island | 0.658959 | 0.953866 | STK32C (margin: STK32C) | 3.65 | 7.94e-03 | 3.19e-02 |
|  | 16 | 15595854 | 15596082 | PDFSTATSENSEMBLUCSC | 229 | 6 | 15107 | tss | 0.436238 | 0.946450 | RP11-1021N1.1; C16orf45 (margin: RP11-1021N1.1; C16orf45) | 3.65 | 8.66e-03 | 3.36e-02 |
|  | 11 | 31832879 | 31833264 | PDFSTATSENSEMBLUCSC | 386 | 8 | 7223 | tss | 0.755876 | 0.974795 | RCN1; PAX6 (margin: RCN1; PAX6) | 3.65 | 1.55e-04 | 2.71e-03 |
|  | 3 | 196366191 | 196366618 | PDFSTATSENSEMBLUCSC | 428 | 7 | 28816 | tss | 0.613406 | 0.909359 | NRROS; PIGX (margin: NRROS; PIGX; AC023797.1) | 3.64 | 6.99e-03 | 3.19e-02 |
|  | 14 | 77737495 | 77737800 | PDFSTATSENSEMBLUCSC | 306 | 6 | 13032 | island | 0.667932 | 0.965155 | NGB (margin: TMEM63C; MIR1260A; NGB; POMT2) | 3.64 | 2.16e-03 | 1.45e-02 |
|  | 8 | 24771114 | 24771645 | PDFSTATSENSEMBLUCSC | 532 | 7 | 39604 | tss;island | 0.365215 | 0.869247 | NEFM; RP11-624C23.1; GS1-72M22.1 (margin: NEFM; RP11-624C23.1; GS1-72M22.1) | 3.64 | 1.17e-03 | 1.13e-02 |
|  | 15 | 83240578 | 83240791 | PDFSTATSENSEMBLUCSC | 214 | 6 | 13857 | tss | 0.495213 | 0.875009 | CPEB1 (margin: RP11-379H8.1; CPEB1) | 3.63 | 2.16e-03 | 1.45e-02 |
|  | 1 | 50889182 | 50889510 | PDFSTATSENSEMBLUCSC | 329 | 6 | 1616 | tss | 0.678095 | 0.976904 | DMRTA2 (margin: DMRTA2) | 3.63 | 4.33e-03 | 2.52e-02 |
|  | 10 | 91295045 | 91295421 | PDFSTATSENSEMBLUCSC | 377 | 9 | 5924 | island | 0.815173 | 0.986377 | SLC16A12 (margin: SLC16A12) | 3.63 | 4.11e-05 | 1.17e-03 |
|  | 14 | 104690040 | 104690244 | PDFSTATSENSEMBLUCSC | 205 | 5 | 13204 | island | 0.488380 | 0.924957 | RP11-260M19.2 (margin: RP11-260M19.2) | 3.63 | 7.94e-03 | 3.19e-02 |
|  | 2 | 65215361 | 65215753 | PDFSTATSENSEMBLUCSC | 393 | 10 | 23393 | tss;island | 0.051784 | 0.430556 | SLC1A4 (margin: SLC1A4; RNU6-548P) | 3.63 | 1.08e-05 | 4.82e-04 |
|  | 10 | 127464165 | 127464762 | PDFSTATSENSEMBLUCSC | 598 | 9 | 6238 | island | 0.575454 | 0.897565 | MMP21 (margin: C10orf137; MMP21) | 3.62 | 5.64e-03 | 3.18e-02 |
|  | 6 | 29523592 | 29524117 | PDFSTATSENSEMBLUCSC | 526 | 8 | 33660 | gene;island | 0.206994 | 0.680344 | OR2I1P; UBD; GABBR1 (margin: OR2I1P; UBD; GABBR1) | 3.62 | 4.66e-03 | 2.68e-02 |
|  | 5 | 176170138 | 176170795 | PDFSTATSENSEMBLUCSC | 658 | 7 | 33433 | island | 0.615944 | 0.911526 | RP11-375B1.3 (margin: RP11-375B1.3; RP11-375B1.2) | 3.62 | 1.11e-02 | 4.14e-02 |
|  | 1 | 1266468 | 1266781 | PDFSTATSENSEMBLUCSC | 314 | 6 | 2426 | island | 0.348044 | 0.807535 | TAS1R3 (margin: GLTPD1; TAS1R3; CPSF3L; DVL1) | 3.62 | 2.16e-03 | 1.45e-02 |
|  | 1 | 50889128 | 50889510 | PDFSTATSENSEMBLUCSC | 383 | 7 | 3253 | island | 0.678901 | 0.977000 | DMRTA2 (margin: DMRTA2) | 3.62 | 1.17e-03 | 1.13e-02 |
|  | 10 | 133951618 | 133951963 | PDFSTATSENSEMBLUCSC | 346 | 5 | 4853 | gene;island | 0.616284 | 0.972076 | JAKMIP3 (margin: JAKMIP3) | 3.61 | 7.94e-03 | 3.19e-02 |
|  | 3 | 192126584 | 192126996 | PDFSTATSENSEMBLUCSC | 413 | 7 | 28085 | gene;island | 0.662388 | 0.956559 | FGF12 (margin: FGF12) | 3.61 | 4.08e-03 | 2.47e-02 |
|  | 19 | 6463949 | 6464214 | PDFSTATSENSEMBLUCSC | 266 | 9 | 20390 | tss | 0.315131 | 0.015143 | CRB3; SLC25A23 (margin: CRB3; SLC25A23; DENND1C) | 3.61 | 7.77e-03 | 3.19e-02 |
|  | 7 | 1748302 | 1748766 | PDFSTATSENSEMBLUCSC | 465 | 9 | 37405 | tss | 0.449636 | 0.920668 | ELFN1 (margin: ELFN1) | 3.60 | 1.65e-04 | 2.80e-03 |
|  | 10 | 135050619 | 135051581 | PDFSTATSENSEMBLUCSC | 963 | 28 | 6348 | island | 0.550924 | 0.956534 | VENTX (margin: KNDC1; UTF1; VENTX; MIR202) | 3.60 | 1.65e-09 | 6.41e-07 |
|  | 11 | 1855746 | 1856307 | PDFSTATSENSEMBLUCSC | 562 | 7 | 6432 | gene | 0.351682 | 0.781362 | SYT8 (margin: SYT8; TNNI2) | 3.60 | 6.99e-03 | 3.19e-02 |
|  | 2 | 177053274 | 177053297 | PDFSTATSENSEMBLUCSC | 24 | 6 | 23744 | tss | 0.074999 | 0.479642 | HOXD1; HOXD-AS1 (margin: AC009336.24; HOXD1; HOXD-AS1) | 3.60 | 2.16e-03 | 1.45e-02 |
|  | 12 | 54343506 | 54343835 | PDFSTATSENSEMBLUCSC | 330 | 5 | 10480 | island | 0.906257 | 0.433785 | (margin: HOXC13; HOXC12; AC012531.23; HOXC-AS5) | 3.60 | 7.94e-03 | 3.19e-02 |
|  | 14 | 101489985 | 101490253 | PDFSTATSENSEMBLUCSC | 269 | 10 | 12620 | tss | 0.434178 | 0.897905 | MIR379; MIR411; MIR299; MIR1197; MIR323A; MIR380 (margin: MIR379; MIR411; MIR299; MIR1197; MIR323A; MIR758; MIR329-1; MIR329-2; MIR494; MIR1193; MIR543; MIR495; MIR380) | 3.60 | 3.89e-03 | 2.43e-02 |
|  | 5 | 178487123 | 178487576 | PDFSTATSENSEMBLUCSC | 454 | 10 | 32548 | tss | 0.081366 | 0.485388 | ZNF354C (margin: ZNF354C) | 3.60 | 1.08e-05 | 4.82e-04 |
|  | 7 | 150778724 | 150779390 | PDFSTATSENSEMBLUCSC | 667 | 14 | 37287 | gene;tss;island | 0.156903 | 0.620475 | FASTK; TMUB1 (margin: SLC4A2; AGAP3; RP11-148K1.12; FASTK; TMUB1) | 3.60 | 9.71e-04 | 1.01e-02 |
|  | 2 | 43019819 | 43020457 | PDFSTATSENSEMBLUCSC | 639 | 8 | 23322 | tss | 0.660483 | 0.966472 | HAAO (margin: HAAO; FTOP1) | 3.59 | 3.11e-04 | 4.77e-03 |
|  | 17 | 44896017 | 44896223 | PDFSTATSENSEMBLUCSC | 207 | 8 | 18447 | island | 0.333662 | 0.051288 | WNT3 (margin: WNT3) | 3.58 | 1.55e-04 | 2.71e-03 |
|  | 5 | 169724590 | 169724803 | PDFSTATSENSEMBLUCSC | 214 | 5 | 31889 | gene | 0.384242 | 0.895692 | LCP2 (margin: LCP2; CTB-114C7.4) | 3.58 | 7.94e-03 | 3.19e-02 |
|  | 11 | 7695165 | 7695528 | PDFSTATSENSEMBLUCSC | 364 | 7 | 7121 | tss;island | 0.527611 | 0.957813 | CYB5R2 (margin: CYB5R2) | 3.58 | 1.17e-03 | 1.13e-02 |
|  | 7 | 29603121 | 29603541 | PDFSTATSENSEMBLUCSC | 421 | 8 | 38376 | island | 0.095105 | 0.006532 | PRR15; AC007255.8 (margin: RP11-550A18.1; PRR15; AC007255.8) | 3.57 | 1.04e-02 | 3.96e-02 |
|  | 19 | 49993125 | 49993217 | PDFSTATSENSEMBLUCSC | 93 | 6 | 20154 | gene;tss;island | 0.115124 | 0.607747 | RPL13A; SNORD32A; SNORD33; SNORD34; SNORD35A (margin: CTD-3148I10.9; FLT3LG; RPL13A; SNORD32A; SNORD33; SNORD34; SNORD35A; RPS11; SNORD35B; hsa-mir-150; MIR150) | 3.57 | 4.33e-03 | 2.52e-02 |
|  | 21 | 37914924 | 37915391 | PDFSTATSENSEMBLUCSC | 468 | 7 | 26418 | gene;tss | 0.646491 | 0.977323 | CLDN14 (margin: AP000695.4; CLDN14) | 3.57 | 2.33e-03 | 1.52e-02 |
|  | 10 | 95361132 | 95361386 | PDFSTATSENSEMBLUCSC | 255 | 5 | 5239 | tss;island | 0.710132 | 0.961811 | FFAR4; RBP4 (margin: FFAR4; AL356214.1; PDE6C; RBP4) | 3.57 | 7.94e-03 | 3.19e-02 |
|  | 5 | 37834672 | 37835168 | PDFSTATSENSEMBLUCSC | 497 | 7 | 31590 | gene;island | 0.809852 | 0.975053 | GDNF (margin: GDNF-AS1; GDNF) | 3.57 | 1.11e-02 | 4.14e-02 |
|  | 16 | 66878426 | 66878810 | PDFSTATSENSEMBLUCSC | 385 | 9 | 15286 | tss | 0.840890 | 0.981504 | CA7; NAE1; RP11-61A14.1 (margin: CA7; NAE1; RP11-61A14.1) | 3.57 | 4.11e-05 | 1.17e-03 |
|  | 16 | 66878426 | 66879016 | PDFSTATSENSEMBLUCSC | 591 | 11 | 14783 | gene;island | 0.813533 | 0.979166 | CA7; NAE1; RP11-61A14.1 (margin: CA7; NAE1; RP11-61A14.1) | 3.57 | 2.75e-04 | 4.46e-03 |
|  | 14 | 56232650 | 56233178 | PDFSTATSENSEMBLUCSC | 529 | 7 | 12871 | island | 0.576525 | 0.935591 | RP11-813I20.2; RPL13AP3 (margin: RP11-813I20.2; RPL13AP3) | 3.56 | 4.08e-03 | 2.47e-02 |
|  | 7 | 35077598 | 35077807 | PDFSTATSENSEMBLUCSC | 210 | 9 | 38405 | island | 0.134038 | 0.006068 | DPY19L1 (margin: DPY19L1) | 3.56 | 7.77e-03 | 3.19e-02 |
|  | 6 | 100054469 | 100054615 | PDFSTATSENSEMBLUCSC | 147 | 6 | 35137 | tss | 0.811298 | 0.985431 | PRDM13 (margin: PRDM13) | 3.56 | 2.16e-03 | 1.45e-02 |
|  | 4 | 110223040 | 110223795 | PDFSTATSENSEMBLUCSC | 756 | 10 | 30089 | gene | 0.661326 | 0.962103 | AC004051.2; COL25A1 (margin: AC004051.2; COL25A1) | 3.56 | 5.20e-03 | 2.96e-02 |
|  | 1 | 210856989 | 210857380 | PDFSTATSENSEMBLUCSC | 392 | 5 | 992 | gene;island | 0.614488 | 0.949080 | KCNH1 (margin: HHAT; KCNH1) | 3.55 | 7.94e-03 | 3.19e-02 |
|  | 19 | 840737 | 841082 | PDFSTATSENSEMBLUCSC | 346 | 6 | 21178 | island | 0.314497 | 0.832261 | PRTN3 (margin: AZU1; PRTN3; ELANE) | 3.55 | 8.66e-03 | 3.36e-02 |
|  | 12 | 2788627 | 2788847 | PDFSTATSENSEMBLUCSC | 221 | 5 | 9005 | gene;island | 0.789263 | 0.984477 | CACNA1C; CACNA1C-AS1 (margin: CACNA1C; CACNA1C-AS2; CACNA1C-AS1) | 3.55 | 7.94e-03 | 3.19e-02 |
|  | 3 | 131080235 | 131080927 | PDFSTATSENSEMBLUCSC | 693 | 9 | 29479 | island | 0.459429 | 0.915605 | NUDT16P; RP11-933H2.4 (margin: NEK11; NUDT16P; RP11-933H2.4) | 3.54 | 1.65e-04 | 2.80e-03 |
|  | 3 | 131080235 | 131080697 | PDFSTATSENSEMBLUCSC | 463 | 7 | 28593 | tss | 0.456731 | 0.888109 | NUDT16P; RP11-933H2.4 (margin: NEK11; NUDT16P; RP11-933H2.4) | 3.54 | 1.17e-03 | 1.13e-02 |
|  | 3 | 136537607 | 136537856 | PDFSTATSENSEMBLUCSC | 250 | 8 | 28614 | tss | 0.735935 | 0.964507 | SLC35G2 (margin: SLC35G2; RP11-731C17.1) | 3.54 | 1.55e-04 | 2.71e-03 |
|  | 1 | 3663164 | 3664084 | PDFSTATSENSEMBLUCSC | 921 | 10 | 2570 | island | 0.629121 | 0.958372 | TP73-AS1 (margin: TP73; CCDC27; TP73-AS1) | 3.54 | 1.50e-03 | 1.41e-02 |
|  | 17 | 7165831 | 7165909 | PDFSTATSENSEMBLUCSC | 79 | 6 | 17069 | tss;island | 0.077430 | 0.009160 | RP1-4G17.5; CLDN7 (margin: ELP5; CTDNEP1; RP1-4G17.5; CLDN7; Y\_RNA) | 3.54 | 2.16e-03 | 1.45e-02 |
|  | 7 | 1248693 | 1249199 | PDFSTATSENSEMBLUCSC | 507 | 7 | 38088 | island | 0.591919 | 0.979996 | (margin: ) | 3.54 | 1.17e-03 | 1.13e-02 |
|  | 5 | 537349 | 537906 | PDFSTATSENSEMBLUCSC | 558 | 7 | 32589 | island | 0.592687 | 0.946129 | MIR4456 (margin: RP11-310P5.2; MIR4456) | 3.54 | 6.99e-03 | 3.19e-02 |
|  | 11 | 110583543 | 110583662 | PDFSTATSENSEMBLUCSC | 120 | 6 | 7679 | tss | 0.454362 | 0.903884 | ARHGAP20 (margin: ARHGAP20) | 3.54 | 4.33e-03 | 2.52e-02 |
|  | 1 | 91191583 | 91192162 | PDFSTATSENSEMBLUCSC | 580 | 6 | 3463 | island | 0.688544 | 0.976154 | (margin: BARHL2) | 3.53 | 4.33e-03 | 2.52e-02 |
|  | 6 | 32974843 | 32975360 | PDFSTATSENSEMBLUCSC | 518 | 13 | 34176 | gene;island | 0.181833 | 0.776744 | HLA-DOA (margin: HLA-DOA) | 3.53 | 7.95e-04 | 8.28e-03 |
|  | 10 | 44500693 | 44501318 | PDFSTATSENSEMBLUCSC | 626 | 6 | 5704 | island | 0.643482 | 0.960450 | (margin: ) | 3.53 | 8.66e-03 | 3.36e-02 |
|  | 5 | 178017571 | 178018186 | PDFSTATSENSEMBLUCSC | 616 | 10 | 32540 | tss | 0.713695 | 0.951440 | COL23A1 (margin: COL23A1; CLK4) | 3.52 | 7.25e-04 | 7.74e-03 |
|  | 2 | 74741573 | 74741941 | PDFSTATSENSEMBLUCSC | 369 | 5 | 24443 | island | 0.366787 | 0.919492 | TLX2 (margin: LBX2-AS1; TLX2; LBX2; PCGF1; DQX1; AUP1) | 3.52 | 7.94e-03 | 3.19e-02 |
|  | 12 | 48398320 | 48398730 | PDFSTATSENSEMBLUCSC | 411 | 7 | 9665 | tss;island | 0.663298 | 0.959711 | COL2A1; RP1-228P16.3 (margin: RP1-228P16.4; COL2A1; RP1-228P16.3) | 3.52 | 4.08e-03 | 2.47e-02 |
|  | 4 | 42399699 | 42399851 | PDFSTATSENSEMBLUCSC | 153 | 6 | 30367 | tss | 0.821382 | 0.983794 | SHISA3 (margin: SHISA3; RP11-63A11.1; ATP8A1) | 3.52 | 2.16e-03 | 1.45e-02 |
|  | 2 | 43019484 | 43020457 | PDFSTATSENSEMBLUCSC | 974 | 11 | 24245 | island | 0.641145 | 0.959661 | HAAO (margin: HAAO; FTOP1) | 3.51 | 1.28e-04 | 2.71e-03 |
|  | 10 | 134599783 | 134600998 | PDFSTATSENSEMBLUCSC | 1216 | 30 | 5450 | tss;island | 0.849043 | 0.979283 | RP11-288G11.3; NKX6-2 (margin: INPP5A; RP11-288G11.3; NKX6-2) | 3.51 | 1.09e-10 | 7.05e-08 |
|  | 19 | 12075601 | 12076348 | PDFSTATSENSEMBLUCSC | 748 | 11 | 21588 | island | 0.497687 | 0.840142 | ZNF763 (margin: ZNF763; CTD-2006C1.12) | 3.51 | 5.53e-04 | 6.47e-03 |
|  | 12 | 49582874 | 49583112 | PDFSTATSENSEMBLUCSC | 239 | 7 | 9684 | tss | 0.091980 | 0.008150 | TUBA1C; TUBA1A (margin: TUBA1C; TUBA1A) | 3.50 | 1.17e-03 | 1.13e-02 |
|  | 6 | 30068471 | 30069319 | PDFSTATSENSEMBLUCSC | 849 | 18 | 35659 | island | 0.005996 | 0.055257 | TRIM31 (margin: TRIM31-AS1; AL669914.1; TRIM31) | 3.50 | 1.13e-04 | 2.70e-03 |
|  | 1 | 202830475 | 202830826 | PDFSTATSENSEMBLUCSC | 352 | 8 | 2182 | tss;island | 0.154165 | 0.012097 | RP11-480I12.7; RP11-480I12.5 (margin: RP11-480I12.7; RP11-480I12.9; RP11-480I12.5) | 3.50 | 1.55e-04 | 2.71e-03 |
|  | 17 | 7791882 | 7792134 | PDFSTATSENSEMBLUCSC | 253 | 5 | 17102 | tss | 0.244385 | 0.033380 | CHD3 (margin: CHD3; LSMD1) | 3.50 | 7.94e-03 | 3.19e-02 |
|  | 11 | 66314211 | 66314378 | PDFSTATSENSEMBLUCSC | 168 | 5 | 7473 | tss;island | 0.125978 | 0.635371 | ACTN3; ZDHHC24 (margin: ACTN3; ZDHHC24; CTD-3074O7.12; CTD-3074O7.2) | 3.50 | 7.94e-03 | 3.19e-02 |
|  | 6 | 33053576 | 33054001 | PDFSTATSENSEMBLUCSC | 426 | 6 | 34187 | gene | 0.209625 | 0.603471 | HLA-DPB1 (margin: HLA-DPB1; RPL32P1; HLA-DPA1; HLA-DPA2) | 3.50 | 4.33e-03 | 2.52e-02 |
|  | 12 | 110211321 | 110211413 | PDFSTATSENSEMBLUCSC | 93 | 5 | 9995 | tss | 0.329349 | 0.912927 | FAM222A-AS1 (margin: FAM222A; FAM222A-AS1; TRPV4) | 3.50 | 7.94e-03 | 3.19e-02 |
|  | 7 | 158886098 | 158886532 | PDFSTATSENSEMBLUCSC | 435 | 6 | 37384 | gene;island | 0.611297 | 0.957890 | VIPR2 (margin: VIPR2) | 3.50 | 4.33e-03 | 2.52e-02 |
|  | 6 | 32028831 | 32029216 | PDFSTATSENSEMBLUCSC | 386 | 8 | 34025 | gene | 0.434914 | 0.924147 | TNXB (margin: TNXB) | 3.49 | 3.11e-04 | 4.77e-03 |
|  | 19 | 23941207 | 23942126 | PDFSTATSENSEMBLUCSC | 920 | 13 | 21848 | island | 0.409618 | 0.075129 | ZNF681 (margin: RP11-255H23.2; RPSAP58; ZNF681) | 3.49 | 1.20e-02 | 4.46e-02 |
|  | 8 | 11561283 | 11561620 | PDFSTATSENSEMBLUCSC | 338 | 9 | 39553 | tss | 0.759037 | 0.956133 | GATA4 (margin: GATA4) | 3.49 | 8.23e-05 | 2.06e-03 |
|  | 8 | 11561283 | 11561728 | PDFSTATSENSEMBLUCSC | 446 | 11 | 40087 | island | 0.781308 | 0.973173 | GATA4 (margin: GATA4) | 3.49 | 8.51e-05 | 2.07e-03 |
|  | 12 | 6419409 | 6419750 | PDFSTATSENSEMBLUCSC | 342 | 6 | 9506 | tss;island | 0.946476 | 0.545470 | PLEKHG6 (margin: PLEKHG6) | 3.49 | 2.16e-03 | 1.45e-02 |
|  | 4 | 74734714 | 74735101 | PDFSTATSENSEMBLUCSC | 388 | 9 | 30430 | tss | 0.170937 | 0.433298 | CXCL1 (margin: CXCL1) | 3.48 | 3.99e-03 | 2.47e-02 |
|  | 19 | 11689488 | 11689726 | PDFSTATSENSEMBLUCSC | 239 | 6 | 19846 | gene | 0.825926 | 0.981594 | ZNF627; ACP5 (margin: ZNF627; AC020947.2; ACP5) | 3.48 | 8.66e-03 | 3.36e-02 |
|  | 15 | 40544855 | 40545511 | PDFSTATSENSEMBLUCSC | 657 | 11 | 13290 | gene;island | 0.794845 | 0.979413 | RP11-133K1.2; PAK6; C15orf56 (margin: RP11-133K1.2; PAK6; C15orf56) | 3.48 | 1.13e-05 | 5.02e-04 |
|  | 20 | 13200929 | 13200992 | PDFSTATSENSEMBLUCSC | 64 | 10 | 25601 | tss | 0.841141 | 0.986677 | ISM1 (margin: ISM1) | 3.48 | 1.08e-05 | 4.82e-04 |
|  | 8 | 25902400 | 25902611 | PDFSTATSENSEMBLUCSC | 212 | 5 | 39608 | tss | 0.507975 | 0.086010 | EBF2 (margin: EBF2) | 3.48 | 7.94e-03 | 3.19e-02 |
|  | 17 | 37762842 | 37763088 | PDFSTATSENSEMBLUCSC | 247 | 5 | 16548 | gene;island | 0.123097 | 0.012799 | NEUROD2 (margin: NEUROD2) | 3.48 | 7.94e-03 | 3.19e-02 |
|  | 2 | 219925065 | 219925330 | PDFSTATSENSEMBLUCSC | 266 | 7 | 25105 | island | 0.844241 | 0.982482 | IHH; MIR3131 (margin: IHH; MIR3131) | 3.48 | 6.99e-03 | 3.19e-02 |
|  | 7 | 14028690 | 14029085 | PDFSTATSENSEMBLUCSC | 396 | 6 | 36898 | gene;island | 0.537135 | 0.079549 | ETV1 (margin: ETV1) | 3.48 | 4.33e-03 | 2.52e-02 |
|  | 6 | 168389700 | 168390347 | PDFSTATSENSEMBLUCSC | 648 | 6 | 36652 | island | 0.634189 | 0.955925 | (margin: KIF25; RP3-470B24.5; KIF25-AS1) | 3.48 | 2.16e-03 | 1.45e-02 |
|  | 1 | 186649153 | 186649985 | PDFSTATSENSEMBLUCSC | 833 | 13 | 4047 | island | 0.188892 | 0.018763 | PTGS2 (margin: PTGS2) | 3.47 | 2.97e-04 | 4.72e-03 |
|  | 6 | 32185818 | 32186244 | PDFSTATSENSEMBLUCSC | 427 | 9 | 34097 | gene | 0.239205 | 0.728843 | NOTCH4 (margin: NOTCH4) | 3.47 | 4.94e-04 | 6.47e-03 |
|  | 16 | 1041053 | 1041333 | PDFSTATSENSEMBLUCSC | 281 | 6 | 15505 | island | 0.604968 | 0.935065 | RP11-161M6.3 (margin: AC009041.2; SOX8; LMF1; RP11-161M6.2; RP11-161M6.3) | 3.47 | 8.66e-03 | 3.36e-02 |
|  | 13 | 100643858 | 100644657 | PDFSTATSENSEMBLUCSC | 800 | 8 | 11850 | island | 0.841986 | 0.971337 | (margin: ZIC2; LINC00554) | 3.47 | 1.86e-03 | 1.45e-02 |
|  | 20 | 39995539 | 39995747 | PDFSTATSENSEMBLUCSC | 209 | 7 | 25746 | tss;island | 0.379364 | 0.938970 | EMILIN3 (margin: LPIN3; EMILIN3) | 3.47 | 4.08e-03 | 2.47e-02 |
|  | 2 | 160918900 | 160919532 | PDFSTATSENSEMBLUCSC | 633 | 12 | 24813 | island | 0.066208 | 0.422863 | PLA2R1 (margin: PLA2R1) | 3.46 | 2.01e-04 | 3.38e-03 |
|  | 17 | 74137336 | 74137522 | PDFSTATSENSEMBLUCSC | 187 | 6 | 18774 | island | 0.068583 | 0.004016 | RNF157-AS1; FOXJ1; RNF157 (margin: RNF157-AS1; FOXJ1; RNF157) | 3.46 | 8.66e-03 | 3.36e-02 |
|  | 18 | 7567426 | 7567799 | PDFSTATSENSEMBLUCSC | 374 | 5 | 19052 | gene;island | 0.440375 | 0.930347 | PTPRM (margin: PTPRM) | 3.46 | 7.94e-03 | 3.19e-02 |
|  | 5 | 131346985 | 131347773 | PDFSTATSENSEMBLUCSC | 789 | 9 | 33083 | island | 0.611266 | 0.134113 | AC034228.2; ACSL6 (margin: AC034228.3; AC034228.2; ACSL6) | 3.46 | 5.64e-03 | 3.18e-02 |
|  | 2 | 169658881 | 169659182 | PDFSTATSENSEMBLUCSC | 302 | 6 | 22913 | gene | 0.660799 | 0.967709 | NOSTRIN (margin: NOSTRIN) | 3.46 | 8.66e-03 | 3.36e-02 |
|  | 10 | 43572065 | 43572795 | PDFSTATSENSEMBLUCSC | 731 | 9 | 5687 | island | 0.287745 | 0.017525 | RET (margin: RET) | 3.46 | 7.77e-03 | 3.19e-02 |
|  | 6 | 32188695 | 32189126 | PDFSTATSENSEMBLUCSC | 432 | 6 | 34099 | gene | 0.205905 | 0.781674 | NOTCH4 (margin: NOTCH4) | 3.45 | 2.16e-03 | 1.45e-02 |
|  | 8 | 140715540 | 140715802 | PDFSTATSENSEMBLUCSC | 263 | 5 | 39923 | tss;island | 0.752739 | 0.963524 | KCNK9 (margin: KCNK9) | 3.45 | 7.94e-03 | 3.19e-02 |
|  | 20 | 982623 | 983130 | PDFSTATSENSEMBLUCSC | 508 | 8 | 25931 | island | 0.722470 | 0.960996 | RSPO4 (margin: RSPO4) | 3.45 | 1.09e-03 | 1.10e-02 |
|  | 20 | 57428282 | 57428473 | PDFSTATSENSEMBLUCSC | 192 | 6 | 25472 | gene;island | 0.697752 | 0.962450 | GNAS (margin: GNAS; GNAS-AS1; RP1-309F20.3) | 3.44 | 2.16e-03 | 1.45e-02 |
|  | 3 | 136537607 | 136537863 | PDFSTATSENSEMBLUCSC | 257 | 9 | 29508 | island | 0.735570 | 0.962306 | SLC35G2 (margin: SLC35G2; RP11-731C17.1) | 3.44 | 4.11e-05 | 1.17e-03 |
|  | 7 | 45066738 | 45067057 | PDFSTATSENSEMBLUCSC | 320 | 5 | 36997 | gene;tss | 0.572416 | 0.917158 | CCM2 (margin: CCM2) | 3.44 | 7.94e-03 | 3.19e-02 |
|  | 8 | 11471395 | 11471881 | PDFSTATSENSEMBLUCSC | 487 | 5 | 40082 | island | 0.540813 | 0.927966 | (margin: ) | 3.44 | 7.94e-03 | 3.19e-02 |
|  | 10 | 42863444 | 42863594 | PDFSTATSENSEMBLUCSC | 151 | 5 | 5680 | island | 0.364125 | 0.723971 | (margin: ) | 3.44 | 7.94e-03 | 3.19e-02 |
|  | 1 | 1177886 | 1178245 | PDFSTATSENSEMBLUCSC | 360 | 5 | 23 | gene | 0.745299 | 0.969138 | FAM132A (margin: B3GALT6; SDF4; FAM132A; RP5-902P8.12; UBE2J2) | 3.44 | 7.94e-03 | 3.19e-02 |
|  | 11 | 64512829 | 64513289 | PDFSTATSENSEMBLUCSC | 461 | 6 | 7402 | tss;island | 0.515863 | 0.911892 | RASGRP2; PYGM (margin: RASGRP2; PYGM) | 3.44 | 2.16e-03 | 1.45e-02 |
|  | 21 | 28218481 | 28218959 | PDFSTATSENSEMBLUCSC | 479 | 5 | 26500 | tss;island | 0.771161 | 0.265980 | ADAMTS1 (margin: ADAMTS1) | 3.43 | 7.94e-03 | 3.19e-02 |
|  | 16 | 967929 | 968265 | PDFSTATSENSEMBLUCSC | 337 | 6 | 14554 | gene;island | 0.797807 | 0.969398 | LMF1 (margin: LA16c-366D3.1; LMF1) | 3.43 | 8.66e-03 | 3.36e-02 |
|  | 7 | 4347499 | 4348107 | PDFSTATSENSEMBLUCSC | 609 | 7 | 38187 | island | 0.447791 | 0.897156 | (margin: ) | 3.43 | 5.83e-04 | 6.47e-03 |
|  | 4 | 6107021 | 6107339 | PDFSTATSENSEMBLUCSC | 319 | 5 | 29925 | gene;island | 0.700225 | 0.968949 | JAKMIP1 (margin: JAKMIP1) | 3.43 | 7.94e-03 | 3.19e-02 |
|  | 5 | 121414025 | 121414425 | PDFSTATSENSEMBLUCSC | 401 | 13 | 33044 | island | 0.393905 | 0.077376 | LOX (margin: SRFBP1; LOX) | 3.42 | 8.65e-06 | 4.82e-04 |
|  | 5 | 121414067 | 121414425 | PDFSTATSENSEMBLUCSC | 359 | 11 | 32243 | tss | 0.408767 | 0.077376 | LOX (margin: SRFBP1; LOX) | 3.42 | 5.39e-05 | 1.47e-03 |
|  | 7 | 129425330 | 129425932 | PDFSTATSENSEMBLUCSC | 603 | 6 | 38913 | island | 0.613647 | 0.951669 | RP11-738B7.1 (margin: MIR96; MIR183; RP11-738B7.1) | 3.42 | 4.33e-03 | 2.52e-02 |
|  | 3 | 30936239 | 30936531 | PDFSTATSENSEMBLUCSC | 293 | 8 | 28201 | tss | 0.376818 | 0.054014 | GADL1 (margin: GADL1) | 3.42 | 6.99e-03 | 3.19e-02 |
|  | 11 | 2828214 | 2828778 | PDFSTATSENSEMBLUCSC | 565 | 7 | 6467 | gene;island | 0.184933 | 0.707890 | KCNQ1 (margin: KCNQ1) | 3.42 | 1.11e-02 | 4.14e-02 |
|  | 6 | 30881112 | 30882094 | PDFSTATSENSEMBLUCSC | 983 | 28 | 34793 | tss | 0.877260 | 0.068837 | GTF2H4; VARS2 (margin: RN7SL175P; GTF2H4; VARS2) | 3.42 | 6.32e-04 | 6.81e-03 |
|  | 11 | 69924046 | 69924390 | PDFSTATSENSEMBLUCSC | 345 | 10 | 7524 | tss;island | 0.155892 | 0.837857 | ANO1 (margin: RNU6-1175P; ANO1; ANO1-AS2) | 3.41 | 5.20e-03 | 2.96e-02 |
|  | 11 | 120110544 | 120110887 | PDFSTATSENSEMBLUCSC | 344 | 9 | 7761 | tss | 0.509804 | 0.924424 | POU2F3 (margin: OAF; POU2F3; RP11-778O17.4) | 3.41 | 4.94e-04 | 6.47e-03 |
|  | 5 | 87980882 | 87981449 | PDFSTATSENSEMBLUCSC | 568 | 7 | 32168 | tss;island | 0.844292 | 0.982850 | CTC-467M3.1; LINC00461 (margin: CTC-467M3.1; CTC-467M3.2; LINC00461; CTC-467M3.3) | 3.41 | 6.99e-03 | 3.19e-02 |
|  | 11 | 120110544 | 120111025 | PDFSTATSENSEMBLUCSC | 482 | 10 | 8893 | island | 0.508810 | 0.914843 | POU2F3 (margin: OAF; POU2F3; RP11-778O17.4) | 3.41 | 3.25e-04 | 4.91e-03 |
|  | 17 | 47210027 | 47210368 | PDFSTATSENSEMBLUCSC | 342 | 7 | 16672 | gene;island | 0.038473 | 0.008607 | B4GALNT2 (margin: B4GALNT2) | 3.40 | 1.11e-02 | 4.14e-02 |
|  | 19 | 19373566 | 19373755 | PDFSTATSENSEMBLUCSC | 190 | 5 | 21809 | island | 0.682450 | 0.957964 | HAPLN4; AC138430.4; TM6SF2 (margin: NCAN; HAPLN4; AC138430.4; TM6SF2) | 3.40 | 7.94e-03 | 3.19e-02 |
|  | 1 | 19600673 | 19601069 | PDFSTATSENSEMBLUCSC | 397 | 9 | 1301 | tss;island | 0.862707 | 0.985108 | AKR7L (margin: AKR7L; AKR7A3) | 3.40 | 2.88e-04 | 4.58e-03 |
|  | 10 | 82116203 | 82116596 | PDFSTATSENSEMBLUCSC | 394 | 10 | 4654 | gene;tss;island | 0.856716 | 0.980260 | DYDC2; DYDC1 (margin: DYDC2; DYDC1) | 3.40 | 1.08e-05 | 4.82e-04 |
|  | 1 | 24645802 | 24646392 | PDFSTATSENSEMBLUCSC | 591 | 13 | 2858 | island | 0.068427 | 0.006043 | GRHL3; RP11-10N16.3 (margin: GRHL3; RP11-10N16.3) | 3.40 | 1.25e-03 | 1.19e-02 |
|  | 14 | 101491377 | 101492406 | PDFSTATSENSEMBLUCSC | 1030 | 11 | 12621 | tss | 0.534747 | 0.894535 | MIR411; MIR299; MIR1197; MIR323A; MIR758; MIR329-1; MIR329-2; MIR380 (margin: MIR379; MIR411; MIR299; MIR1197; MIR323A; MIR758; MIR329-1; MIR329-2; MIR494; MIR1193; MIR543; MIR495; MIR380) | 3.40 | 8.33e-03 | 3.34e-02 |
|  | 1 | 1266468 | 1266700 | PDFSTATSENSEMBLUCSC | 233 | 5 | 1147 | tss | 0.257530 | 0.782159 | TAS1R3 (margin: GLTPD1; TAS1R3; CPSF3L; DVL1) | 3.40 | 7.94e-03 | 3.19e-02 |
|  | 6 | 84742998 | 84743364 | PDFSTATSENSEMBLUCSC | 367 | 10 | 35099 | tss;island | 0.282185 | 0.858128 | MRAP2 (margin: RP11-51G5.1; MRAP2) | 3.39 | 2.06e-04 | 3.42e-03 |
|  | 6 | 33160847 | 33161589 | PDFSTATSENSEMBLUCSC | 743 | 22 | 34890 | tss;island | 0.637827 | 0.974676 | COL11A2; RXRB (margin: RNY4P10; SLC39A7; HSD17B8; COL11A2; RXRB) | 3.39 | 1.06e-02 | 4.01e-02 |
|  | 7 | 116139180 | 116139425 | PDFSTATSENSEMBLUCSC | 246 | 7 | 37849 | tss | 0.110220 | 0.363104 | CAV2; AC002066.1 (margin: CAV2; AC002066.1) | 3.39 | 5.83e-04 | 6.47e-03 |
|  | 17 | 38334161 | 38334186 | PDFSTATSENSEMBLUCSC | 26 | 5 | 17326 | tss;island | 0.167223 | 0.658804 | RAPGEFL1 (margin: CASC3; RAPGEFL1) | 3.39 | 7.94e-03 | 3.19e-02 |
|  | 4 | 3464653 | 3465064 | PDFSTATSENSEMBLUCSC | 412 | 10 | 30839 | island | 0.371364 | 0.028259 | DOK7 (margin: DOK7) | 3.38 | 5.20e-03 | 2.96e-02 |
|  | 15 | 45406534 | 45406939 | PDFSTATSENSEMBLUCSC | 406 | 6 | 13326 | gene | 0.413827 | 0.835745 | DUOXA2; DUOX2 (margin: DUOXA2; DUOX2; DUOXA1) | 3.38 | 2.16e-03 | 1.45e-02 |
|  | 1 | 2344979 | 2345475 | PDFSTATSENSEMBLUCSC | 497 | 11 | 1173 | tss;island | 0.151810 | 0.028824 | PEX10 (margin: RER1; PLCH2; PEX10) | 3.38 | 5.39e-05 | 1.47e-03 |
|  | 17 | 29719023 | 29719431 | PDFSTATSENSEMBLUCSC | 409 | 5 | 16510 | gene;island | 0.636787 | 0.967381 | RAB11FIP4 (margin: NF1; RAB11FIP4) | 3.38 | 7.94e-03 | 3.19e-02 |
|  | 11 | 93063516 | 93064234 | PDFSTATSENSEMBLUCSC | 719 | 9 | 8727 | island | 0.571087 | 0.942023 | CCDC67 (margin: CCDC67) | 3.38 | 1.06e-02 | 4.01e-02 |
|  | 13 | 102104976 | 102105129 | PDFSTATSENSEMBLUCSC | 154 | 5 | 11213 | gene | 0.843664 | 0.464102 | ITGBL1 (margin: ITGBL1) | 3.37 | 7.94e-03 | 3.19e-02 |
|  | 16 | 2260770 | 2261237 | PDFSTATSENSEMBLUCSC | 468 | 10 | 15593 | island | 0.655808 | 0.951020 | MLST8; RP11-304L19.8; BRICD5; PGP (margin: MLST8; RP11-304L19.8; AC009065.3; BRICD5; PGP) | 3.37 | 4.87e-04 | 6.47e-03 |
|  | 19 | 51219975 | 51220537 | PDFSTATSENSEMBLUCSC | 563 | 10 | 22320 | island | 0.405063 | 0.883728 | SHANK1 (margin: CLEC11A; SHANK1) | 3.37 | 6.84e-03 | 3.19e-02 |
|  | 17 | 27346732 | 27347260 | PDFSTATSENSEMBLUCSC | 529 | 6 | 18164 | island | 0.677408 | 0.969269 | PIPOX; RP11-321A17.3 (margin: PIPOX; AC024619.2; RP11-321A17.5; RP11-321A17.3) | 3.37 | 8.66e-03 | 3.36e-02 |
|  | 19 | 53635967 | 53636398 | PDFSTATSENSEMBLUCSC | 432 | 8 | 22368 | island | 0.672489 | 0.965156 | ZNF415; ZNF347; CTD-2620I22.7 (margin: ZNF415; ZNF347; CTD-2620I22.7) | 3.36 | 1.04e-02 | 3.96e-02 |
|  | 5 | 169930841 | 169930938 | PDFSTATSENSEMBLUCSC | 98 | 6 | 32484 | tss | 0.517026 | 0.947366 | KCNIP1 (margin: KCNIP1) | 3.35 | 2.16e-03 | 1.45e-02 |
|  | 5 | 122429966 | 122430821 | PDFSTATSENSEMBLUCSC | 856 | 10 | 31730 | gene;island | 0.730700 | 0.969105 | PRDM6 (margin: PRDM6; AC106786.1) | 3.34 | 8.93e-03 | 3.45e-02 |
|  | 19 | 1449857 | 1450023 | PDFSTATSENSEMBLUCSC | 167 | 5 | 20306 | tss;island | 0.295988 | 0.033282 | APC2 (margin: RPS15; AC027307.3; APC2; CTB-25B13.12; C19orf25) | 3.34 | 7.94e-03 | 3.19e-02 |
|  | 1 | 205818956 | 205819609 | PDFSTATSENSEMBLUCSC | 654 | 8 | 4138 | island | 0.815614 | 0.984642 | PM20D1 (margin: PM20D1) | 3.33 | 4.66e-03 | 2.68e-02 |
|  | 6 | 84562778 | 84563098 | PDFSTATSENSEMBLUCSC | 321 | 9 | 36292 | island | 0.657288 | 0.971256 | RIPPLY2 (margin: RIPPLY2; CYB5R4) | 3.33 | 1.85e-03 | 1.45e-02 |
|  | 11 | 1855286 | 1855665 | PDFSTATSENSEMBLUCSC | 380 | 7 | 7047 | tss | 0.016628 | 0.140630 | SYT8 (margin: SYT8; TNNI2) | 3.33 | 1.11e-02 | 4.14e-02 |
|  | 2 | 466712 | 467135 | PDFSTATSENSEMBLUCSC | 424 | 8 | 24002 | island | 0.676559 | 0.967818 | (margin: ) | 3.32 | 6.99e-03 | 3.19e-02 |
|  | 4 | 42399699 | 42399858 | PDFSTATSENSEMBLUCSC | 160 | 7 | 31014 | island | 0.833178 | 0.982343 | SHISA3 (margin: SHISA3; RP11-63A11.1; ATP8A1) | 3.32 | 5.83e-04 | 6.47e-03 |
|  | 11 | 115530150 | 115530590 | PDFSTATSENSEMBLUCSC | 441 | 6 | 8829 | island | 0.268739 | 0.027701 | AP000797.3 (margin: AP000797.3) | 3.32 | 8.66e-03 | 3.36e-02 |
|  | 6 | 31508106 | 31508318 | PDFSTATSENSEMBLUCSC | 213 | 6 | 33894 | gene;island | 0.154720 | 0.550541 | DDX39B-AS1; DDX39B; ATP6V1G2-DDX39B; SNORD84 (margin: RPL15P4; MCCD1; DDX39B-AS1; NFKBIL1; DDX39B; ATP6V1G2-DDX39B; SNORD117; SNORD84; ATP6V1G2) | 3.32 | 4.33e-03 | 2.52e-02 |
|  | 3 | 138153127 | 138153439 | PDFSTATSENSEMBLUCSC | 313 | 9 | 29521 | island | 0.223319 | 0.700838 | ESYT3 (margin: ESYT3) | 3.31 | 2.88e-04 | 4.58e-03 |
|  | 20 | 13200685 | 13200992 | PDFSTATSENSEMBLUCSC | 308 | 12 | 26007 | island | 0.847832 | 0.985514 | ISM1 (margin: ISM1) | 3.31 | 7.40e-07 | 7.21e-05 |
|  | 2 | 222436947 | 222437130 | PDFSTATSENSEMBLUCSC | 184 | 8 | 25140 | island | 0.052708 | 0.007025 | EPHA4 (margin: EPHA4) | 3.31 | 1.09e-03 | 1.10e-02 |
|  | 18 | 6414958 | 6415252 | PDFSTATSENSEMBLUCSC | 295 | 9 | 19172 | tss;island | 0.527883 | 0.956500 | L3MBTL4 (margin: L3MBTL4) | 3.30 | 1.65e-04 | 2.80e-03 |
|  | 6 | 2903533 | 2904005 | PDFSTATSENSEMBLUCSC | 473 | 7 | 35379 | island | 0.889985 | 0.450722 | SERPINB9 (margin: SERPINB9; RP1-90J20.10) | 3.30 | 6.99e-03 | 3.19e-02 |
|  | 15 | 90792609 | 90793056 | PDFSTATSENSEMBLUCSC | 448 | 7 | 14450 | island | 0.455505 | 0.022243 | TTLL13; RP11-697E2.6 (margin: GDPGP1; RP11-697E2.4; TTLL13; RP11-697E2.6) | 3.30 | 4.08e-03 | 2.47e-02 |
|  | 10 | 43818243 | 43818911 | PDFSTATSENSEMBLUCSC | 669 | 8 | 5693 | island | 0.462587 | 0.946942 | RP11-168L22.2 (margin: RP11-168L22.2) | 3.29 | 1.04e-02 | 3.96e-02 |
|  | 15 | 28344273 | 28344730 | PDFSTATSENSEMBLUCSC | 458 | 13 | 13969 | island | 0.304239 | 0.940446 | OCA2 (margin: OCA2; HERC2) | 3.29 | 8.60e-03 | 3.36e-02 |
|  | 5 | 2564514 | 2564751 | PDFSTATSENSEMBLUCSC | 238 | 5 | 32653 | island | 0.804395 | 0.974510 | (margin: ) | 3.29 | 7.94e-03 | 3.19e-02 |
|  | 19 | 46999224 | 46999444 | PDFSTATSENSEMBLUCSC | 221 | 7 | 20893 | tss | 0.651436 | 0.958508 | AC011484.1; PPP5D1; PNMAL2 (margin: AC011484.1; PPP5D1; PNMAL2) | 3.28 | 2.33e-03 | 1.52e-02 |
|  | 14 | 96180319 | 96181044 | PDFSTATSENSEMBLUCSC | 726 | 11 | 13113 | island | 0.587336 | 0.971111 | RP11-164H13.1; TCL1A (margin: RP11-164H13.1; TCL1A) | 3.28 | 5.67e-06 | 3.54e-04 |
|  | 4 | 154073371 | 154074259 | PDFSTATSENSEMBLUCSC | 889 | 14 | 30639 | tss | 0.447257 | 0.023493 | TRIM2 (margin: TRIM2) | 3.28 | 2.96e-03 | 1.88e-02 |
|  | 13 | 113642457 | 113642784 | PDFSTATSENSEMBLUCSC | 328 | 6 | 11265 | gene | 0.747506 | 0.970027 | MCF2L (margin: MCF2L) | 3.27 | 2.16e-03 | 1.45e-02 |
|  | 6 | 42927940 | 42928546 | PDFSTATSENSEMBLUCSC | 607 | 25 | 36145 | island | 0.122307 | 0.014601 | GNMT (margin: RP3-475N16.1; GNMT; RPL24P4; PEX6) | 3.27 | 5.98e-08 | 1.06e-05 |
|  | 6 | 31324916 | 31325455 | PDFSTATSENSEMBLUCSC | 540 | 16 | 35768 | island | 0.016340 | 0.119231 | XXbac-BPG248L24.12; HLA-B (margin: XXbac-BPG248L24.12; HLA-B; DHFRP2) | 3.27 | 9.55e-03 | 3.69e-02 |
|  | 2 | 120281354 | 120282327 | PDFSTATSENSEMBLUCSC | 974 | 11 | 24657 | island | 0.865636 | 0.982762 | SCTR (margin: SCTR) | 3.27 | 1.28e-02 | 4.76e-02 |
|  | 4 | 154073371 | 154074387 | PDFSTATSENSEMBLUCSC | 1017 | 15 | 31370 | island | 0.422430 | 0.025247 | TRIM2 (margin: TRIM2) | 3.26 | 2.70e-03 | 1.76e-02 |
|  | 1 | 3191481 | 3191883 | PDFSTATSENSEMBLUCSC | 403 | 6 | 116 | gene;island | 0.617175 | 0.959315 | PRDM16 (margin: PRDM16) | 3.26 | 8.66e-03 | 3.36e-02 |
|  | 19 | 40005557 | 40005836 | PDFSTATSENSEMBLUCSC | 280 | 10 | 22001 | island | 0.532278 | 0.853407 | SELV (margin: DLL3; SELV) | 3.25 | 3.25e-04 | 4.91e-03 |
|  | 3 | 42814705 | 42814948 | PDFSTATSENSEMBLUCSC | 244 | 8 | 29020 | island | 0.870840 | 0.985517 | RP11-70C1.1; CCDC13; HIGD1A (margin: RP11-70C1.1; CCDC13; HIGD1A) | 3.25 | 4.66e-03 | 2.68e-02 |
|  | 5 | 140514711 | 140515207 | PDFSTATSENSEMBLUCSC | 497 | 6 | 33212 | island | 0.630734 | 0.956007 | PCDHB5 (margin: PCDHB4; PCDHB5) | 3.25 | 8.66e-03 | 3.36e-02 |
|  | 6 | 167275778 | 167275999 | PDFSTATSENSEMBLUCSC | 222 | 7 | 35338 | tss | 0.751792 | 0.184061 | RPS6KA2; RP11-514O12.4 (margin: RPS6KA2; RP11-514O12.4) | 3.25 | 5.83e-04 | 6.47e-03 |
|  | 21 | 34442160 | 34442674 | PDFSTATSENSEMBLUCSC | 515 | 10 | 26643 | island | 0.625716 | 0.976222 | OLIG1; AP000282.2 (margin: LINC00945; OLIG1; AP000282.2) | 3.24 | 2.88e-03 | 1.86e-02 |
|  | 15 | 100882213 | 100882647 | PDFSTATSENSEMBLUCSC | 435 | 8 | 13926 | tss | 0.159388 | 0.017360 | ADAMTS17 (margin: ADAMTS17; SPATA41) | 3.24 | 6.99e-03 | 3.19e-02 |
|  | 11 | 3253581 | 3254504 | PDFSTATSENSEMBLUCSC | 924 | 15 | 7996 | island | 0.483828 | 0.943079 | AC109309.4; MRGPRE (margin: MRGPRG-AS1; AC109309.4; MRGPRE) | 3.24 | 6.57e-03 | 3.19e-02 |
|  | 17 | 79099711 | 79100287 | PDFSTATSENSEMBLUCSC | 577 | 10 | 16894 | gene;tss;island | 0.690632 | 0.978000 | MIR338; AATK; MIR657 (margin: BAIAP2; MIR338; RP11-149I9.2; AATK; MIR657; MIR1250) | 3.24 | 3.89e-03 | 2.43e-02 |
|  | 6 | 46702683 | 46703670 | PDFSTATSENSEMBLUCSC | 988 | 19 | 36189 | island | 0.463165 | 0.930078 | PLA2G7 (margin: ANKRD66; PLA2G7) | 3.23 | 5.23e-06 | 3.42e-04 |
|  | 17 | 21280288 | 21280740 | PDFSTATSENSEMBLUCSC | 453 | 5 | 16489 | gene;island | 0.402064 | 0.781919 | KCNJ12 (margin: KCNJ12) | 3.23 | 7.94e-03 | 3.19e-02 |
|  | 3 | 124931535 | 124931746 | PDFSTATSENSEMBLUCSC | 212 | 5 | 29419 | island | 0.101737 | 0.500125 | SLC12A8 (margin: SLC12A8) | 3.23 | 7.94e-03 | 3.19e-02 |
|  | 4 | 41753439 | 41754125 | PDFSTATSENSEMBLUCSC | 687 | 8 | 31004 | island | 0.503217 | 0.827346 | RP11-227F19.1; RP11-227F19.2 (margin: RP11-227F19.1; RP11-227F19.2; PHOX2B) | 3.22 | 1.86e-03 | 1.45e-02 |
|  | 12 | 132689907 | 132690782 | PDFSTATSENSEMBLUCSC | 876 | 11 | 9434 | gene;island | 0.101196 | 0.757603 | GALNT9 (margin: GALNT9) | 3.22 | 1.86e-03 | 1.45e-02 |
|  | 3 | 42947263 | 42947771 | PDFSTATSENSEMBLUCSC | 509 | 7 | 29023 | island | 0.898288 | 0.987141 | ACKR2; KRBOX1; ZNF662 (margin: ACKR2; KRBOX1; ZNF662) | 3.22 | 5.83e-04 | 6.47e-03 |
|  | 7 | 99517136 | 99517509 | PDFSTATSENSEMBLUCSC | 374 | 12 | 38741 | island | 0.045404 | 0.337407 | TRIM4 (margin: RP4-604G5.1; TRIM4; GJC3) | 3.22 | 8.88e-06 | 4.82e-04 |
|  | 12 | 81471311 | 81471757 | PDFSTATSENSEMBLUCSC | 447 | 7 | 9894 | tss | 0.093108 | 0.017143 | ACSS3 (margin: ACSS3) | 3.22 | 5.83e-04 | 6.47e-03 |
|  | 17 | 65040527 | 65040983 | PDFSTATSENSEMBLUCSC | 457 | 10 | 18677 | island | 0.666991 | 0.969173 | CACNG1 (margin: CACNG4; CACNG1; RP11-74H8.1) | 3.21 | 7.25e-04 | 7.74e-03 |
|  | 19 | 46999055 | 46999444 | PDFSTATSENSEMBLUCSC | 390 | 10 | 22175 | island | 0.666773 | 0.963491 | AC011484.1; PPP5D1; PNMAL2 (margin: AC011484.1; PPP5D1; PNMAL2) | 3.21 | 1.15e-02 | 4.29e-02 |
|  | 5 | 169930841 | 169931363 | PDFSTATSENSEMBLUCSC | 523 | 9 | 31890 | gene;island | 0.583777 | 0.933579 | KCNIP1 (margin: KCNIP1) | 3.21 | 1.85e-03 | 1.45e-02 |
|  | 18 | 42259977 | 42260234 | PDFSTATSENSEMBLUCSC | 258 | 7 | 19249 | tss;island | 0.107152 | 0.015072 | SETBP1; RP11-456K23.1 (margin: SETBP1; RP11-456K23.1) | 3.21 | 5.83e-04 | 6.47e-03 |
|  | 1 | 160039962 | 160040667 | PDFSTATSENSEMBLUCSC | 706 | 11 | 2035 | tss | 0.362764 | 0.853883 | RP11-536C5.2; KCNJ10 (margin: RP11-536C5.2; KCNJ9; KCNJ10) | 3.20 | 1.86e-03 | 1.45e-02 |
|  | 17 | 7791882 | 7792191 | PDFSTATSENSEMBLUCSC | 310 | 6 | 16420 | gene;island | 0.254442 | 0.040153 | CHD3 (margin: CHD3; LSMD1) | 3.20 | 8.66e-03 | 3.36e-02 |
|  | 6 | 130339410 | 130339998 | PDFSTATSENSEMBLUCSC | 589 | 10 | 36475 | island | 0.112041 | 0.534391 | L3MBTL3 (margin: L3MBTL3) | 3.20 | 1.50e-03 | 1.41e-02 |
|  | 8 | 21644953 | 21645524 | PDFSTATSENSEMBLUCSC | 572 | 6 | 39224 | gene;island | 0.833643 | 0.951462 | GFRA2 (margin: GFRA2) | 3.20 | 4.33e-03 | 2.52e-02 |
|  | 17 | 46805785 | 46806134 | PDFSTATSENSEMBLUCSC | 350 | 5 | 18503 | island | 0.095870 | 0.776043 | HOXB13 (margin: HOXB-AS5; CTD-2377D24.4; PRAC; MIR3185; HOXB13) | 3.20 | 7.94e-03 | 3.19e-02 |
|  | 6 | 134639203 | 134639482 | PDFSTATSENSEMBLUCSC | 280 | 5 | 35235 | tss | 0.485189 | 0.878407 | SGK1 (margin: SGK1) | 3.19 | 7.94e-03 | 3.19e-02 |
|  | 4 | 74734714 | 74735149 | PDFSTATSENSEMBLUCSC | 436 | 10 | 31097 | island | 0.109363 | 0.421688 | CXCL1 (margin: CXCL1) | 3.19 | 5.20e-03 | 2.96e-02 |
|  | 11 | 61322742 | 61323413 | PDFSTATSENSEMBLUCSC | 672 | 6 | 6630 | gene;island | 0.655293 | 0.960546 | SYT7 (margin: SYT7) | 3.18 | 8.66e-03 | 3.36e-02 |
|  | 22 | 43505892 | 43506651 | PDFSTATSENSEMBLUCSC | 760 | 24 | 27200 | tss | 0.282503 | 0.031980 | BIK (margin: BIK) | 3.18 | 8.78e-03 | 3.41e-02 |
|  | 17 | 78865087 | 78865755 | PDFSTATSENSEMBLUCSC | 669 | 7 | 16881 | gene;island | 0.585803 | 0.921721 | RPTOR (margin: RPTOR) | 3.17 | 6.99e-03 | 3.19e-02 |
|  | 12 | 56040025 | 56040336 | PDFSTATSENSEMBLUCSC | 312 | 5 | 10507 | island | 0.876983 | 0.446474 | RP11-644F5.16 (margin: OR10P1; RP11-644F5.16; RP11-644F5.15) | 3.17 | 7.94e-03 | 3.19e-02 |
|  | 8 | 82192519 | 82192777 | PDFSTATSENSEMBLUCSC | 259 | 7 | 39784 | tss | 0.074019 | 0.450075 | FABP5; RP11-363E6.3 (margin: FABP5; RP11-363E6.4; RP11-363E6.3) | 3.17 | 5.83e-04 | 6.47e-03 |
|  | 1 | 1177703 | 1178245 | PDFSTATSENSEMBLUCSC | 543 | 6 | 2417 | island | 0.753598 | 0.972845 | FAM132A (margin: B3GALT6; SDF4; FAM132A; RP5-902P8.12; UBE2J2) | 3.17 | 4.33e-03 | 2.52e-02 |
|  | 5 | 134871645 | 134872315 | PDFSTATSENSEMBLUCSC | 671 | 18 | 32305 | tss | 0.681826 | 0.974917 | NEUROG1 (margin: NEUROG1) | 3.16 | 3.44e-05 | 1.17e-03 |
|  | 6 | 32062160 | 32062554 | PDFSTATSENSEMBLUCSC | 395 | 5 | 34045 | gene;island | 0.530061 | 0.904281 | TNXB (margin: TNXB; ATF6B) | 3.16 | 7.94e-03 | 3.19e-02 |
|  | 6 | 32363875 | 32364075 | PDFSTATSENSEMBLUCSC | 201 | 6 | 34106 | gene | 0.722917 | 0.967283 | BTNL2 (margin: HCG23; BTNL2) | 3.16 | 2.16e-03 | 1.45e-02 |
|  | 12 | 52626814 | 52627047 | PDFSTATSENSEMBLUCSC | 234 | 6 | 10437 | island | 0.847509 | 0.982064 | KRT7 (margin: LINC00592; KRT7; METTL7AP1; RP3-416H24.1) | 3.15 | 2.16e-03 | 1.45e-02 |
|  | 11 | 69634240 | 69634709 | PDFSTATSENSEMBLUCSC | 470 | 11 | 7523 | tss;island | 0.572511 | 0.945047 | FGF3 (margin: FGF3) | 3.15 | 3.18e-03 | 2.01e-02 |
|  | 5 | 1948875 | 1949277 | PDFSTATSENSEMBLUCSC | 403 | 5 | 32647 | island | 0.055897 | 0.380277 | RP11-259O2.1 (margin: RP11-259O2.1) | 3.15 | 7.94e-03 | 3.19e-02 |
|  | 1 | 2410885 | 2411643 | PDFSTATSENSEMBLUCSC | 759 | 9 | 85 | gene;island | 0.600498 | 0.965712 | PLCH2 (margin: PLCH2) | 3.15 | 1.23e-03 | 1.18e-02 |
|  | 12 | 103889772 | 103890274 | PDFSTATSENSEMBLUCSC | 503 | 9 | 9952 | tss | 0.533415 | 0.053521 | C12orf42 (margin: RP11-626I20.1; C12orf42; RP11-626I20.2) | 3.13 | 7.77e-03 | 3.19e-02 |
|  | 6 | 167275684 | 167275999 | PDFSTATSENSEMBLUCSC | 316 | 9 | 36637 | island | 0.751792 | 0.184061 | RPS6KA2; RP11-514O12.4 (margin: RPS6KA2; RP11-514O12.4) | 3.13 | 3.99e-03 | 2.47e-02 |
|  | 5 | 169532686 | 169532775 | PDFSTATSENSEMBLUCSC | 90 | 6 | 32482 | tss | 0.137076 | 0.528543 | FOXI1 (margin: FOXI1) | 3.13 | 8.66e-03 | 3.36e-02 |
|  | 3 | 5024761 | 5024963 | PDFSTATSENSEMBLUCSC | 203 | 5 | 27644 | gene;island | 0.829536 | 0.963436 | BHLHE40 (margin: BHLHE40; BHLHE40-AS1) | 3.12 | 7.94e-03 | 3.19e-02 |
|  | 11 | 107798913 | 107799287 | PDFSTATSENSEMBLUCSC | 375 | 10 | 8777 | island | 0.304828 | 0.026530 | RAB39A; SLC35F2 (margin: RAB39A; SLC35F2) | 3.12 | 3.25e-04 | 4.91e-03 |
|  | 3 | 42814752 | 42814948 | PDFSTATSENSEMBLUCSC | 197 | 7 | 28247 | tss | 0.878643 | 0.982948 | RP11-70C1.1; CCDC13; HIGD1A (margin: RP11-70C1.1; CCDC13; HIGD1A) | 3.12 | 1.11e-02 | 4.14e-02 |
|  | 5 | 140743780 | 140744155 | PDFSTATSENSEMBLUCSC | 376 | 6 | 31823 | gene;island | 0.614717 | 0.946463 | PCDHGA1; PCDHGA2; PCDHGA3; PCDHGB1; PCDHGA4; PCDHGB2; PCDHGA5 (margin: PCDHGA1; PCDHGA2; PCDHGA3; PCDHGB1; PCDHGA4; PCDHGB2; PCDHGA5; PCDHGB3; PCDHGA6) | 3.12 | 8.66e-03 | 3.36e-02 |
|  | 15 | 91642909 | 91643135 | PDFSTATSENSEMBLUCSC | 227 | 9 | 13910 | tss;island | 0.391796 | 0.727080 | SV2B (margin: SV2B) | 3.11 | 7.77e-03 | 3.19e-02 |
|  | 1 | 13910138 | 13910224 | PDFSTATSENSEMBLUCSC | 87 | 6 | 1261 | tss;island | 0.410967 | 0.858160 | PDPN (margin: PDPN) | 3.10 | 2.16e-03 | 1.45e-02 |
|  | 10 | 8094431 | 8094860 | PDFSTATSENSEMBLUCSC | 430 | 6 | 4487 | gene;island | 0.928300 | 0.596975 | RP11-379F12.4; GATA3; GATA3-AS1; RP11-379F12.3 (margin: RP11-379F12.4; GATA3; GATA3-AS1; RP11-379F12.3) | 3.10 | 2.16e-03 | 1.45e-02 |
|  | 11 | 8290295 | 8290476 | PDFSTATSENSEMBLUCSC | 182 | 5 | 8032 | island | 0.890313 | 0.973319 | LMO1 (margin: LMO1) | 3.10 | 7.94e-03 | 3.19e-02 |
|  | 20 | 56725695 | 56725899 | PDFSTATSENSEMBLUCSC | 205 | 8 | 25847 | tss | 0.757011 | 0.962071 | C20orf85 (margin: C20orf85) | 3.09 | 4.66e-03 | 2.68e-02 |
|  | 1 | 67772896 | 67773175 | PDFSTATSENSEMBLUCSC | 280 | 8 | 3365 | island | 0.868547 | 0.981446 | IL12RB2 (margin: IL12RB2) | 3.09 | 6.99e-03 | 3.19e-02 |
|  | 2 | 106959205 | 106959525 | PDFSTATSENSEMBLUCSC | 321 | 5 | 24592 | island | 0.733189 | 0.973352 | AC114755.3 (margin: AC114755.3) | 3.09 | 7.94e-03 | 3.19e-02 |
|  | 2 | 209271661 | 209272057 | PDFSTATSENSEMBLUCSC | 397 | 5 | 23000 | gene | 0.773230 | 0.942661 | PTH2R (margin: PTH2R) | 3.08 | 7.94e-03 | 3.19e-02 |
|  | 7 | 75157345 | 75157765 | PDFSTATSENSEMBLUCSC | 421 | 9 | 38585 | island | 0.008794 | 0.042409 | PMS2P3 (margin: PMS2P3; HIP1) | 3.08 | 7.82e-04 | 8.17e-03 |
|  | 11 | 116694005 | 116694372 | PDFSTATSENSEMBLUCSC | 368 | 6 | 8837 | island | 0.562780 | 0.912153 | APOA4 (margin: APOC3; AP006216.5; APOA4) | 3.08 | 8.66e-03 | 3.36e-02 |
|  | 10 | 52750736 | 52751341 | PDFSTATSENSEMBLUCSC | 606 | 8 | 5746 | island | 0.851085 | 0.348427 | PRKG1 (margin: PRKG1) | 3.08 | 3.11e-04 | 4.77e-03 |
|  | 12 | 54426498 | 54427884 | PDFSTATSENSEMBLUCSC | 1387 | 17 | 9160 | gene;island | 0.066249 | 0.401070 | HOXC5; HOXC6; HOXC4; MIR615 (margin: HOXC5; HOXC6; HOXC4; AC012531.25; MIR615) | 3.07 | 7.61e-03 | 3.19e-02 |
|  | 4 | 3742095 | 3742357 | PDFSTATSENSEMBLUCSC | 263 | 5 | 30852 | island | 0.517326 | 0.947119 | (margin: ) | 3.07 | 7.94e-03 | 3.19e-02 |
|  | 20 | 56725695 | 56726185 | PDFSTATSENSEMBLUCSC | 491 | 11 | 26286 | island | 0.732469 | 0.958262 | C20orf85 (margin: C20orf85) | 3.07 | 1.90e-04 | 3.21e-03 |
|  | 1 | 201617847 | 201618284 | PDFSTATSENSEMBLUCSC | 438 | 6 | 940 | gene;island | 0.603554 | 0.146408 | NAV1 (margin: NAV1) | 3.07 | 4.33e-03 | 2.52e-02 |
|  | 7 | 75931606 | 75932007 | PDFSTATSENSEMBLUCSC | 402 | 11 | 38597 | island | 0.163115 | 0.015843 | HSPB1 (margin: HSPB1) | 3.07 | 2.45e-03 | 1.59e-02 |
|  | 4 | 111544271 | 111544463 | PDFSTATSENSEMBLUCSC | 193 | 7 | 30549 | tss | 0.050937 | 0.008848 | PITX2 (margin: RP11-380D23.2; PITX2) | 3.07 | 1.11e-02 | 4.14e-02 |
|  | 11 | 65339691 | 65339797 | PDFSTATSENSEMBLUCSC | 107 | 5 | 7434 | tss;island | 0.003847 | 0.025951 | SSSCA1; FAM89B; SSSCA1-AS1 (margin: SSSCA1; FAM89B; EHBP1L1; SSSCA1-AS1) | 3.06 | 7.94e-03 | 3.19e-02 |
|  | 14 | 77647955 | 77648283 | PDFSTATSENSEMBLUCSC | 329 | 8 | 13031 | island | 0.059544 | 0.008973 | RP11-463C8.4; TMEM63C (margin: RP11-463C8.4; TMEM63C; RP11-463C8.5) | 3.06 | 1.04e-02 | 3.96e-02 |
|  | 1 | 77747875 | 77748480 | PDFSTATSENSEMBLUCSC | 606 | 9 | 594 | gene;island | 0.454476 | 0.033711 | AK5 (margin: AK5) | 3.06 | 1.06e-02 | 4.01e-02 |
|  | 1 | 1395945 | 1396296 | PDFSTATSENSEMBLUCSC | 352 | 7 | 37 | gene;island | 0.669091 | 0.979590 | ATAD3C (margin: ATAD3C; ATAD3B) | 3.06 | 4.08e-03 | 2.47e-02 |
|  | 11 | 2322286 | 2323459 | PDFSTATSENSEMBLUCSC | 1174 | 23 | 6454 | gene;tss | 0.453420 | 0.904338 | TSPAN32; C11orf21 (margin: TSPAN32; C11orf21) | 3.06 | 5.58e-05 | 1.52e-03 |
|  | 16 | 1030383 | 1030619 | PDFSTATSENSEMBLUCSC | 237 | 8 | 14986 | tss;island | 0.828697 | 0.984763 | AC009041.2; SOX8; LMF1; RP11-161M6.2 (margin: AC009041.2; SOX8; LMF1; RP11-161M6.2; RP11-161M6.3) | 3.05 | 2.95e-03 | 1.88e-02 |
|  | 3 | 122399230 | 122399601 | PDFSTATSENSEMBLUCSC | 372 | 7 | 28540 | tss | 0.014170 | 0.150127 | PARP14 (margin: PARP14) | 3.05 | 4.08e-03 | 2.47e-02 |
|  | 11 | 14993866 | 14995322 | PDFSTATSENSEMBLUCSC | 1457 | 34 | 7167 | tss | 0.575779 | 0.961273 | CALCB; CALCA (margin: CALCB; CALCA) | 3.05 | 7.89e-07 | 7.54e-05 |
|  | 2 | 131512651 | 131513216 | PDFSTATSENSEMBLUCSC | 566 | 12 | 23629 | tss;island | 0.582384 | 0.912881 | AMER3; AC140481.8 (margin: AMER3; AC140481.8) | 3.04 | 1.03e-04 | 2.47e-03 |
|  | 12 | 114841536 | 114842031 | PDFSTATSENSEMBLUCSC | 496 | 8 | 9325 | gene;island | 0.706190 | 0.978781 | TBX5 (margin: TBX5-AS1; TBX5) | 3.03 | 1.86e-03 | 1.45e-02 |
|  | 4 | 54965829 | 54966247 | PDFSTATSENSEMBLUCSC | 419 | 6 | 31051 | island | 0.561406 | 0.921350 | FIP1L1; GSX2 (margin: FIP1L1; MORF4L2P1; GSX2; AC110298.1) | 3.03 | 8.66e-03 | 3.36e-02 |
|  | 16 | 89114613 | 89114919 | PDFSTATSENSEMBLUCSC | 307 | 5 | 16236 | island | 0.562019 | 0.912537 | CTD-2555A7.2 (margin: CTD-2555A7.2) | 3.03 | 7.94e-03 | 3.19e-02 |
|  | 5 | 134871462 | 134872315 | PDFSTATSENSEMBLUCSC | 854 | 21 | 33138 | island | 0.641149 | 0.972742 | NEUROG1 (margin: NEUROG1) | 3.02 | 1.23e-05 | 5.38e-04 |
|  | 13 | 27131484 | 27131780 | PDFSTATSENSEMBLUCSC | 297 | 9 | 11355 | tss;island | 0.174481 | 0.011724 | WASF3 (margin: WASF3) | 3.02 | 7.82e-04 | 8.17e-03 |
|  | 2 | 242801896 | 242802192 | PDFSTATSENSEMBLUCSC | 297 | 6 | 23995 | tss;island | 0.661121 | 0.967204 | PDCD1 (margin: CXXC11; PDCD1) | 3.02 | 4.33e-03 | 2.52e-02 |
|  | 5 | 178487123 | 178488100 | PDFSTATSENSEMBLUCSC | 978 | 14 | 33479 | island | 0.116434 | 0.521014 | ZNF354C (margin: ZNF354C) | 3.02 | 2.96e-03 | 1.88e-02 |
|  | 11 | 18433500 | 18433745 | PDFSTATSENSEMBLUCSC | 246 | 6 | 7184 | tss | 0.880257 | 0.969245 | LDHC (margin: LDHA; AC084117.3; LDHC) | 3.02 | 8.66e-03 | 3.36e-02 |
|  | 5 | 176314231 | 176314737 | PDFSTATSENSEMBLUCSC | 507 | 6 | 31913 | gene;island | 0.506191 | 0.905026 | HK3 (margin: UNC5A; HK3) | 3.02 | 8.66e-03 | 3.36e-02 |
|  | 5 | 52083512 | 52084055 | PDFSTATSENSEMBLUCSC | 544 | 13 | 32058 | tss;island | 0.016522 | 0.114728 | ITGA1; PELO; CTD-2288O8.1 (margin: ITGA1; PELO; CTD-2288O8.1) | 3.02 | 2.35e-03 | 1.53e-02 |
|  | 6 | 33180841 | 33181565 | PDFSTATSENSEMBLUCSC | 725 | 14 | 35985 | island | 0.101880 | 0.436361 | RING1; ZNF70P1 (margin: SLC39A7; HSD17B8; MIR219-1; RING1; ZNF70P1) | 3.02 | 1.18e-03 | 1.14e-02 |
|  | 17 | 79410470 | 79410752 | PDFSTATSENSEMBLUCSC | 283 | 5 | 16909 | gene;island | 0.637046 | 0.960364 | RP11-1055B8.7 (margin: RP11-1055B8.7; MIR3186) | 3.01 | 7.94e-03 | 3.19e-02 |
|  | 5 | 126365232 | 126365876 | PDFSTATSENSEMBLUCSC | 645 | 6 | 31736 | gene;island | 0.109818 | 0.012095 | MARCH3 (margin: MARCH3) | 3.01 | 2.16e-03 | 1.45e-02 |
|  | 2 | 160919147 | 160919532 | PDFSTATSENSEMBLUCSC | 386 | 10 | 23684 | tss | 0.082234 | 0.430195 | PLA2R1 (margin: PLA2R1) | 3.01 | 1.50e-03 | 1.41e-02 |
|  | 3 | 46939978 | 46940439 | PDFSTATSENSEMBLUCSC | 462 | 5 | 27758 | gene;island | 0.850327 | 0.985406 | PTH1R (margin: PTH1R; AC109583.1) | 3.01 | 7.94e-03 | 3.19e-02 |
|  | 6 | 30853948 | 30854233 | PDFSTATSENSEMBLUCSC | 286 | 8 | 33827 | gene;island | 0.013970 | 0.109298 | DDR1 (margin: DDR1; MIR4640; DDR1-AS1) | 3.01 | 1.04e-02 | 3.96e-02 |
|  | 5 | 140736762 | 140737111 | PDFSTATSENSEMBLUCSC | 350 | 5 | 31821 | gene;island | 0.584983 | 0.899338 | PCDHGA1; PCDHGA2; PCDHGA3; PCDHGB1; PCDHGA4 (margin: PCDHGA1; PCDHGA2; PCDHGA3; PCDHGB1; PCDHGA4; PCDHGB2; PCDHGA5) | 3.01 | 7.94e-03 | 3.19e-02 |
|  | 17 | 1627652 | 1627862 | PDFSTATSENSEMBLUCSC | 211 | 7 | 16320 | gene;tss;island | 0.279942 | 0.019005 | WDR81; RP11-961A15.1 (margin: WDR81; MIR22HG; RP11-961A15.1) | 3.00 | 1.11e-02 | 4.14e-02 |
|  | 1 | 231296640 | 231297103 | PDFSTATSENSEMBLUCSC | 464 | 5 | 4323 | island | 0.832683 | 0.974919 | TRIM67 (margin: TRIM67) | 3.00 | 7.94e-03 | 3.19e-02 |
|  | 3 | 181429233 | 181429704 | PDFSTATSENSEMBLUCSC | 472 | 9 | 28042 | gene;tss;island | 0.030949 | 0.006170 | SOX2-OT; SOX2 (margin: SOX2-OT; SOX2) | 3.00 | 1.06e-02 | 4.01e-02 |
|  | 2 | 74875227 | 74875548 | PDFSTATSENSEMBLUCSC | 322 | 8 | 23465 | tss;island | 0.881241 | 0.986365 | M1AP (margin: SEMA4F; M1AP) | 3.00 | 1.55e-04 | 2.71e-03 |
|  | 12 | 72667326 | 72667707 | PDFSTATSENSEMBLUCSC | 382 | 7 | 9877 | tss | 0.221887 | 0.698764 | TRHDE; TRHDE-AS1 (margin: TRHDE; TRHDE-AS1) | 2.99 | 5.83e-04 | 6.47e-03 |
|  | 19 | 54041163 | 54041329 | PDFSTATSENSEMBLUCSC | 167 | 5 | 21031 | tss | 0.810397 | 0.421753 | ZNF331 (margin: ZNF331) | 2.99 | 7.94e-03 | 3.19e-02 |
|  | 4 | 76555547 | 76556042 | PDFSTATSENSEMBLUCSC | 496 | 11 | 31107 | island | 0.079013 | 0.449509 | CDKL2 (margin: CDKL2; G3BP2) | 2.98 | 2.84e-06 | 1.96e-04 |
|  | 3 | 153838788 | 153839287 | PDFSTATSENSEMBLUCSC | 500 | 8 | 29599 | island | 0.268477 | 0.011175 | ARHGEF26; ARHGEF26-AS1 (margin: ARHGEF26; ARHGEF26-AS1) | 2.98 | 6.99e-03 | 3.19e-02 |
|  | 11 | 74459620 | 74459861 | PDFSTATSENSEMBLUCSC | 242 | 7 | 7562 | tss | 0.003658 | 0.012680 | RNF169 (margin: RNF169; RP11-864N7.1; RP11-864N7.2; AP000560.1) | 2.98 | 6.99e-03 | 3.19e-02 |
|  | 15 | 40545143 | 40545511 | PDFSTATSENSEMBLUCSC | 369 | 9 | 13585 | tss | 0.794845 | 0.968249 | RP11-133K1.2; PAK6; C15orf56 (margin: RP11-133K1.2; PAK6; C15orf56) | 2.98 | 1.65e-04 | 2.80e-03 |
|  | 1 | 1115378 | 1115920 | PDFSTATSENSEMBLUCSC | 543 | 5 | 19 | gene;island | 0.872427 | 0.982155 | TTLL10; TTLL10-AS1 (margin: MIR429; RP11-465B22.8; TTLL10; TTLL10-AS1) | 2.98 | 7.94e-03 | 3.19e-02 |
|  | 17 | 74533845 | 74534090 | PDFSTATSENSEMBLUCSC | 246 | 7 | 17681 | tss;island | 0.919732 | 0.982989 | PRCD; CYGB (margin: PRCD; RP11-666A8.7; CYGB; RP11-666A8.8) | 2.98 | 5.83e-04 | 6.47e-03 |
|  | 6 | 44186914 | 44187226 | PDFSTATSENSEMBLUCSC | 313 | 9 | 35007 | tss | 0.844003 | 0.345616 | RP1-302G2.5; SLC29A1 (margin: RP1-302G2.5; SLC29A1) | 2.97 | 1.65e-04 | 2.80e-03 |
|  | 1 | 15271830 | 15272326 | PDFSTATSENSEMBLUCSC | 497 | 6 | 241 | gene;tss | 0.548713 | 0.940837 | KAZN (margin: KAZN) | 2.97 | 2.16e-03 | 1.45e-02 |
|  | 19 | 54041163 | 54041398 | PDFSTATSENSEMBLUCSC | 236 | 6 | 20206 | gene;island | 0.802056 | 0.429679 | ZNF331 (margin: ZNF331) | 2.97 | 2.16e-03 | 1.45e-02 |
|  | 1 | 205425468 | 205425944 | PDFSTATSENSEMBLUCSC | 477 | 5 | 4128 | island | 0.529758 | 0.917859 | RP11-576D8.4; LEMD1 (margin: RP11-576D8.4; LEMD1; MIR135B) | 2.97 | 7.94e-03 | 3.19e-02 |
|  | 11 | 107798913 | 107799237 | PDFSTATSENSEMBLUCSC | 325 | 9 | 7666 | tss | 0.311595 | 0.029098 | RAB39A; SLC35F2 (margin: RAB39A; SLC35F2) | 2.97 | 1.23e-03 | 1.18e-02 |
|  | 4 | 74718978 | 74719289 | PDFSTATSENSEMBLUCSC | 312 | 5 | 31096 | island | 0.915464 | 0.581288 | PF4V1 (margin: CXCL6; PPBPP1; PF4V1) | 2.97 | 7.94e-03 | 3.19e-02 |
|  | 19 | 21106002 | 21106053 | PDFSTATSENSEMBLUCSC | 52 | 6 | 20657 | tss | 0.752598 | 0.285301 | ZNF85 (margin: ZNF85) | 2.96 | 2.16e-03 | 1.45e-02 |
|  | 12 | 52626814 | 52626904 | PDFSTATSENSEMBLUCSC | 91 | 5 | 9725 | tss | 0.840879 | 0.981483 | KRT7 (margin: LINC00592; KRT7; METTL7AP1; RP3-416H24.1) | 2.96 | 7.94e-03 | 3.19e-02 |
|  | 6 | 161188022 | 161188822 | PDFSTATSENSEMBLUCSC | 801 | 8 | 36615 | island | 0.670357 | 0.934106 | (margin: ) | 2.96 | 6.99e-03 | 3.19e-02 |
|  | 5 | 24644988 | 24645147 | PDFSTATSENSEMBLUCSC | 160 | 5 | 32002 | tss | 0.334124 | 0.038743 | CDH10 (margin: CDH10) | 2.96 | 7.94e-03 | 3.19e-02 |
|  | 1 | 185703201 | 185703877 | PDFSTATSENSEMBLUCSC | 677 | 13 | 4045 | island | 0.158705 | 0.017050 | HMCN1 (margin: HMCN1) | 2.95 | 2.28e-04 | 3.75e-03 |
|  | 1 | 185703201 | 185703659 | PDFSTATSENSEMBLUCSC | 459 | 11 | 2144 | tss | 0.158705 | 0.017050 | HMCN1 (margin: HMCN1) | 2.95 | 5.53e-04 | 6.47e-03 |
|  | 2 | 223176606 | 223177008 | PDFSTATSENSEMBLUCSC | 403 | 5 | 25150 | island | 0.899745 | 0.976073 | (margin: CCDC140; AC010980.2; RP11-384O8.1) | 2.95 | 7.94e-03 | 3.19e-02 |
|  | 20 | 23402051 | 23402404 | PDFSTATSENSEMBLUCSC | 354 | 10 | 26051 | island | 0.001629 | 0.004748 | NAPB (margin: NAPB) | 2.95 | 2.88e-03 | 1.86e-02 |
|  | 12 | 39299132 | 39299844 | PDFSTATSENSEMBLUCSC | 713 | 9 | 10325 | island | 0.450830 | 0.897062 | RP11-396F22.1; CPNE8 (margin: RP11-396F22.1; CPNE8; AC067735.1) | 2.95 | 4.11e-05 | 1.17e-03 |
|  | 8 | 101661767 | 101662246 | PDFSTATSENSEMBLUCSC | 480 | 9 | 40498 | island | 0.879867 | 0.978901 | SNX31 (margin: SNX31) | 2.95 | 7.82e-04 | 8.17e-03 |
|  | 11 | 1083178 | 1083509 | PDFSTATSENSEMBLUCSC | 332 | 6 | 6402 | gene;island | 0.760257 | 0.958687 | MUC2 (margin: MUC2) | 2.95 | 4.33e-03 | 2.52e-02 |
|  | 6 | 46703193 | 46703670 | PDFSTATSENSEMBLUCSC | 478 | 15 | 35022 | tss | 0.463165 | 0.930078 | PLA2G7 (margin: ANKRD66; PLA2G7) | 2.95 | 8.98e-05 | 2.18e-03 |
|  | 8 | 82192519 | 82192931 | PDFSTATSENSEMBLUCSC | 413 | 10 | 40417 | island | 0.102944 | 0.433480 | FABP5; RP11-363E6.3 (margin: FABP5; RP11-363E6.4; RP11-363E6.3) | 2.94 | 1.08e-05 | 4.82e-04 |
|  | 6 | 169026928 | 169027218 | PDFSTATSENSEMBLUCSC | 291 | 5 | 34565 | gene | 0.861789 | 0.971820 | SMOC2 (margin: SMOC2) | 2.94 | 7.94e-03 | 3.19e-02 |
|  | 17 | 6735063 | 6735316 | PDFSTATSENSEMBLUCSC | 254 | 8 | 17054 | tss | 0.139902 | 0.020322 | TEKT1 (margin: TEKT1) | 2.94 | 4.66e-03 | 2.68e-02 |
|  | 16 | 84328104 | 84328391 | PDFSTATSENSEMBLUCSC | 288 | 6 | 15392 | tss;island | 0.139590 | 0.599424 | WFDC1; RP11-558A11.2 (margin: WFDC1; RP11-558A11.2) | 2.94 | 8.66e-03 | 3.36e-02 |
|  | 6 | 33156779 | 33157222 | PDFSTATSENSEMBLUCSC | 444 | 10 | 34213 | gene;island | 0.377141 | 0.835403 | COL11A2 (margin: RNY4P10; SLC39A7; COL11A2; RXRB) | 2.94 | 8.93e-03 | 3.45e-02 |
|  | 8 | 102504447 | 102504859 | PDFSTATSENSEMBLUCSC | 413 | 9 | 40506 | island | 0.043483 | 0.005669 | GRHL2; KB-1562D12.1 (margin: GRHL2; KB-1562D12.1) | 2.93 | 3.99e-03 | 2.47e-02 |
|  | 11 | 67777618 | 67777952 | PDFSTATSENSEMBLUCSC | 335 | 8 | 6766 | gene | 0.015284 | 0.111954 | ALDH3B1 (margin: ALDH3B1; UNC93B1) | 2.93 | 3.11e-04 | 4.77e-03 |
|  | 6 | 26240307 | 26240579 | PDFSTATSENSEMBLUCSC | 273 | 5 | 34699 | tss;island | 0.976786 | 0.775857 | HIST1H4F (margin: HIST1H2APS3; HIST1H4F; HIST1H2BH; HIST1H1D; HIST1H4G; HIST1H3F) | 2.92 | 7.94e-03 | 3.19e-02 |
|  | 17 | 41277694 | 41279022 | PDFSTATSENSEMBLUCSC | 1329 | 28 | 16595 | gene | 0.009157 | 0.137068 | NBR2; BRCA1 (margin: NBR2; BRCA1) | 2.92 | 3.69e-03 | 2.33e-02 |
|  | 7 | 121784351 | 121784596 | PDFSTATSENSEMBLUCSC | 246 | 7 | 37864 | tss | 0.074548 | 0.378571 | AASS (margin: AASS) | 2.92 | 4.08e-03 | 2.47e-02 |
|  | 14 | 74705942 | 74706336 | PDFSTATSENSEMBLUCSC | 395 | 8 | 12997 | island | 0.622617 | 0.876807 | VSX2 (margin: VSX2) | 2.92 | 1.55e-04 | 2.71e-03 |
|  | 14 | 74705942 | 74706113 | PDFSTATSENSEMBLUCSC | 172 | 6 | 12505 | tss | 0.630797 | 0.862092 | VSX2 (margin: VSX2) | 2.92 | 2.16e-03 | 1.45e-02 |
|  | 5 | 118603742 | 118603938 | PDFSTATSENSEMBLUCSC | 197 | 5 | 32236 | tss;island | 0.038421 | 0.008623 | TNFAIP8 (margin: TNFAIP8; AC027320.1) | 2.91 | 7.94e-03 | 3.19e-02 |
|  | 13 | 36919674 | 36919960 | PDFSTATSENSEMBLUCSC | 287 | 5 | 11130 | gene;island | 0.916728 | 0.988508 | SPG20OS; SPG20 (margin: SPG20OS; AL139377.1; SPG20) | 2.91 | 7.94e-03 | 3.19e-02 |
|  | 1 | 182361258 | 182361557 | PDFSTATSENSEMBLUCSC | 300 | 7 | 2131 | tss;island | 0.311984 | 0.725665 | GLUL (margin: GLUL; TEDDM1) | 2.91 | 1.17e-03 | 1.13e-02 |
|  | 14 | 100111183 | 100111392 | PDFSTATSENSEMBLUCSC | 210 | 8 | 12599 | tss;island | 0.819104 | 0.943878 | HHIPL1 (margin: HHIPL1) | 2.91 | 1.04e-02 | 3.96e-02 |
|  | 10 | 49892741 | 49893026 | PDFSTATSENSEMBLUCSC | 286 | 8 | 5068 | tss | 0.564010 | 0.921854 | WDFY4 (margin: WDFY4) | 2.91 | 1.86e-03 | 1.45e-02 |
|  | 6 | 44186914 | 44187674 | PDFSTATSENSEMBLUCSC | 761 | 12 | 36171 | island | 0.866913 | 0.360541 | RP1-302G2.5; SLC29A1 (margin: RP1-302G2.5; SLC29A1) | 2.90 | 3.33e-05 | 1.17e-03 |
|  | 17 | 72931569 | 72931857 | PDFSTATSENSEMBLUCSC | 289 | 10 | 17637 | tss | 0.514874 | 0.859491 | OTOP2; OTOP3 (margin: OTOP2; OTOP3) | 2.90 | 7.58e-05 | 1.98e-03 |
|  | 6 | 32632565 | 32633163 | PDFSTATSENSEMBLUCSC | 599 | 22 | 34126 | gene;island | 0.537709 | 0.936018 | HLA-DQB1 (margin: HLA-DQB1-AS1; HLA-DQB1; XXbac-BPG254F23.6) | 2.90 | 1.03e-05 | 4.82e-04 |
|  | 17 | 1811181 | 1811625 | PDFSTATSENSEMBLUCSC | 445 | 5 | 17833 | island | 0.831830 | 0.971403 | (margin: RPA1) | 2.89 | 7.94e-03 | 3.19e-02 |
|  | 3 | 138153127 | 138153381 | PDFSTATSENSEMBLUCSC | 255 | 8 | 28623 | tss | 0.213739 | 0.663162 | ESYT3 (margin: ESYT3) | 2.89 | 1.55e-04 | 2.71e-03 |
|  | 1 | 156211409 | 156212005 | PDFSTATSENSEMBLUCSC | 597 | 11 | 3848 | island | 0.448341 | 0.893082 | PMF1-BGLAP; PMF1; BGLAP; PAQR6 (margin: PMF1-BGLAP; PMF1; BGLAP; PAQR6; SMG5) | 2.88 | 7.65e-04 | 8.13e-03 |
|  | 1 | 179711859 | 179712361 | PDFSTATSENSEMBLUCSC | 503 | 10 | 4001 | island | 0.775828 | 0.961744 | RP11-12M5.1; FAM163A (margin: RP11-12M5.1; FAM163A) | 2.88 | 7.58e-05 | 1.98e-03 |
|  | 14 | 96180576 | 96181044 | PDFSTATSENSEMBLUCSC | 469 | 9 | 12590 | tss | 0.728216 | 0.977181 | RP11-164H13.1; TCL1A (margin: RP11-164H13.1; TCL1A) | 2.88 | 8.23e-05 | 2.06e-03 |
|  | 11 | 30038615 | 30038797 | PDFSTATSENSEMBLUCSC | 183 | 9 | 7218 | tss;island | 0.870632 | 0.514975 | KCNA4 (margin: KCNA4) | 2.88 | 1.23e-03 | 1.18e-02 |
|  | 1 | 78511140 | 78511488 | PDFSTATSENSEMBLUCSC | 349 | 7 | 1710 | tss | 0.918831 | 0.980894 | GIPC2; RP11-386I14.2 (margin: GIPC2; RP11-386I14.2) | 2.87 | 6.99e-03 | 3.19e-02 |
|  | 19 | 19050971 | 19051482 | PDFSTATSENSEMBLUCSC | 512 | 8 | 19944 | gene;tss;island | 0.013192 | 0.083671 | AC005932.1; HOMER3 (margin: DDX49; AC005932.1; AC002985.3; HOMER3) | 2.87 | 2.95e-03 | 1.88e-02 |
|  | 6 | 122931154 | 122931308 | PDFSTATSENSEMBLUCSC | 155 | 8 | 35206 | tss | 0.112124 | 0.014630 | PKIB (margin: PKIB) | 2.87 | 1.09e-03 | 1.10e-02 |
|  | 17 | 8230130 | 8230940 | PDFSTATSENSEMBLUCSC | 811 | 7 | 18001 | island | 0.816762 | 0.971844 | SNORA69 (margin: ARHGEF15; SNORA69; RP11-849F2.4; AC135178.7) | 2.86 | 5.83e-04 | 6.47e-03 |
|  | 13 | 25745946 | 25746644 | PDFSTATSENSEMBLUCSC | 699 | 11 | 11350 | tss | 0.657428 | 0.917557 | AMER2; RP11-165I9.4 (margin: RP11-165I9.8; AMER2; RP11-165I9.4) | 2.85 | 4.10e-03 | 2.48e-02 |
|  | 16 | 62069806 | 62070365 | PDFSTATSENSEMBLUCSC | 560 | 11 | 15985 | island | 0.855866 | 0.387991 | CDH8 (margin: CDH8) | 2.85 | 1.28e-02 | 4.76e-02 |
|  | 11 | 86383182 | 86383585 | PDFSTATSENSEMBLUCSC | 404 | 12 | 6854 | gene | 0.796912 | 0.961128 | ME3 (margin: ME3) | 2.85 | 7.40e-07 | 7.21e-05 |
|  | 6 | 87646738 | 87647154 | PDFSTATSENSEMBLUCSC | 417 | 11 | 36305 | island | 0.426752 | 0.041357 | HTR1E (margin: HTR1E) | 2.85 | 6.63e-03 | 3.19e-02 |
|  | 13 | 36788667 | 36789025 | PDFSTATSENSEMBLUCSC | 359 | 8 | 11667 | island | 0.648314 | 0.972219 | SOHLH2; CCDC169-SOHLH2 (margin: SOHLH2; CCDC169-SOHLH2) | 2.84 | 6.99e-03 | 3.19e-02 |
|  | 2 | 220378996 | 220379260 | PDFSTATSENSEMBLUCSC | 265 | 9 | 23036 | gene;island | 0.555074 | 0.950989 | ASIC4; AC053503.11 (margin: GMPPA; ASIC4; AC053503.11) | 2.84 | 1.06e-02 | 4.01e-02 |
|  | 8 | 65489863 | 65489939 | PDFSTATSENSEMBLUCSC | 77 | 5 | 39734 | tss;island | 0.546337 | 0.927886 | RP11-21C4.1 (margin: BHLHE22; RP11-21C4.5; RP11-21C4.1; CYP7B1) | 2.83 | 7.94e-03 | 3.19e-02 |
|  | 7 | 96653617 | 96654198 | PDFSTATSENSEMBLUCSC | 582 | 9 | 38696 | island | 0.551820 | 0.119323 | DLX5 (margin: DLX6-AS1; DLX5) | 2.83 | 1.06e-02 | 4.01e-02 |
|  | 17 | 36105064 | 36105517 | PDFSTATSENSEMBLUCSC | 454 | 7 | 18250 | island | 0.619792 | 0.972102 | RP11-115K3.1; HNF1B (margin: RP11-115K3.1; HNF1B) | 2.83 | 1.11e-02 | 4.14e-02 |
|  | 1 | 160039847 | 160040667 | PDFSTATSENSEMBLUCSC | 821 | 13 | 3887 | island | 0.389606 | 0.853883 | RP11-536C5.2; KCNJ10 (margin: RP11-536C5.2; KCNJ9; KCNJ10) | 2.83 | 6.28e-04 | 6.77e-03 |
|  | 10 | 27547536 | 27547965 | PDFSTATSENSEMBLUCSC | 430 | 6 | 5628 | island | 0.799525 | 0.977925 | ARMC4P1; LRRC37A6P (margin: RP11-85G18.6; ARMC4P1; LRRC37A6P) | 2.83 | 2.16e-03 | 1.45e-02 |
|  | 19 | 46526333 | 46526675 | PDFSTATSENSEMBLUCSC | 343 | 7 | 20889 | tss | 0.600238 | 0.940981 | CCDC61; PGLYRP1 (margin: CCDC61; MIR769; CTC-344H19.4; PGLYRP1) | 2.83 | 4.08e-03 | 2.47e-02 |
|  | 1 | 3104999 | 3105326 | PDFSTATSENSEMBLUCSC | 328 | 5 | 107 | gene;island | 0.682747 | 0.947706 | PRDM16 (margin: PRDM16) | 2.82 | 7.94e-03 | 3.19e-02 |
|  | 11 | 115630531 | 115631762 | PDFSTATSENSEMBLUCSC | 1232 | 14 | 8831 | island | 0.655795 | 0.971369 | AP000797.3; LINC00900 (margin: AP000797.3; LINC00900) | 2.82 | 1.31e-04 | 2.71e-03 |
|  | 11 | 14993378 | 14995322 | PDFSTATSENSEMBLUCSC | 1945 | 40 | 8081 | island | 0.551579 | 0.954245 | CALCB; CALCA (margin: CALCB; CALCA) | 2.82 | 5.34e-08 | 9.64e-06 |
|  | 7 | 92464881 | 92465342 | PDFSTATSENSEMBLUCSC | 462 | 8 | 37089 | gene;island | 0.010133 | 0.071381 | AC002454.1; CDK6 (margin: AC002454.1; CDK6) | 2.82 | 1.86e-03 | 1.45e-02 |
|  | 2 | 209271164 | 209272057 | PDFSTATSENSEMBLUCSC | 894 | 10 | 25050 | island | 0.796132 | 0.968239 | PTH2R (margin: PTH2R) | 2.80 | 2.06e-04 | 3.42e-03 |
|  | 2 | 239140340 | 239141043 | PDFSTATSENSEMBLUCSC | 704 | 9 | 23097 | gene | 0.558933 | 0.934166 | AC096574.4; AC016757.3 (margin: AC096574.4; AC016757.3; HES6; PER2) | 2.79 | 7.77e-03 | 3.19e-02 |
|  | 6 | 148663353 | 148663680 | PDFSTATSENSEMBLUCSC | 328 | 10 | 35280 | tss | 0.046244 | 0.271113 | SASH1 (margin: SASH1) | 2.79 | 1.15e-02 | 4.29e-02 |
|  | 3 | 68981852 | 68982098 | PDFSTATSENSEMBLUCSC | 247 | 9 | 28437 | tss;island | 0.752155 | 0.392432 | FAM19A4 (margin: FAM19A4) | 2.79 | 8.23e-05 | 2.06e-03 |
|  | 1 | 2984218 | 2984869 | PDFSTATSENSEMBLUCSC | 652 | 10 | 2528 | island | 0.884607 | 0.974694 | PRDM16; LINC00982 (margin: PRDM16; LINC00982; AL008733.1) | 2.79 | 2.06e-04 | 3.42e-03 |
|  | 1 | 101702519 | 101702782 | PDFSTATSENSEMBLUCSC | 264 | 5 | 654 | gene;island | 0.323418 | 0.765093 | S1PR1; RP4-575N6.4 (margin: S1PR1; RP4-575N6.5; snoU13; RP4-575N6.4) | 2.78 | 7.94e-03 | 3.19e-02 |
|  | 22 | 43505892 | 43506952 | PDFSTATSENSEMBLUCSC | 1061 | 27 | 27551 | island | 0.234504 | 0.027424 | BIK (margin: BIK) | 2.78 | 1.25e-02 | 4.66e-02 |
|  | 11 | 63803781 | 63804119 | PDFSTATSENSEMBLUCSC | 339 | 6 | 6659 | gene;island | 0.500129 | 0.908768 | RP11-21A7A.2; MACROD1 (margin: RP11-21A7A.2; MACROD1) | 2.77 | 8.66e-03 | 3.36e-02 |
|  | 6 | 32063394 | 32064258 | PDFSTATSENSEMBLUCSC | 865 | 28 | 34046 | gene;island | 0.783652 | 0.981094 | TNXB; ATF6B (margin: TNXB; ATF6B) | 2.77 | 2.28e-06 | 1.96e-04 |
|  | 17 | 79393463 | 79393718 | PDFSTATSENSEMBLUCSC | 256 | 5 | 16908 | gene;island | 0.516805 | 0.912538 | RP11-1055B8.7 (margin: RP11-1055B8.7) | 2.77 | 7.94e-03 | 3.19e-02 |
|  | 5 | 80256371 | 80256738 | PDFSTATSENSEMBLUCSC | 368 | 5 | 32925 | island | 0.777217 | 0.972353 | RASGRF2; CTC-459I6.1 (margin: RASGRF2; CTC-459I6.1) | 2.77 | 7.94e-03 | 3.19e-02 |
|  | 20 | 10199210 | 10199536 | PDFSTATSENSEMBLUCSC | 327 | 8 | 25999 | island | 0.041065 | 0.005844 | SNAP25; SNAP25-AS1 (margin: SNAP25; SNAP25-AS1) | 2.76 | 2.95e-03 | 1.88e-02 |
|  | 7 | 35292896 | 35293309 | PDFSTATSENSEMBLUCSC | 414 | 6 | 36963 | gene;island | 0.687405 | 0.919239 | TBX20 (margin: TBX20; AC009531.2) | 2.76 | 8.66e-03 | 3.36e-02 |
|  | 7 | 99517192 | 99517509 | PDFSTATSENSEMBLUCSC | 318 | 10 | 37762 | tss | 0.056448 | 0.337407 | TRIM4 (margin: RP4-604G5.1; TRIM4; GJC3) | 2.74 | 1.30e-04 | 2.71e-03 |
|  | 17 | 56832804 | 56833425 | PDFSTATSENSEMBLUCSC | 622 | 13 | 18605 | island | 0.805132 | 0.375615 | PPM1E (margin: PPM1E) | 2.74 | 1.29e-05 | 5.58e-04 |
|  | 17 | 56832804 | 56833201 | PDFSTATSENSEMBLUCSC | 398 | 11 | 17540 | tss | 0.807239 | 0.383988 | PPM1E (margin: PPM1E) | 2.74 | 8.51e-05 | 2.07e-03 |
|  | 1 | 114471711 | 114471974 | PDFSTATSENSEMBLUCSC | 264 | 9 | 1847 | tss | 0.003018 | 0.024771 | HIPK1; RP5-1073O3.7 (margin: HIPK1; RP5-1073O3.7) | 2.74 | 1.06e-02 | 4.01e-02 |
|  | 20 | 57089933 | 57090317 | PDFSTATSENSEMBLUCSC | 385 | 10 | 26291 | island | 0.726587 | 0.919282 | APCDD1L-AS1; APCDD1L (margin: APCDD1L-AS1; APCDD1L) | 2.72 | 1.15e-02 | 4.29e-02 |
|  | 5 | 43396802 | 43397288 | PDFSTATSENSEMBLUCSC | 487 | 7 | 31603 | gene;island | 0.817542 | 0.980732 | CCL28 (margin: CCL28) | 2.72 | 6.99e-03 | 3.19e-02 |
|  | 20 | 61583537 | 61583987 | PDFSTATSENSEMBLUCSC | 451 | 8 | 25886 | tss | 0.663194 | 0.947722 | SLC17A9 (margin: GID8; SLC17A9) | 2.72 | 6.99e-03 | 3.19e-02 |
|  | 10 | 126138593 | 126138879 | PDFSTATSENSEMBLUCSC | 287 | 6 | 5422 | tss | 0.107762 | 0.463447 | RP13-238F13.3; NKX1-2 (margin: RP13-238F13.3; LHPP; RP13-238F13.5; NKX1-2) | 2.72 | 2.16e-03 | 1.45e-02 |
|  | 17 | 3438857 | 3439372 | PDFSTATSENSEMBLUCSC | 516 | 7 | 16335 | gene;island | 0.901378 | 0.988272 | TRPV3 (margin: TRPV3) | 2.71 | 6.99e-03 | 3.19e-02 |
|  | 1 | 109656708 | 109657035 | PDFSTATSENSEMBLUCSC | 328 | 5 | 665 | gene;tss;island | 0.357968 | 0.048760 | KIAA1324; C1orf194 (margin: KIAA1324; RP5-1065J22.4; C1orf194) | 2.71 | 7.94e-03 | 3.19e-02 |
|  | 5 | 178017260 | 178018186 | PDFSTATSENSEMBLUCSC | 927 | 13 | 33471 | island | 0.679628 | 0.926302 | COL23A1 (margin: COL23A1; CLK4) | 2.70 | 1.20e-02 | 4.46e-02 |
|  | 17 | 79304498 | 79304683 | PDFSTATSENSEMBLUCSC | 186 | 7 | 17737 | tss | 0.910714 | 0.650869 | TMEM105 (margin: TMEM105) | 2.70 | 5.83e-04 | 6.47e-03 |
|  | 19 | 40732691 | 40732902 | PDFSTATSENSEMBLUCSC | 212 | 8 | 20784 | tss | 0.416655 | 0.097025 | CNTD2 (margin: MAP3K10; TTC9B; CNTD2; AKT2) | 2.70 | 1.55e-04 | 2.71e-03 |
|  | 1 | 209979111 | 209979779 | PDFSTATSENSEMBLUCSC | 669 | 13 | 4167 | island | 0.056728 | 0.009393 | IRF6 (margin: IRF6) | 2.70 | 3.49e-03 | 2.20e-02 |
|  | 16 | 10275767 | 10276119 | PDFSTATSENSEMBLUCSC | 353 | 5 | 14658 | gene;island | 0.119076 | 0.464712 | GRIN2A (margin: GRIN2A) | 2.69 | 7.94e-03 | 3.19e-02 |
|  | 7 | 44365289 | 44365627 | PDFSTATSENSEMBLUCSC | 339 | 8 | 37567 | tss;island | 0.154695 | 0.032935 | CAMK2B (margin: CAMK2B) | 2.69 | 6.99e-03 | 3.19e-02 |
|  | 1 | 2984245 | 2984869 | PDFSTATSENSEMBLUCSC | 625 | 9 | 1178 | tss | 0.904245 | 0.974739 | PRDM16; LINC00982 (margin: PRDM16; LINC00982; AL008733.1) | 2.69 | 7.82e-04 | 8.17e-03 |
|  | 8 | 101661918 | 101662246 | PDFSTATSENSEMBLUCSC | 329 | 7 | 39844 | tss | 0.895678 | 0.975119 | SNX31 (margin: SNX31) | 2.69 | 1.11e-02 | 4.14e-02 |
|  | 7 | 93204959 | 93205295 | PDFSTATSENSEMBLUCSC | 337 | 11 | 37714 | tss;island | 0.025972 | 0.154391 | CALCR (margin: CALCR) | 2.69 | 1.90e-04 | 3.21e-03 |
|  | 14 | 70655686 | 70656116 | PDFSTATSENSEMBLUCSC | 431 | 9 | 12976 | island | 0.620561 | 0.207382 | RP11-486O13.2; SLC8A3 (margin: RP11-486O13.2; SLC8A3) | 2.69 | 1.85e-03 | 1.45e-02 |
|  | 1 | 146556239 | 146556859 | PDFSTATSENSEMBLUCSC | 621 | 5 | 3691 | island | 0.111727 | 0.450950 | NBPF13P; RP11-325P15.2 (margin: RNVU1-8; NBPF13P; RP11-325P15.2) | 2.69 | 7.94e-03 | 3.19e-02 |
|  | 13 | 36872048 | 36872346 | PDFSTATSENSEMBLUCSC | 299 | 8 | 11393 | tss | 0.439499 | 0.731273 | SOHLH2; CCDC169-SOHLH2; CCDC169 (margin: SOHLH2; CCDC169-SOHLH2; CCDC169; SPG20) | 2.68 | 2.95e-03 | 1.88e-02 |
|  | 6 | 31831340 | 31831890 | PDFSTATSENSEMBLUCSC | 551 | 16 | 33975 | gene;tss;island | 0.083374 | 0.011966 | NEU1; SLC44A4 (margin: NEU1; SLC44A4) | 2.68 | 7.70e-04 | 8.17e-03 |
|  | 11 | 17373103 | 17373302 | PDFSTATSENSEMBLUCSC | 200 | 6 | 7174 | tss;island | 0.098264 | 0.018108 | NUCB2; NCR3LG1 (margin: NUCB2; NCR3LG1) | 2.67 | 4.33e-03 | 2.52e-02 |
|  | 1 | 47696212 | 47696788 | PDFSTATSENSEMBLUCSC | 577 | 6 | 1607 | tss;island | 0.855688 | 0.978024 | RP1-18D14.7; TAL1 (margin: RP1-18D14.7; TAL1) | 2.67 | 4.33e-03 | 2.52e-02 |
|  | 12 | 133532874 | 133533354 | PDFSTATSENSEMBLUCSC | 481 | 7 | 10135 | tss;island | 0.013801 | 0.203317 | CHFR; ZNF605 (margin: CHFR; ZNF605) | 2.67 | 6.99e-03 | 3.19e-02 |
|  | 1 | 78511140 | 78512129 | PDFSTATSENSEMBLUCSC | 990 | 12 | 3402 | island | 0.860338 | 0.980520 | GIPC2; RP11-386I14.2 (margin: GIPC2; RP11-386I14.2) | 2.66 | 3.71e-04 | 5.59e-03 |
|  | 8 | 27468981 | 27469338 | PDFSTATSENSEMBLUCSC | 358 | 5 | 39257 | gene;tss;island | 0.820086 | 0.969287 | CLU (margin: CLU) | 2.66 | 7.94e-03 | 3.19e-02 |
|  | 14 | 101526485 | 101527144 | PDFSTATSENSEMBLUCSC | 660 | 10 | 12631 | tss | 0.600021 | 0.900026 | MIR154; MIR496; MIR377 (margin: MIR381HG; MIR544A; MIR655; AL132709.3; MIR487A; MIR134; MIR485; MIR323B; MIR154; MIR496; MIR377; MIR541; MIR409; MIR412; MIR369; MIR410; MIR656; MEG9; MIR382) | 2.66 | 1.15e-02 | 4.29e-02 |
|  | 14 | 96505296 | 96505874 | PDFSTATSENSEMBLUCSC | 579 | 12 | 13114 | island | 0.130796 | 0.018927 | C14orf132 (margin: C14orf132) | 2.66 | 4.96e-04 | 6.47e-03 |
|  | 12 | 57588031 | 57588350 | PDFSTATSENSEMBLUCSC | 320 | 8 | 9187 | gene | 0.970962 | 0.806002 | LRP1; MIR1228 (margin: LRP1; MIR1228) | 2.66 | 1.86e-03 | 1.45e-02 |
|  | 3 | 42947263 | 42947565 | PDFSTATSENSEMBLUCSC | 303 | 5 | 28250 | tss | 0.907377 | 0.987141 | ACKR2; KRBOX1; ZNF662 (margin: ACKR2; KRBOX1; ZNF662) | 2.66 | 7.94e-03 | 3.19e-02 |
|  | 4 | 76555772 | 76556042 | PDFSTATSENSEMBLUCSC | 271 | 9 | 30439 | tss | 0.115174 | 0.453354 | CDKL2 (margin: CDKL2; G3BP2) | 2.66 | 4.11e-05 | 1.17e-03 |
|  | 12 | 49740639 | 49740996 | PDFSTATSENSEMBLUCSC | 358 | 10 | 9687 | tss | 0.085685 | 0.017948 | DNAJC22 (margin: DNAJC22; C1QL4) | 2.65 | 6.84e-03 | 3.19e-02 |
|  | 12 | 49740639 | 49741245 | PDFSTATSENSEMBLUCSC | 607 | 12 | 10392 | island | 0.085685 | 0.021227 | DNAJC22 (margin: DNAJC22; C1QL4) | 2.65 | 8.29e-03 | 3.33e-02 |
|  | 5 | 169532686 | 169533120 | PDFSTATSENSEMBLUCSC | 435 | 8 | 33376 | island | 0.170298 | 0.636744 | FOXI1 (margin: FOXI1) | 2.65 | 4.66e-03 | 2.68e-02 |
|  | 1 | 45792540 | 45792733 | PDFSTATSENSEMBLUCSC | 194 | 7 | 3198 | island | 0.375137 | 0.113030 | HPDL (margin: HPDL; MUTYH) | 2.65 | 5.83e-04 | 6.47e-03 |
|  | 4 | 105411974 | 105412536 | PDFSTATSENSEMBLUCSC | 563 | 8 | 31218 | island | 0.135523 | 0.027622 | AC093628.1; AC004053.1; CXXC4 (margin: AC093628.1; AC004053.1; CXXC4) | 2.65 | 1.55e-04 | 2.71e-03 |
|  | 1 | 177150789 | 177151164 | PDFSTATSENSEMBLUCSC | 376 | 5 | 898 | gene;island | 0.329806 | 0.064670 | FAM5B (margin: FAM5B; AL021398.1) | 2.65 | 7.94e-03 | 3.19e-02 |
|  | 3 | 25469392 | 25469720 | PDFSTATSENSEMBLUCSC | 329 | 8 | 28190 | tss | 0.155647 | 0.547067 | RARB (margin: RARB) | 2.64 | 2.95e-03 | 1.88e-02 |
|  | 6 | 133561614 | 133562494 | PDFSTATSENSEMBLUCSC | 881 | 38 | 36486 | island | 0.829707 | 0.971963 | EYA4 (margin: EYA4) | 2.64 | 1.52e-08 | 3.90e-06 |
|  | 4 | 154680808 | 154681323 | PDFSTATSENSEMBLUCSC | 516 | 6 | 30155 | gene;island | 0.885930 | 0.975538 | RNF175 (margin: RNF175) | 2.63 | 2.16e-03 | 1.45e-02 |
|  | 10 | 102279690 | 102279954 | PDFSTATSENSEMBLUCSC | 265 | 7 | 5294 | tss;island | 0.989507 | 0.955458 | SEC31B; NDUFB8 (margin: HIF1AN; SEC31B; NDUFB8) | 2.62 | 1.11e-02 | 4.14e-02 |
|  | 5 | 2112109 | 2112561 | PDFSTATSENSEMBLUCSC | 453 | 7 | 32652 | island | 0.640088 | 0.934477 | (margin: ) | 2.62 | 4.08e-03 | 2.47e-02 |
|  | 15 | 40762807 | 40763522 | PDFSTATSENSEMBLUCSC | 716 | 10 | 14038 | island | 0.010622 | 0.053347 | CHST14 (margin: BAHD1; CHST14; RP11-64K12.8; RP11-64K12.1) | 2.62 | 3.89e-03 | 2.43e-02 |
|  | 6 | 133561614 | 133562492 | PDFSTATSENSEMBLUCSC | 879 | 37 | 35231 | tss | 0.813407 | 0.970430 | EYA4 (margin: EYA4) | 2.61 | 2.76e-08 | 6.41e-06 |
|  | 19 | 10046864 | 10047420 | PDFSTATSENSEMBLUCSC | 557 | 8 | 21511 | island | 0.096399 | 0.021051 | OLFM2 (margin: OLFM2) | 2.60 | 3.11e-04 | 4.77e-03 |
|  | 19 | 11689488 | 11689997 | PDFSTATSENSEMBLUCSC | 510 | 12 | 20477 | tss;island | 0.876010 | 0.981594 | ZNF627; ACP5 (margin: ZNF627; AC020947.2; ACP5) | 2.60 | 1.11e-03 | 1.12e-02 |
|  | 5 | 178450457 | 178450763 | PDFSTATSENSEMBLUCSC | 307 | 6 | 32547 | tss | 0.074159 | 0.305924 | ZNF879 (margin: ZNF879) | 2.60 | 2.16e-03 | 1.45e-02 |
|  | 10 | 61122358 | 61122716 | PDFSTATSENSEMBLUCSC | 359 | 8 | 5093 | tss | 0.071865 | 0.006552 | FAM13C (margin: FAM13C) | 2.60 | 1.04e-02 | 3.96e-02 |
|  | 16 | 56622951 | 56623215 | PDFSTATSENSEMBLUCSC | 265 | 6 | 15246 | tss | 0.908559 | 0.983984 | MT3 (margin: MT3) | 2.60 | 2.16e-03 | 1.45e-02 |
|  | 1 | 45792557 | 45792733 | PDFSTATSENSEMBLUCSC | 177 | 5 | 501 | gene | 0.434480 | 0.116702 | HPDL (margin: HPDL; MUTYH) | 2.60 | 7.94e-03 | 3.19e-02 |
|  | 14 | 96505296 | 96505636 | PDFSTATSENSEMBLUCSC | 341 | 9 | 12591 | tss | 0.124201 | 0.022925 | C14orf132 (margin: C14orf132) | 2.59 | 1.06e-02 | 4.01e-02 |
|  | 2 | 80531483 | 80531898 | PDFSTATSENSEMBLUCSC | 416 | 10 | 22756 | gene;island | 0.828147 | 0.453306 | CTNNA2; LRRTM1 (margin: CTNNA2; LRRTM1) | 2.59 | 2.06e-04 | 3.42e-03 |
|  | 8 | 55370192 | 55370434 | PDFSTATSENSEMBLUCSC | 243 | 8 | 39706 | tss | 0.776897 | 0.962341 | SOX17 (margin: SOX17) | 2.59 | 1.04e-02 | 3.96e-02 |
|  | 11 | 32454718 | 32455735 | PDFSTATSENSEMBLUCSC | 1018 | 12 | 6567 | gene;island | 0.566199 | 0.958000 | WT1-AS; WT1 (margin: WT1-AS; WT1) | 2.57 | 2.32e-03 | 1.52e-02 |
|  | 1 | 200011684 | 200011988 | PDFSTATSENSEMBLUCSC | 305 | 6 | 934 | gene;island | 0.913987 | 0.977029 | NR5A2 (margin: NR5A2; RNU6-570P) | 2.57 | 4.33e-03 | 2.52e-02 |
|  | 20 | 10199210 | 10199473 | PDFSTATSENSEMBLUCSC | 264 | 7 | 25595 | tss | 0.040608 | 0.007249 | SNAP25; SNAP25-AS1 (margin: SNAP25; SNAP25-AS1) | 2.57 | 1.11e-02 | 4.14e-02 |
|  | 19 | 40732518 | 40732902 | PDFSTATSENSEMBLUCSC | 385 | 9 | 22017 | island | 0.410154 | 0.098825 | CNTD2 (margin: MAP3K10; TTC9B; CNTD2; AKT2) | 2.57 | 5.64e-03 | 3.18e-02 |
|  | 1 | 114471711 | 114472148 | PDFSTATSENSEMBLUCSC | 438 | 11 | 3619 | island | 0.005606 | 0.029867 | HIPK1; RP5-1073O3.7 (margin: HIPK1; RP5-1073O3.7) | 2.57 | 1.04e-02 | 3.96e-02 |
|  | 2 | 24232603 | 24232974 | PDFSTATSENSEMBLUCSC | 372 | 11 | 24125 | island | 0.918705 | 0.984519 | MFSD2B (margin: UBXN2A; MFSD2B) | 2.56 | 5.53e-04 | 6.47e-03 |
|  | 11 | 2920052 | 2920819 | PDFSTATSENSEMBLUCSC | 768 | 16 | 6469 | gene;tss;island | 0.394485 | 0.799497 | SLC22A18; SLC22A18AS (margin: SLC22A18; SLC22A18AS) | 2.56 | 3.92e-03 | 2.46e-02 |
|  | 6 | 69345836 | 69346248 | PDFSTATSENSEMBLUCSC | 413 | 5 | 34379 | gene;island | 0.480994 | 0.834948 | BAI3; RP3-525N10.2 (margin: BAI3; RP3-525N10.2) | 2.56 | 7.94e-03 | 3.19e-02 |
|  | 2 | 24232603 | 24232891 | PDFSTATSENSEMBLUCSC | 289 | 10 | 23220 | tss | 0.923183 | 0.985503 | MFSD2B (margin: UBXN2A; MFSD2B) | 2.56 | 1.50e-03 | 1.41e-02 |
|  | 14 | 65016517 | 65016602 | PDFSTATSENSEMBLUCSC | 86 | 5 | 12460 | tss;island | 0.927390 | 0.638439 | PPP1R36 (margin: HSPA2; PPP1R36; RP11-973N13.4; RP11-973N13.3) | 2.55 | 7.94e-03 | 3.19e-02 |
|  | 1 | 209979487 | 209979779 | PDFSTATSENSEMBLUCSC | 293 | 8 | 2233 | tss | 0.074692 | 0.010071 | IRF6 (margin: IRF6) | 2.55 | 6.99e-03 | 3.19e-02 |
|  | 14 | 85996101 | 85996499 | PDFSTATSENSEMBLUCSC | 399 | 10 | 13053 | island | 0.217010 | 0.580131 | RP11-497E19.2; FLRT2; RP11-497E19.1 (margin: RP11-497E19.2; FLRT2; RP11-497E19.1) | 2.55 | 2.09e-03 | 1.45e-02 |
|  | 12 | 58012960 | 58013687 | PDFSTATSENSEMBLUCSC | 728 | 20 | 9822 | tss;island | 0.714889 | 0.966286 | ARHGEF25; SLC26A10; AC025165.8 (margin: DTX3; ARHGEF25; SLC26A10; AC025165.8; B4GALNT1) | 2.54 | 3.45e-06 | 2.32e-04 |
|  | 1 | 156646293 | 156646746 | PDFSTATSENSEMBLUCSC | 454 | 5 | 827 | gene;island | 0.931531 | 0.984801 | NES (margin: NES; RP11-66D17.3) | 2.54 | 7.94e-03 | 3.19e-02 |
|  | 6 | 106959535 | 106959651 | PDFSTATSENSEMBLUCSC | 117 | 6 | 35151 | tss;island | 0.015386 | 0.077110 | AIM1 (margin: AIM1) | 2.54 | 4.33e-03 | 2.52e-02 |
|  | 20 | 61583537 | 61584159 | PDFSTATSENSEMBLUCSC | 623 | 11 | 26338 | island | 0.749194 | 0.956542 | SLC17A9 (margin: GID8; SLC17A9) | 2.54 | 1.86e-03 | 1.45e-02 |
|  | 6 | 26183508 | 26184245 | PDFSTATSENSEMBLUCSC | 738 | 12 | 35521 | island | 0.158193 | 0.459467 | HIST1H2BE (margin: HIST1H2BD; HIST1H2BE; HIST1H1PS1; HIST1H4D) | 2.53 | 8.29e-03 | 3.33e-02 |
|  | 7 | 151136940 | 151137192 | PDFSTATSENSEMBLUCSC | 253 | 5 | 39040 | island | 0.862262 | 0.946307 | RP4-555L14.4; CRYGN (margin: RP4-555L14.4; CRYGN; MIR3907) | 2.53 | 7.94e-03 | 3.19e-02 |
|  | 10 | 82168043 | 82168295 | PDFSTATSENSEMBLUCSC | 253 | 6 | 5890 | island | 0.036516 | 0.006279 | FAM213A (margin: FAM213A) | 2.52 | 8.66e-03 | 3.36e-02 |
|  | 3 | 71833543 | 71833907 | PDFSTATSENSEMBLUCSC | 365 | 5 | 27860 | gene;island | 0.819900 | 0.965387 | PROK2 (margin: PROK2) | 2.52 | 7.94e-03 | 3.19e-02 |
|  | 14 | 101521409 | 101521809 | PDFSTATSENSEMBLUCSC | 401 | 10 | 12629 | tss | 0.495308 | 0.865412 | MIR134; MIR485; MIR323B; MIR382 (margin: MIR1185-2; MIR381HG; MIR381; MIR487B; MIR539; MIR889; MIR544A; MIR655; AL132709.3; MIR487A; MIR134; MIR485; MIR323B; MIR154; MIR496; MIR377; MIR541; MIR409; MIR412; MIR369; MIR410; MIR656; MIR382) | 2.52 | 4.87e-04 | 6.47e-03 |
|  | 3 | 49057661 | 49058586 | PDFSTATSENSEMBLUCSC | 926 | 16 | 27776 | gene;tss;island | 0.005954 | 0.039678 | NDUFAF3; DALRD3; MIR425; MIR191 (margin: WDR6; NDUFAF3; RP13-131K19.6; DALRD3; MIR425; MIR191; IMPDH2; QRICH1) | 2.52 | 6.61e-03 | 3.19e-02 |
|  | 1 | 40782713 | 40783264 | PDFSTATSENSEMBLUCSC | 552 | 10 | 3120 | island | 0.896823 | 0.978132 | COL9A2 (margin: COL9A2) | 2.51 | 7.58e-05 | 1.98e-03 |
|  | 16 | 67678593 | 67678846 | PDFSTATSENSEMBLUCSC | 254 | 5 | 15307 | tss;island | 0.891240 | 0.974688 | RLTPR (margin: CTCF; RLTPR) | 2.49 | 7.94e-03 | 3.19e-02 |
|  | 6 | 32728786 | 32730299 | PDFSTATSENSEMBLUCSC | 1514 | 40 | 34132 | gene;island | 0.744104 | 0.943447 | HLA-DQB2 (margin: MIR3135B; HLA-DQB2) | 2.49 | 3.94e-04 | 5.86e-03 |
|  | 1 | 179711859 | 179712281 | PDFSTATSENSEMBLUCSC | 423 | 8 | 2120 | tss | 0.767768 | 0.946841 | RP11-12M5.1; FAM163A (margin: RP11-12M5.1; FAM163A) | 2.49 | 1.09e-03 | 1.10e-02 |
|  | 19 | 12946231 | 12946465 | PDFSTATSENSEMBLUCSC | 235 | 6 | 20515 | tss | 0.350878 | 0.112208 | MAST1; HOOK2; RTBDN (margin: CTD-2265O21.3; MAST1; HOOK2; RTBDN) | 2.49 | 8.66e-03 | 3.36e-02 |
|  | 3 | 111717534 | 111717969 | PDFSTATSENSEMBLUCSC | 436 | 7 | 28498 | tss | 0.548037 | 0.827476 | TAGLN3 (margin: ABHD10; TAGLN3) | 2.48 | 5.83e-04 | 6.47e-03 |
|  | 1 | 31845923 | 31846110 | PDFSTATSENSEMBLUCSC | 188 | 6 | 1445 | tss | 0.899896 | 0.973886 | FABP3 (margin: ZCCHC17; FABP3) | 2.48 | 2.16e-03 | 1.45e-02 |
|  | 8 | 85096835 | 85097056 | PDFSTATSENSEMBLUCSC | 222 | 5 | 39789 | tss | 0.703274 | 0.444129 | RALYL (margin: RALYL) | 2.48 | 7.94e-03 | 3.19e-02 |
|  | 6 | 130686592 | 130686865 | PDFSTATSENSEMBLUCSC | 274 | 5 | 36477 | island | 0.880337 | 0.981155 | TMEM200A; SAMD3 (margin: TMEM200A; SAMD3) | 2.47 | 7.94e-03 | 3.19e-02 |
|  | 8 | 143459774 | 143460209 | PDFSTATSENSEMBLUCSC | 436 | 6 | 39462 | gene | 0.863331 | 0.967512 | TSNARE1 (margin: TSNARE1) | 2.47 | 4.33e-03 | 2.52e-02 |
|  | 17 | 35290307 | 35291580 | PDFSTATSENSEMBLUCSC | 1274 | 12 | 18235 | island | 0.896785 | 0.980525 | RP11-445F12.1 (margin: LHX1; RP11-445F12.1; RP11-445F12.2) | 2.47 | 1.11e-03 | 1.12e-02 |
|  | 10 | 126138415 | 126138879 | PDFSTATSENSEMBLUCSC | 465 | 7 | 6228 | island | 0.147673 | 0.487684 | RP13-238F13.3; NKX1-2 (margin: RP13-238F13.3; LHPP; RP13-238F13.5; NKX1-2) | 2.47 | 5.83e-04 | 6.47e-03 |
|  | 13 | 37004536 | 37005582 | PDFSTATSENSEMBLUCSC | 1047 | 29 | 11396 | tss;island | 0.745720 | 0.934621 | CCNA1 (margin: CCNA1) | 2.46 | 2.32e-04 | 3.81e-03 |
|  | 4 | 123747447 | 123747750 | PDFSTATSENSEMBLUCSC | 304 | 7 | 30581 | tss;island | 0.919190 | 0.979003 | FGF2; AC021205.1 (margin: FGF2; AC021205.1; RP11-170N16.1) | 2.46 | 6.99e-03 | 3.19e-02 |
|  | 12 | 57588031 | 57588243 | PDFSTATSENSEMBLUCSC | 213 | 7 | 9811 | tss | 0.972239 | 0.832512 | LRP1; MIR1228 (margin: LRP1; MIR1228) | 2.46 | 2.33e-03 | 1.52e-02 |
|  | 4 | 154605087 | 154605468 | PDFSTATSENSEMBLUCSC | 382 | 5 | 31375 | island | 0.536941 | 0.157156 | (margin: RP11-153M7.3) | 2.46 | 7.94e-03 | 3.19e-02 |
|  | 14 | 101531403 | 101532352 | PDFSTATSENSEMBLUCSC | 950 | 20 | 12634 | tss;island | 0.565064 | 0.922784 | MIR541; MIR409; MIR412; MIR369; MIR410; MIR656 (margin: MIR134; MIR485; MIR323B; MIR154; MIR496; MIR377; MIR541; MIR409; MIR412; MIR369; MIR410; MIR656; MEG9; MIR382; AL132709.1) | 2.45 | 5.13e-03 | 2.94e-02 |
|  | 4 | 111544235 | 111544463 | PDFSTATSENSEMBLUCSC | 229 | 8 | 30095 | gene;island | 0.038634 | 0.007945 | PITX2 (margin: RP11-380D23.2; PITX2) | 2.45 | 6.99e-03 | 3.19e-02 |
|  | 10 | 105036611 | 105036863 | PDFSTATSENSEMBLUCSC | 253 | 7 | 5334 | tss;island | 0.949957 | 0.986519 | INA (margin: INA) | 2.45 | 6.99e-03 | 3.19e-02 |
|  | 3 | 169530268 | 169530621 | PDFSTATSENSEMBLUCSC | 354 | 6 | 29650 | island | 0.597743 | 0.911782 | LRRC34 (margin: LRRIQ4; LRRC34) | 2.44 | 2.16e-03 | 1.45e-02 |
|  | 4 | 57410776 | 57411219 | PDFSTATSENSEMBLUCSC | 444 | 5 | 31069 | island | 0.371883 | 0.112490 | THEGL (margin: THEGL) | 2.44 | 7.94e-03 | 3.19e-02 |
|  | 10 | 130339134 | 130339927 | PDFSTATSENSEMBLUCSC | 794 | 12 | 6256 | island | 0.886813 | 0.030842 | (margin: ) | 2.44 | 1.43e-03 | 1.36e-02 |
|  | 2 | 198669113 | 198669339 | PDFSTATSENSEMBLUCSC | 227 | 6 | 23804 | tss;island | 0.122686 | 0.014745 | PLCL1 (margin: PLCL1) | 2.44 | 4.33e-03 | 2.52e-02 |
|  | 17 | 46655164 | 46655736 | PDFSTATSENSEMBLUCSC | 573 | 11 | 16653 | gene | 0.843091 | 0.977841 | HOXB-AS3; HOXB3; HOXB4; MIR10A (margin: HOXB-AS3; HOXB3; HOXB4; MIR10A) | 2.44 | 1.28e-04 | 2.71e-03 |
|  | 2 | 223162666 | 223163326 | PDFSTATSENSEMBLUCSC | 661 | 9 | 23047 | gene;island | 0.810193 | 0.979164 | CCDC140; PAX3 (margin: CCDC140; PAX3) | 2.43 | 3.99e-03 | 2.47e-02 |
|  | 13 | 95364333 | 95364675 | PDFSTATSENSEMBLUCSC | 343 | 7 | 11822 | island | 0.619329 | 0.908187 | SOX21-AS1; SOX21 (margin: SOX21-AS1; LINC00391; SOX21) | 2.43 | 6.99e-03 | 3.19e-02 |
|  | 1 | 40782991 | 40783264 | PDFSTATSENSEMBLUCSC | 274 | 7 | 1535 | tss | 0.900811 | 0.977509 | COL9A2 (margin: COL9A2) | 2.43 | 4.08e-03 | 2.47e-02 |
|  | 10 | 102893925 | 102894639 | PDFSTATSENSEMBLUCSC | 715 | 8 | 4724 | gene;island | 0.901336 | 0.977732 | TLX1; RP11-31L23.3 (margin: HUG1; TLX1; TLX1NB; RP11-31L23.3) | 2.43 | 1.09e-03 | 1.10e-02 |
|  | 2 | 239139911 | 239141043 | PDFSTATSENSEMBLUCSC | 1133 | 17 | 23972 | tss;island | 0.764399 | 0.969619 | AC096574.4; AC016757.3 (margin: AC096574.4; AC016757.3; HES6; PER2) | 2.42 | 1.00e-03 | 1.04e-02 |
|  | 17 | 15847977 | 15848264 | PDFSTATSENSEMBLUCSC | 288 | 9 | 18045 | island | 0.052174 | 0.016739 | ADORA2B (margin: ADORA2B) | 2.42 | 1.06e-02 | 4.01e-02 |
|  | 2 | 80531500 | 80531898 | PDFSTATSENSEMBLUCSC | 399 | 9 | 23473 | tss | 0.805138 | 0.459509 | CTNNA2; LRRTM1 (margin: CTNNA2; LRRTM1) | 2.42 | 7.82e-04 | 8.17e-03 |
|  | 19 | 52391234 | 52391789 | PDFSTATSENSEMBLUCSC | 556 | 10 | 20999 | tss | 0.964228 | 0.793461 | CTC-429C10.2; ZNF577; ZNF649 (margin: CTC-429C10.2; ZNF577; ZNF649) | 2.41 | 5.20e-03 | 2.96e-02 |
|  | 6 | 160679391 | 160680213 | PDFSTATSENSEMBLUCSC | 823 | 12 | 36612 | island | 0.527601 | 0.912152 | SLC22A2 (margin: SLC22A2) | 2.41 | 1.00e-02 | 3.88e-02 |
|  | 2 | 217236425 | 217237043 | PDFSTATSENSEMBLUCSC | 619 | 14 | 25072 | island | 0.246139 | 0.052272 | MARCH4 (margin: MARCH4) | 2.41 | 6.74e-03 | 3.19e-02 |
|  | 11 | 32457124 | 32457386 | PDFSTATSENSEMBLUCSC | 263 | 7 | 7226 | tss;island | 0.816882 | 0.962981 | WT1-AS; WT1 (margin: WT1-AS; WT1) | 2.40 | 1.11e-02 | 4.14e-02 |
|  | 6 | 49518347 | 49518627 | PDFSTATSENSEMBLUCSC | 281 | 5 | 34363 | gene;island | 0.052299 | 0.010456 | C6orf141 (margin: C6orf141) | 2.40 | 7.94e-03 | 3.19e-02 |
|  | 2 | 242808427 | 242808907 | PDFSTATSENSEMBLUCSC | 481 | 5 | 25312 | island | 0.483445 | 0.904697 | (margin: CXXC11; PDCD1) | 2.40 | 7.94e-03 | 3.19e-02 |
|  | 7 | 4210949 | 4211755 | PDFSTATSENSEMBLUCSC | 807 | 8 | 36831 | gene | 0.704586 | 0.919150 | SDK1 (margin: SDK1) | 2.40 | 1.86e-03 | 1.45e-02 |
|  | 7 | 115850376 | 115850450 | PDFSTATSENSEMBLUCSC | 75 | 5 | 37848 | tss;island | 0.009799 | 0.050344 | TES (margin: TES) | 2.40 | 7.94e-03 | 3.19e-02 |
|  | 8 | 41167660 | 41168481 | PDFSTATSENSEMBLUCSC | 822 | 12 | 39668 | tss;island | 0.618888 | 0.926521 | SFRP1 (margin: RNU6-895P; SFRP1) | 2.39 | 1.11e-03 | 1.12e-02 |
|  | 12 | 53613892 | 53614284 | PDFSTATSENSEMBLUCSC | 393 | 7 | 9148 | gene;island | 0.796755 | 0.960686 | RARG (margin: RARG) | 2.39 | 6.99e-03 | 3.19e-02 |
|  | 8 | 109799518 | 109799909 | PDFSTATSENSEMBLUCSC | 392 | 5 | 40534 | island | 0.567192 | 0.129559 | TMEM74 (margin: TMEM74) | 2.38 | 7.94e-03 | 3.19e-02 |
|  | 1 | 113051685 | 113052167 | PDFSTATSENSEMBLUCSC | 483 | 6 | 691 | gene;island | 0.615342 | 0.874311 | WNT2B (margin: WNT2B) | 2.38 | 8.66e-03 | 3.36e-02 |
|  | 11 | 61584319 | 61584773 | PDFSTATSENSEMBLUCSC | 455 | 8 | 8335 | island | 0.507546 | 0.163056 | FADS2; FADS1; MIR1908 (margin: FADS2; FADS1; MIR1908) | 2.37 | 1.86e-03 | 1.45e-02 |
|  | 11 | 61584616 | 61584773 | PDFSTATSENSEMBLUCSC | 158 | 6 | 7341 | tss | 0.520046 | 0.163056 | FADS2; FADS1; MIR1908 (margin: FADS2; FADS1; MIR1908) | 2.37 | 8.66e-03 | 3.36e-02 |
|  | 15 | 26108612 | 26108948 | PDFSTATSENSEMBLUCSC | 337 | 12 | 13539 | tss;island | 0.751221 | 0.956845 | RP11-2C7.1; ATP10A (margin: RP11-2C7.1; ATP10A) | 2.36 | 4.51e-03 | 2.62e-02 |
|  | 17 | 72357992 | 72358370 | PDFSTATSENSEMBLUCSC | 379 | 5 | 17624 | tss;island | 0.314482 | 0.681140 | BTBD17 (margin: KIF19; GPR142; AC103809.2; BTBD17) | 2.35 | 7.94e-03 | 3.19e-02 |
|  | 6 | 32551749 | 32552670 | PDFSTATSENSEMBLUCSC | 922 | 17 | 34122 | gene;island | 0.844318 | 0.411100 | HLA-DRB1 (margin: HLA-DRB1) | 2.35 | 8.51e-03 | 3.36e-02 |
|  | 6 | 100050480 | 100051116 | PDFSTATSENSEMBLUCSC | 637 | 8 | 36356 | island | 0.888308 | 0.965139 | (margin: PRDM13) | 2.35 | 6.99e-03 | 3.19e-02 |
|  | 17 | 48911983 | 48912556 | PDFSTATSENSEMBLUCSC | 574 | 12 | 17508 | tss | 0.843804 | 0.954189 | WFIKKN2 (margin: WFIKKN2; RP11-506D12.5) | 2.34 | 1.03e-04 | 2.47e-03 |
|  | 2 | 102091195 | 102091321 | PDFSTATSENSEMBLUCSC | 127 | 6 | 23548 | tss | 0.767366 | 0.937072 | RFX8 (margin: AC092570.2; RFX8) | 2.34 | 2.16e-03 | 1.45e-02 |
|  | 4 | 156680007 | 156680518 | PDFSTATSENSEMBLUCSC | 512 | 7 | 31392 | island | 0.837231 | 0.448121 | GUCY1B3 (margin: GUCY1B3) | 2.34 | 5.83e-04 | 6.47e-03 |
|  | 6 | 32805142 | 32806489 | PDFSTATSENSEMBLUCSC | 1348 | 25 | 34146 | gene | 0.011911 | 0.065753 | TAP2 (margin: XXbac-BPG246D15.8; PSMB9; TAP2; PSMB8; TAP1) | 2.33 | 6.49e-04 | 6.98e-03 |
|  | 14 | 70655803 | 70656116 | PDFSTATSENSEMBLUCSC | 314 | 8 | 12485 | tss | 0.568964 | 0.207406 | RP11-486O13.2; SLC8A3 (margin: RP11-486O13.2; SLC8A3) | 2.33 | 6.99e-03 | 3.19e-02 |
|  | 10 | 43866966 | 43867071 | PDFSTATSENSEMBLUCSC | 106 | 5 | 5046 | tss | 0.461032 | 0.814038 | FXYD4 (margin: FXYD4) | 2.33 | 7.94e-03 | 3.19e-02 |
|  | 10 | 100993553 | 100994143 | PDFSTATSENSEMBLUCSC | 591 | 11 | 4706 | gene;island | 0.868307 | 0.959582 | HPSE2 (margin: HPSE2) | 2.33 | 1.28e-02 | 4.76e-02 |
|  | 14 | 85996101 | 85996393 | PDFSTATSENSEMBLUCSC | 293 | 8 | 12542 | tss | 0.175518 | 0.487793 | RP11-497E19.2; FLRT2; RP11-497E19.1 (margin: RP11-497E19.2; FLRT2; RP11-497E19.1) | 2.33 | 2.95e-03 | 1.88e-02 |
|  | 3 | 132756721 | 132757106 | PDFSTATSENSEMBLUCSC | 386 | 9 | 28599 | tss | 0.927082 | 0.980595 | TMEM108; RP11-469L4.1 (margin: TMEM108; RP11-469L4.1) | 2.33 | 1.06e-02 | 4.01e-02 |
|  | 6 | 33288180 | 33289280 | PDFSTATSENSEMBLUCSC | 1101 | 36 | 34268 | gene;island | 0.786933 | 0.901894 | DAXX (margin: TAPBP; ZBTB22; DAXX) | 2.33 | 1.64e-03 | 1.45e-02 |
|  | 16 | 77469538 | 77469710 | PDFSTATSENSEMBLUCSC | 173 | 5 | 15369 | tss;island | 0.272443 | 0.041833 | RP11-449J10.1; ADAMTS18; AC025284.1 (margin: RP11-449J10.1; ADAMTS18; AC025284.1) | 2.33 | 7.94e-03 | 3.19e-02 |
|  | 1 | 229569877 | 229570288 | PDFSTATSENSEMBLUCSC | 412 | 11 | 2311 | tss | 0.768394 | 0.924758 | ACTA1 (margin: RP5-1068B5.1; ACTA1; RP5-1068B5.3; NUP133) | 2.32 | 1.86e-03 | 1.45e-02 |
|  | 8 | 144457677 | 144458069 | PDFSTATSENSEMBLUCSC | 393 | 5 | 39474 | gene;island | 0.541181 | 0.870810 | RHPN1 (margin: RHPN1; RHPN1-AS1) | 2.32 | 7.94e-03 | 3.19e-02 |
|  | 7 | 2556962 | 2557466 | PDFSTATSENSEMBLUCSC | 505 | 8 | 36802 | gene;tss;island | 0.564502 | 0.820074 | LFNG (margin: LFNG; MIR4648) | 2.30 | 1.04e-02 | 3.96e-02 |
|  | 17 | 80668611 | 80668944 | PDFSTATSENSEMBLUCSC | 334 | 5 | 19006 | island | 0.696083 | 0.886419 | (margin: FN3KRP; RP11-388C12.1) | 2.30 | 7.94e-03 | 3.19e-02 |
|  | 12 | 39299501 | 39299844 | PDFSTATSENSEMBLUCSC | 344 | 6 | 9636 | tss | 0.453800 | 0.760164 | RP11-396F22.1; CPNE8 (margin: RP11-396F22.1; CPNE8; AC067735.1) | 2.29 | 2.16e-03 | 1.45e-02 |
|  | 6 | 33133329 | 33133752 | PDFSTATSENSEMBLUCSC | 424 | 9 | 34195 | gene | 0.368527 | 0.726795 | COL11A2 (margin: COL11A2) | 2.29 | 7.77e-03 | 3.19e-02 |
|  | 15 | 74494781 | 74494909 | PDFSTATSENSEMBLUCSC | 129 | 5 | 13420 | gene | 0.602113 | 0.840038 | RP11-60L3.1; STRA6 (margin: RP11-60L3.1; STRA6) | 2.29 | 7.94e-03 | 3.19e-02 |
|  | 4 | 3464653 | 3465004 | PDFSTATSENSEMBLUCSC | 352 | 9 | 30262 | tss | 0.409553 | 0.030037 | DOK7 (margin: DOK7) | 2.29 | 5.64e-03 | 3.18e-02 |
|  | 7 | 94285270 | 94286955 | PDFSTATSENSEMBLUCSC | 1686 | 61 | 37093 | gene;tss;island | 0.816427 | 0.441327 | PEG10; SGCE (margin: PEG10; SGCE) | 2.28 | 3.43e-12 | 5.17e-09 |
|  | 10 | 93392644 | 93393509 | PDFSTATSENSEMBLUCSC | 866 | 14 | 5932 | island | 0.504912 | 0.901141 | PPP1R3C (margin: PPP1R3C) | 2.28 | 5.23e-04 | 6.47e-03 |
|  | 6 | 118228382 | 118228650 | PDFSTATSENSEMBLUCSC | 269 | 6 | 35199 | tss;island | 0.691474 | 0.318317 | SLC35F1 (margin: SLC35F1) | 2.28 | 2.16e-03 | 1.45e-02 |
|  | 17 | 57184330 | 57184624 | PDFSTATSENSEMBLUCSC | 295 | 9 | 17541 | tss | 0.007384 | 0.021756 | AC099850.1; TRIM37 (margin: AC099850.1; TRIM37; SKA2) | 2.28 | 3.99e-03 | 2.47e-02 |
|  | 6 | 30881112 | 30882384 | PDFSTATSENSEMBLUCSC | 1273 | 39 | 35740 | island | 0.441796 | 0.049736 | GTF2H4; VARS2 (margin: RN7SL175P; GTF2H4; VARS2) | 2.27 | 1.16e-02 | 4.31e-02 |
|  | 1 | 12123262 | 12124114 | PDFSTATSENSEMBLUCSC | 853 | 10 | 2698 | island | 0.626779 | 0.868370 | TNFRSF8 (margin: TNFRSF8) | 2.27 | 6.84e-03 | 3.19e-02 |
|  | 6 | 33135753 | 33136061 | PDFSTATSENSEMBLUCSC | 309 | 7 | 34198 | gene | 0.558704 | 0.787015 | COL11A2 (margin: COL11A2) | 2.27 | 1.11e-02 | 4.14e-02 |
|  | 3 | 169530417 | 169530621 | PDFSTATSENSEMBLUCSC | 205 | 5 | 28709 | tss | 0.577273 | 0.906356 | LRRC34 (margin: LRRIQ4; LRRC34) | 2.27 | 7.94e-03 | 3.19e-02 |
|  | 1 | 229569608 | 229570288 | PDFSTATSENSEMBLUCSC | 681 | 14 | 4310 | island | 0.774712 | 0.930603 | ACTA1 (margin: RP5-1068B5.1; ACTA1; RP5-1068B5.3; NUP133) | 2.26 | 1.43e-03 | 1.36e-02 |
|  | 12 | 7055452 | 7056008 | PDFSTATSENSEMBLUCSC | 557 | 7 | 10221 | island | 0.066742 | 0.022265 | C12orf57; PTPN6; U47924.31 (margin: ATN1; C12orf57; RNU7-1; PTPN6; U47924.31) | 2.25 | 4.08e-03 | 2.47e-02 |
|  | 17 | 57184151 | 57184624 | PDFSTATSENSEMBLUCSC | 474 | 12 | 18606 | island | 0.007404 | 0.021810 | AC099850.1; TRIM37 (margin: AC099850.1; TRIM37; SKA2) | 2.25 | 5.56e-03 | 3.16e-02 |
|  | 1 | 107682504 | 107682963 | PDFSTATSENSEMBLUCSC | 460 | 6 | 3537 | island | 0.353124 | 0.719032 | NTNG1 (margin: NTNG1) | 2.24 | 2.16e-03 | 1.45e-02 |
|  | 5 | 178450457 | 178451176 | PDFSTATSENSEMBLUCSC | 720 | 9 | 33478 | island | 0.076743 | 0.309135 | ZNF879 (margin: ZNF879) | 2.24 | 3.99e-03 | 2.47e-02 |
|  | 10 | 18429643 | 18429760 | PDFSTATSENSEMBLUCSC | 118 | 6 | 4520 | gene;island | 0.733489 | 0.424320 | CACNB2 (margin: CACNB2) | 2.24 | 2.16e-03 | 1.45e-02 |
|  | 1 | 153650884 | 153651325 | PDFSTATSENSEMBLUCSC | 442 | 10 | 3767 | island | 0.092802 | 0.449420 | NPR1 (margin: NPR1; ILF2) | 2.24 | 1.50e-03 | 1.41e-02 |
|  | 8 | 48675647 | 48676092 | PDFSTATSENSEMBLUCSC | 446 | 7 | 40273 | island | 0.892919 | 0.970268 | (margin: PRKDC) | 2.24 | 5.83e-04 | 6.47e-03 |
|  | 17 | 72931569 | 72932048 | PDFSTATSENSEMBLUCSC | 480 | 12 | 18731 | island | 0.527000 | 0.861809 | OTOP2; OTOP3 (margin: OTOP2; OTOP3) | 2.23 | 2.01e-04 | 3.38e-03 |
|  | 19 | 52391078 | 52391789 | PDFSTATSENSEMBLUCSC | 712 | 13 | 22344 | island | 0.968067 | 0.836599 | CTC-429C10.2; ZNF577; ZNF649 (margin: CTC-429C10.2; ZNF577; ZNF649) | 2.23 | 3.49e-03 | 2.20e-02 |
|  | 10 | 119000927 | 119001590 | PDFSTATSENSEMBLUCSC | 664 | 8 | 4778 | gene;island | 0.773323 | 0.411506 | SLC18A2; RP11-501J20.5 (margin: SLC18A2; RP11-501J20.5) | 2.23 | 1.09e-03 | 1.10e-02 |
|  | 15 | 50979137 | 50979486 | PDFSTATSENSEMBLUCSC | 350 | 6 | 13670 | tss;island | 0.014115 | 0.036465 | RN7SL354P; TRPM7 (margin: RN7SL354P; TRPM7) | 2.21 | 4.33e-03 | 2.52e-02 |
|  | 1 | 247694271 | 247694557 | PDFSTATSENSEMBLUCSC | 287 | 5 | 1127 | gene;tss;island | 0.914779 | 0.980201 | GCSAML; OR2C3 (margin: GCSAML; GCSAML-AS1; OR2C3) | 2.21 | 7.94e-03 | 3.19e-02 |
|  | 10 | 15130387 | 15131036 | PDFSTATSENSEMBLUCSC | 650 | 10 | 5567 | island | 0.140784 | 0.008964 | DCLRE1CP1; ACBD7 (margin: RPP38; DCLRE1CP1; ACBD7; GAPDHP45; C10orf111) | 2.20 | 3.89e-03 | 2.43e-02 |
|  | 11 | 86383182 | 86383940 | PDFSTATSENSEMBLUCSC | 759 | 20 | 8712 | island | 0.838721 | 0.963529 | ME3 (margin: ME3) | 2.20 | 2.90e-05 | 1.16e-03 |
|  | 5 | 1212434 | 1212641 | PDFSTATSENSEMBLUCSC | 208 | 5 | 31526 | gene;island | 0.899383 | 0.970390 | SLC6A19 (margin: SLC6A19) | 2.20 | 7.94e-03 | 3.19e-02 |
|  | 4 | 166795260 | 166795913 | PDFSTATSENSEMBLUCSC | 654 | 9 | 30173 | gene;island | 0.475516 | 0.799892 | TLL1 (margin: TLL1) | 2.20 | 2.88e-04 | 4.58e-03 |
|  | 1 | 167599371 | 167599830 | PDFSTATSENSEMBLUCSC | 460 | 5 | 3947 | island | 0.882381 | 0.982408 | RCSD1; RP3-455J7.4 (margin: RCSD1; RP3-455J7.4) | 2.19 | 7.94e-03 | 3.19e-02 |
|  | 3 | 129407365 | 129407548 | PDFSTATSENSEMBLUCSC | 184 | 5 | 27943 | gene | 0.970907 | 0.877302 | TMCC1 (margin: TMCC1) | 2.19 | 7.94e-03 | 3.19e-02 |
|  | 5 | 37835506 | 37836392 | PDFSTATSENSEMBLUCSC | 887 | 11 | 31591 | gene;tss;island | 0.931966 | 0.988828 | GDNF (margin: GDNF-AS1; GDNF) | 2.19 | 1.86e-03 | 1.45e-02 |
|  | 17 | 6616644 | 6617192 | PDFSTATSENSEMBLUCSC | 549 | 8 | 17909 | island | 0.867168 | 0.963489 | SLC13A5 (margin: SLC13A5) | 2.18 | 4.66e-03 | 2.68e-02 |
|  | 6 | 32908466 | 32908794 | PDFSTATSENSEMBLUCSC | 329 | 6 | 34163 | gene | 0.009984 | 0.045460 | HLA-DMB; XXbac-BPG181M17.5 (margin: HLA-DMB; AL645941.1; XXbac-BPG181M17.5; HLA-DMA) | 2.18 | 4.33e-03 | 2.52e-02 |
|  | 17 | 46507549 | 46507895 | PDFSTATSENSEMBLUCSC | 347 | 8 | 18472 | island | 0.823462 | 0.956090 | SKAP1 (margin: SKAP1) | 2.18 | 1.04e-02 | 3.96e-02 |
|  | 16 | 82660596 | 82660873 | PDFSTATSENSEMBLUCSC | 278 | 5 | 14858 | gene | 0.184664 | 0.582719 | CDH13 (margin: CDH13) | 2.18 | 7.94e-03 | 3.19e-02 |
|  | 19 | 53073242 | 53073420 | PDFSTATSENSEMBLUCSC | 179 | 7 | 21016 | tss | 0.834115 | 0.477259 | ZNF701 (margin: ZNF808; ZNF701; RPL39P34; CTD-3099C6.7) | 2.17 | 4.08e-03 | 2.47e-02 |
|  | 10 | 71333152 | 71333216 | PDFSTATSENSEMBLUCSC | 65 | 5 | 5126 | tss | 0.361784 | 0.094450 | NEUROG3 (margin: RP11-343J3.2; NEUROG3) | 2.17 | 7.94e-03 | 3.19e-02 |
|  | 10 | 99790438 | 99790910 | PDFSTATSENSEMBLUCSC | 473 | 5 | 6000 | island | 0.872728 | 0.973013 | CRTAC1 (margin: CRTAC1) | 2.17 | 7.94e-03 | 3.19e-02 |
|  | 6 | 32811884 | 32812708 | PDFSTATSENSEMBLUCSC | 825 | 32 | 34877 | tss | 0.007803 | 0.061036 | XXbac-BPG246D15.8; PSMB9; PSMB8; TAP1 (margin: XXbac-BPG246D15.8; PSMB9; TAP2; PSMB8; TAP1) | 2.16 | 5.20e-05 | 1.45e-03 |
|  | 4 | 155547985 | 155548273 | PDFSTATSENSEMBLUCSC | 289 | 5 | 31383 | island | 0.147137 | 0.434336 | LRAT (margin: LRAT) | 2.16 | 7.94e-03 | 3.19e-02 |
|  | 4 | 5021203 | 5021328 | PDFSTATSENSEMBLUCSC | 126 | 5 | 30272 | tss | 0.642237 | 0.942385 | CYTL1 (margin: CYTL1) | 2.15 | 7.94e-03 | 3.19e-02 |
|  | 19 | 11784246 | 11785337 | PDFSTATSENSEMBLUCSC | 1092 | 15 | 21580 | island | 0.690615 | 0.957947 | ZNF833P (margin: ZNF833P; CTC-499B15.4; HNRNPA1P10) | 2.15 | 4.94e-03 | 2.83e-02 |
|  | 10 | 102976327 | 102977342 | PDFSTATSENSEMBLUCSC | 1016 | 11 | 6056 | island | 0.880302 | 0.974015 | (margin: LBX1) | 2.14 | 1.40e-03 | 1.33e-02 |
|  | 6 | 31628050 | 31628440 | PDFSTATSENSEMBLUCSC | 391 | 15 | 33929 | gene | 0.002256 | 0.020221 | C6orf47-AS1; C6orf47; GPANK1 (margin: APOM; C6orf47-AS1; Y\_RNA; CSNK2B; CSNK2B-LY6G5B-1181; LY6G5B; BAG6; C6orf47; GPANK1) | 2.14 | 1.67e-03 | 1.45e-02 |
|  | 3 | 10805956 | 10806569 | PDFSTATSENSEMBLUCSC | 614 | 11 | 28152 | tss | 0.754395 | 0.941563 | LINC00606 (margin: AC018495.3; LINC00606) | 2.13 | 1.40e-03 | 1.33e-02 |
|  | 22 | 39640835 | 39641154 | PDFSTATSENSEMBLUCSC | 320 | 7 | 27500 | island | 0.107085 | 0.027388 | PDGFB (margin: PDGFB) | 2.13 | 4.08e-03 | 2.47e-02 |
|  | 15 | 74218418 | 74218921 | PDFSTATSENSEMBLUCSC | 504 | 11 | 14292 | island | 0.803470 | 0.946542 | LOXL1; LOXL1-AS1 (margin: LOXL1; LOXL1-AS1) | 2.13 | 5.39e-05 | 1.47e-03 |
|  | 5 | 157098338 | 157098555 | PDFSTATSENSEMBLUCSC | 218 | 6 | 32457 | tss | 0.896274 | 0.975185 | C5orf52; SOX30 (margin: C5orf52; SOX30; AC008694.3) | 2.13 | 8.66e-03 | 3.36e-02 |
|  | 5 | 157098338 | 157098619 | PDFSTATSENSEMBLUCSC | 282 | 7 | 33346 | island | 0.906247 | 0.978656 | C5orf52; SOX30 (margin: C5orf52; SOX30; AC008694.3) | 2.13 | 1.11e-02 | 4.14e-02 |
|  | 8 | 79427993 | 79428725 | PDFSTATSENSEMBLUCSC | 733 | 8 | 40406 | island | 0.221891 | 0.662897 | PKIA; RP11-594N15.2 (margin: PKIA; RP11-594N15.2) | 2.12 | 4.66e-03 | 2.68e-02 |
|  | 17 | 15165908 | 15166153 | PDFSTATSENSEMBLUCSC | 246 | 6 | 17149 | tss | 0.068811 | 0.015919 | RP11-849N15.1; PMP22 (margin: RP11-849N15.1; PMP22; MIR4731; AC005703.3) | 2.12 | 4.33e-03 | 2.52e-02 |
|  | 7 | 27224092 | 27225897 | PDFSTATSENSEMBLUCSC | 1806 | 34 | 37499 | tss;island | 0.809211 | 0.952166 | HOXA11-AS; HOXA11; RP1-170O19.14 (margin: HOXA11-AS; RP1-170O19.20; HOXA10; HOXA11; RP1-170O19.14; HOXA13) | 2.11 | 3.93e-05 | 1.17e-03 |
|  | 8 | 63161579 | 63161676 | PDFSTATSENSEMBLUCSC | 98 | 5 | 39318 | gene;island | 0.813229 | 0.496320 | NKAIN3 (margin: NKAIN3) | 2.11 | 7.94e-03 | 3.19e-02 |
|  | 14 | 101192190 | 101193038 | PDFSTATSENSEMBLUCSC | 849 | 16 | 12607 | tss;island | 0.889818 | 0.967475 | DLK1 (margin: DLK1) | 2.11 | 6.53e-04 | 7.01e-03 |
|  | 10 | 3823758 | 3824016 | PDFSTATSENSEMBLUCSC | 259 | 5 | 4475 | gene;island | 0.899679 | 0.981222 | KLF6 (margin: KLF6) | 2.10 | 7.94e-03 | 3.19e-02 |
|  | 7 | 73894573 | 73895278 | PDFSTATSENSEMBLUCSC | 706 | 7 | 37044 | gene;island | 0.790658 | 0.948969 | GTF2IRD1 (margin: GTF2IRD1; RNA5SP233) | 2.10 | 5.83e-04 | 6.47e-03 |
|  | 17 | 33775295 | 33776116 | PDFSTATSENSEMBLUCSC | 822 | 12 | 18214 | island | 0.648494 | 0.953507 | SLFN13 (margin: SLFN13; RP11-1094M14.7) | 2.10 | 2.91e-03 | 1.88e-02 |
|  | 7 | 23720292 | 23720838 | PDFSTATSENSEMBLUCSC | 547 | 6 | 36920 | gene;island | 0.616283 | 0.309860 | FAM221A; AC006026.13 (margin: FAM221A; AC006026.1; AC006026.13) | 2.09 | 2.16e-03 | 1.45e-02 |
|  | 17 | 79495268 | 79495348 | PDFSTATSENSEMBLUCSC | 81 | 5 | 17740 | tss | 0.379580 | 0.682061 | RP13-766D20.2; FSCN2 (margin: RP13-766D20.2; FSCN2; ACTG1; C17orf70) | 2.08 | 7.94e-03 | 3.19e-02 |
|  | 7 | 150755072 | 150755981 | PDFSTATSENSEMBLUCSC | 910 | 20 | 37987 | tss | 0.012987 | 0.050783 | SLC4A2; CDK5 (margin: ABCB8; ASIC3; SLC4A2; CDK5) | 2.07 | 5.62e-03 | 3.18e-02 |
|  | 20 | 29611652 | 29611924 | PDFSTATSENSEMBLUCSC | 273 | 8 | 26069 | island | 0.591716 | 0.237872 | FRG1B (margin: FRG1B) | 2.07 | 6.22e-04 | 6.71e-03 |
|  | 4 | 89618324 | 89619573 | PDFSTATSENSEMBLUCSC | 1250 | 20 | 30055 | gene | 0.916552 | 0.727714 | HERC3; NAP1L5 (margin: HERC3; FAM13A-AS1; NAP1L5) | 2.06 | 1.77e-03 | 1.45e-02 |
|  | 11 | 65601167 | 65601427 | PDFSTATSENSEMBLUCSC | 261 | 10 | 8481 | island | 0.817298 | 0.968632 | SNX32; CFL1 (margin: SNX32; CFL1) | 2.06 | 5.20e-03 | 2.96e-02 |
|  | 1 | 156261200 | 156261403 | PDFSTATSENSEMBLUCSC | 204 | 5 | 820 | gene;island | 0.865515 | 0.963143 | TMEM79; C1orf85 (margin: TMEM79; SMG5; C1orf85; VHLL) | 2.06 | 7.94e-03 | 3.19e-02 |
|  | 14 | 106938234 | 106938621 | PDFSTATSENSEMBLUCSC | 388 | 10 | 13248 | island | 0.870637 | 0.966609 | LINC00221 (margin: LINC00221; IGHV3-43; IGHVII-43-1; IGHVIII-44; IGHVIV-44-1; IGHVII-44-2) | 2.05 | 5.20e-03 | 2.96e-02 |
|  | 4 | 80247171 | 80247281 | PDFSTATSENSEMBLUCSC | 111 | 5 | 30456 | tss | 0.886046 | 0.976266 | NAA11 (margin: NAA11) | 2.05 | 7.94e-03 | 3.19e-02 |
|  | 7 | 27225058 | 27225897 | PDFSTATSENSEMBLUCSC | 840 | 23 | 36942 | gene | 0.833675 | 0.959871 | HOXA11-AS; HOXA11; RP1-170O19.14 (margin: HOXA11-AS; RP1-170O19.20; HOXA10; HOXA11; RP1-170O19.14; HOXA13) | 2.04 | 1.01e-06 | 9.49e-05 |
|  | 7 | 150864885 | 150865291 | PDFSTATSENSEMBLUCSC | 407 | 10 | 37993 | tss;island | 0.140529 | 0.032386 | GBX1 (margin: GBX1; ASB10) | 2.03 | 1.15e-02 | 4.29e-02 |
|  | 11 | 3187271 | 3188016 | PDFSTATSENSEMBLUCSC | 746 | 24 | 7092 | tss;island | 0.814123 | 0.934281 | OSBPL5 (margin: OSBPL5) | 2.03 | 7.11e-05 | 1.91e-03 |
|  | 19 | 58038573 | 58038689 | PDFSTATSENSEMBLUCSC | 117 | 6 | 21118 | tss | 0.661445 | 0.356010 | ZNF549 (margin: ZNF773; ZNF549; ZNF550) | 2.02 | 2.16e-03 | 1.45e-02 |
|  | 15 | 45815087 | 45815405 | PDFSTATSENSEMBLUCSC | 319 | 7 | 13334 | gene;tss;island | 0.046648 | 0.011735 | HMGN2P46; SLC30A4 (margin: RP11-519G16.3; HMGN2P46; SLC30A4) | 2.01 | 1.11e-02 | 4.14e-02 |
|  | 4 | 81187601 | 81187906 | PDFSTATSENSEMBLUCSC | 306 | 5 | 31136 | island | 0.521121 | 0.212256 | FGF5 (margin: FGF5) | 2.01 | 7.94e-03 | 3.19e-02 |
|  | 17 | 4544925 | 4545157 | PDFSTATSENSEMBLUCSC | 233 | 5 | 17873 | island | 0.903767 | 0.972708 | ALOX15 (margin: ALOX15) | 2.01 | 7.94e-03 | 3.19e-02 |
|  | 11 | 2017362 | 2018247 | PDFSTATSENSEMBLUCSC | 886 | 13 | 6443 | gene;island | 0.591597 | 0.937674 | H19 (margin: MRPL23; AC051649.6; MRPL23-AS1; H19) | 2.01 | 7.95e-04 | 8.28e-03 |
|  | 2 | 239139911 | 239140296 | PDFSTATSENSEMBLUCSC | 386 | 7 | 23096 | gene | 0.937505 | 0.983479 | AC096574.4; AC016757.3 (margin: AC096574.4; AC016757.3; HES6) | 2.01 | 2.33e-03 | 1.52e-02 |
|  | 2 | 128422179 | 128422717 | PDFSTATSENSEMBLUCSC | 539 | 8 | 23614 | tss | 0.863115 | 0.953905 | LIMS2 (margin: GPR17; LIMS2) | 2.01 | 1.04e-02 | 3.96e-02 |
|  | 19 | 37157565 | 37157995 | PDFSTATSENSEMBLUCSC | 431 | 11 | 21944 | island | 0.039552 | 0.005840 | ZNF461 (margin: ZNF461) | 2.00 | 4.10e-03 | 2.48e-02 |
|  | 11 | 1036714 | 1036938 | PDFSTATSENSEMBLUCSC | 225 | 6 | 7036 | tss | 0.098237 | 0.384009 | MUC6 (margin: MUC6) | 2.00 | 8.66e-03 | 3.36e-02 |
|  | 17 | 6947288 | 6947464 | PDFSTATSENSEMBLUCSC | 177 | 8 | 17060 | tss | 0.852850 | 0.915995 | SLC16A11 (margin: SLC16A13; SLC16A11) | 1.99 | 6.22e-04 | 6.71e-03 |
|  | 1 | 46951291 | 46951683 | PDFSTATSENSEMBLUCSC | 393 | 6 | 3219 | island | 0.936853 | 0.981450 | (margin: LINC00505) | 1.99 | 2.16e-03 | 1.45e-02 |
|  | 6 | 122931154 | 122931702 | PDFSTATSENSEMBLUCSC | 549 | 11 | 34463 | gene;island | 0.073470 | 0.019806 | PKIB (margin: PKIB) | 1.98 | 3.94e-04 | 5.86e-03 |
|  | 10 | 90750169 | 90750580 | PDFSTATSENSEMBLUCSC | 412 | 7 | 4669 | gene;island | 0.007729 | 0.035683 | FAS; ACTA2 (margin: FAS; ACTA2) | 1.97 | 1.11e-02 | 4.14e-02 |
|  | 2 | 162283434 | 162284206 | PDFSTATSENSEMBLUCSC | 773 | 8 | 24823 | island | 0.902938 | 0.976036 | TBR1; AC009487.5; RP11-334E15.2 (margin: TBR1; AC009487.5; RP11-334E15.2; AC009487.4) | 1.97 | 1.09e-03 | 1.10e-02 |
|  | 11 | 2907670 | 2908471 | PDFSTATSENSEMBLUCSC | 802 | 14 | 7081 | tss;island | 0.891219 | 0.972002 | CDKN1C; SLC22A18AS (margin: CDKN1C; SLC22A18AS) | 1.96 | 1.22e-02 | 4.56e-02 |
|  | 19 | 53073242 | 53074012 | PDFSTATSENSEMBLUCSC | 771 | 11 | 22360 | island | 0.772057 | 0.438828 | ZNF701 (margin: ZNF808; ZNF701; RPL39P34; CTD-3099C6.7) | 1.96 | 2.75e-04 | 4.46e-03 |
|  | 11 | 67777618 | 67777770 | PDFSTATSENSEMBLUCSC | 153 | 6 | 7503 | tss | 0.021765 | 0.087019 | ALDH3B1 (margin: ALDH3B1; UNC93B1) | 1.96 | 4.33e-03 | 2.52e-02 |
|  | 15 | 74218418 | 74218780 | PDFSTATSENSEMBLUCSC | 363 | 9 | 13785 | tss | 0.881955 | 0.967210 | LOXL1; LOXL1-AS1 (margin: LOXL1; LOXL1-AS1) | 1.95 | 7.82e-04 | 8.17e-03 |
|  | 15 | 74659901 | 74660566 | PDFSTATSENSEMBLUCSC | 666 | 7 | 13792 | tss;island | 0.489115 | 0.745669 | CTD-2311M21.3; CYP11A1 (margin: CTD-2311M21.2; CTD-2311M21.3; CYP11A1) | 1.94 | 6.99e-03 | 3.19e-02 |
|  | 6 | 31324227 | 31324684 | PDFSTATSENSEMBLUCSC | 458 | 13 | 33869 | gene;island | 0.045498 | 0.250892 | XXbac-BPG248L24.12; HLA-B (margin: XXbac-BPG248L24.12; HLA-B; DHFRP2) | 1.92 | 7.24e-03 | 3.19e-02 |
|  | 6 | 31628050 | 31629199 | PDFSTATSENSEMBLUCSC | 1150 | 25 | 35813 | island | 0.005318 | 0.027250 | C6orf47-AS1; Y\_RNA; C6orf47; GPANK1 (margin: APOM; C6orf47-AS1; Y\_RNA; CSNK2B; CSNK2B-LY6G5B-1181; LY6G5B; BAG6; C6orf47; GPANK1) | 1.92 | 4.53e-03 | 2.63e-02 |
|  | 6 | 17102310 | 17102678 | PDFSTATSENSEMBLUCSC | 369 | 6 | 35473 | island | 0.660293 | 0.363502 | STMND1 (margin: STMND1) | 1.91 | 8.66e-03 | 3.36e-02 |
|  | 8 | 55370192 | 55370579 | PDFSTATSENSEMBLUCSC | 388 | 10 | 40302 | island | 0.862163 | 0.972374 | SOX17 (margin: SOX17) | 1.90 | 1.50e-03 | 1.41e-02 |
|  | 3 | 50383167 | 50383485 | PDFSTATSENSEMBLUCSC | 319 | 7 | 28354 | tss | 0.315713 | 0.490518 | ZMYND10-AS1; ZMYND10; NPRL2 (margin: ZMYND10-AS1; CYB561D2; XXcos-LUCA11.5; RASSF1; ZMYND10; NPRL2; TMEM115) | 1.90 | 6.99e-03 | 3.19e-02 |
|  | 6 | 32187918 | 32188404 | PDFSTATSENSEMBLUCSC | 487 | 5 | 34098 | gene | 0.231089 | 0.536047 | NOTCH4 (margin: NOTCH4) | 1.90 | 7.94e-03 | 3.19e-02 |
|  | 11 | 60623415 | 60623918 | PDFSTATSENSEMBLUCSC | 504 | 7 | 8314 | island | 0.584662 | 0.857171 | PTGDR2 (margin: CCDC86; ZP1; RP11-804A23.4; PTGDR2) | 1.89 | 1.17e-03 | 1.13e-02 |
|  | 11 | 44331905 | 44333192 | PDFSTATSENSEMBLUCSC | 1288 | 40 | 7261 | tss;island | 0.795993 | 0.961162 | ALX4 (margin: ALX4) | 1.88 | 6.54e-05 | 1.76e-03 |
|  | 8 | 1094484 | 1094904 | PDFSTATSENSEMBLUCSC | 421 | 6 | 40012 | island | 0.554453 | 0.757834 | CTD-2281E23.2 (margin: ERICH1-AS1; CTD-2281E23.2) | 1.87 | 8.66e-03 | 3.36e-02 |
|  | 6 | 31762353 | 31762901 | PDFSTATSENSEMBLUCSC | 549 | 15 | 33958 | gene;island | 0.009670 | 0.031706 | VARS (margin: VARS; LSM2) | 1.87 | 5.78e-04 | 6.47e-03 |
|  | 11 | 60623504 | 60623918 | PDFSTATSENSEMBLUCSC | 415 | 6 | 7323 | tss | 0.598316 | 0.873791 | PTGDR2 (margin: CCDC86; ZP1; RP11-804A23.4; PTGDR2) | 1.87 | 4.33e-03 | 2.52e-02 |
|  | 6 | 29759718 | 29760164 | PDFSTATSENSEMBLUCSC | 447 | 14 | 33693 | gene;island | 0.126507 | 0.362549 | HLA-V; HCG4 (margin: HLA-V; HLA-P; HCG4; RPL7AP7) | 1.86 | 7.87e-03 | 3.19e-02 |
|  | 16 | 80966033 | 80966491 | PDFSTATSENSEMBLUCSC | 459 | 6 | 16118 | island | 0.848212 | 0.583217 | (margin: ) | 1.86 | 2.16e-03 | 1.45e-02 |
|  | 2 | 128422113 | 128422717 | PDFSTATSENSEMBLUCSC | 605 | 10 | 22862 | gene;island | 0.863115 | 0.952667 | LIMS2 (margin: GPR17; LIMS2) | 1.86 | 2.88e-03 | 1.86e-02 |
|  | 17 | 46655164 | 46656093 | PDFSTATSENSEMBLUCSC | 930 | 18 | 18480 | island | 0.861904 | 0.973590 | HOXB-AS3; HOXB3; HOXB4; MIR10A (margin: HOXB-AS3; HOXB3; HOXB4; MIR10A) | 1.85 | 2.39e-05 | 9.70e-04 |
|  | 17 | 9728972 | 9729337 | PDFSTATSENSEMBLUCSC | 366 | 8 | 17131 | tss | 0.630880 | 0.779803 | GLP2R (margin: GLP2R; RP11-477N12.3) | 1.85 | 6.99e-03 | 3.19e-02 |
|  | 10 | 93392870 | 93393509 | PDFSTATSENSEMBLUCSC | 640 | 10 | 5223 | tss | 0.504912 | 0.842422 | PPP1R3C (margin: PPP1R3C) | 1.84 | 6.84e-03 | 3.19e-02 |
|  | 11 | 86383241 | 86383940 | PDFSTATSENSEMBLUCSC | 700 | 18 | 7610 | tss | 0.848051 | 0.967069 | ME3 (margin: ME3) | 1.84 | 2.11e-04 | 3.49e-03 |
|  | 11 | 368351 | 369192 | PDFSTATSENSEMBLUCSC | 842 | 24 | 7009 | tss | 0.351758 | 0.134187 | B4GALNT4 (margin: B4GALNT4) | 1.83 | 9.36e-03 | 3.61e-02 |
|  | 13 | 100641646 | 100642106 | PDFSTATSENSEMBLUCSC | 461 | 6 | 11849 | island | 0.929146 | 0.977717 | (margin: ZIC2; LINC00554) | 1.82 | 2.16e-03 | 1.45e-02 |
|  | 7 | 150754936 | 150755981 | PDFSTATSENSEMBLUCSC | 1046 | 23 | 39022 | island | 0.013311 | 0.047650 | SLC4A2; CDK5 (margin: ABCB8; ASIC3; SLC4A2; CDK5) | 1.82 | 3.35e-03 | 2.12e-02 |
|  | 22 | 39640959 | 39641154 | PDFSTATSENSEMBLUCSC | 196 | 6 | 27150 | tss | 0.101303 | 0.027173 | PDGFB (margin: PDGFB) | 1.82 | 4.33e-03 | 2.52e-02 |
|  | 14 | 70346151 | 70346477 | PDFSTATSENSEMBLUCSC | 327 | 5 | 12127 | gene | 0.797379 | 0.935126 | SMOC1 (margin: SMOC1; RPL7AP6) | 1.82 | 7.94e-03 | 3.19e-02 |
|  | 7 | 56031952 | 56032122 | PDFSTATSENSEMBLUCSC | 171 | 6 | 37606 | tss;island | 0.034393 | 0.010456 | GBAS (margin: MRPS17; GBAS) | 1.81 | 8.66e-03 | 3.36e-02 |
|  | 2 | 209271164 | 209271505 | PDFSTATSENSEMBLUCSC | 342 | 5 | 23850 | tss | 0.892352 | 0.977309 | PTH2R (margin: PTH2R) | 1.80 | 7.94e-03 | 3.19e-02 |
|  | 20 | 61051317 | 61052060 | PDFSTATSENSEMBLUCSC | 744 | 18 | 25872 | tss;island | 0.721073 | 0.921595 | RP13-379O24.3; GATA5 (margin: RP13-379O24.3; GATA5) | 1.80 | 1.32e-04 | 2.71e-03 |
|  | 6 | 32817662 | 32818477 | PDFSTATSENSEMBLUCSC | 816 | 12 | 34154 | gene;island | 0.985170 | 0.948747 | PSMB9; TAP1 (margin: XXbac-BPG246D15.8; PSMB9; TAP2; PSMB8; TAP1) | 1.80 | 1.83e-03 | 1.45e-02 |
|  | 4 | 11430353 | 11430908 | PDFSTATSENSEMBLUCSC | 556 | 9 | 30928 | island | 0.010810 | 0.042721 | HS3ST1 (margin: HS3ST1) | 1.79 | 2.76e-03 | 1.78e-02 |
|  | 4 | 11430552 | 11430908 | PDFSTATSENSEMBLUCSC | 357 | 7 | 30302 | tss | 0.010992 | 0.051810 | HS3ST1 (margin: HS3ST1) | 1.79 | 4.08e-03 | 2.47e-02 |
|  | 8 | 41504884 | 41505042 | PDFSTATSENSEMBLUCSC | 159 | 5 | 39671 | tss | 0.612834 | 0.862125 | NKX6-3 (margin: NKX6-3; ANK1) | 1.79 | 7.94e-03 | 3.19e-02 |
|  | 8 | 41504566 | 41505042 | PDFSTATSENSEMBLUCSC | 477 | 7 | 40247 | island | 0.612834 | 0.892720 | NKX6-3 (margin: NKX6-3; ANK1) | 1.79 | 6.99e-03 | 3.19e-02 |
|  | 14 | 70345898 | 70346477 | PDFSTATSENSEMBLUCSC | 580 | 10 | 12974 | island | 0.695844 | 0.874666 | SMOC1 (margin: SMOC1; RPL7AP6) | 1.79 | 8.93e-03 | 3.45e-02 |
|  | 11 | 64360724 | 64361020 | PDFSTATSENSEMBLUCSC | 297 | 6 | 6690 | gene | 0.573100 | 0.839890 | SLC22A12 (margin: SLC22A12) | 1.78 | 2.16e-03 | 1.45e-02 |
|  | 20 | 61446962 | 61448325 | PDFSTATSENSEMBLUCSC | 1364 | 42 | 25882 | tss;island | 0.564441 | 0.877416 | OGFR; COL9A3 (margin: OGFR; COL9A3; OGFR-AS1) | 1.77 | 5.73e-06 | 3.56e-04 |
|  | 12 | 54392734 | 54393725 | PDFSTATSENSEMBLUCSC | 992 | 16 | 9751 | tss;island | 0.880733 | 0.652767 | HOXC5; HOXC6; HOXC9; HOXC-AS1 (margin: HOXC10; HOXC5; HOXC6; MIR196A2; HOXC9; HOXC8; HOXC-AS2; HOXC-AS1) | 1.77 | 6.53e-04 | 7.01e-03 |
|  | 22 | 45680498 | 45681036 | PDFSTATSENSEMBLUCSC | 539 | 12 | 27573 | island | 0.762491 | 0.522119 | UPK3A (margin: CTA-268H5.9; UPK3A) | 1.77 | 5.56e-03 | 3.16e-02 |
|  | 7 | 50861418 | 50862266 | PDFSTATSENSEMBLUCSC | 849 | 20 | 37598 | tss;island | 0.655393 | 0.929866 | GRB10 (margin: GRB10) | 1.77 | 3.89e-03 | 2.43e-02 |
|  | 10 | 129845375 | 129845766 | PDFSTATSENSEMBLUCSC | 392 | 6 | 5438 | tss | 0.088373 | 0.246684 | PTPRE (margin: PTPRE) | 1.77 | 2.16e-03 | 1.45e-02 |
|  | 10 | 129845375 | 129846082 | PDFSTATSENSEMBLUCSC | 708 | 8 | 4821 | gene | 0.089666 | 0.246684 | PTPRE (margin: PTPRE) | 1.77 | 1.04e-02 | 3.96e-02 |
|  | 14 | 101291068 | 101292392 | PDFSTATSENSEMBLUCSC | 1325 | 22 | 12608 | tss;island | 0.817094 | 0.942019 | MEG3 (margin: MEG3; MIR2392; AL117190.2; RP11-123M6.2) | 1.76 | 1.69e-03 | 1.45e-02 |
|  | 3 | 37033625 | 37034787 | PDFSTATSENSEMBLUCSC | 1163 | 22 | 27716 | gene | 0.004070 | 0.017113 | MLH1; EPM2AIP1 (margin: MLH1; RP11-129K12.4; RP11-129K12.3; EPM2AIP1) | 1.75 | 9.18e-03 | 3.55e-02 |
|  | 6 | 32805142 | 32806699 | PDFSTATSENSEMBLUCSC | 1558 | 31 | 35928 | island | 0.013101 | 0.037644 | TAP2; PSMB8 (margin: XXbac-BPG246D15.8; PSMB9; TAP2; PSMB8; TAP1) | 1.74 | 9.55e-04 | 9.93e-03 |
|  | 15 | 93631578 | 93631991 | PDFSTATSENSEMBLUCSC | 414 | 6 | 13491 | gene;island | 0.220184 | 0.053667 | RGMA (margin: RGMA) | 1.73 | 8.66e-03 | 3.36e-02 |
|  | 11 | 2292361 | 2293309 | PDFSTATSENSEMBLUCSC | 949 | 32 | 7064 | tss;island | 0.760247 | 0.505816 | ASCL2 (margin: ASCL2) | 1.72 | 1.90e-09 | 6.85e-07 |
|  | 17 | 6947032 | 6947464 | PDFSTATSENSEMBLUCSC | 433 | 10 | 17918 | island | 0.842418 | 0.915213 | SLC16A11 (margin: SLC16A13; SLC16A11) | 1.72 | 3.89e-03 | 2.43e-02 |
|  | 13 | 36871646 | 36872346 | PDFSTATSENSEMBLUCSC | 701 | 14 | 11668 | island | 0.557836 | 0.670678 | SOHLH2; CCDC169-SOHLH2; CCDC169 (margin: SOHLH2; CCDC169-SOHLH2; CCDC169; SPG20) | 1.71 | 5.76e-03 | 3.19e-02 |
|  | 1 | 3634811 | 3635210 | PDFSTATSENSEMBLUCSC | 400 | 6 | 142 | gene;island | 0.882698 | 0.965819 | TP73 (margin: TP73; RP5-1092A11.2) | 1.71 | 8.66e-03 | 3.36e-02 |
|  | 1 | 2058230 | 2059086 | PDFSTATSENSEMBLUCSC | 857 | 6 | 62 | gene | 0.918789 | 0.969282 | PRKCZ (margin: PRKCZ) | 1.70 | 8.66e-03 | 3.36e-02 |
|  | 16 | 88717374 | 88717989 | PDFSTATSENSEMBLUCSC | 616 | 13 | 16212 | island | 0.617213 | 0.239391 | CYBA; MVD (margin: IL17C; SNAI3-AS1; CYBA; MVD) | 1.70 | 2.87e-03 | 1.85e-02 |
|  | 7 | 14030127 | 14030482 | PDFSTATSENSEMBLUCSC | 356 | 5 | 36900 | gene;tss;island | 0.010044 | 0.026710 | ETV1 (margin: ETV1) | 1.69 | 7.94e-03 | 3.19e-02 |
|  | 2 | 61371880 | 61372226 | PDFSTATSENSEMBLUCSC | 347 | 7 | 23377 | tss | 0.413036 | 0.670610 | KIAA1841; C2orf74; AC016747.3 (margin: KIAA1841; C2orf74; AC016747.3; RP11-493E12.1) | 1.69 | 1.17e-03 | 1.13e-02 |
|  | 4 | 17782853 | 17783502 | PDFSTATSENSEMBLUCSC | 650 | 11 | 30949 | island | 0.834711 | 0.605013 | FAM184B (margin: FAM184B) | 1.67 | 1.28e-02 | 4.76e-02 |
|  | 6 | 31865522 | 31866286 | PDFSTATSENSEMBLUCSC | 765 | 26 | 34849 | tss;island | 0.003202 | 0.010547 | C2; EHMT2; ZBTB12 (margin: C2; EHMT2; ZBTB12) | 1.66 | 5.18e-04 | 6.47e-03 |
|  | 1 | 111888719 | 111889159 | PDFSTATSENSEMBLUCSC | 441 | 8 | 1823 | tss | 0.072979 | 0.019086 | PIFO (margin: PIFO; CHIAP3) | 1.66 | 1.04e-02 | 3.96e-02 |
|  | 18 | 76740088 | 76740284 | PDFSTATSENSEMBLUCSC | 197 | 7 | 19593 | island | 0.950974 | 0.987203 | SALL3; RP11-849I19.1 (margin: SALL3; RP11-849I19.1) | 1.66 | 2.33e-03 | 1.52e-02 |
|  | 22 | 42896105 | 42896573 | PDFSTATSENSEMBLUCSC | 469 | 6 | 27191 | tss | 0.700385 | 0.904744 | SERHL (margin: SERHL; RRP7A) | 1.65 | 8.66e-03 | 3.36e-02 |
|  | 5 | 88179867 | 88180330 | PDFSTATSENSEMBLUCSC | 464 | 6 | 31683 | gene;tss;island | 0.654113 | 0.397112 | MEF2C-AS1; MEF2C (margin: MEF2C-AS1; MEF2C) | 1.65 | 2.16e-03 | 1.45e-02 |
|  | 16 | 3068014 | 3068707 | PDFSTATSENSEMBLUCSC | 694 | 11 | 15629 | island | 0.911663 | 0.970124 | TNFRSF12A; CLDN6 (margin: LA16c-380H5.5; CLDN9; TNFRSF12A; THOC6; CLDN6; HCFC1R1; CCDC64B) | 1.65 | 1.28e-02 | 4.76e-02 |
|  | 18 | 76740088 | 76740262 | PDFSTATSENSEMBLUCSC | 175 | 6 | 19332 | tss | 0.957786 | 0.987872 | SALL3; RP11-849I19.1 (margin: SALL3; RP11-849I19.1) | 1.65 | 2.16e-03 | 1.45e-02 |
|  | 11 | 2293552 | 2293665 | PDFSTATSENSEMBLUCSC | 114 | 6 | 7065 | tss;island | 0.874190 | 0.952409 | ASCL2 (margin: ASCL2) | 1.65 | 2.16e-03 | 1.45e-02 |
|  | 6 | 33047944 | 33048919 | PDFSTATSENSEMBLUCSC | 976 | 25 | 34186 | gene;island | 0.884545 | 0.955398 | HLA-DPB1; RPL32P1; HLA-DPA1 (margin: HLA-DPB1; RPL32P1; HLA-DPA1; HLA-DPA2) | 1.63 | 7.99e-03 | 3.21e-02 |
|  | 1 | 158083159 | 158083569 | PDFSTATSENSEMBLUCSC | 411 | 6 | 3876 | island | 0.319420 | 0.622104 | (margin: ) | 1.63 | 2.16e-03 | 1.45e-02 |
|  | 6 | 33256078 | 33258581 | PDFSTATSENSEMBLUCSC | 2504 | 57 | 34899 | tss;island | 0.007424 | 0.022272 | PFDN6; WDR46; RGL2 (margin: RPS18; B3GALT4; PFDN6; WDR46; RGL2; TAPBP) | 1.62 | 6.98e-03 | 3.19e-02 |
|  | 3 | 50382952 | 50383485 | PDFSTATSENSEMBLUCSC | 534 | 11 | 29154 | island | 0.315713 | 0.522278 | ZMYND10-AS1; ZMYND10; NPRL2 (margin: ZMYND10-AS1; CYB561D2; XXcos-LUCA11.5; RASSF1; ZMYND10; NPRL2; TMEM115) | 1.61 | 3.18e-03 | 2.01e-02 |
|  | 6 | 31914952 | 31915198 | PDFSTATSENSEMBLUCSC | 247 | 8 | 33996 | gene | 0.978659 | 0.934543 | C2; CFB (margin: C2; CFB; SKIV2L; NELFE; MIR1236) | 1.61 | 1.04e-02 | 3.96e-02 |
|  | 3 | 49314370 | 49314920 | PDFSTATSENSEMBLUCSC | 551 | 11 | 29113 | island | 0.948245 | 0.981949 | C3orf62; USP4 (margin: Y\_RNA; MIR4271; C3orf62; USP4) | 1.58 | 7.65e-04 | 8.13e-03 |
|  | 3 | 49314539 | 49314920 | PDFSTATSENSEMBLUCSC | 382 | 9 | 28325 | tss | 0.952964 | 0.981949 | C3orf62; USP4 (margin: Y\_RNA; MIR4271; C3orf62; USP4) | 1.58 | 3.99e-03 | 2.47e-02 |
|  | 3 | 49314595 | 49314920 | PDFSTATSENSEMBLUCSC | 326 | 8 | 27779 | gene | 0.956234 | 0.984618 | C3orf62; USP4 (margin: Y\_RNA; MIR4271; C3orf62; USP4) | 1.57 | 1.04e-02 | 3.96e-02 |
|  | 2 | 44065259 | 44065893 | PDFSTATSENSEMBLUCSC | 635 | 13 | 22661 | gene | 0.769590 | 0.888537 | ABCG8; ABCG5 (margin: ABCG8; ABCG5) | 1.57 | 4.22e-03 | 2.52e-02 |
|  | 2 | 44065259 | 44066294 | PDFSTATSENSEMBLUCSC | 1036 | 19 | 23329 | tss | 0.769590 | 0.888537 | ABCG8; ABCG5 (margin: ABCG8; ABCG5) | 1.57 | 1.19e-02 | 4.42e-02 |
|  | 8 | 86350889 | 86351018 | PDFSTATSENSEMBLUCSC | 130 | 5 | 39795 | tss | 0.685549 | 0.428133 | CA3 (margin: CA3; RP11-317J10.2) | 1.57 | 7.94e-03 | 3.19e-02 |
|  | 14 | 88792913 | 88793311 | PDFSTATSENSEMBLUCSC | 399 | 8 | 13057 | island | 0.297270 | 0.524384 | KCNK10 (margin: KCNK10) | 1.57 | 4.66e-03 | 2.68e-02 |
|  | 1 | 3566701 | 3568245 | PDFSTATSENSEMBLUCSC | 1545 | 34 | 1182 | tss | 0.840915 | 0.970906 | TP73; WRAP73 (margin: TP73; WRAP73; RP5-1092A11.5) | 1.57 | 7.07e-03 | 3.19e-02 |
|  | 19 | 291986 | 292498 | PDFSTATSENSEMBLUCSC | 513 | 12 | 20270 | tss;island | 0.925731 | 0.973256 | PPAP2C (margin: VN2R11P; PPAP2C) | 1.56 | 4.96e-04 | 6.47e-03 |
|  | 6 | 32819858 | 32821742 | PDFSTATSENSEMBLUCSC | 1885 | 54 | 34155 | gene | 0.013181 | 0.032161 | PSMB9; TAP1 (margin: XXbac-BPG246D15.8; PSMB9; PSMB8; TAP1) | 1.54 | 6.31e-03 | 3.19e-02 |
|  | 6 | 33130918 | 33132727 | PDFSTATSENSEMBLUCSC | 1810 | 30 | 34193 | gene;island | 0.498822 | 0.730910 | COL11A2 (margin: COL11A2) | 1.54 | 4.76e-03 | 2.73e-02 |
|  | 16 | 88717464 | 88717989 | PDFSTATSENSEMBLUCSC | 526 | 11 | 15410 | tss | 0.595808 | 0.228342 | CYBA; MVD (margin: IL17C; SNAI3-AS1; CYBA; MVD) | 1.53 | 1.04e-02 | 3.96e-02 |
|  | 7 | 27197455 | 27198429 | PDFSTATSENSEMBLUCSC | 975 | 10 | 38354 | island | 0.897687 | 0.985365 | HOXA-AS3; RP1-170O19.21; HOXA7 (margin: HOXA-AS3; RP1-170O19.21; HOXA-AS4; HOXA3; HOXA6; HOXA7; HOXA9; RP1-170O19.20; MIR196B; HOXA10) | 1.53 | 1.15e-02 | 4.29e-02 |
|  | 8 | 143859143 | 143859410 | PDFSTATSENSEMBLUCSC | 268 | 5 | 39469 | gene;tss;island | 0.940767 | 0.982508 | LYNX1 (margin: RP11-706C16.7; RP11-706C16.8; LYNX1; LY6D) | 1.53 | 7.94e-03 | 3.19e-02 |
|  | 15 | 95869918 | 95870475 | PDFSTATSENSEMBLUCSC | 558 | 9 | 14484 | island | 0.867446 | 0.956379 | CTD-2536I1.1 (margin: CTD-2536I1.1) | 1.52 | 7.77e-03 | 3.19e-02 |
|  | 6 | 31165290 | 31166174 | PDFSTATSENSEMBLUCSC | 885 | 29 | 35756 | island | 0.008159 | 0.019397 | HCG27; XXbac-BPG299F13.17; XXbac-BPG299F13.14 (margin: HCG27; XXbac-BPG299F13.17; XXbac-BPG299F13.14) | 1.52 | 8.68e-03 | 3.37e-02 |
|  | 4 | 82393069 | 82393369 | PDFSTATSENSEMBLUCSC | 301 | 8 | 30464 | tss | 0.064561 | 0.023399 | RASGEF1B (margin: RASGEF1B) | 1.50 | 6.99e-03 | 3.19e-02 |
|  | 19 | 58220295 | 58220837 | PDFSTATSENSEMBLUCSC | 543 | 10 | 22480 | island | 0.951825 | 0.985694 | AC003006.7; ZNF154 (margin: AC003006.7; ZNF154; ZNF671) | 1.50 | 2.09e-03 | 1.45e-02 |
|  | 7 | 128809100 | 128809442 | PDFSTATSENSEMBLUCSC | 343 | 5 | 38906 | island | 0.909393 | 0.964072 | TSPAN33; RP11-286H14.6 (margin: TSPAN33; Y\_RNA; RP11-286H14.6) | 1.48 | 7.94e-03 | 3.19e-02 |
|  | 16 | 3493336 | 3494155 | PDFSTATSENSEMBLUCSC | 820 | 11 | 15056 | tss;island | 0.294576 | 0.527597 | LA16c-306E5.2; NAA60; ZNF597 (margin: LA16c-306E5.2; NAA60; ZNF597) | 1.47 | 5.67e-06 | 3.54e-04 |
|  | 6 | 33561181 | 33561449 | PDFSTATSENSEMBLUCSC | 269 | 7 | 34910 | tss | 0.971292 | 0.933673 | LINC00336 (margin: GGNBP1; LINC00336) | 1.46 | 4.08e-03 | 2.47e-02 |
|  | 6 | 33560953 | 33561449 | PDFSTATSENSEMBLUCSC | 497 | 9 | 36042 | island | 0.978411 | 0.950541 | LINC00336 (margin: GGNBP1; LINC00336) | 1.46 | 7.77e-03 | 3.19e-02 |
|  | 6 | 32809263 | 32809657 | PDFSTATSENSEMBLUCSC | 395 | 10 | 34148 | gene;island | 0.982997 | 0.951179 | PSMB8 (margin: XXbac-BPG246D15.8; PSMB9; TAP2; PSMB8; TAP1) | 1.46 | 2.17e-05 | 8.94e-04 |
|  | 16 | 82660206 | 82660873 | PDFSTATSENSEMBLUCSC | 668 | 14 | 16129 | island | 0.195635 | 0.433019 | CDH13 (margin: CDH13) | 1.45 | 1.43e-03 | 1.36e-02 |
|  | 14 | 54976234 | 54976718 | PDFSTATSENSEMBLUCSC | 485 | 10 | 12858 | island | 0.004687 | 0.010315 | CGRRF1 (margin: CGRRF1) | 1.45 | 1.15e-02 | 4.29e-02 |
|  | 12 | 56511647 | 56511953 | PDFSTATSENSEMBLUCSC | 307 | 8 | 9784 | tss | 0.004145 | 0.007093 | RPL41; ZC3H10; ESYT1; RP11-603J24.6; RP11-603J24.5 (margin: RP11-603J24.9; PA2G4; RPL41; ZC3H10; ESYT1; RP11-603J24.17; RP11-603J24.6; RP11-603J24.5) | 1.44 | 4.66e-03 | 2.68e-02 |
|  | 7 | 83824255 | 83824481 | PDFSTATSENSEMBLUCSC | 227 | 7 | 37679 | tss | 0.014877 | 0.100027 | SEMA3A (margin: SEMA3A) | 1.43 | 4.08e-03 | 2.47e-02 |
|  | 6 | 32064491 | 32065211 | PDFSTATSENSEMBLUCSC | 721 | 29 | 34047 | gene;island | 0.809616 | 0.892447 | TNXB; ATF6B (margin: TNXB; ATF6B) | 1.43 | 5.87e-03 | 3.19e-02 |
|  | 5 | 169659618 | 169659950 | PDFSTATSENSEMBLUCSC | 333 | 10 | 33377 | island | 0.911544 | 0.978889 | C5orf58 (margin: C5orf58) | 1.42 | 1.15e-02 | 4.29e-02 |
|  | 14 | 102027514 | 102027797 | PDFSTATSENSEMBLUCSC | 284 | 6 | 12636 | tss;island | 0.686037 | 0.454886 | DIO3; DIO3OS (margin: DIO3; DIO3OS) | 1.41 | 2.16e-03 | 1.45e-02 |
